# Runs of homozygosity in Swiss goats reveal genetic changes associated with domestication and modern selection

**DOI:** 10.1186/s12711-022-00695-w

**Published:** 2022-01-24

**Authors:** Heidi Signer-Hasler, Jan Henkel, Erika Bangerter, Zafer Bulut, The VarGoats Consortium, Cord Drögemüller, Tosso Leeb, Christine Flury

**Affiliations:** 1 School of Agricultural, Forest and Food Sciences, Bern University of Applied Sciences, 3052 Zollikofen, Switzerland; 2 Institute of Genetics, Vetsuisse Faculty, University of Bern, 3001 Bern, Switzerland; 3 Swiss Goat Breeding Association SZZV, Schützenstrasse 10, 3052 Zollikofen, Switzerland; 4 Department of Biochemistry, Faculty of Veterinary Medicine, Selcuk University, Konya, Turkey

## Abstract

**Background:**

The domestication of goat (*Capra hircus*) started 11,000 years ago in the fertile crescent. Breed formation in the nineteenth century, establishment of herd books, and selection for specific traits resulted in 10 modern goat breeds in Switzerland. We analyzed whole-genome sequencing (WGS) data from 217 modern goats and nine wild Bezoar goats (*Capra aegagrus*). After quality control, 27,728,288 biallelic single nucleotide variants (SNVs) were used for the identification of runs of homozygosity (ROH) and the detection of ROH islands.

**Results:**

Across the 226 caprine genomes from 11 populations, we detected 344 ROH islands that harbor 1220 annotated genes. We compared the ROH islands between the modern breeds and the Bezoar goats. As a proof of principle, we confirmed a signature of selection, which contains the *ASIP* gene that controls several breed-specific coat color patterns. In two other ROH islands, we identified two missense variants, *STC1:*p.Lys139Arg and *TSHR*:p.Ala239Thr, which might represent causative functional variants for domestication signatures.

**Conclusions:**

We have shown that the information from ROH islands using WGS data is suitable for the analysis of signatures of selection and allowed the detection of protein coding variants that may have conferred beneficial phenotypes during goat domestication. We hypothesize that the *TSHR*:p.Ala239Thr variant may have played a role in changing the seasonality of reproduction in modern domesticated goats. The exact functional significance of the *STC1*:p.Lys139Arg variant remains unclear and requires further investigation. Nonetheless, *STC1* might represent a new domestication gene affecting relevant traits such as body size and/or milk yield in goats.

**Supplementary Information:**

The online version contains supplementary material available at 10.1186/s12711-022-00695-w.

## Background

The domestication of goat (*Capra hircus*) started 11,000 years ago in the fertile crescent [1, 2]. Based on archaeological caprine remains (around 8200 years BC) from the Zagros mountains (eastern Fertile Crescent, western Iran) and by combining archaeozoological and archaeogenomic approaches, Daly et al. [3] reported the existence of two distinct clusters of goats: one with domestic affinity and one with wild affinity. These findings indicate, that these goats from the late ninth/early eighth millennium BC were genetically diverse, already distinct from wild goats and ancestral to later domestic goats [3]. A previous study [4], based on sequence data from 83 ancient goats, has already demonstrated that multiple divergent ancient wild goats were domesticated in a dispersed process leading to genetically and geographically distinct Neolithic goat populations. Based on these results, the assumption that animal domestication required the establishment and maintenance of reproductive isolation between wild and domestic populations is questioned [1].

The early Neolithic goat populations contributed in varying proportions to modern goats in Asia, Africa and Europe. Modern goat breeds from Europe clustered together with the western Neolithic samples [4]. After domestication, goats spread through the Danubian and Mediterranean corridors into Europe [5]. The high Y-chromosome differentiation between Swiss and Southern European breeds might be due to the post-domestication spread of two different Near Eastern genetic stocks [6]. Since the eighteenth century, the formation of goat breeds in Europe started via morphological standardization and systematic selection to improve production traits. This led to closed breeds with small effective population sizes and remarkable levels of linkage disequilibrium (LD) at the genomic level [7]. Breed formation followed by different breeding goals and selection programs resulted in 10 modern Swiss goat breeds [8] with different phenotypic properties (Table 1).

**Table 1:** Overview of the phenotypic characteristics of the 11 investigated caprine populations

Breed	Shoulder height (cm, ♂/♀)	Body weight (kg, ♂/♀)	Coat color (predominant agouti allele)
Appenzell goat (APZ)	85/75	75/55	White (*A*^*Wt*^)
Grisons Striped goat (BST)	85/75	75/55	Black with Swiss markings (*A*^*sm*^)
Capra Grigia (CAG)	87/80	65/45	Grey/Roan (*A*^*bz*^)
Chamois Colored goat (GFG)	85/75	75/55	Reddish brown with black badgerface pattern (*A*^*b*^)
Nera Verzasca (NER)	90/80	80/60	Solid black (*A*^*bz*^)
Peacock goat (PFA)	85/75	85/60	Black and white peacock pattern (*A*^*pc*^)
Saanen goat (SAN)	90/80	85/60	White (*A*^*Wt*^)
St. Gallen Booted goat (STG)	80/72	70/45	Reddish brown with black badgerface pattern (A^b^)
Toggenburg goat (TOG)	85/75	75/55	Brown with Swiss markings (*A*^*sm*^)
Valais Blackneck goat (VAG)	85/75	75/55	Black cranial half and white caudal half (*A*^*bz*^)
Bezoar goat (*Capra aegagrus*, BEZ)^a^	100/90	85/60	Complex pattern (wildtype, Bezoar) (*A*^*bz*^)

^a^Ref. [45]

Large-scale genomic data is essential to understand changes in the genetic make-up of goats due to domestication, breed formation or recent selection. In a previous study, we used whole-genome sequencing (WGS) data from pooled DNA (pool-seq) for the identification of signatures of selection among the 10 goat breeds from Switzerland and 10 additional breeds originating from Pakistan and Africa [9]. This study revealed signatures of selection at the *ASIP* locus that harbors four copy number variants (CNV), which control specific pigmentation patterns [white or tan (*A*^*Wt*^), Swiss markings (*A*^*sm*^), badgerface (*A*^*b*^) and peacock (*A*^*pc*^)] [9].

In the current study, we analyzed individual whole-genome sequence data from 10 Swiss goat breeds and Bezoar goats (*Capra aegagrus*) to overcome the potential limitations of pool-seq data as well as of low to medium density SNP-data. We investigated population structure, runs of homozygosity (ROH) and ROH islands [10, 11] as potential signatures of selection at the nucleotide level. Finally, we searched for variants that may be causally related to domestication in goats by screening individual variants in candidate genes that harbor ROH islands with extremely divergent genotype frequencies between goat populations.

## Methods

### Individual whole-genome sequences

This study is based on data from 217 modern goats and nine wild Bezoar goats (Tables 1, 2). We used 119 previously reported whole-genome sequences from five Swiss breeds (Appenzell goat (APZ), Grisons Striped goat (BST), Peacock goat (PFA), St. Gallen Booted goat (STG) and Valais Blackneck goat (VAG) [9] and eight whole-genome sequences for Chamois Colored goat and Saanen goat from Switzerland that were kindly shared by the VarGoats consortium (http://www.goatgenome.org/vargoats.html; [12]). In addition, we generated new WGS data for 90 goats from five Swiss goat breeds (Capra Grigia (CAG), Chamois Colored goat (GFG), Nera Verzasca (NER), Saanen goat (SAN), Toggenburg goat (TOG)) and nine wild Bezoar goats (BEZ) sampled in Turkey. Illumina TruSeq PCR-free DNA libraries were prepared, and 2 $\times$ 150 bp reads were collected on an Illumina NovaSeq 6000 instrument as described in [9]. All accession numbers are in Additional file 1: Table S1.

**Table 2:** Summary of the number of sequenced animals per population, average genome coverage, average nucleotide diversity, average ROH estimates (number, length) and maximum length of ROH per breed

Breed	Number of animals	Average genome coverage	Average nucleotide diversity(π)	Average number of ROH	Average length of ROH^a^ (kb)	Maximum length of ROH (kb)
Appenzell goat (APZ)	24	18.76	1.43 × 10^–3^	1328	433	21,851
Grisons Striped goat (BST)	24	21.34	1.61 × 10^–3^	1174	321	21,564
Capra Grigia (CAG)	12	22.58	1.62 × 10^–3^	1376	246	23,157
Chamois Colored goat (GFG)	19	20.65	1.60 × 10^–3^	1321	292	20,806
Nera Verzasca (NER)	24	17.11	1.57 × 10^–3^	1436	299	33,837
Peacock goat (PFA)	24	20.26	1.59 × 10^–3^	1377	263	17,846
Saanen goat (SAN)	19	17.56	1.52 × 10^–3^	1382	361	33,602
St. Gallen Booted goat (STG)	24	16.95	1.51 × 10^–3^	1327	365	20,689
Toggenburg goat (TOG)	24	19.46	1.43 × 10^–3^	1375	427	23,486
Valais Blackneck goat (VAG)	23	25.97	1.46 × 10^–3^	1399	413	17,566
Bezoar goat (*Capra aegagrus*, BEZ)	9	11.55	1.81 × 10^–3^	2018	323	25,268
Total	226	19.60	1.72 × 10^–3^	1374	347	33,837

^a^Average number of segments estimated based on all segments per breed

The cleaned reads were mapped to the goat reference genome ARS1 [13] with the Burrows-Wheeler Aligner (BWA-MEM) algorithm version 0.7.13 [14] using the “-M” flag to mark shorter alignments as secondary. For a detailed description of whole-genome re-sequencing of individual goats and variant calling procedures, please refer to [9].

For quality control, we considered only high quality (“pass”), autosomal, polymorphic biallelic SNVs with call rates ≥ 90% across all breeds. The minimal coverage was set to eightfold (see Additional file 1: Table S1). To account for remaining differences in genotyping quality mainly between the Swiss and Bezoar goat samples, an additional filter step was implemented by excluding SNVs with call rates < 90% within breed. In total, 27,728,288 SNVs and 226 goats from 11 populations (Table 2) and (see Additional file 1: Table S1) remained for analysis. Phenotypic characteristics of the different breeds are in Table 1.

### Population structure

LD pruning was performed prior to multidimensional scaling (MDS) and admixture analyses [15]. Data were pruned using the Plink1.9 software [16] and the following setting: --indep-pairwise 5000 500 0.80. In total, 4,831,063 SNVs remained in the LD-pruned dataset used for MDS and admixture analyses. For MDS analysis, genetic distances were computed using the Plink1.9 --genome --cluster --mds-plot options. The software Admixture version 1.3.0 was used for admixture analysis [17]. The optimal number of clusters was determined by adding the cv-flag. Within this analysis, the number of clusters was increased from 1 to 15, and the *k* with the lowest cross-validation error was used for selecting the optimal number of clusters for the genotypes under investigation (see Additional file 2: Fig. S1). The software Distruct [18] was used for the graphical presentation of each cluster assignment, increasing *k* from 2 to 10. The NeighborNet-graph was drawn based on pairwise F_ST_ values using the SplitsTree4 software [19]

### Runs of homozygosity

The Plink1.9 --homozyg option [16] was used for the identification of ROH. According to the guidelines from Meyermans et al. [20] we did not apply LD-pruning prior ROH detection. To minimize false positive ROH results, the Plink standard parameters were validated by comparing ROH estimates with the maximal genome coverage reached. The maximal genome coverage equals the proportion of the maximum detectable ROH length of a fully homozygous individual over the length of the total autosomal genome and was derived for varying sets of Plink parameters [20].

When using WGS data, the marker density is no longer limiting, but the number of heterozygous SNVs per window needs to be checked to account for possible erroneous heterozygous genotype calls that incorrectly may break a long ROH [21]. We varied the option --homozyg-window-het from 1 to 5. In addition, the minimum length of a ROH was evaluated by setting the option --homozyg-kb to 100, 300 and 1000 kb. One-Mb ROH-segments indicate that the homozygosity originated from a common ancestor 50 generations ago, whereas 100-kb segments allow to trace back common ancestors 500 generations ago [22]. For concise derivation of ROH islands being related to domestication, we finally allowed three heterozygous SNVs per window and a minimal ROH length of 100 kb for the final analysis.

Inbreeding coefficients (${F}_{ROH}$) for each individual were calculated according to McQuillan et al. [23]:

$${F}_{ROH}=\sum \frac{{L}_{ROH}}{{L}_{AUTO}},$$

where ${L}_{ROH}$ stands for the total length of an individual’s genome fraction within ROH and ${L}_{AUTO}$ stands for the total length of the autosomal genome spanning SNV positions and equals 2,465,617 kb in the current study.

### ROH islands and detection of candidate causal variants

A ROH island was defined as a genomic region for which at least 80% of the animals in a predefined group had consecutive SNVs in a ROH. In a first step, we derived ROH islands by considering all 217 individuals from the modern goat breeds, regardless of their breed assignment (see Additional file 2: Fig. S2). In a second step, we derived ROH islands using the information from the 11 populations separately (see Additional file 2: Fig. S2). The gene annotation in the identified ROH islands was taken from the NCBI annotation release 102 of the ARS1 reference genome assembly. Average proportions of homozygosity for the SNVs in these genes were calculated for each population (see Additional file 2: Fig. S2).

Finally, the complete list of annotated genes in ROH islands found for the 11 populations was visually inspected and eight genes with previously described functions that are potentially relevant to domestication and/or selection in livestock populations and divergent genotype frequencies between populations were arbitrarily selected (see Additional file 1: Tables S2 and S3). We hypothesized that these genes might have been involved in the domestication process. The genotypes of all variants in the eight genes were tabulated and compared within and among the 226 individuals from the 11 populations (Table 4).

## Results

### Population structure

**Fig. 1:**
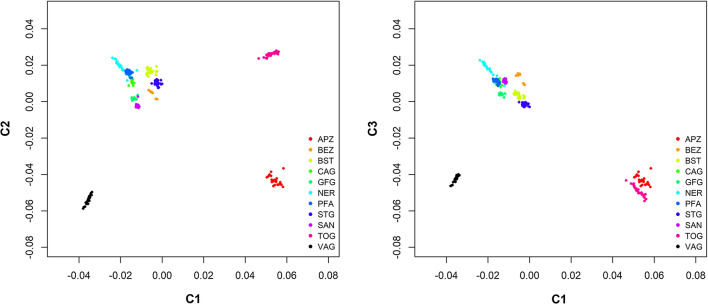
Population structure of the 11 caprine populations analysed. Multidimensional scaling plot representing population structure based on the first (C1) and second (C2) components (left) and on the first (C1) and third (C3) components (right). Both plots are based on pruned genotypes at 4,831,063 SNVs from 226 sequenced caprine genomes

The first two components of the multi-dimensional scaling (MDS) plot separates APZ, TOG and VAG from each other and from the cluster of all other populations including the Bezoar goats (Fig. 1). Interestingly, BEZ clusters loosely together with modern breeds, although separate clusters are discernible (Fig. 1). An obvious genetic uniqueness of APZ, TOG and VAG is further supported by the results from the admixture analyses and from the NeighborNet-graph based on pairwise F_ST_ (see Additional file 2: Figs. S3 and S4). Based on the results for *k* = 8 [optimal number of clusters, (see Additional file 2: Fig. S1)] BEZ and SAN are not differentiated, whereas CAG and GFG both appear to be highly admixed populations. At *k* = 10, all animals, except the 12 CAG goats, are separated according to their breed assignment (see Additional file 2: Fig. S3). Six NER individuals show remarkable levels of admixture – one individual with STG (*k* = 4 to 10,) and five individuals with PFA (*k* = 8 to 10) (see Additional file 2: Fig. S3). As recently described for other modern goat populations [24], introgression might be a result of the traditional reunion of animals from different herds and breeds on alpine pastures during the summer season.

### Runs of homozygosity and F_ROH_

Across the 226 animals, we found an average of 1374 ROH per individual with an average length of 347 kb (Table 2). The average number of ROH ranged from 1174 (BST) to 2018 (BEZ). The average length of ROH per breed ranged from 246 kb (CAG) to 433 kb (APZ). The minimum length of ROH was predefined at 100 kb (see “Methods” section). The maximum length of ROH reached 33,837 kb in a NER goat. For each breed, the fraction of ROH for five length classes (0.1 to 0.3 Mb, 0.3 to 0.5 Mb, 0.5 to 1.0 Mb, 1.0 to 5.0 Mb and > 5.0 Mb) is shown in Additional file 2: Fig. S5.

**Fig. 2:**
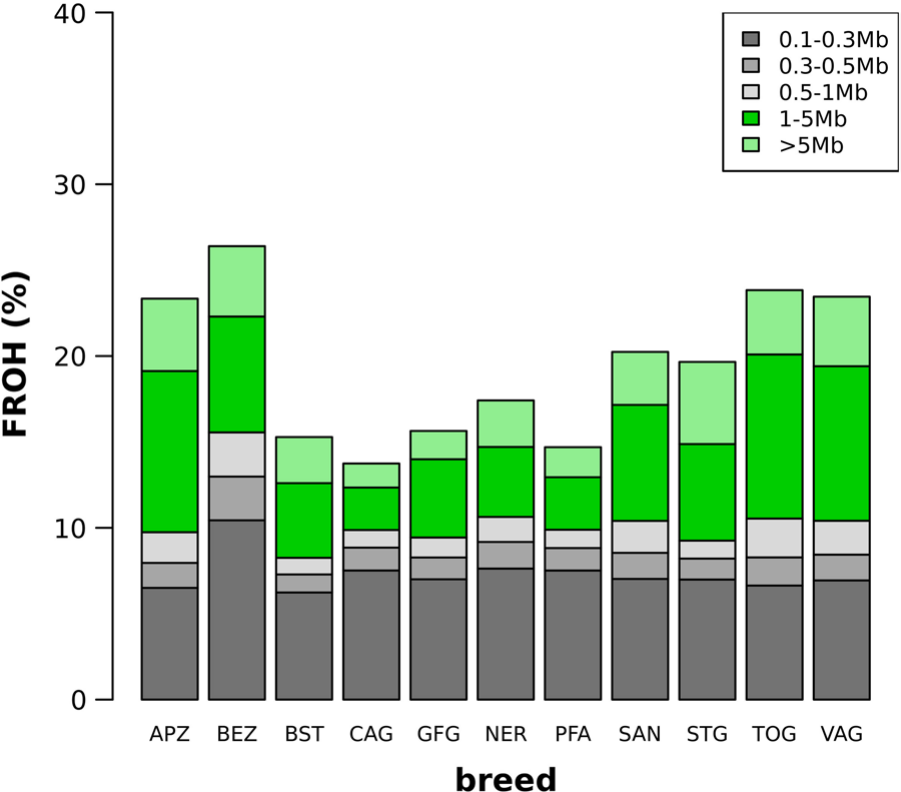
Genomic inbreeding (F_ROH_) in 11 caprine populations. Average F_ROH_ per breed and relative contribution of the five length classes (0.1 to 0.3 Mb, 0.3 to 0.5 Mb, 0.5 to 1.0 Mb, 1.0 to 5.0 Mb and > 5.0 Mb)

The autosomal average genomic inbreeding (F_ROH_) ranged from 13.74% (CAG) to 26.39% (BEZ) (Fig. 2). The relative contribution of the five length classes to average F_ROH_ is indicated in Fig. 2. For APZ, STG, TOG and VAG, the segments longer than 1 Mb exceeded 50% of all ROH, while the fraction of ROH segments of this length ranged from 40 to 50% in BEZ, BST, GFG and SAN, and was lower than 40% in CAG, NER and PFA. If the genome of an individual contains ROH segments between 1 to 5 Mb, we conclude, that the individuals’ ROH originated from common ancestors between 10 to 50 generations ago [22]. Assuming an approximate generation interval of three years for modern goats, segments 1 to 5 Mb long describe genomic inbreeding originating from a common ancestor 30 to 150 years ago.

### ROH islands in modern goat breeds

**Fig. 3:**
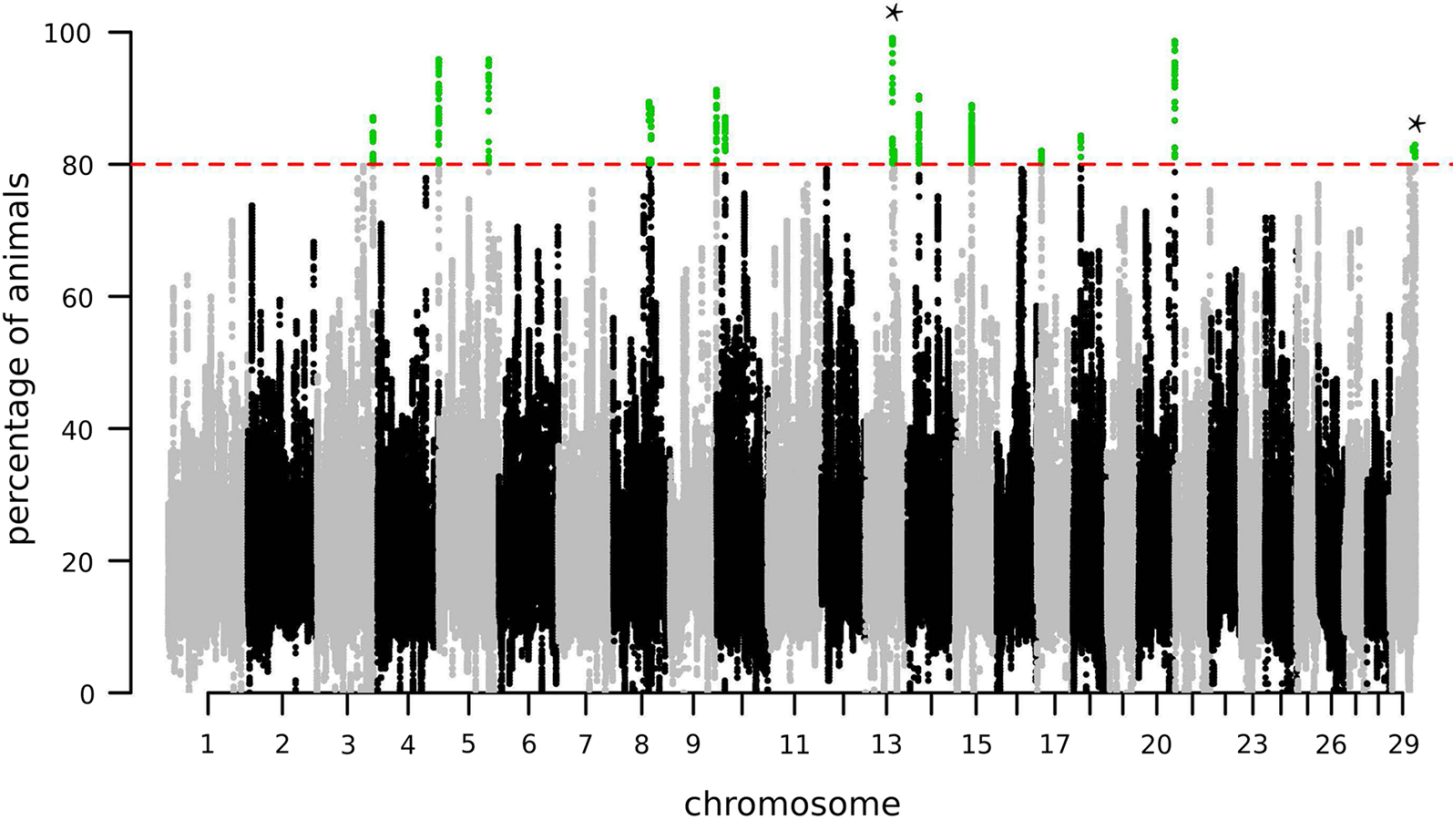
Genome-wide detection of 15 ROH islands. Manhattan plot representing the average fraction of the 217 individuals of the modern goat breeds having a given SNV in a ROH. The threshold (red dashed line) was set to 80% and SNVs defining the ROH islands are visualized in green color. Autosomal regions harboring two ROH islands are indicated with an asterisk

Regions, for which more than 80% of the animals of a given group were homozygous, were defined as ROH islands. In a first step, we derived ROH islands from the 217 combined individuals of the modern breeds irrespective of their breed assignment (see Additional file 2: Fig. S2). Of the 27,728,288 SNVs that passed quality control, 27,647,288 (99.7%) are located in an ROH in at least one animal, and among these, 10,530 SNVs (0.04%) exceeded the threshold of 80% among the 217 individuals from modern breeds. These SNVs defined 15 ROH islands on 12 chromosomes that harbor 53 annotated genes (Fig. 3, Table 3) and (see Additional file 2: Fig. S2). To disentangle ROH islands that are potentially related to domestication, we compared the average proportions of individuals having the SNVs in an ROH in the modern breeds sample (N = 217) to the average proportion in Bezoar goats (N = 9) (Table 3). In nine of the 15 ROH islands, the proportion of homozygous animals in BEZ was lower than 55%. The remaining six ROH islands with a high homozygosity in modern breeds and wild Bezoars might be involved in the control of basic functions that are not related to domestication.

**Table 3:** Description of the 15 ROH islands shared among the 217 individuals from the modern breeds

Chr	Start pos	End pos	Average fraction modern breeds	Average fraction Bezoar	Genes annotated in ROH regions
*3*	*110,837,228*	*110,952,170*	*84.94*	*21.28*	*CD84, LOC102170372, SLAMF1*
*5*	*36,289*	*216,534*	*88.58*	*53.86*	*No genes*
*5*	*98,924,460*	*99,173,351*	*94.59*	*33.57*	*LOC108636060, LOC102183506*
*8*	*70,737,252*	*74,882,654*	*86.15*	*42.62*	*STC1, LOC102183600, APTX, DNAJA1, SMU1, B4GALT1, SPINK4, BAG1, CHMP5, NFX1, AQP7, AQP3, NOL6, UBE2R2, UBAP2, DCAF12*
9	91,295,758	91,426,085	86.01	83.13	*DLL1, FAM120B*
*10*	*17,036,648*	*17,211,934*	*84.75*	*27.09*	*PROX2, YLPM1, FCF1*
13	53,007,666	53,319,681	95.31	73.16	*MYT1, OPRL1, LKAAEAR1, RGS19, TCEA2, PRPF6, SAMD10, DNAJC5, TPD52L2, ZBTB46*
*13*	*56,416,469*	*56,517,376*	*80.75*	*29.76*	*ZNF831*
*14*	*22,802,897*	*22,937,158*	*82.65*	*33.03*	*ZFPM2*
*15*	*32,098,192*	*32,367,622*	*84.46*	*50.56*	*RHOG, STIM1, RRM1, LOC108637588, LOC102177547*
17	8,865,949	8,935,273	81.32	88.89	*LOC108637850, LOC102177554*
18	15,526,880	15,552,439	82.90	88.89	*CBFA2T3*
*20*	*71,475,167*	*71,668,860*	*95.43*	*45.68*	*SLC9A3, EXOC3, AHRR*
29	45,996,028	46,047,264	82.44	74.07	*PTDSS2, ANO9, IFITM5*
29	50,476,146	50,541,044	81.81	88.89	*LOC102176024*
Total			87.00	44.64	

Chr: chromosome number; Start pos: start position of each ROH island; End pos: end position of each ROH island;

Average fraction for the individuals of the modern breeds sample (N = 217) and average fraction for the individuals from wild bezoar (N = 9)

Rows entirely in italic characters indicate ROH islands that are only seen in modern breeds, and not in Bezoar goats (homozygous bezoar goats < 55%)

In a second step, we derived ROH islands separately for each of the 11 populations (see Additional file 2: Fig. S2). We found 407,202 SNVs (1.5%) in ROH islands (see Additional file 3: Fig. S6), which spanned 344 regions harboring 1220 annotated genes (see Additional file 1: Tables S2 and S3). This list of 1220 genes (see Additional file 1: Table S3) was visually inspected and eight genes with functions that are potentially relevant to domestication and/or selection in livestock populations and divergent average proportions between the investigated goat populations were arbitrarily selected for a more detailed analysis (Table 4).

**Table 4:** Selection of the eight genes potentially related to domestication and/or selection from the 1220 genes found in ROH islands in at least one of the 11 caprine populations investigated

Coordinates of the selected genes				Average fraction per breed										
Chr	Gene	Start	End	APZ	BEZ	BST	CAG	GFG	NER	PFA	SAN	STG	TOG	VAG
2	*RUNX3*	7,884,247	7,949,392	0.66	0.28	1.00^a^	0.75^a^	0.66	0.42	0.79^a^	1.00^a^	0.51	0.43	0.83^a^
3	*LEPR*	41,024,000	41,180,997	0.61	0.22^b^	0.68	0.68	0.19^b^	0.88^a^	0.35	0.41	0.50	0.29	0.28
6	*LCORL*	37,904,351	38,069,012	0.60	0.56	0.61	0.81^a^	0.58	0.52	0.80^a^	0.93^a^	0.68	0.37	0.53
8	*STC1*	70,791,218	70,806,076	0.96^a^	0.22^b^	0.92^a^	1.00^a^	0.84^a^	0.88^a^	0.96^a^	0.84^a^	0.92^a^	0.79^a^	0.83^a^
10	*TSHR*	10,656,656	10,833,540	0.58	0.59	0.71^a^	0.92^a^	0.53	0.50	0.54	0.74	0.96^a^	0.61	0.70
13	*ASIP*	63,228,709	63,249,542	0.21^b^*	0.11^b^	0.83^a^	0.17^b^	0.39*	0.25	0.34*	0.16^b^*	0.41*	0.84^a^	0.51
20	*SLC9A3*	71,521,733	71,560,687	1.00^a^	0.44	0.98^a^	1.00^a^	1.00^a^	1.00^a^	1.00^a^	0.84^a^	1.00^a^	0.97^a^	1.00^a^
29	*MUC6*	46,244,530	46,267,909	0.46	1.00^a^	0.83^a^	0.83^a^	0.63	0.92^a^	0.71	0.68	0.42	0.71	0.96^a^

Average fraction per breed: fraction of individuals for which all intragenic SNVs are within a ROH, and for each population. ^a^Gene fractions, higher than 0.75. ^b^Gene fractions lower than 0.25

^*^Goats from these five breeds are nearly fixed for specific *ASIP* alleles that control breed-specific coat color patterns ([9] and Table 1). However, the vcf-files from Illumina whole-genome sequence data contain a large number of erroneous heterozygous genotype calls because these goats carry amplified CNV alleles with some nucleotide differences between the individual copies. Therefore, due to the technical limitations of short read sequencing, the ROH islands at *ASIP* are not detected in all of these five breeds

### Identification of protein coding variants in the *STC1* and *TSHR* genes

**Fig. 4:**
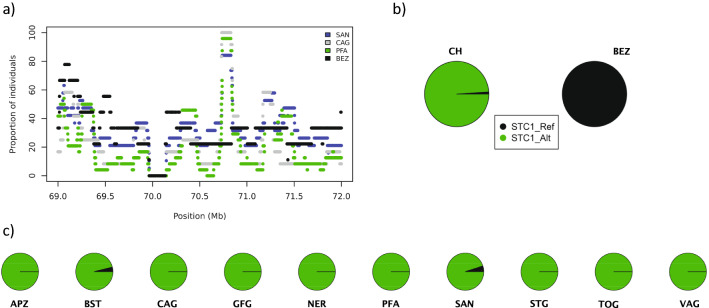
Details of the *STC1*: p.Lys139Arg variant. **a** Fraction of individuals having the tested SNV in a ROH is given for Bezoars and three modern goat breeds (SAN, CAG, PFA). **b** Allele frequencies of the *STC1*:p.Lys139Arg variant in domesticated goats (CH) and in Bezoars in (BEZ). **c** Allele frequencies for each of the 10 modern goat breeds

The eight genes listed in Table 4 were screened for protein-changing variants. The *ASIP* gene was chosen as a positive control since we previously reported functional variants in *ASIP* that control breed-specific coat color patterns and explain the ROH islands [9]. The analysis of the remaining seven genes revealed a missense variant in the *STC1* gene encoding stanniocalcin 1 (Chr8:70,801,961*T* > *C*; c.416*A* > *G*; p.Lys139Arg). All nine sequenced Bezoar goats were homozygous for the reference *T*-allele, while the alternate *C*-allele had a frequency of 99% in Swiss goats (Fig. 4) and (see Additional file 1: Table S4). For further validation of this variant, we compared the allelic frequencies using the 1372 individual goat whole-genome sequences from different geographic origins collected by the VarGoats consortium (see Additional file 1: Table S5). The derived allele is totally absent in the extended sample of wild Bezoars (N = 44) while it has an allelic frequency of 90% in the other European goats (N = 282, excluding the information from animals sampled in Switzerland).

**Fig. 5:**
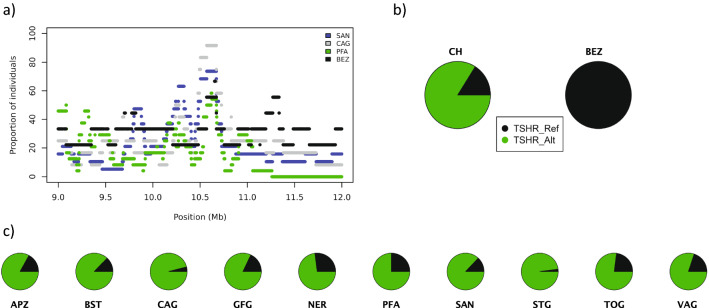
Details of the *TSHR*:p.Ala239Thr variant. **a** Fraction of individuals having the tested SNV in an ROH is given for Bezoar goats and three modern goat breeds (SAN, CAG, PFA). **b** Allele frequencies of the *TSHR*:p.Ala239Thr variant in domesticated goats (CH) and in Bezoar goats (BEZ). **c** Allele frequencies for each of the 10 modern goat breeds

A second missense variant with marked differences in allelic frequencies between the Bezoar and the modern goat breeds was discovered in the *TSHR* gene encoding the thyroid stimulating hormone receptor (Chr10:10,659,811*C* > *T*; c.715*G* > *A*; p.Ala239Thr; Fig. 5 and (see Additional file 1: Table S4). Whereas all the Bezoar goats in our study were homozygous for the reference *C*-allele, the derived *T*-allele had a frequency of 84% in the sample of the 217 modern goats (Fig. 5) and (see Additional file 1: Table S4).

By including the data from the VarGoats consortium, we observed increasing allelic frequencies for the *TSHR* reference allele in relation to the geographic origin of the samples from 0.2 in samples from European breeds to 0.8 and higher in African, Asian, Oceanian and American samples (see Additional file 1: Table S5).

## Discussion

### Population structure

In line with previous studies, we confirm the genetic uniqueness of the three local Swiss goat populations (APZ, TOG and VAG) [8]. Interestingly, they were genetically more distinct than the wild Bezoar goats from the remaining seven modern Swiss goat breeds. The nine Bezoar goats sampled in Turkey were included as an outgroup for the detection of genomic regions that are relevant for domestication. Initially, we expected a more distinct separation of this subgroup from the 10 modern breeds. Daly et al*.* [4] showed that western Neolithic goat derive almost 50% of their ancestry from a divergent source that had affinity to the Anatolian wild population. A current model based on speciation theory proposes that only a limited number of loci (termed ‘islands of domestication’) contribute to the phenotypic differentiation between wild and domestic populations [25]. In addition, the highest average F_ROH_ (26.39%) was observed in BEZ. This is in line with previous studies reporting the highest rates of extreme F_ROH;5 Mb_ in modern wild bezoar from Iran [3] and lower levels of diversity compared to domesticated goats [26]. The differences in the genetic make-up of Bezoar goats and domesticated purebred goats and the high genomic inbreeding in BEZ needs further clarification by sampling more wild Bezoar goats from different geographic origins.

### ROH islands for the derivation of domestication genes

In this study, we retrieved ROH islands from WGS data from 10 modern goat populations and the wild Bezoar as an indicator for signatures of selection [10]. For an unbiased comparison of the proportions of individuals having SNVs in a ROH, it is crucial that the genotyping quality between populations is equal. If the within-population calling rate at the marker level is not considered during quality control, a ROH might not be observed due to impaired genotyping quality in one of the populations. In this situation, the fraction of animals having such SNVs in a ROH will be set to zero, and consequently comparisons with other populations will lead to overestimated differences between breeds.

We presented ROH islands first among the 217 individuals from modern breeds and second at individual breed level. The first approach is more stringent and powerful for the detection of ROH islands being related to domestication while the second is less stringent but enables the recognition of breed-specific ROH islands that potentially arose after domestication.

For the 11 populations, we found ROH islands that harbor 1220 annotated genes (see Additional file 1: Table S3). We report several ROH islands that harbor well-known candidate genes for coat color (*ASIP* [9]), milk production (*ABCG2* [27]), growth (*LEPR*, *LCORL*, and *BMPR2* [28]), fertility (*THSR* and *THSB* [29, 30]) and adaptation to high altitude (*HMOX2* [31]) (Table 3) and (see Additional file 1: Table S3). We confirmed the occurrence of ROH islands that harbor genes such as *HTT* [8, 32, 33], which were previously recognized as signatures of selection in goats but for which the function still needs to be elucidated (see Additional file 1: Table S3).

As a proof of principle, we confirmed the signature of selection on chromosome 13 (62,969,108 bp—63,462,630 bp) that contains the *ASIP* gene. Using pool-sequences and z-transformed heterozygosity scores Henkel et al*.* [9] revealed four CNVs in the *ASIP* locus, that control specific pigmentation patterns (white or tan (*A*^*Wt*^), Swiss markings (*A*^*sm*^), badgerface (*A*^*b*^) and peacock (*A*^*pc*^) in modern goat breeds.

Zheng et al*.* [34] reported a strong signature of selection in the *MUC6* gene and hypothesized, that the almost fixed haplotype in modern goat breeds was introgressed from a lineage close to the West Caucasian tur and is linked to pathogen resistance. In line with this study, we report a ROH island that harbors *MUC6* (Table 4) and (see Additional file 1: Table S3). However, we cannot confirm the fixation of the haplotype in the 10 modern goat populations investigated here (results not shown).

In addition to the signature of selection containing *MUC6*, Zheng et al*.* [34] located the strongest signature of selection on chromosome 15 that harbors the *STIM1* and *RRM1* genes. *STIM1* was previously presented as a specific signature of directional selection in domestic goats [26]. In our data, the region on chromosome 15 (32,098,192–32,367,622 bp) that contains the *RHOG*, *STIM1* and *RRM1* genes was one among the 15 common ROH islands in the 217 individuals from the 10 modern breeds (Fig. 3, Table 3) and (see Additional file 1: Tables S2 and S3). Whether the reduced genetic diversity in modern breeds is the result of selection on behavioral advantages [3, 34], improvement of meat quantity [26] or other phenotypes is not yet clear. The underlying causative variant(s) of this signature of selection and the selected phenotype(s) need to be investigated in future studies.

We present a more detailed analysis of the eight genes that are in ROH islands (Table 3). Two of these genes, *STC1* and *SLC9A3* showed the highest differentiation between BEZ and modern goat breeds (see Additional file 1: Table S3). In all the modern breeds, the fraction of individuals that carry these genes in a ROH is ubiquitously high, while the corresponding proportions in BEZ are remarkably lower (Tables 3 and 4) and (see Additional file 1: Tables S2 and S3). Both of these genes harbor variants that nearly reached fixation in the modern breeds. Interestingly, *SLC9A3* was previously detected in a signature of selection by comparing WGS data from 24 BEZ and 164 domestic modern goats [34]. We speculate that these genes played an important role during differentiation between the wild Bezoar and modern goat breeds and consequently, they could represent new domestication genes.

### Protein coding variants in the *STC1* and *TSHR* genes

Detailed inspection of the *STC1* gene revealed a missense variant (p.Lys139Arg). Modern European goats are nearly fixed for the derived allele, while Bezoar goats are completely fixed for the reference allele (Fig. 4). The data from the VarGoats consortium confirmed the absence of the alternate allele in wild goats (N = 44) and its high frequency in modern European goats (0.90; N = 282) (see Additional file 1: Table S5). The *STC1* gene encodes stanniocalcin-1, a glycoprotein that is involved in different biological processes including angiogenesis, bone and muscle development, and cellular metabolism [35]. Jellinek et al*.* [36] proposed, that *STC1* and *STC2* play a role in calcium and phosphate homoeostasis. Based on immunocytochemistry analysis, it was concluded that the stanniocalcin-1 protein is involved in muscle and bone development of the mouse fetus [37]. The *STC2* gene was suggested to explain size reduction in dogs [28, 38]. Rahmalla et al*.* [39] showed that the Taggar goat breed, which is an achondroplastic dwarf goat breed, was strongly differentiated from other Sudanese goat breeds at 208 genes including *STC1*. In dairy cattle, *STC1* was proposed to be involved in the lactation process and the control of involution of milk-producing tissue [40]. Unfortunately, information describing phenotypic differences between modern goat breeds and wild Bezoar goats is sparse (Table 1) but it is assumed, that the domestication of goats led to a reduction in body size and horns [41] and that milk yield increased [42]. We speculate that the *STC1*:p.Lys139Arg variant may contribute to differences in body size or milk yield between modern goat breeds and wild Bezoar goats. The exact functional role of this variant needs to be validated in future studies.

The ROH island that contains the *TSHR* gene and the *TSHR*:p.Ala239Thr variant raised our interest. A variant in the *TSHR* gene was previously shown to cause reduced seasonality of reproduction in domestic chicken compared to its wild ancestor, the jungle fowl [29]. In sheep, *TSHR* was highlighted in an ancestral signature of selection by Fariello et al*.* [43] and the authors concluded, that sheep raised in temperate climates experience a reproductive cycle under photoperiodic control. All the modern goat breeds investigated here have a seasonal reproductive cycle starting in late August with decreasing day length, followed by a birth season from January till March after 150 days of gestation. In contrast, for wild Bezoar goats, rut is described to last from November until February [44, 45]. Including the information from the VarGoats consortium, we found that the frequencies for the derived ^239^Thr allele were close to 80% in the European goat breeds, while the frequencies were lower than 20% for goats sampled in tropical regions such as Africa, Asia, Oceania and America (see Additional file 1: Table S5). Goats bred in tropical and equatorial regions are subject to less variation in photoperiod and temperature. They display a longer breeding season than those bred in temperate and polar regions, which exhibit more pronounced seasonal effects [46]. As a prominent example, the Boer goat originating from Africa has a non-seasonal reproductive cycle [47, 48]. Only five of the 37 sequences from the Boer individuals collected by the VarGoats consortium carried the alternate allele in a heterozygous state, while the remaining 32 goats were homozygous for the reference allele (see Additional file 1: Table S5). In previous studies, *SOX14*, *NOCT*, *RAI1*, *TH* and *TSHB* were proposed as candidate genes that are linked to the circadian clock and seasonality of reproduction in goats [30, 49]. Based on our results, we propose that this list should be expanded by including the *TSHR* gene. The potential functional impact of the p.Ala239Thr variant on seasonality in goats needs careful validation in additional studies by including breeds from the tropics with documented non-seasonal reproduction.

## Conclusions

We extracted 1220 genes in ROH islands from WGS data of wild Bezoar goats and 10 modern goat breeds, which are likely to represent signatures of selection. The identified ROH islands facilitated the identification of two candidate causative genetic variants that might be involved in the domestication process of goats. We hypothesize that the *TSHR*:p.Ala239Thr variant explains differences in the reproductive cycle in modern goat breeds. The exact function of the *STC1*:p.Lys139Arg variant is not yet clear. *STC1* might represent a new domestication gene that affects body size and/or milk yield in goats. Further analyses including functional evaluation are required to confirm the exact role of these two variants.

## Supplementary Information

**Additional file 1: Table S1.** Accession numbers of the sequenced goats in this study. Information on 226 goats from different breeds and ENA accessions for the sequence data. **Table S2.** 344 ROH islands observed in the 11 caprine populations investigated. Chromosomes and end positions of 1-Mb windows harbouring ROH-regions are provided. Breeds with a ROH-proportion exceeding 80% in this 1-Mb window are indicated with 1. The table on the right gives a summary over the 29 autosomes and the number of ROH islands. **Table S3.** List of the 1220 annotated genes in the regions of ROH islands. For each gene, the chromosome number, the start- and end position, the number of SNVs are given within a gene and that is part of the ROH (X), and also the average proportion of individuals of each population that has the X-SNPs in an ROH. The rows with the eight genes selected for Table 3 are marked in grey. **Table S4.** Information and the 226 individual genotypes for the two protein coding variants in the *STC1* and *TSHR* genes. **Table S5.** Frequencies of the reference alleles at the two coding variants in *STC1* and *TSHR* in samples from the 1372 individual sequences collected by the The VarGoats Consortium different origins.

**Additional file 2: Figure S1.** Development of log cross-validation error (CVE) by increasing the number of *k*, with *k* = 8 determined as the optimal number of clusters. **Figure S2.** Schematic representation of the different steps for the derivation of ROH islands. **Figure S3.** Admixture results for *k* = 2 to 10 and the investigated 11 breeds (APZ = Appenzell goat, BEZ = Bezoar goat, BST = Grisons stripped goat, CAG = Tessin grey goat, GFG = Chamois goat, NER = Nera Verzasca goat, PFA = Peacock goat. SAN = Saanen goat, STG = St. Gallen booted goat, TOG = Toggenburg goat, VAG = Valais goat). The optimal number of clusters (*k* = 8) according to the cross‐validation analysis is indicated in red. **Figure S4.** NeighborNet-graph based on pairwise F_ST_ values. **Figure S5.** Fraction of ROH for the five different length classes 0.1–0.3 Mb, 0.3–0.5 Mb, 0.5–1.0 Mb, 1.0–5.0 Mb and > 5.0 Mb for each of the 11 breeds.

**Additional file 3: Figure S6.** Manhattan plots representing the breed-wise average fraction of goats having a given SNV in a ROH for the 29 autosomes. The threshold (red dashed line) was set to 80%. SNVs that are above the threshold describe ROH islands.

## Data Availability

Sequences associated with this study are deposited at the NCBI Sequence Read Archive https://www.ncbi.nlm.nih.gov/sra under project PRJNA310684 (see Additional file 1: Table S1).
