# Supplementary material for: Runs of homozygosity in Swiss goats reveal genetic changes associated with domestication and modern selection
Source: Genet Sel Evol. 2022 Jan 24;54:6. doi: 10.1186/s12711-022-00695-w (PMC8785455; doi:10.1186/s12711-022-00695-w)
Supplement: Supplementary file 2 — Additional file 2: Figure S1. Development of log cross-validation error (CVE) by increasing the number of k, with k = 8 determined as the optimal number of clusters. Figure S2. Schematic representation of the different steps for the derivation of ROH islands. Figure S3. Admixture results for k = 2 to 10 and the investigated 11 breeds (APZ = Appenzell goat, BEZ = Bezoar goat, BST = Grisons stripped goat, CAG = Tessin grey goat, GFG = Chamois goat, NER = Nera Verzasca goat, PFA = Peacock goat. SAN = Saanen goat, STG = St. Gallen booted goat, TOG = Toggenburg goat, VAG = Valais goat). The optimal number of clusters (k = 8) according to the cross‐validation analysis is indicated in red. Figure S4. NeighborNet-graph based on pairwise FST values. Figure S5. Fraction of ROH for the five different length classes 0.1–0.3 Mb, 0.3–0.5 Mb, 0.5–1.0 Mb, 1.0–5.0 Mb and > 5.0 Mb for each of the 11 breeds. [file 12711_2022_695_MOESM2_ESM.docx]

Figure S1: Development of log cross validation error (CVE) by increasing number of k. k=8 was determined as optimal number of clusters.





Figure S2: Schematic representation of the different steps for the derivation of ROH-islands on the left side based on the incidence >80% among the 217 individuals from modern breeds and on the right side based on the incidence >80% within at least one of the 11 populations and 226 individuals.


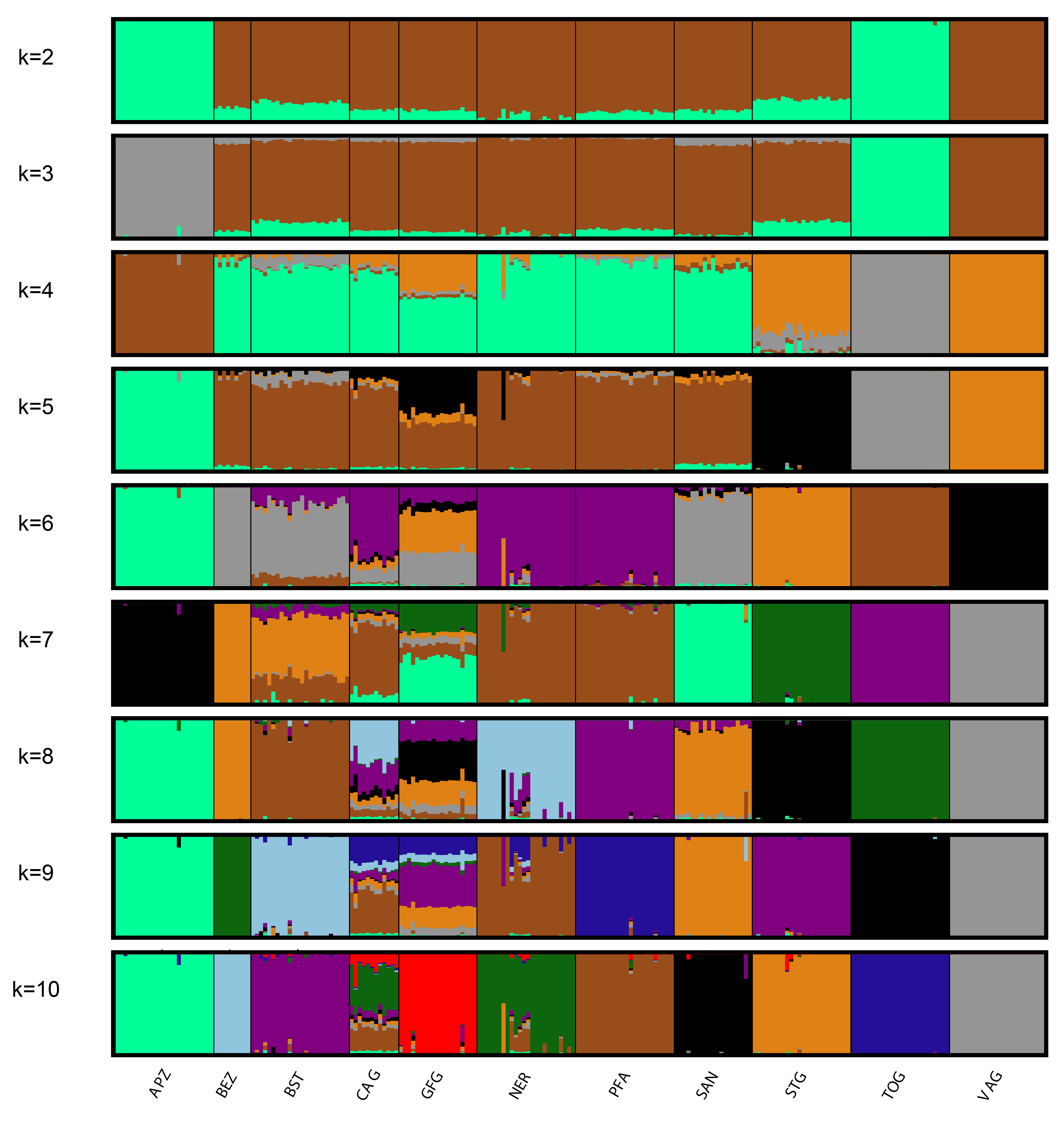


Figure S3: Admixture results for *K*= 2–10 and the investigated 11 breeds (APZ= Appenzell goat, BEZ= Bezoar goat, BST= Grisons stripped goat, CAG= Tessin grey goat, GFG= Chamois goat, NER= Nera Verzasca goat, PFA= Peacock goat. SAN= Saanen goat, STG= St. Gallen booted goat, TOG= Toggenburg goat, VAG= Valais goat). The optimal number of clusters (*k* = 8) according to the cross‐validation analysis is indicated in red.


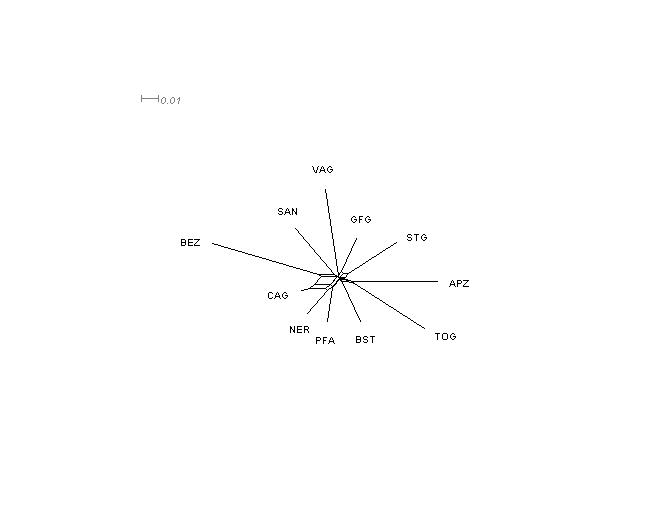


Figure S4: NeighborNet-graph based on pairwise FST.

Figure S5: Fraction of ROHs for 5 different length classes 0.1-0.3 Mb, 0.3-0.5 Mb, 0.5-1.0 Mb, 1.0-5.0 Mb and >5.0 Mb for each of the 11 breeds.
