# Supplementary figures and images for: Runs of homozygosity in Swiss goats reveal genetic changes associated with domestication and modern selection
Source: Genet Sel Evol. 2022 Jan 24;54:6. doi: 10.1186/s12711-022-00695-w (PMC8785455; doi:10.1186/s12711-022-00695-w)

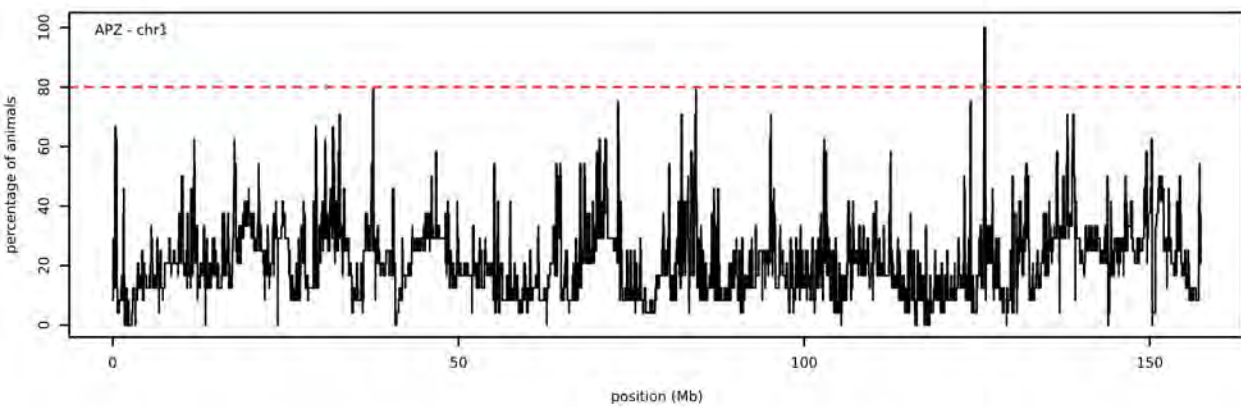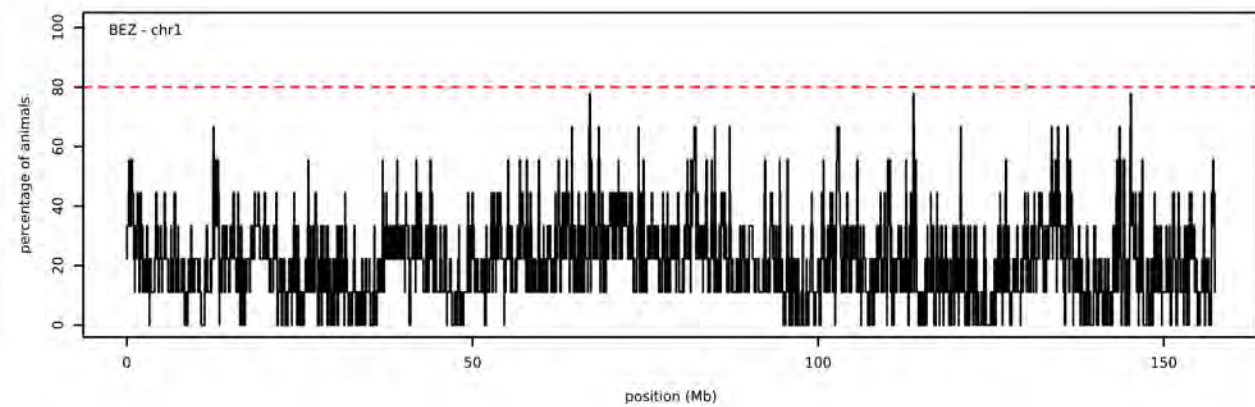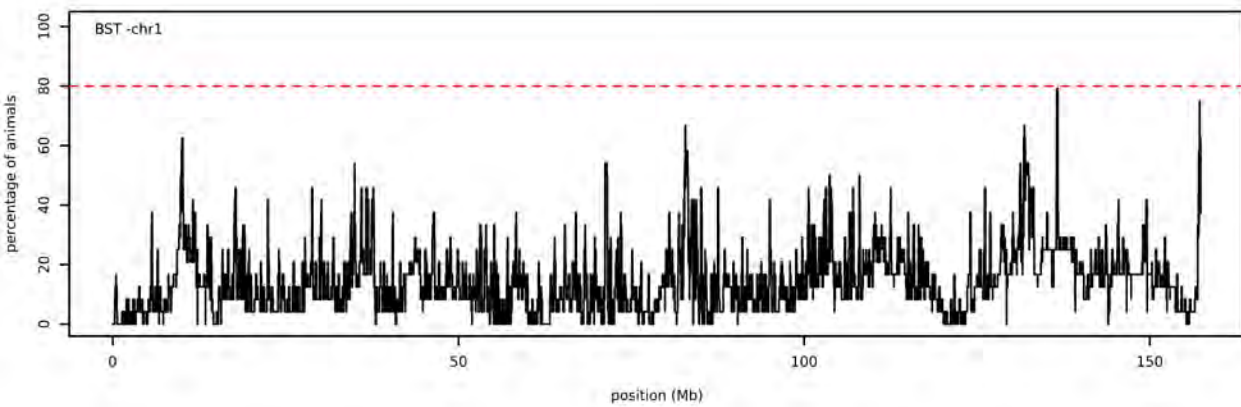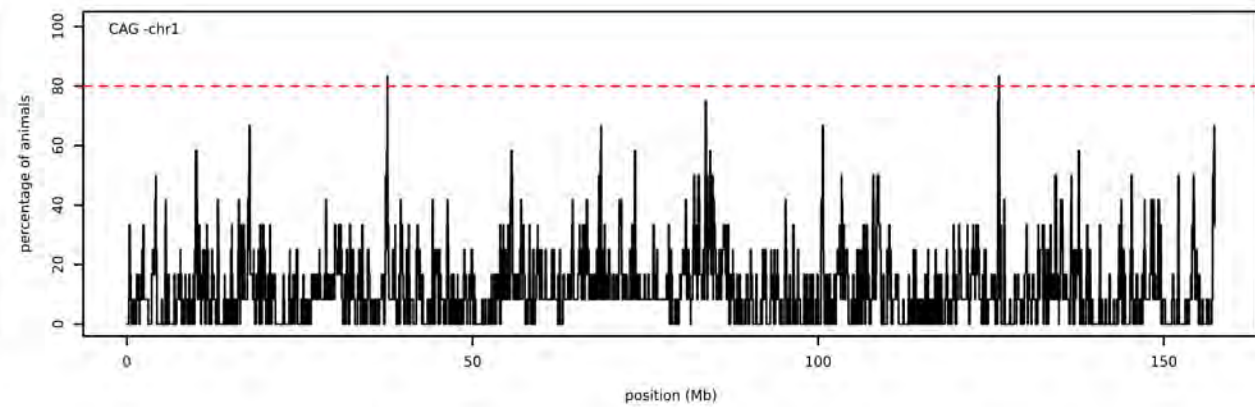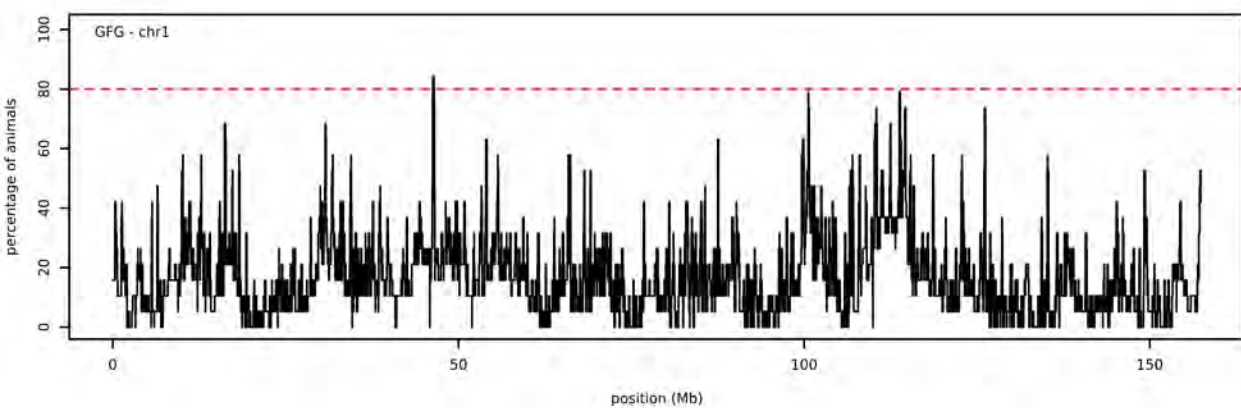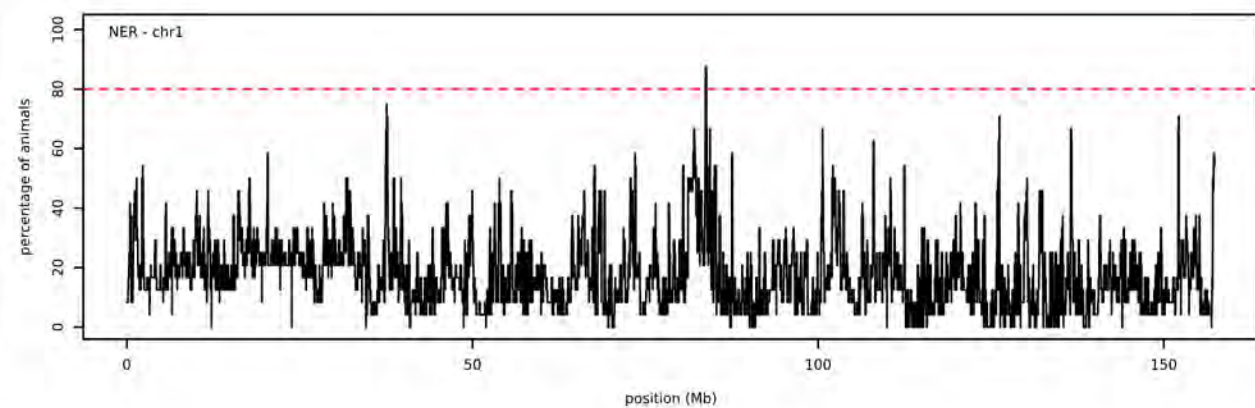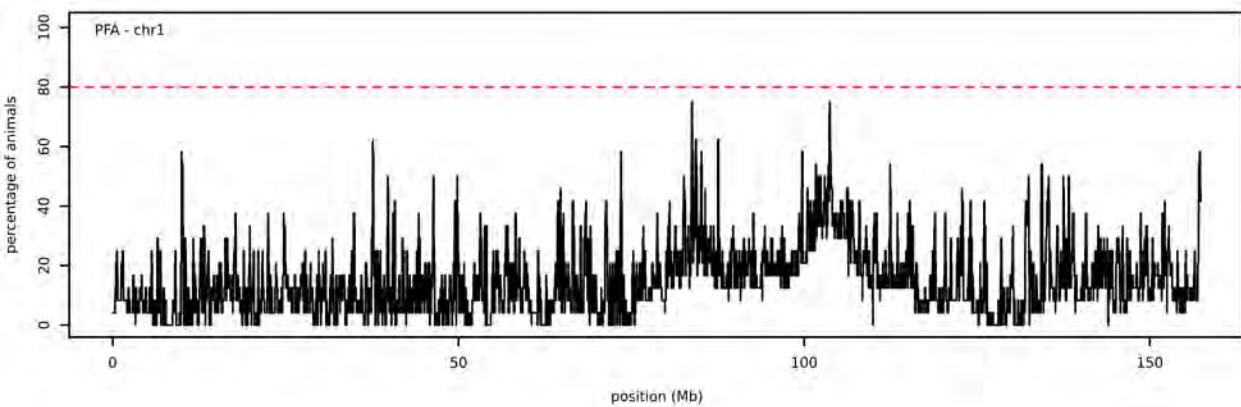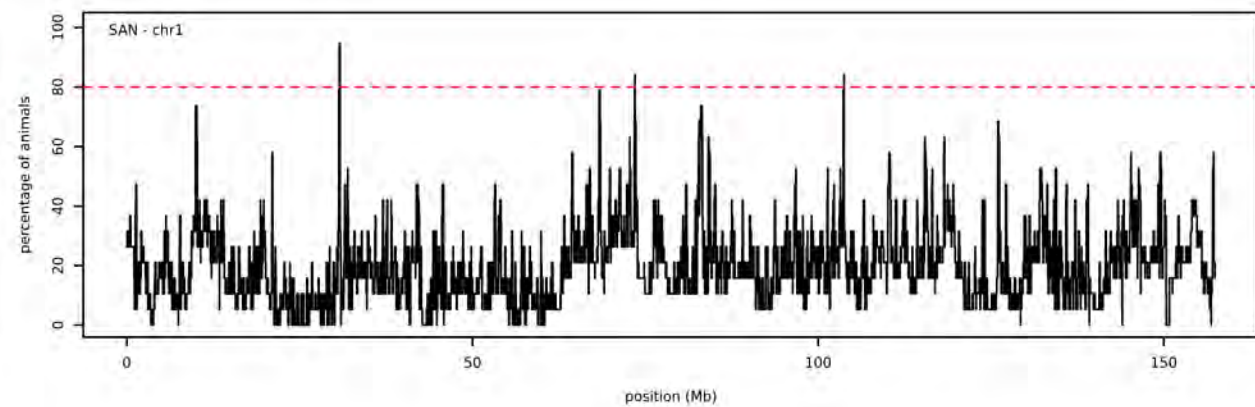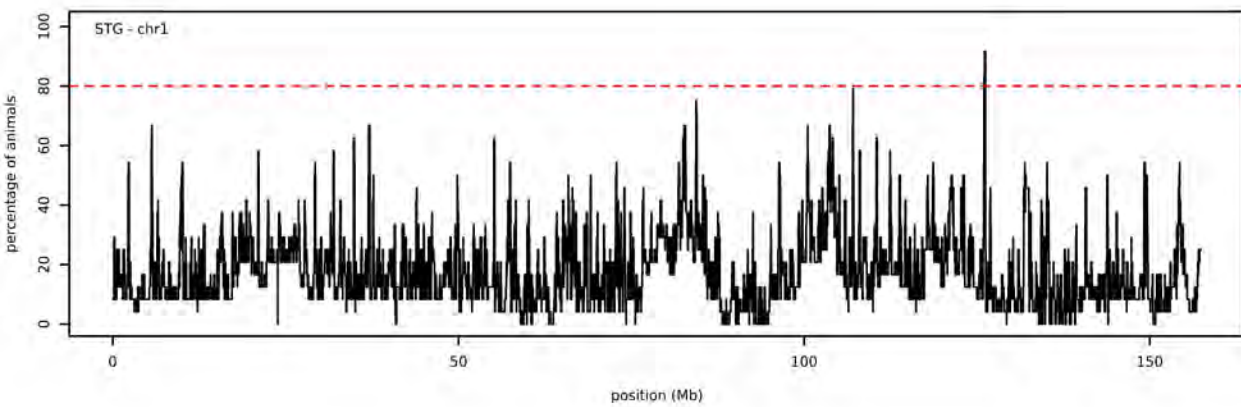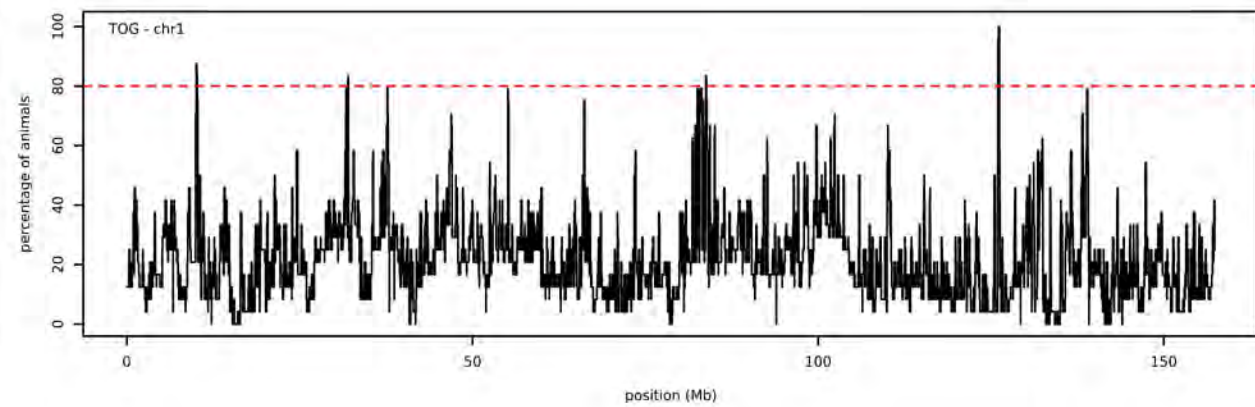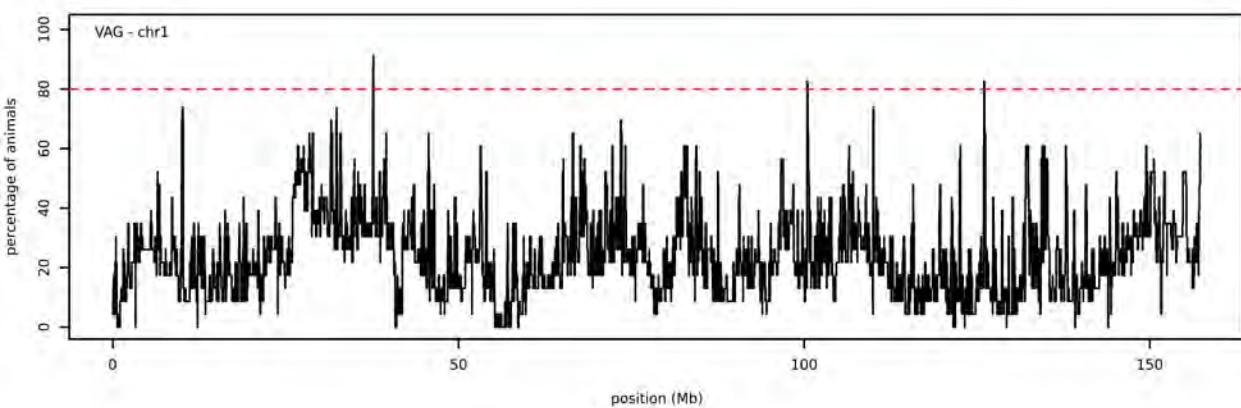

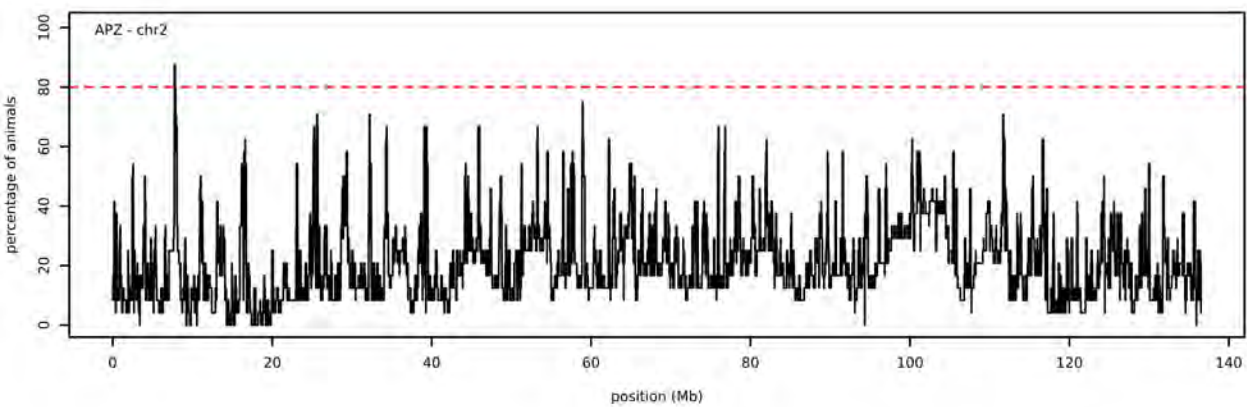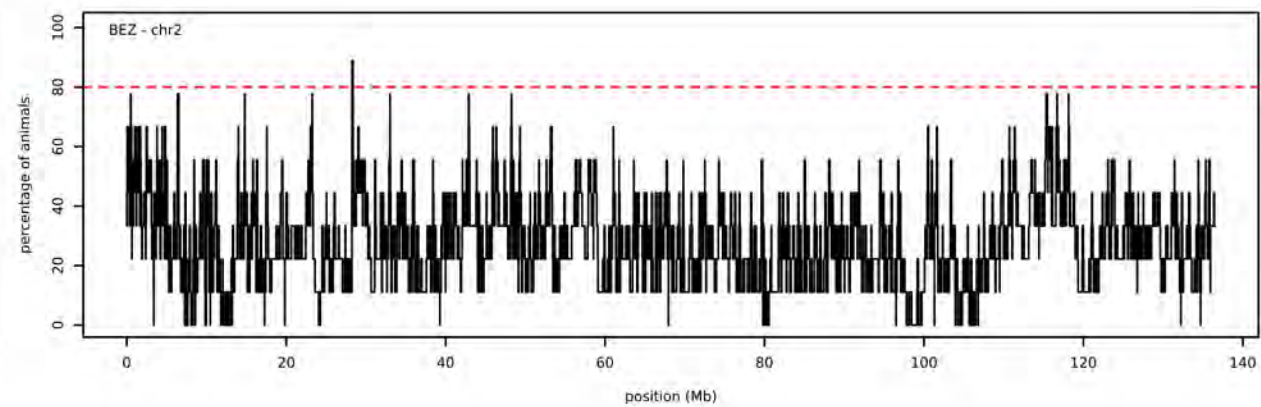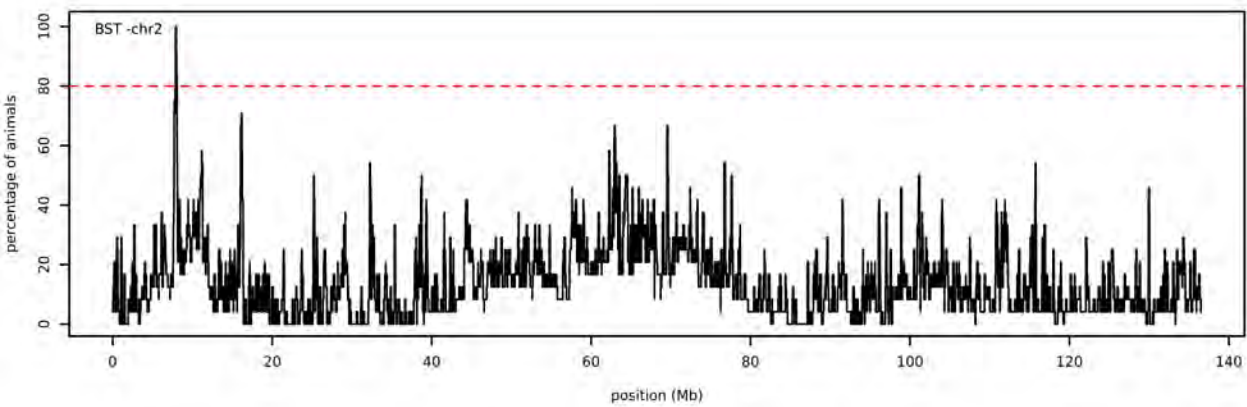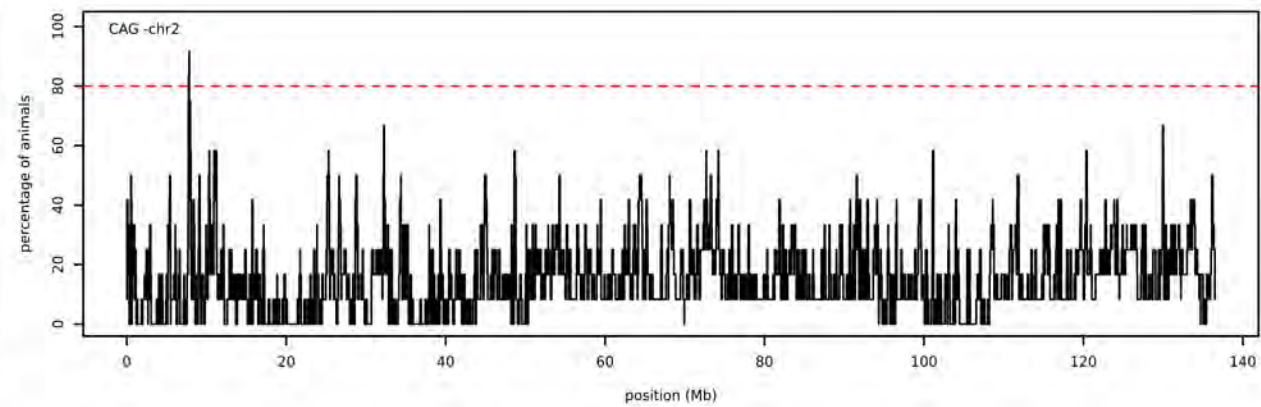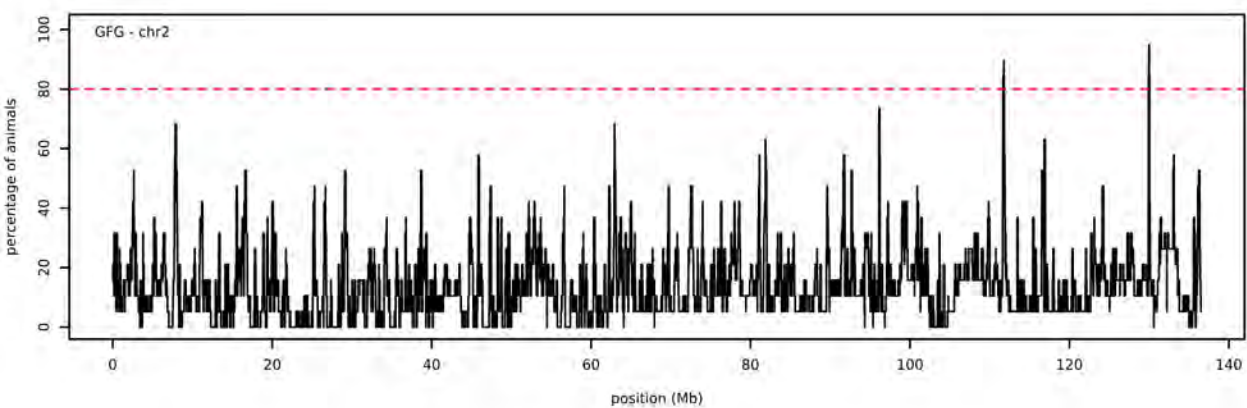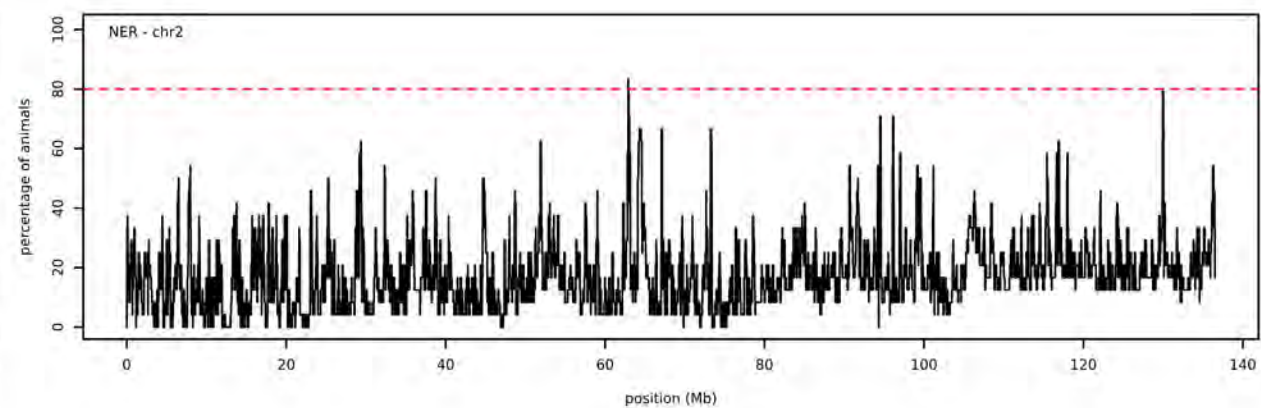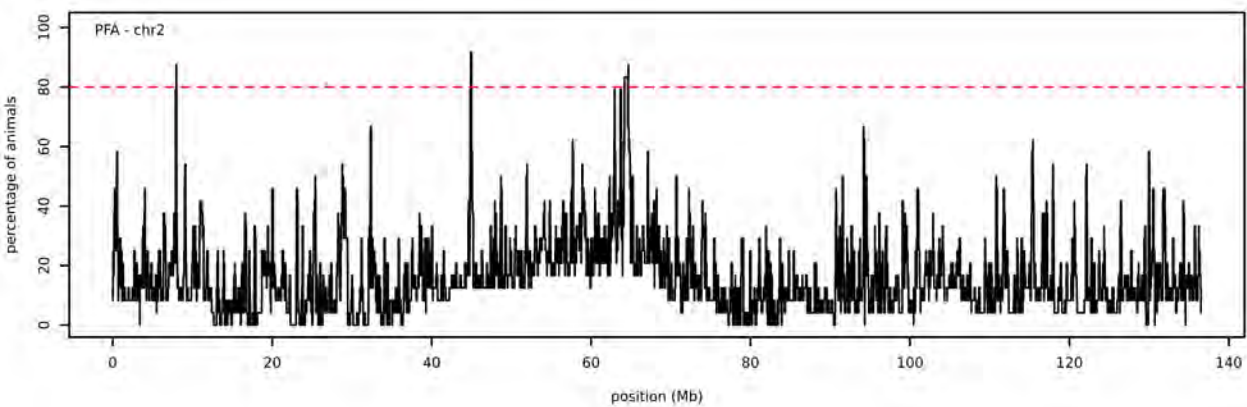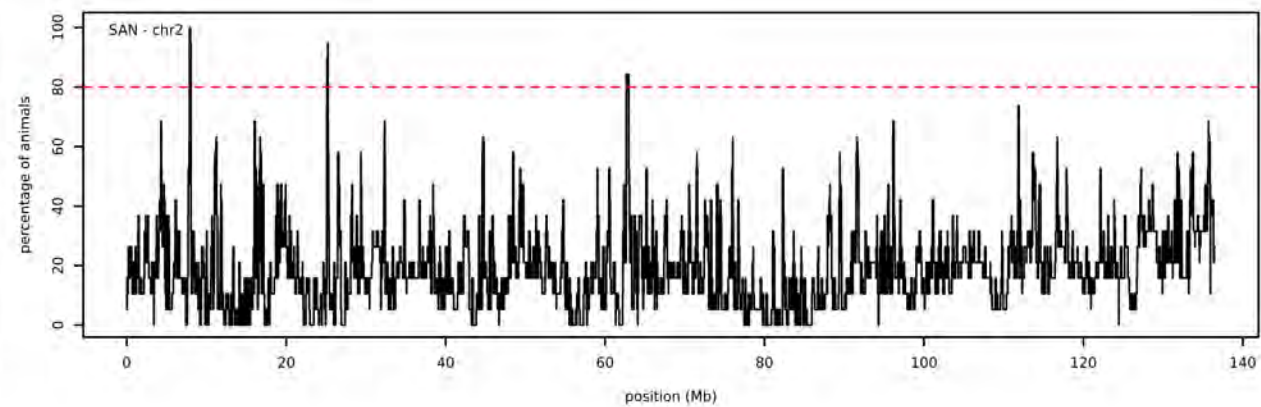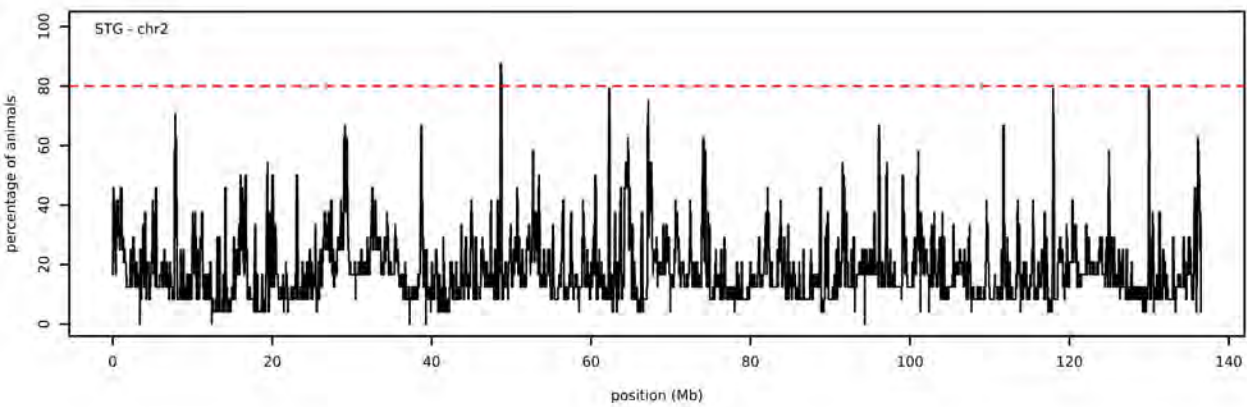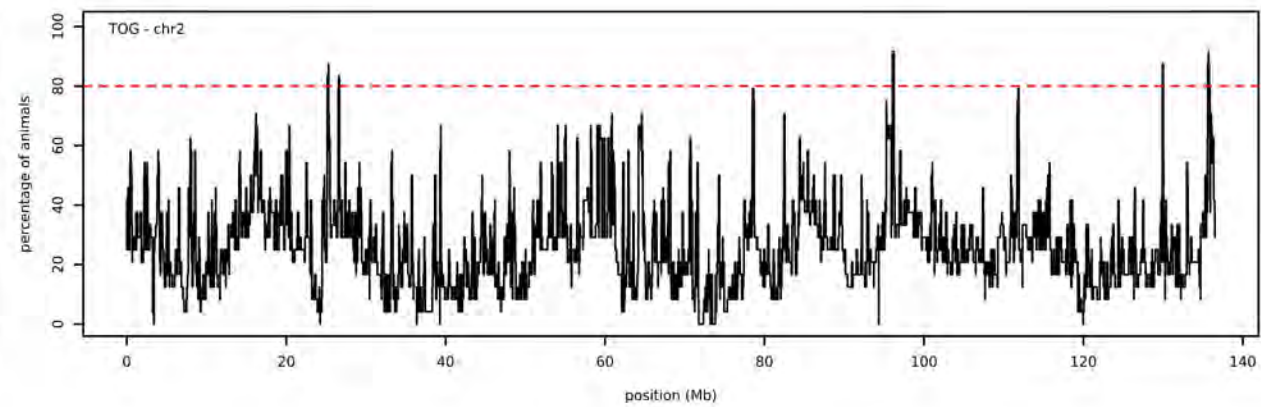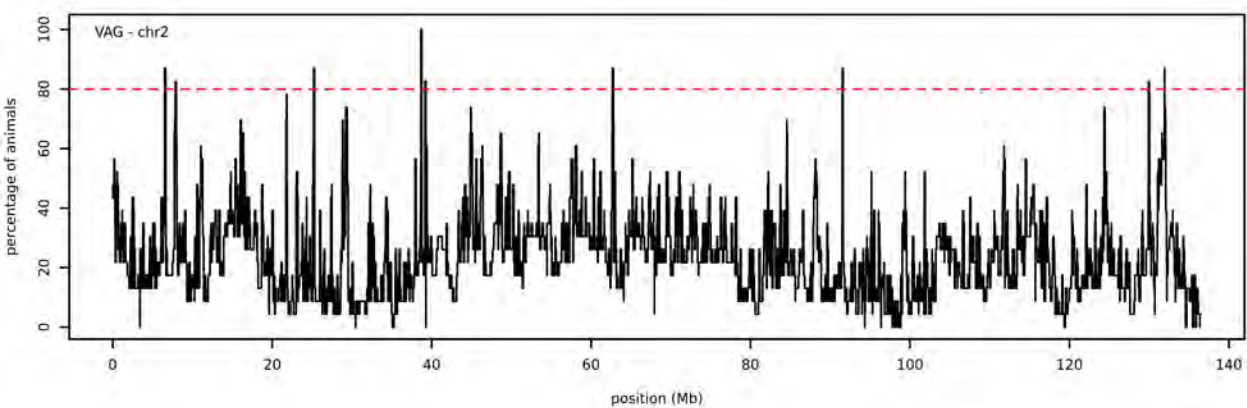

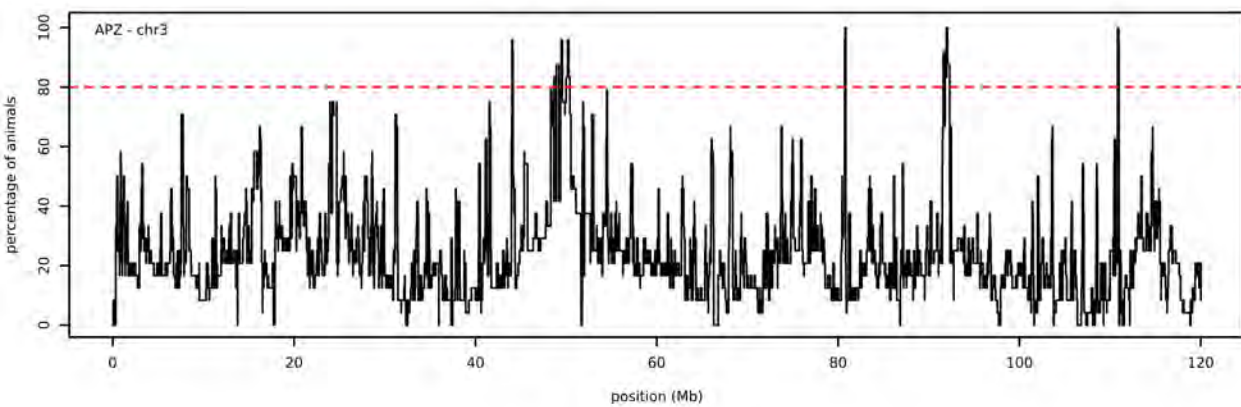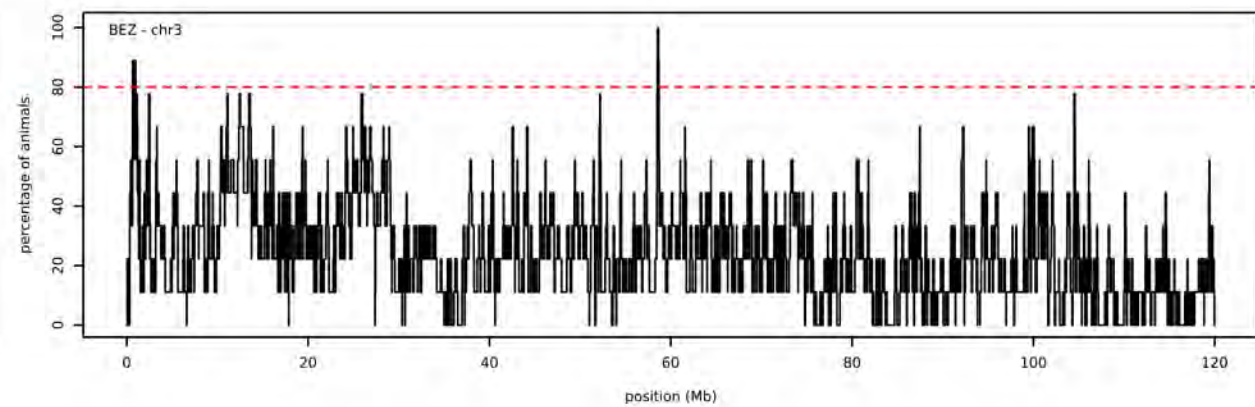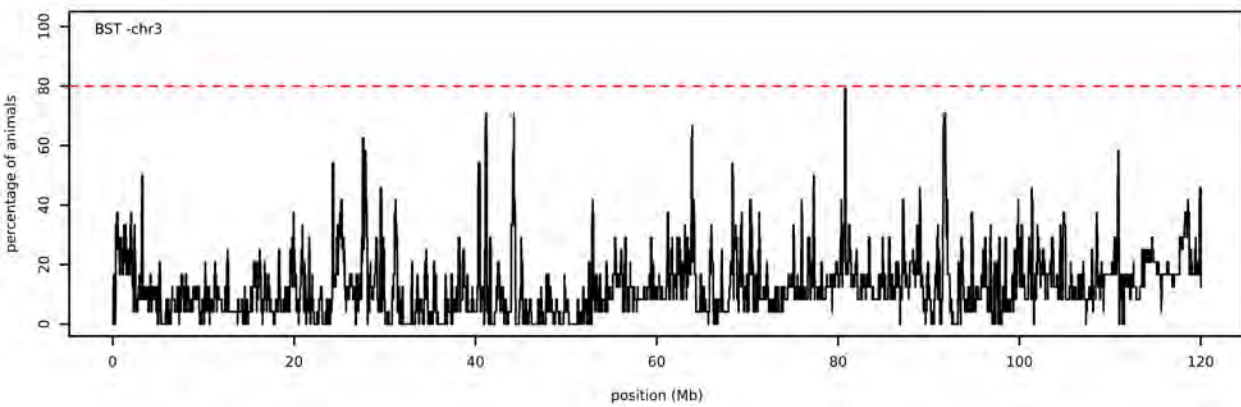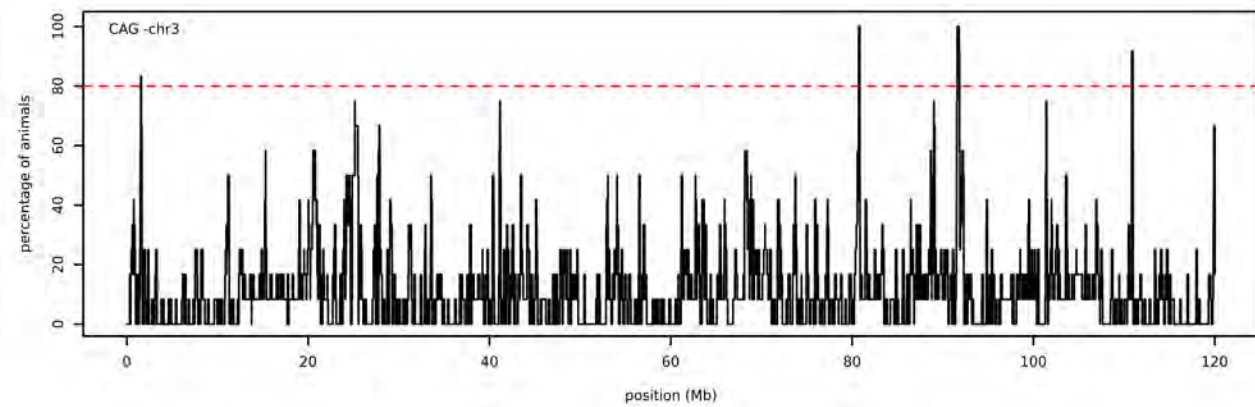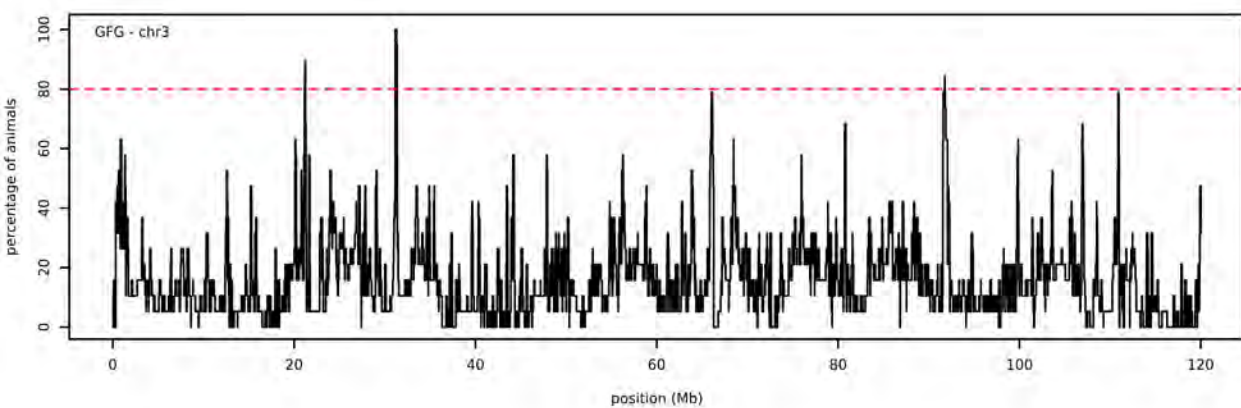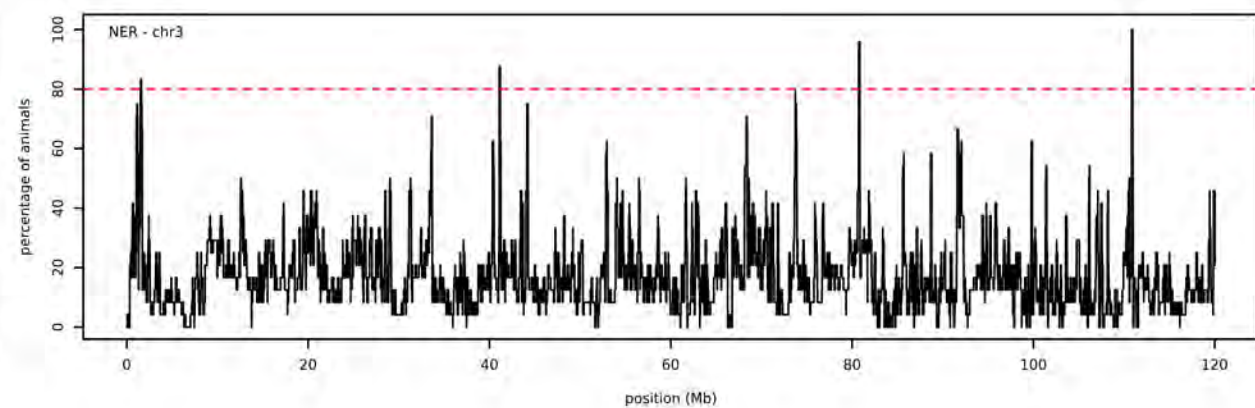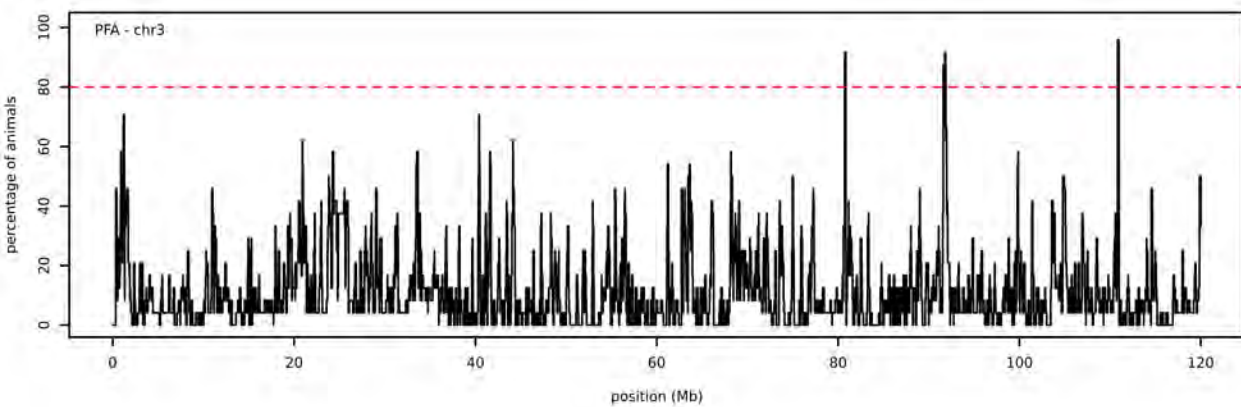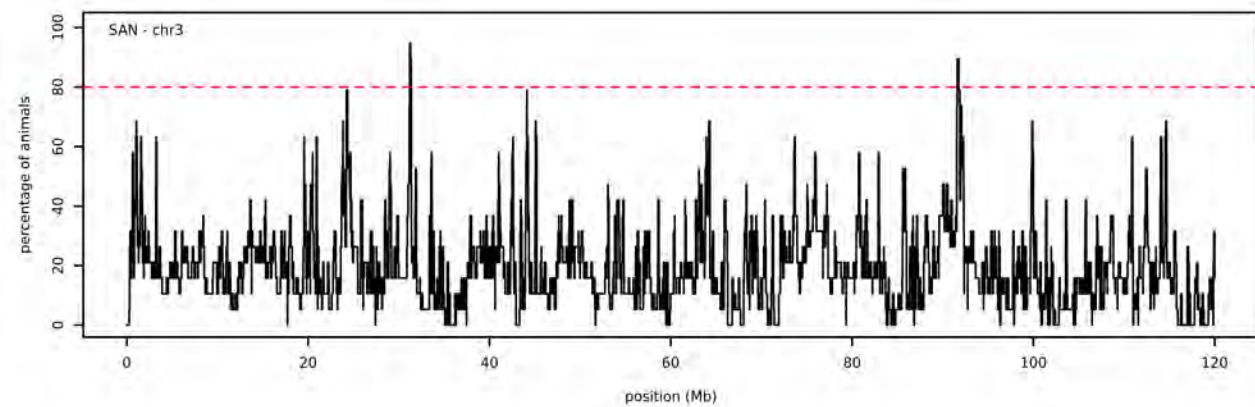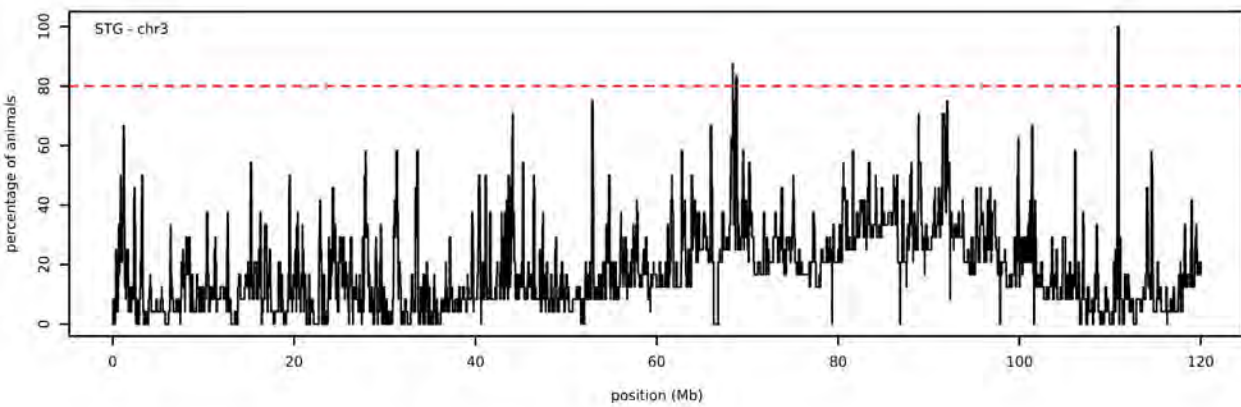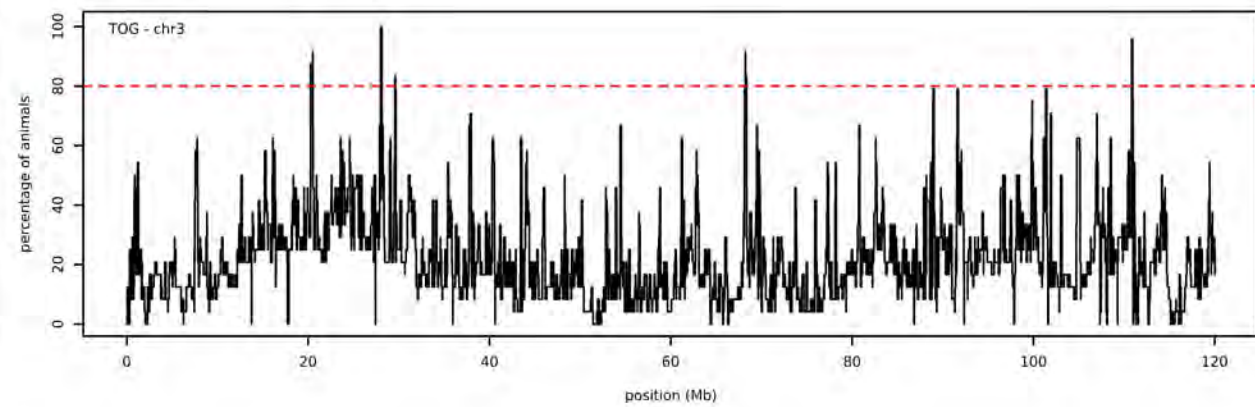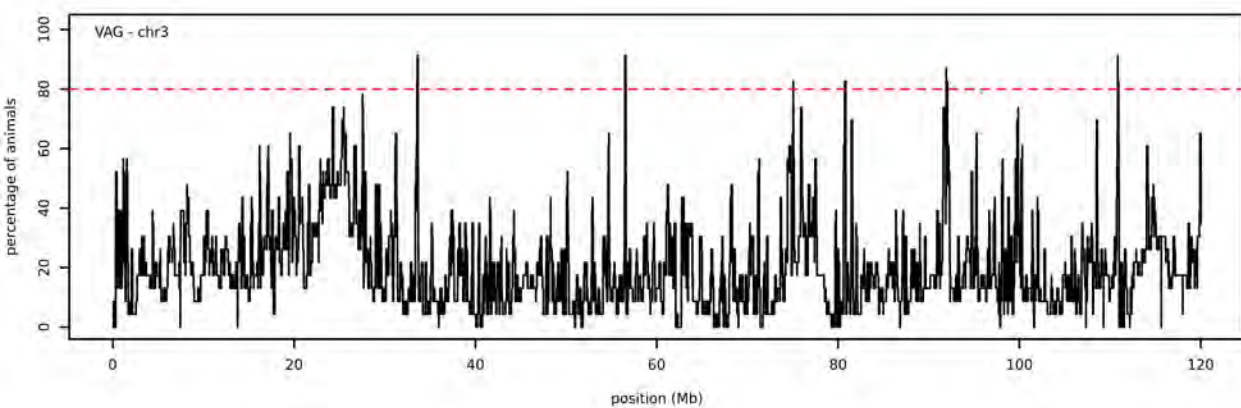

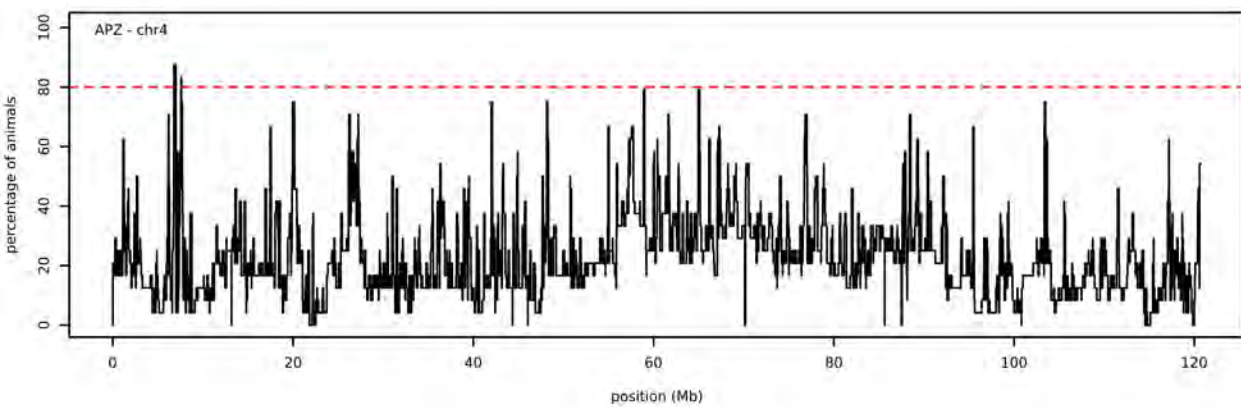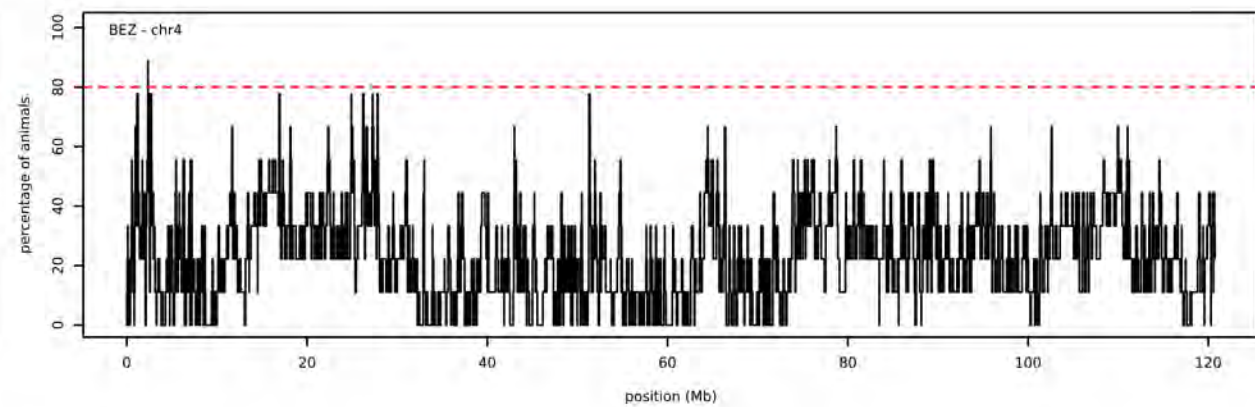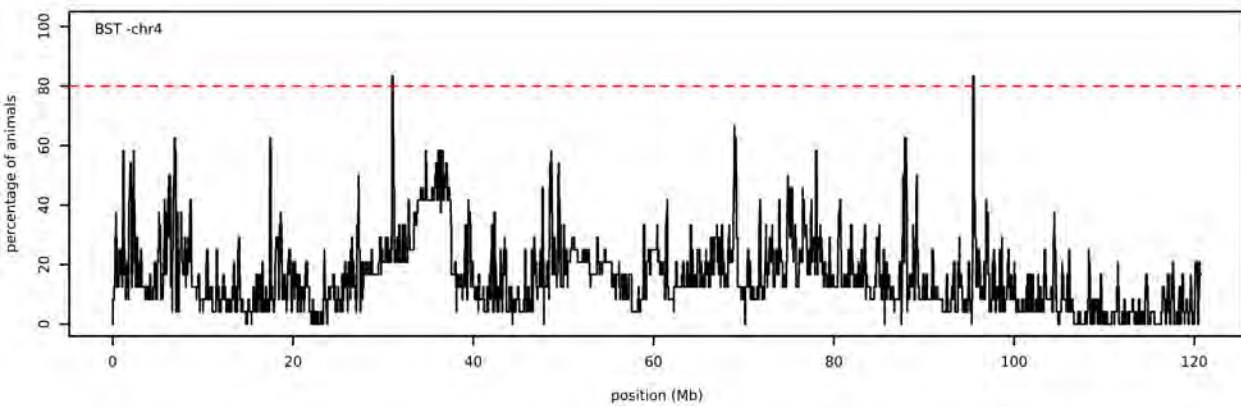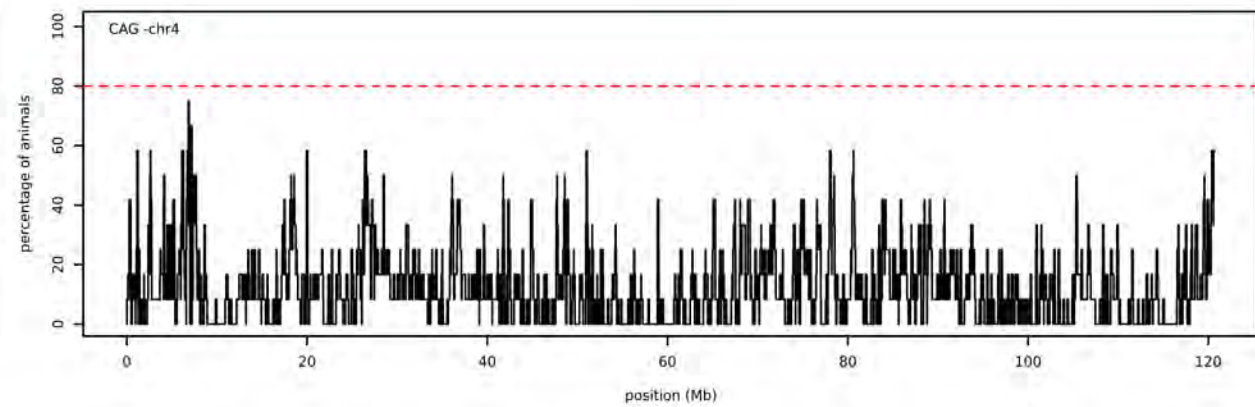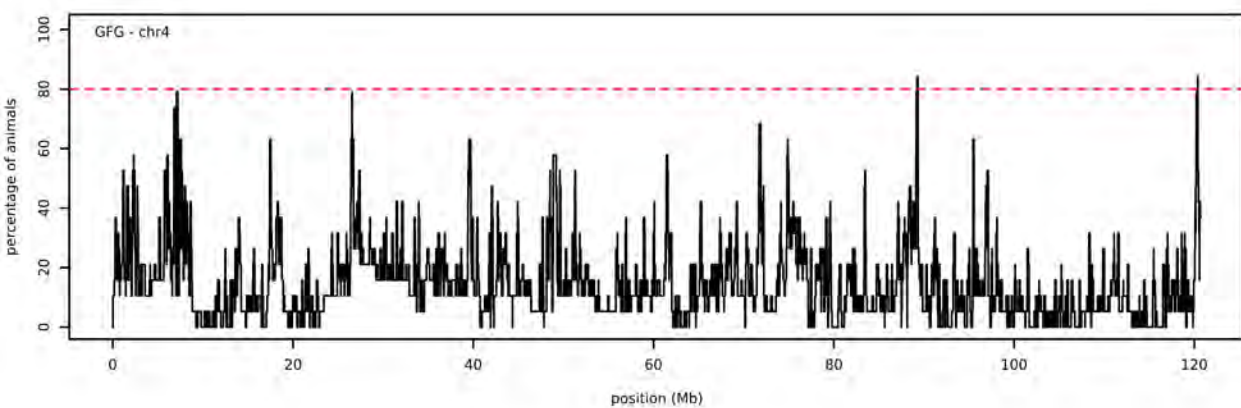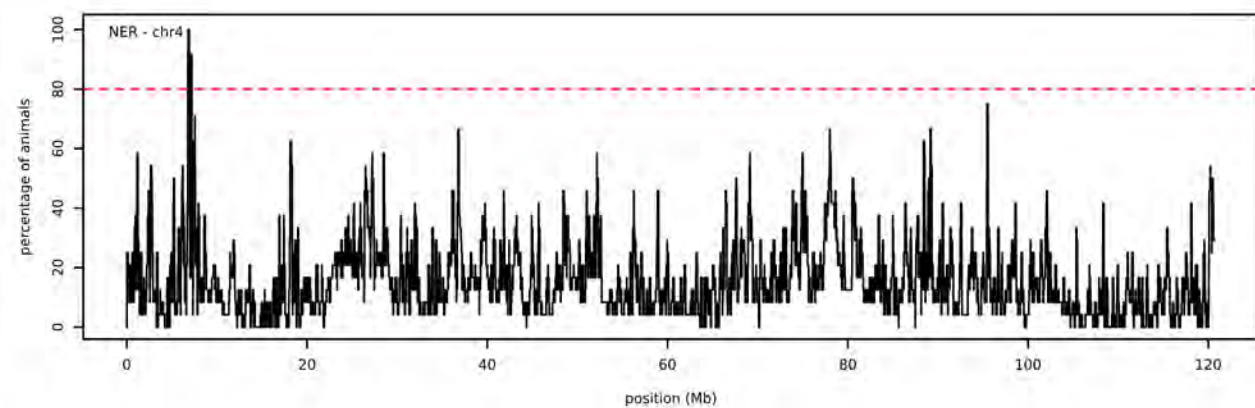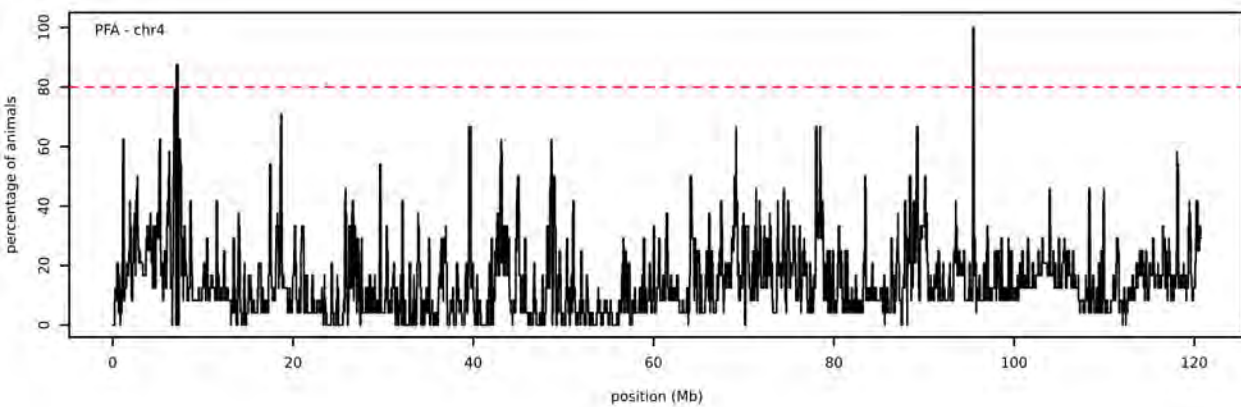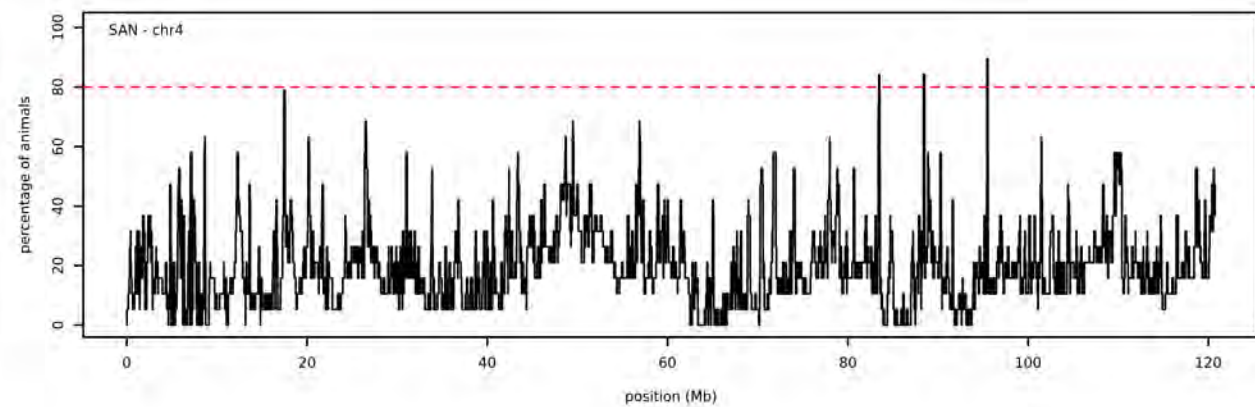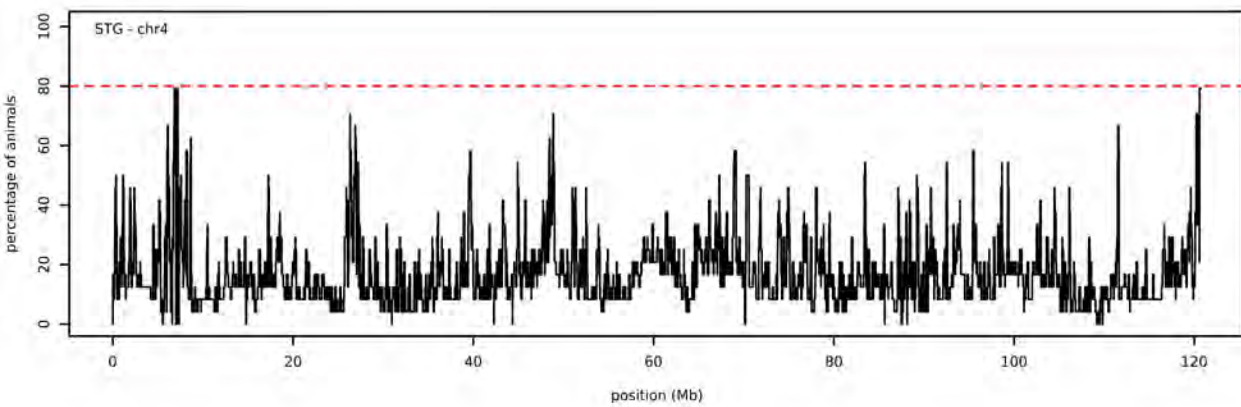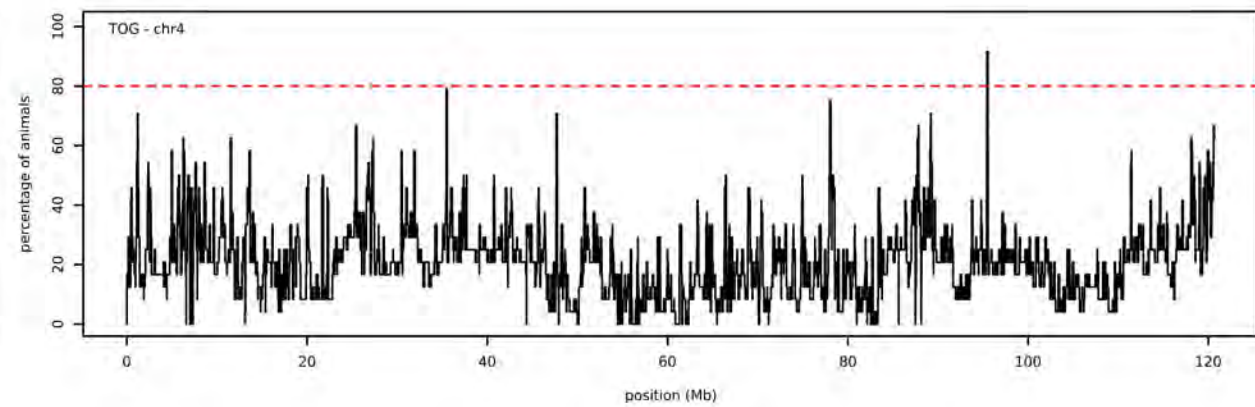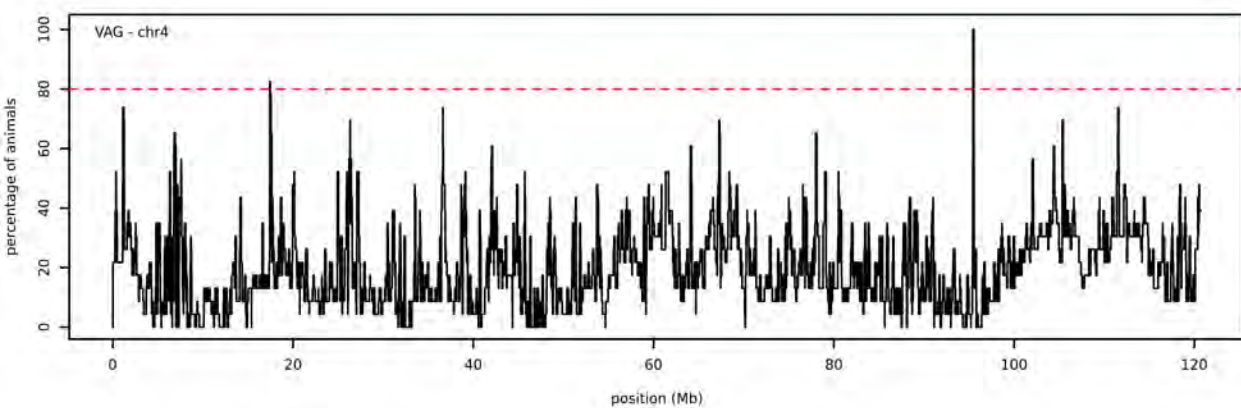

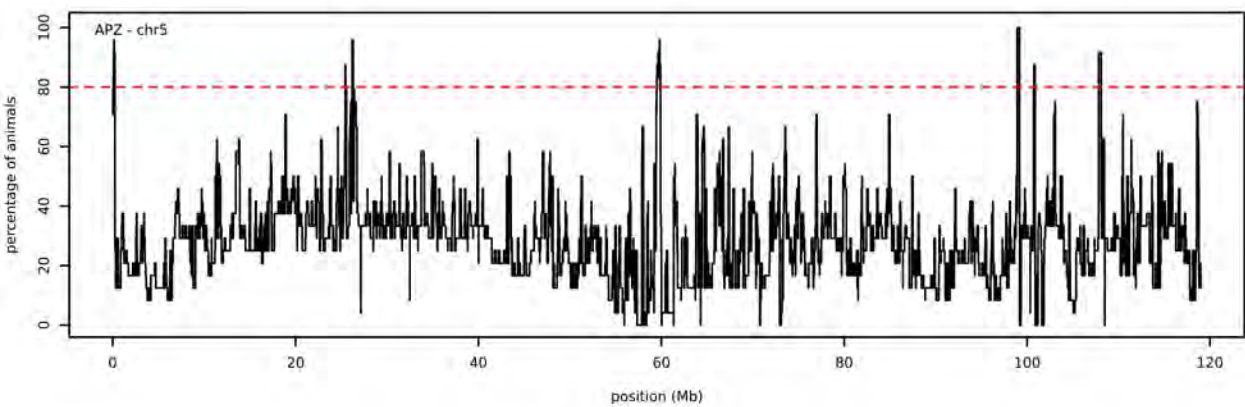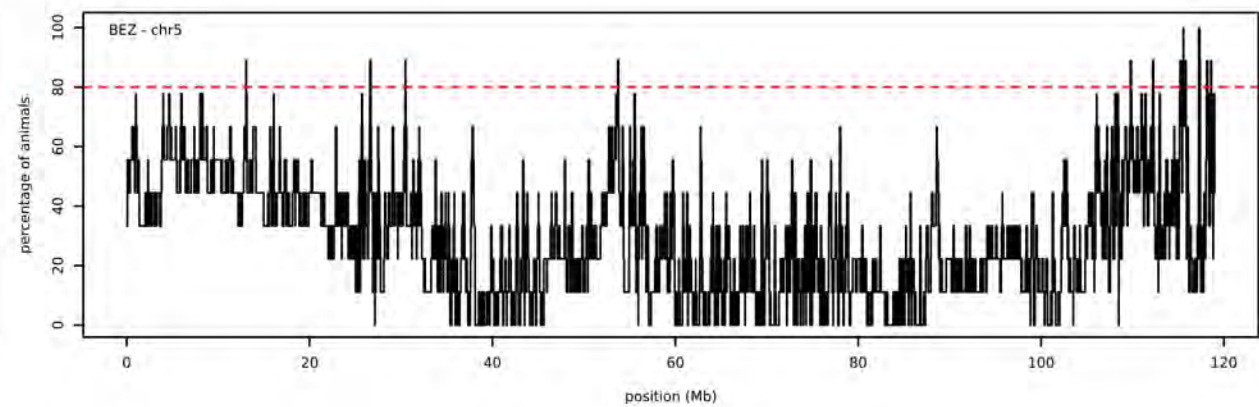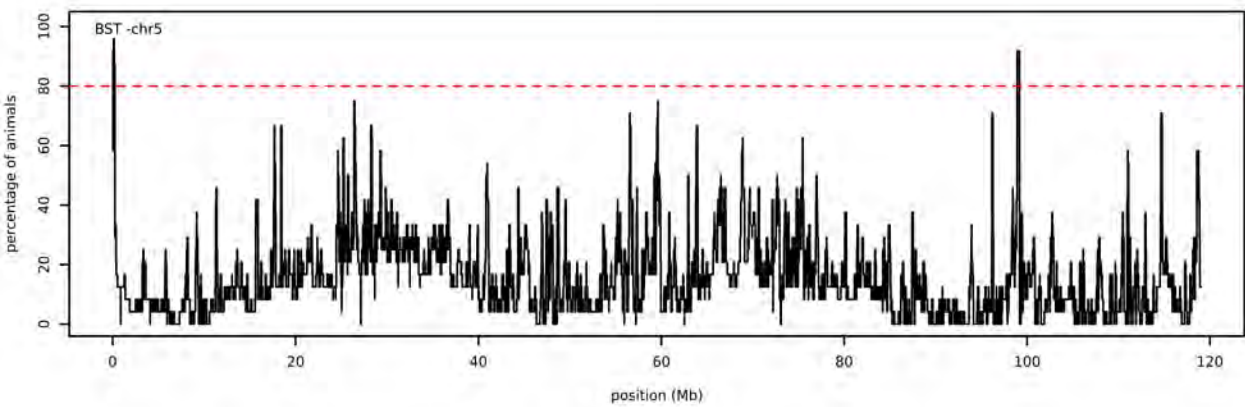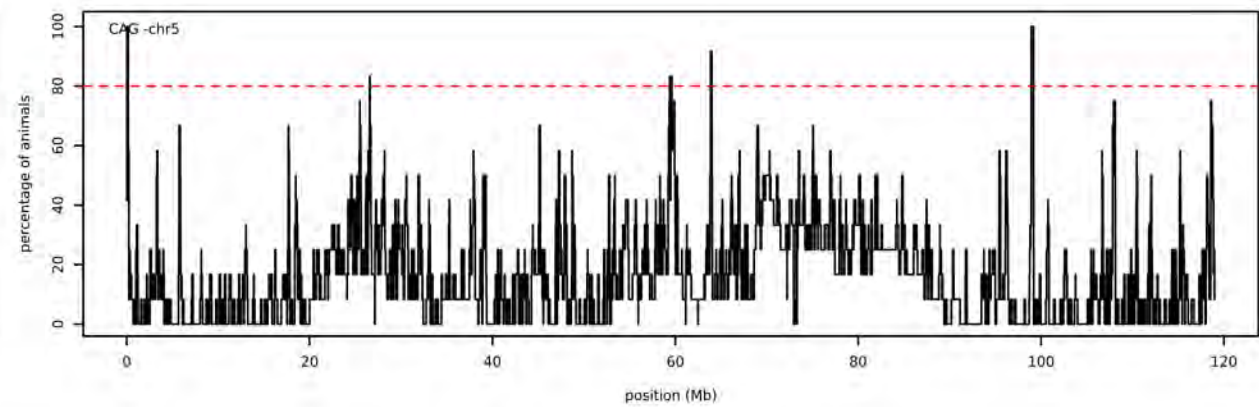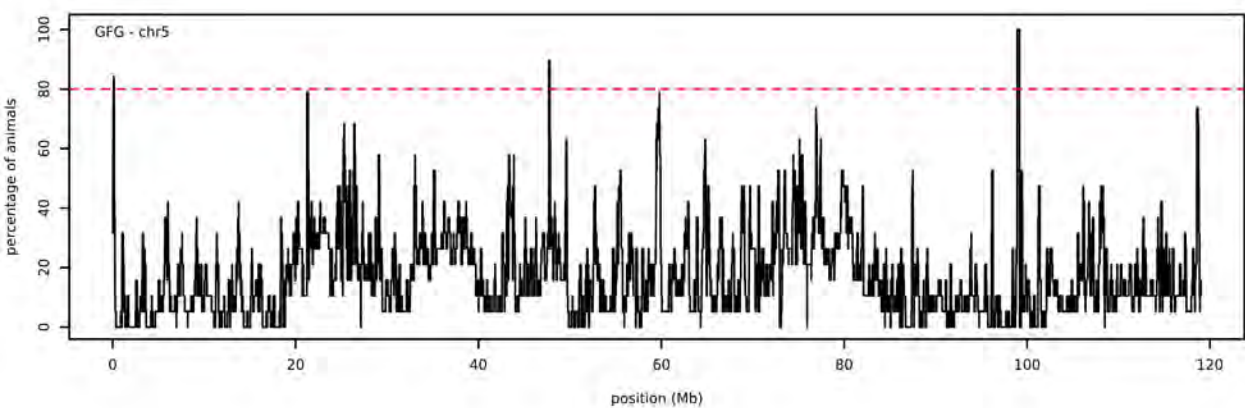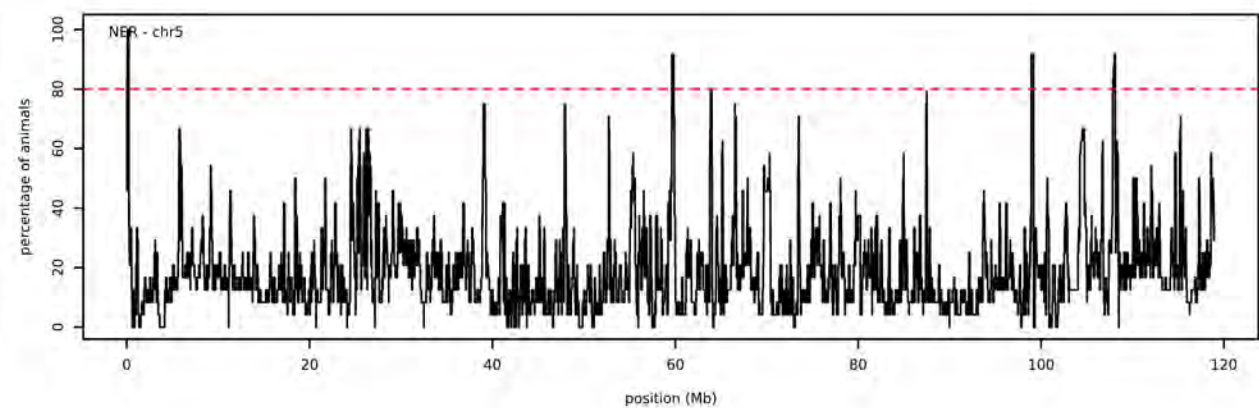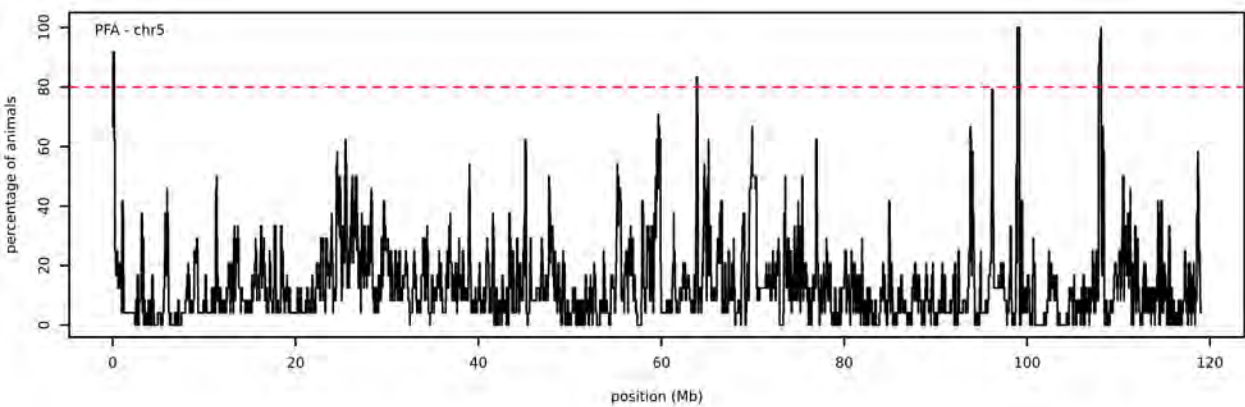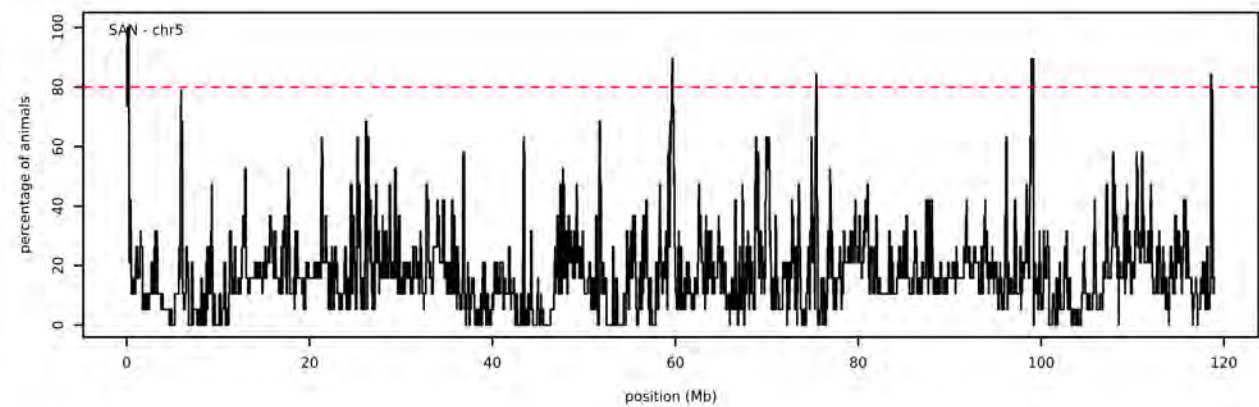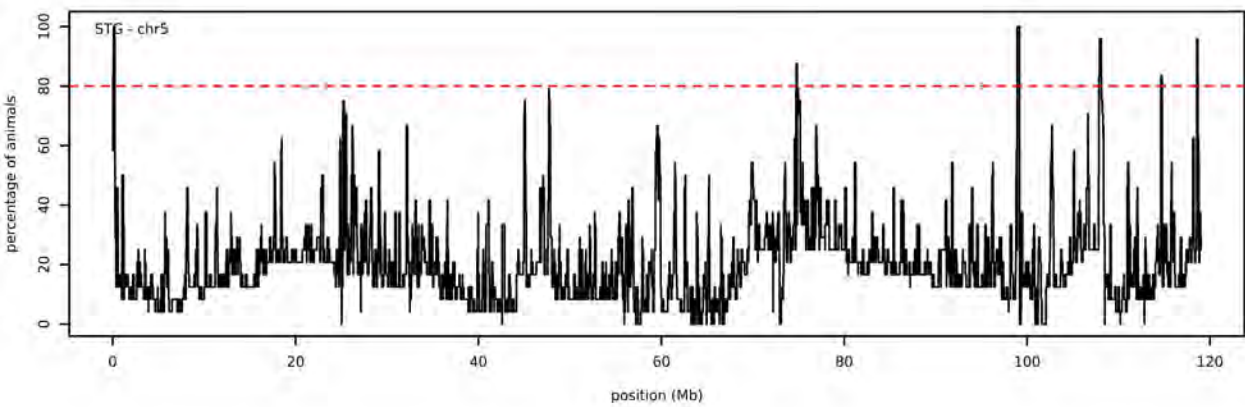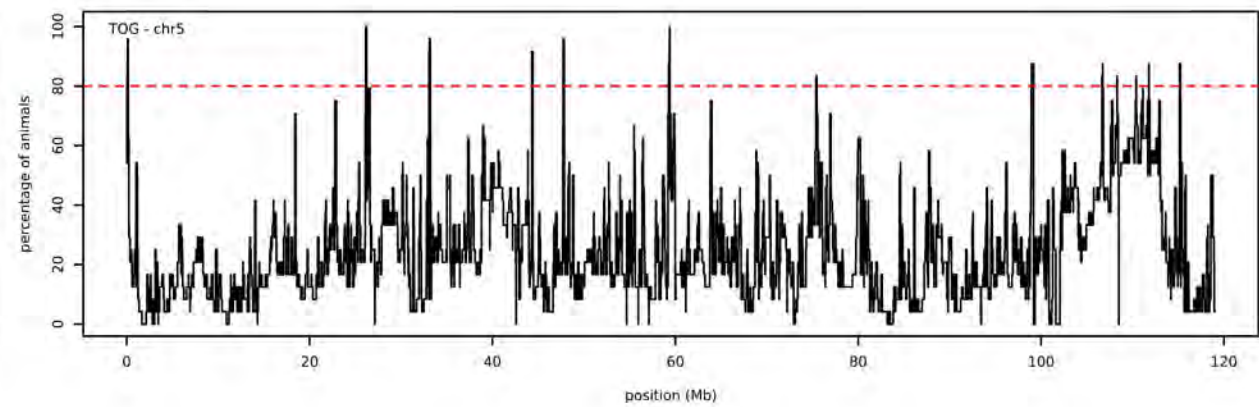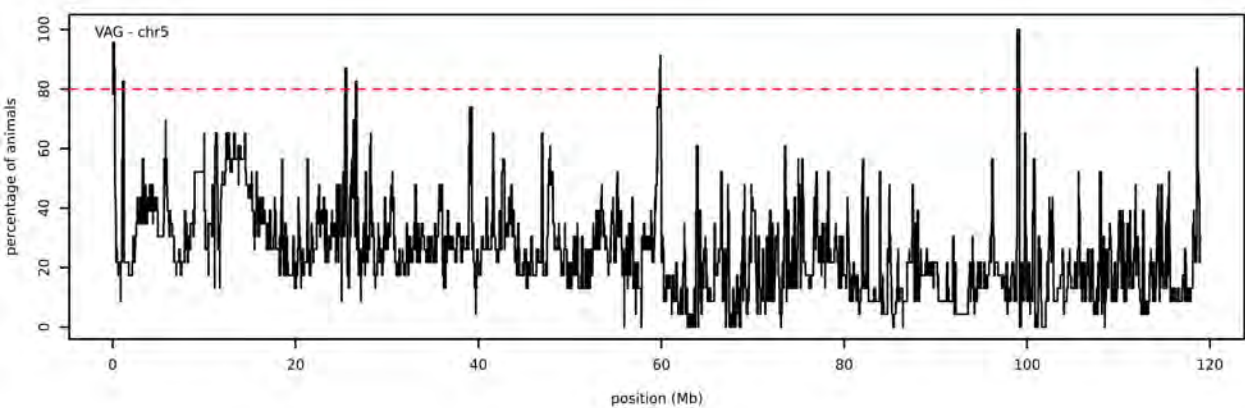

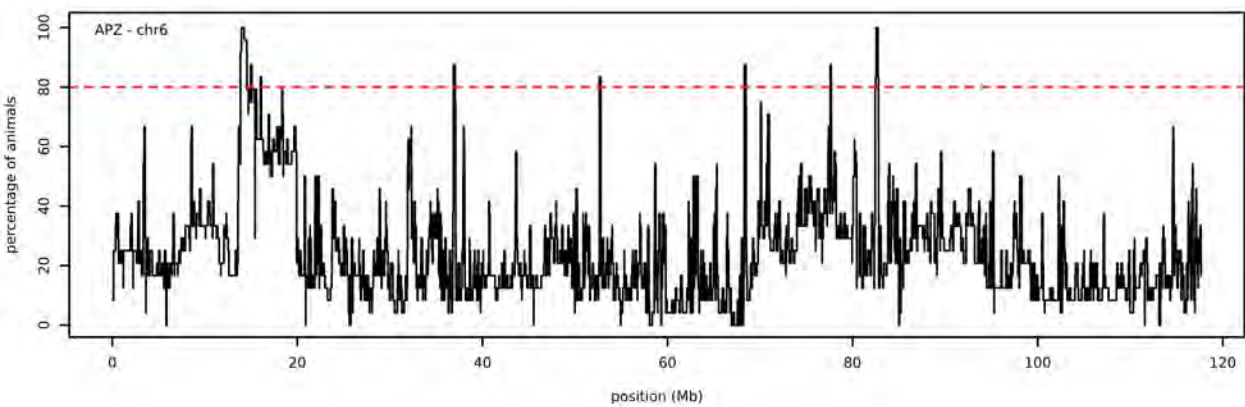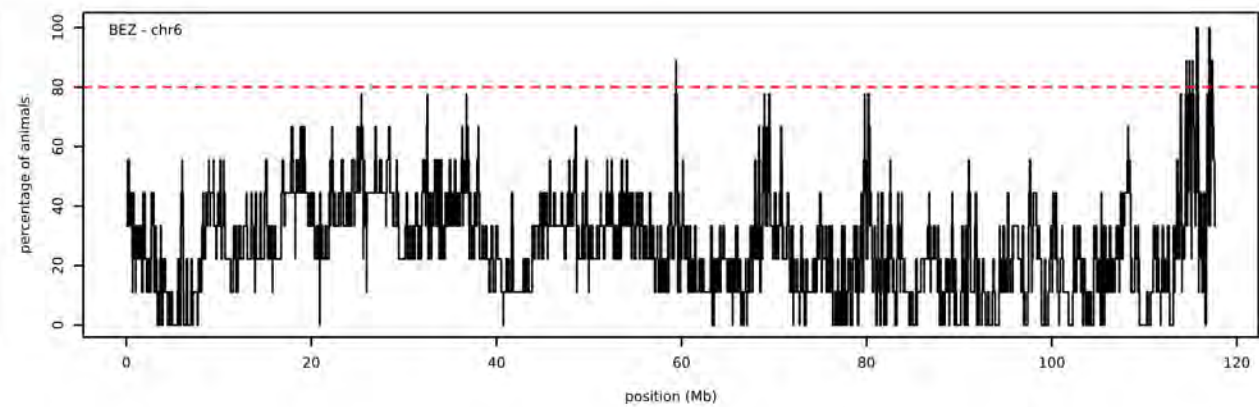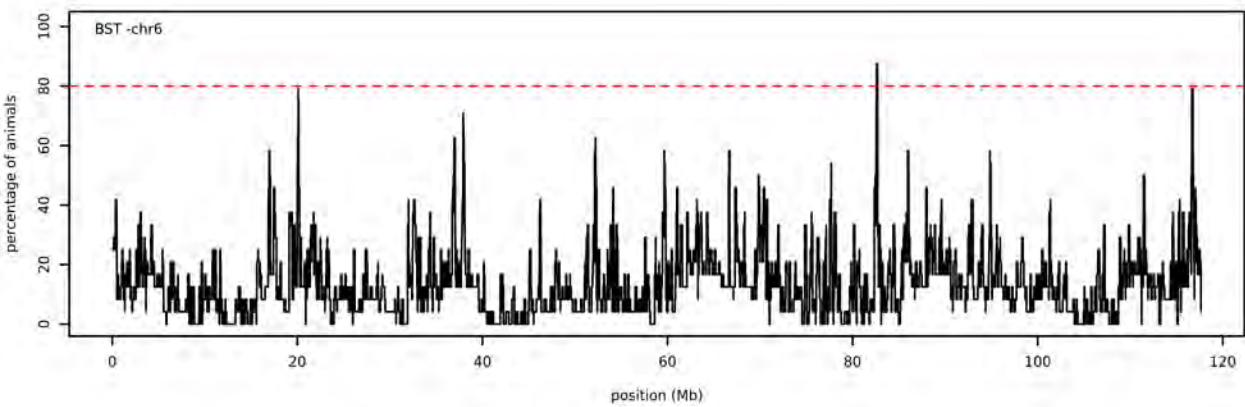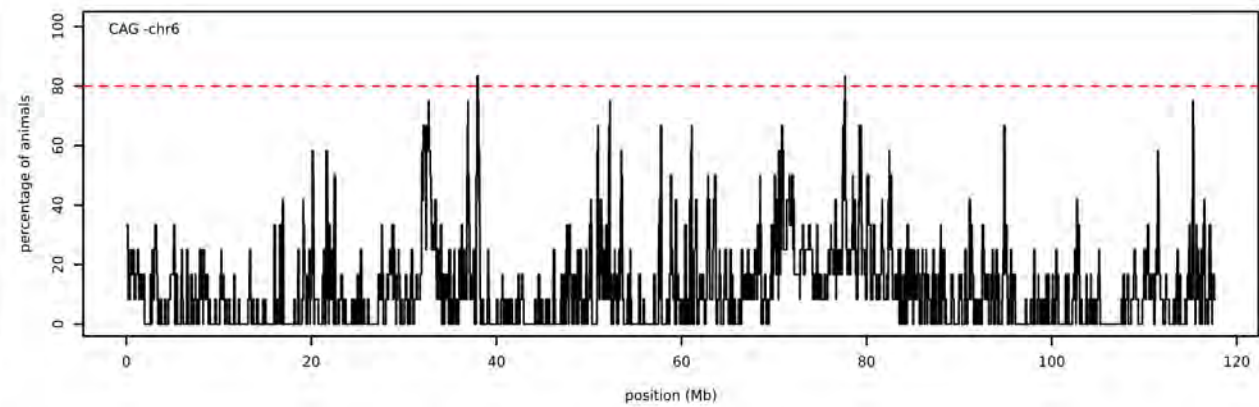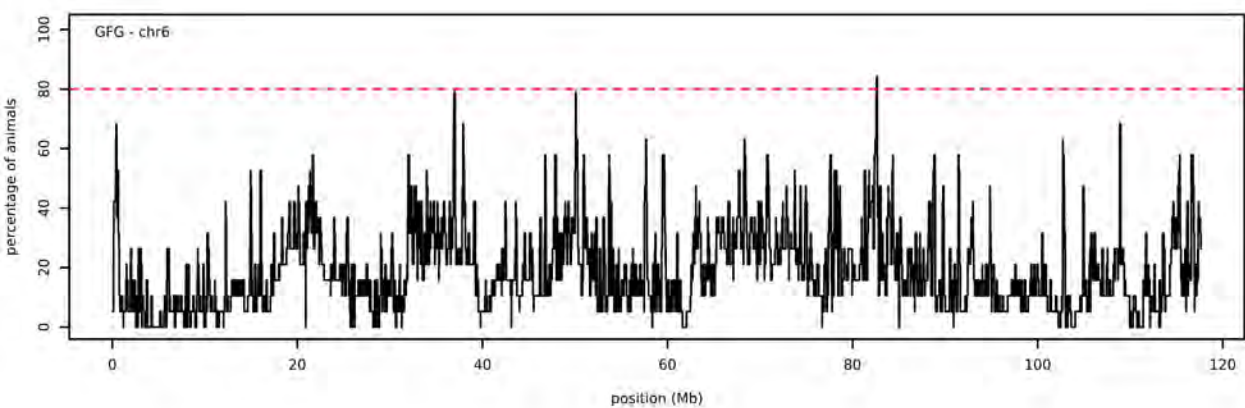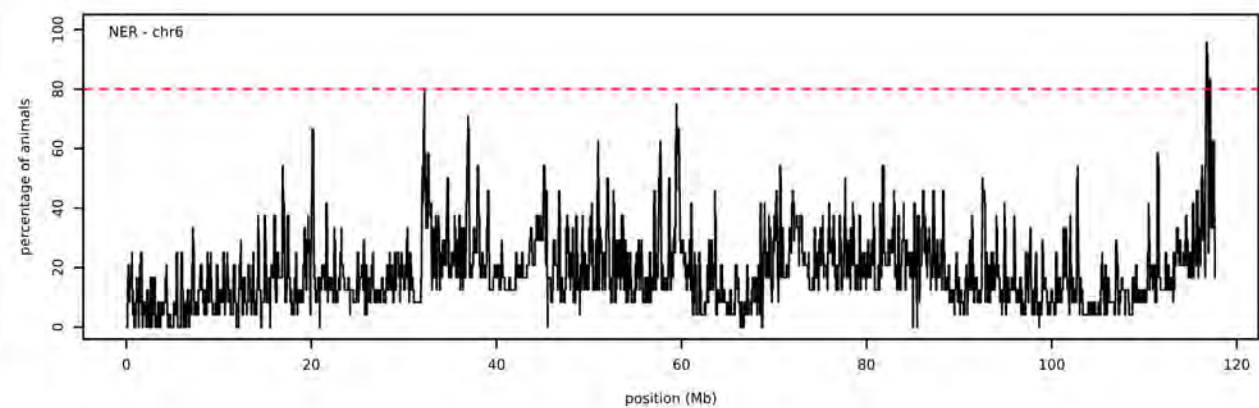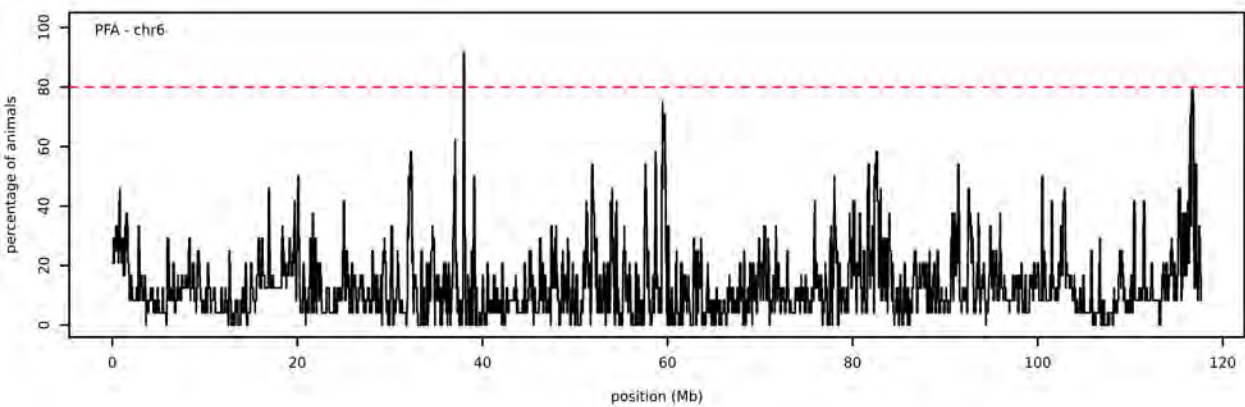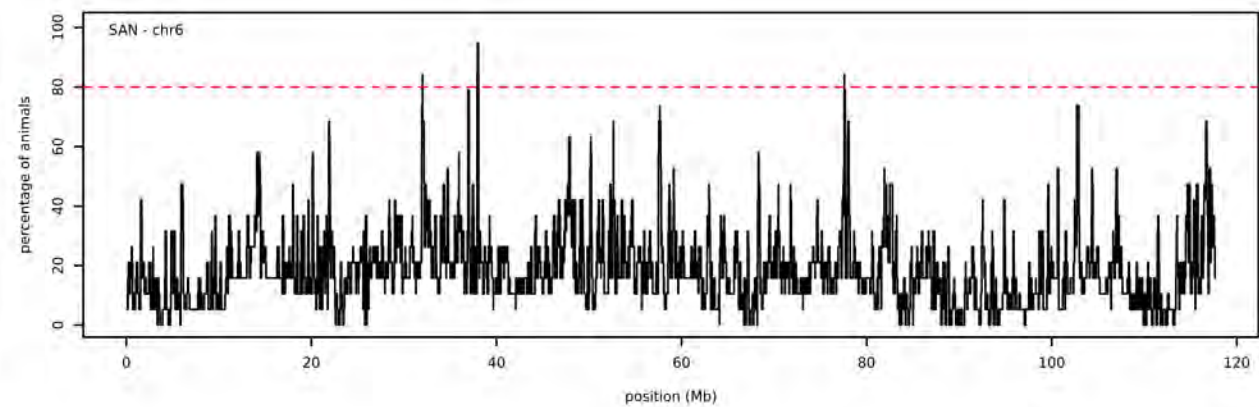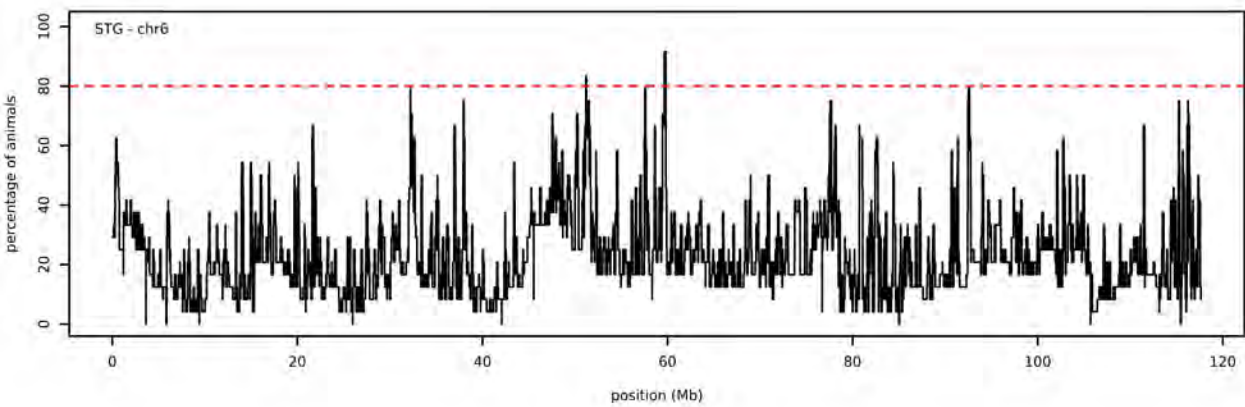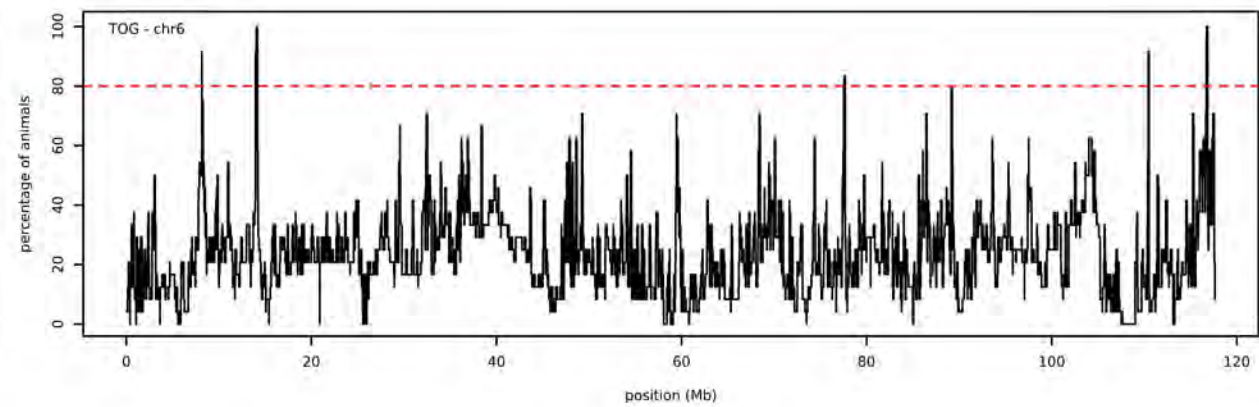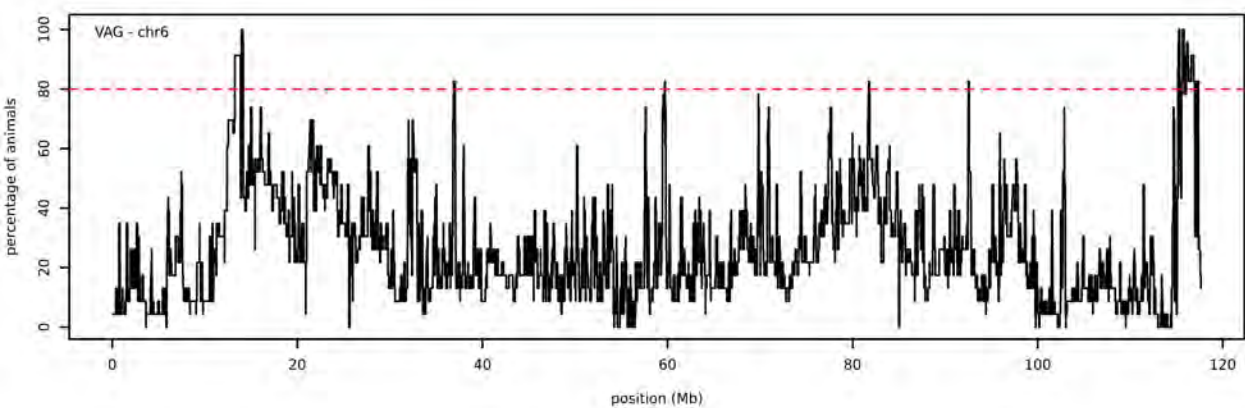

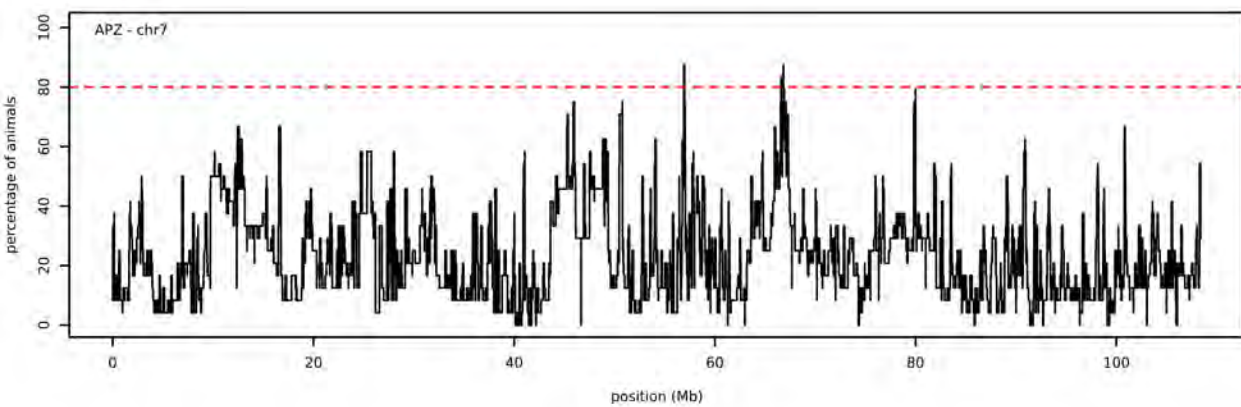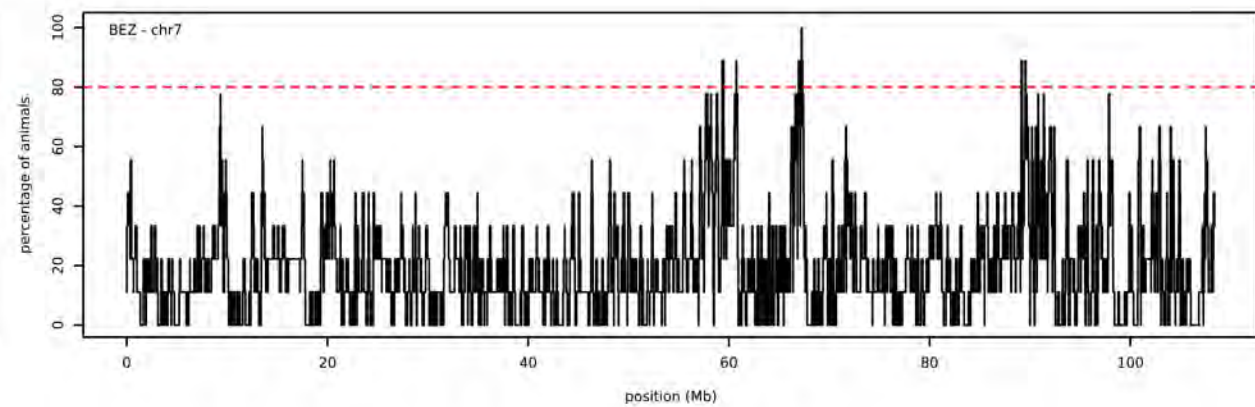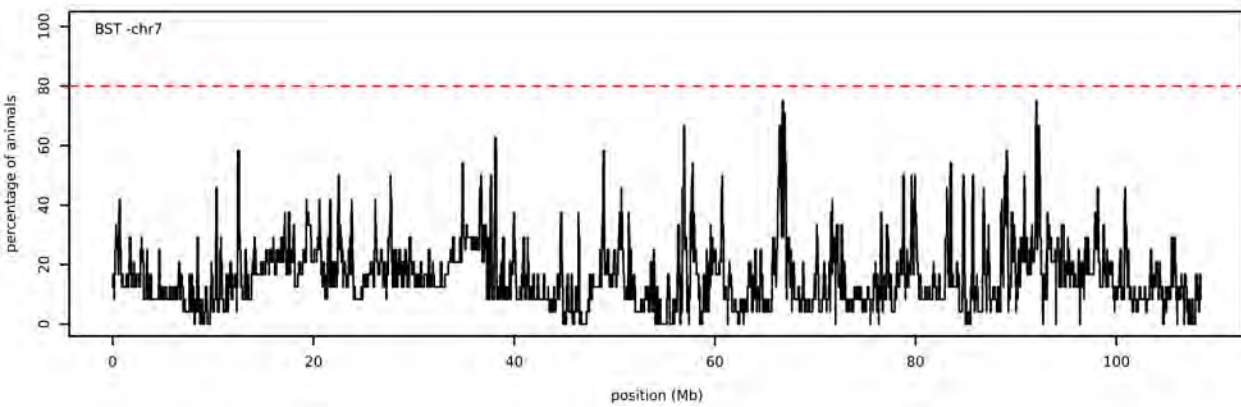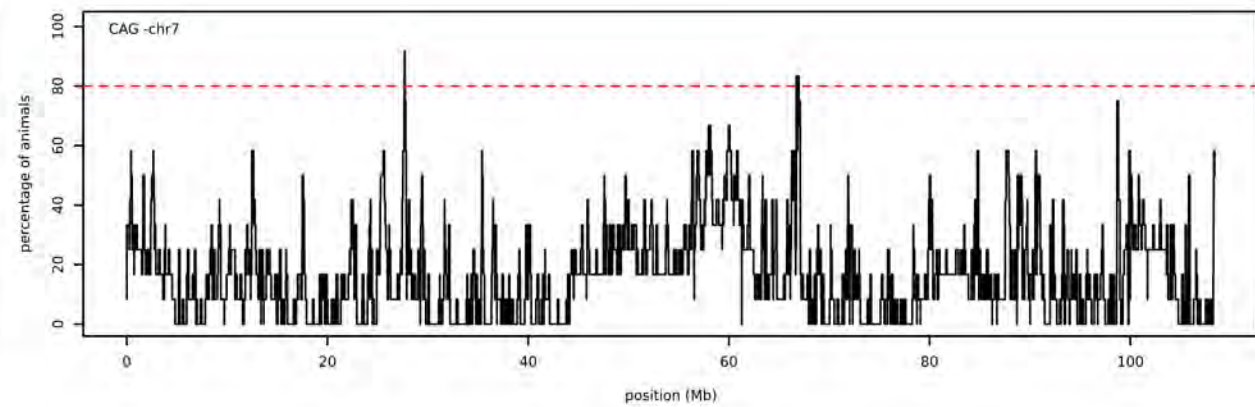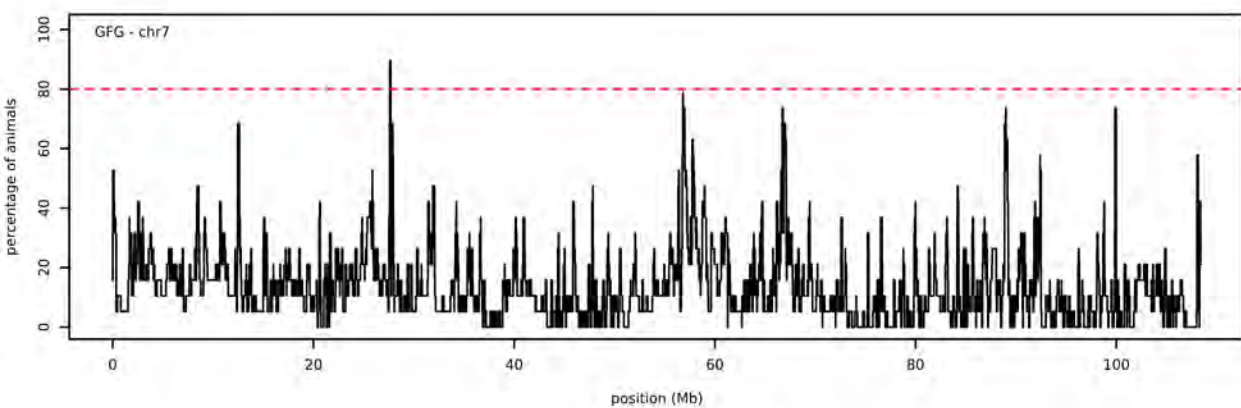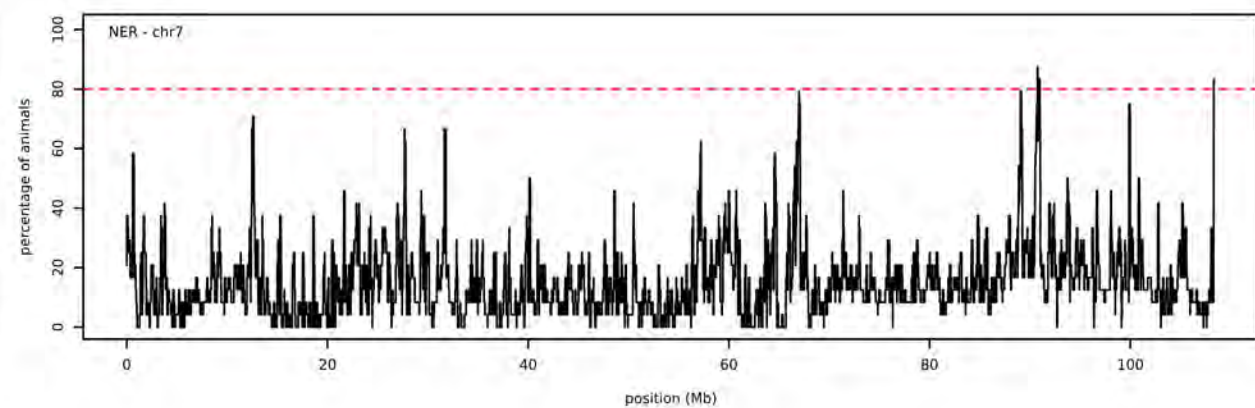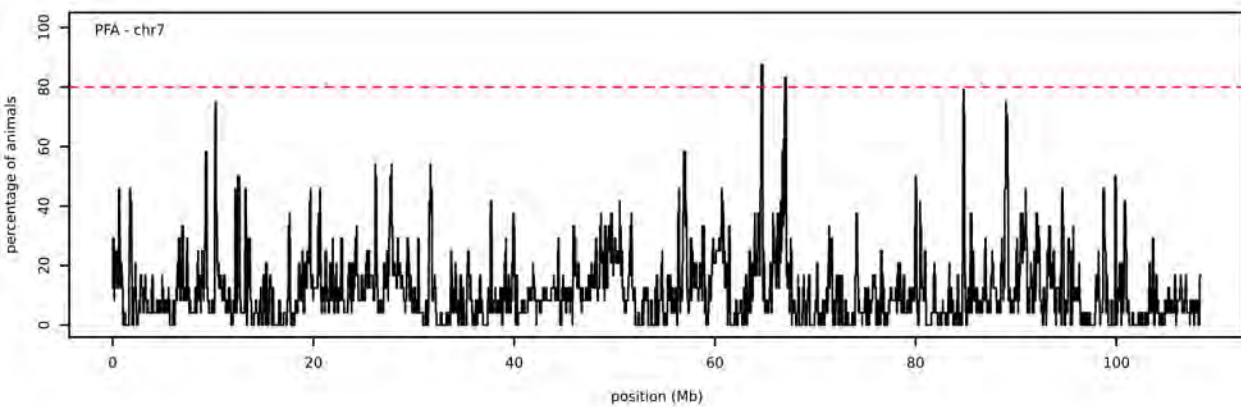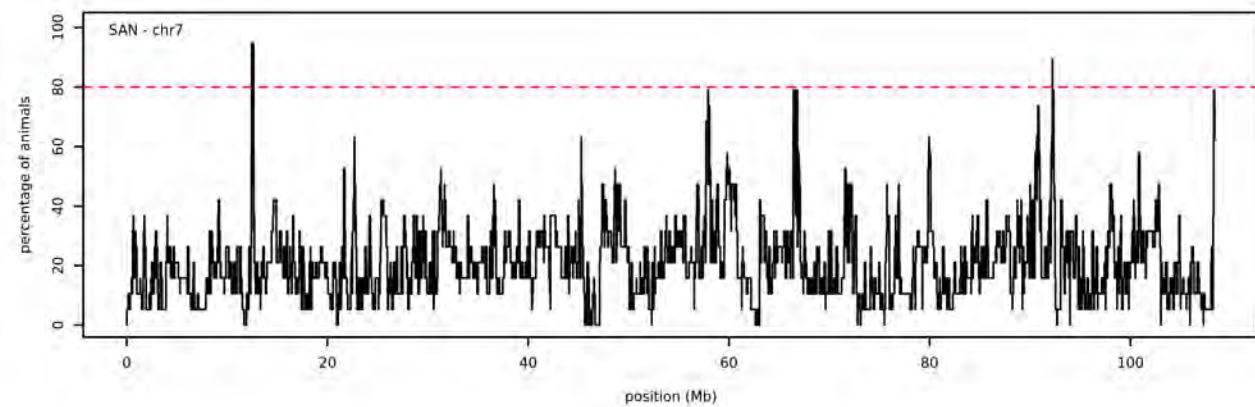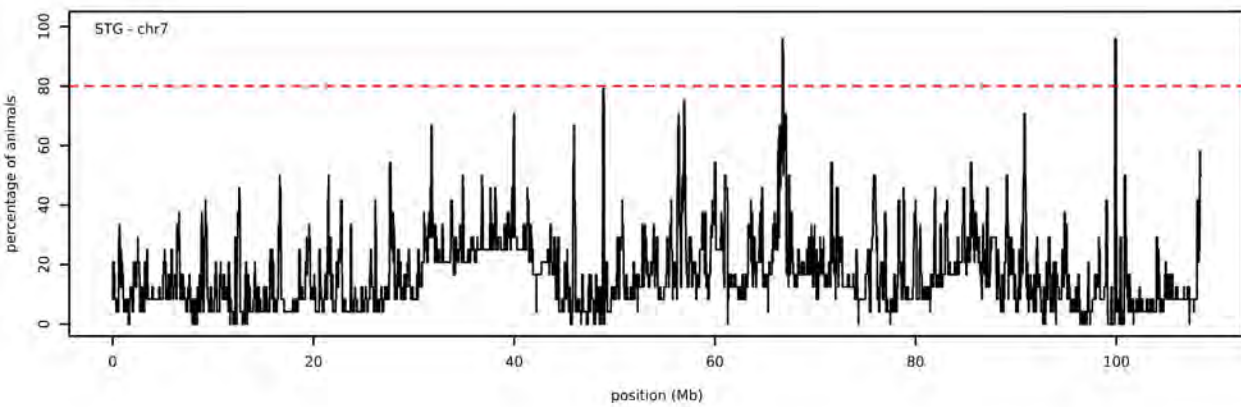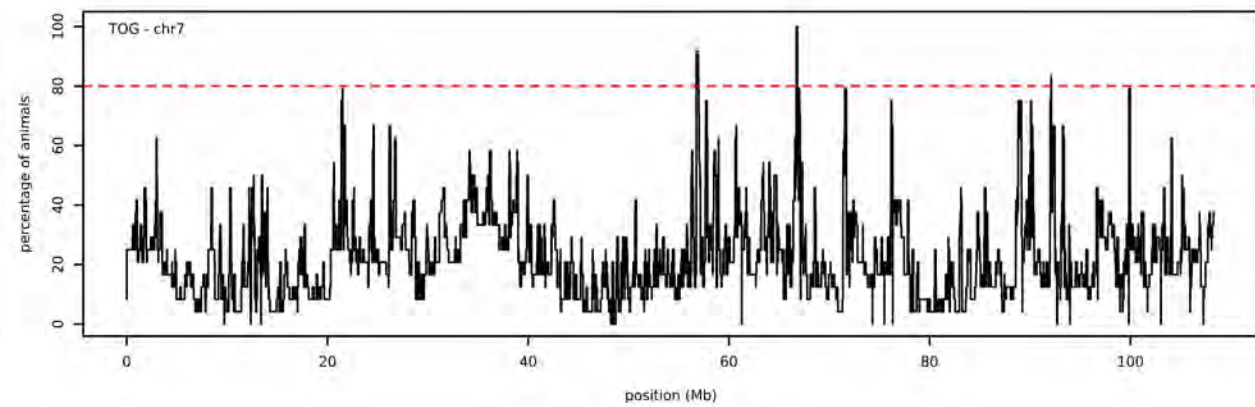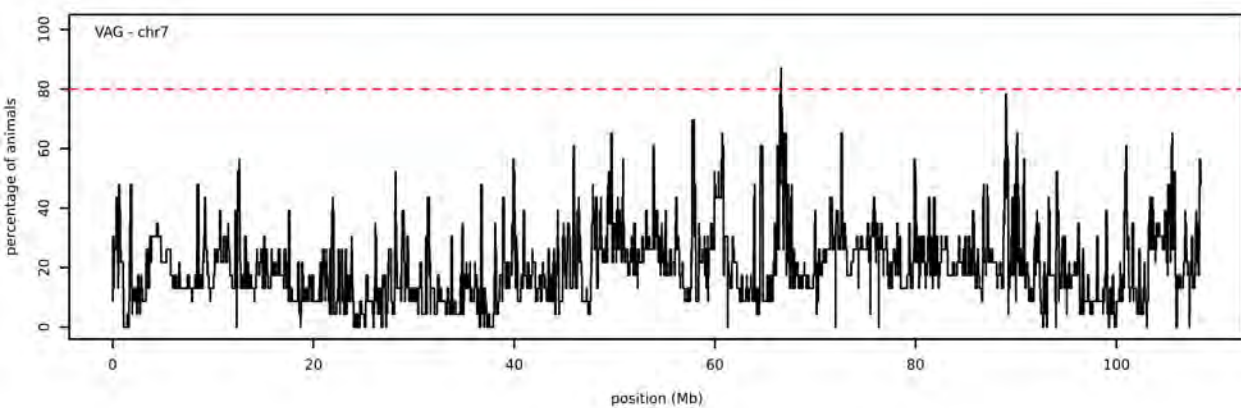

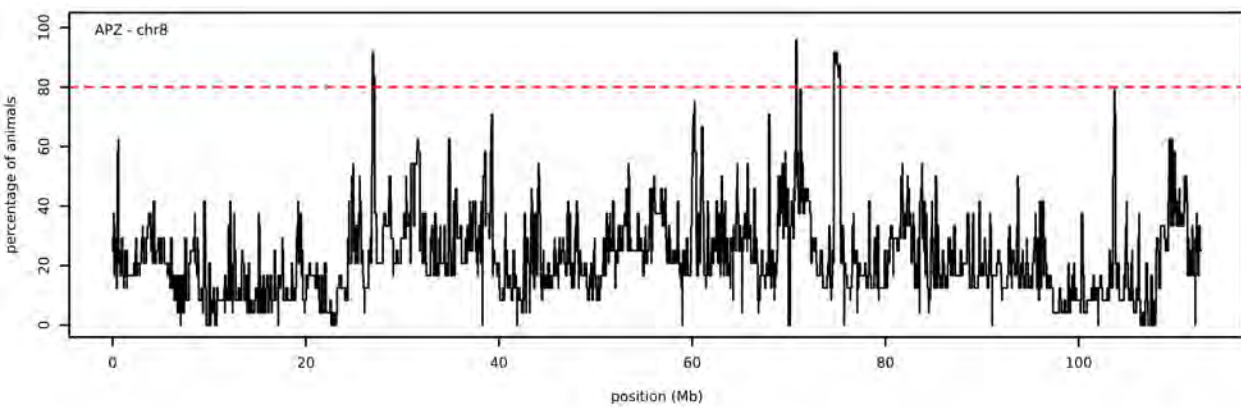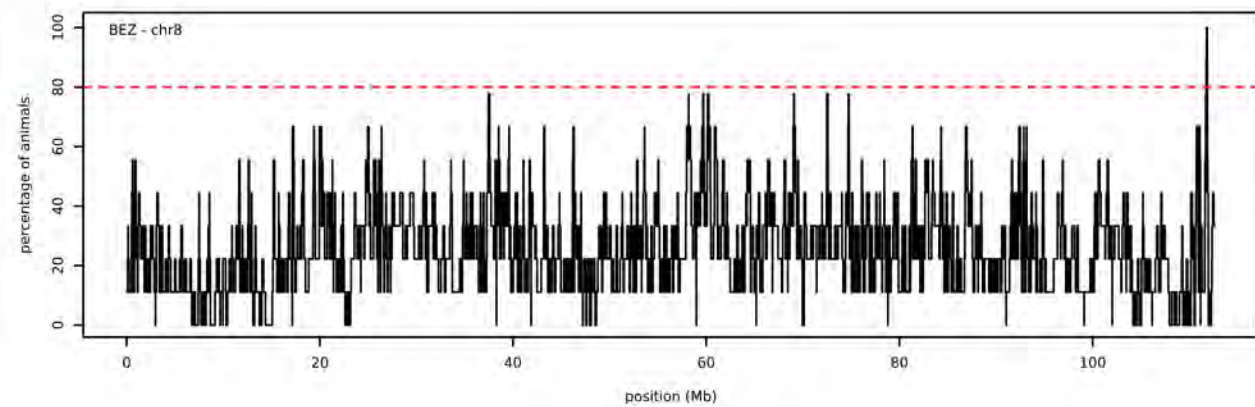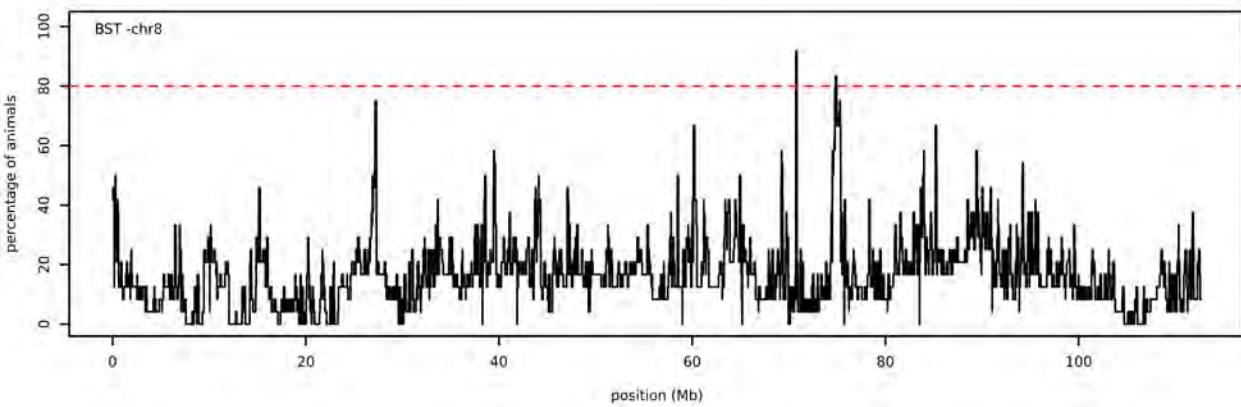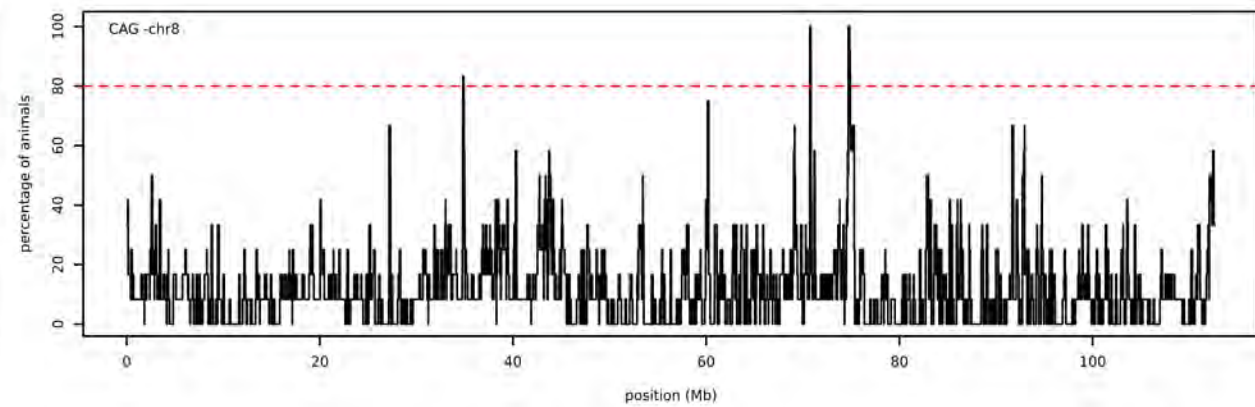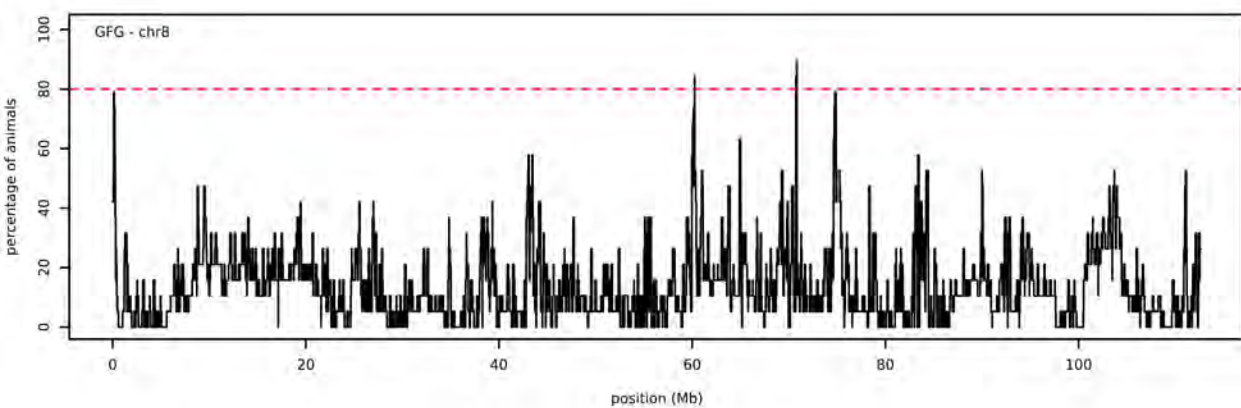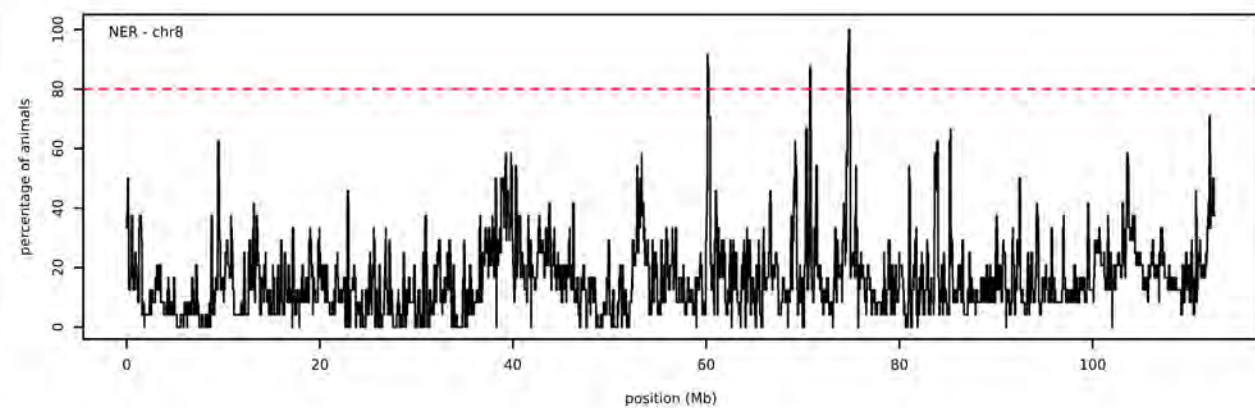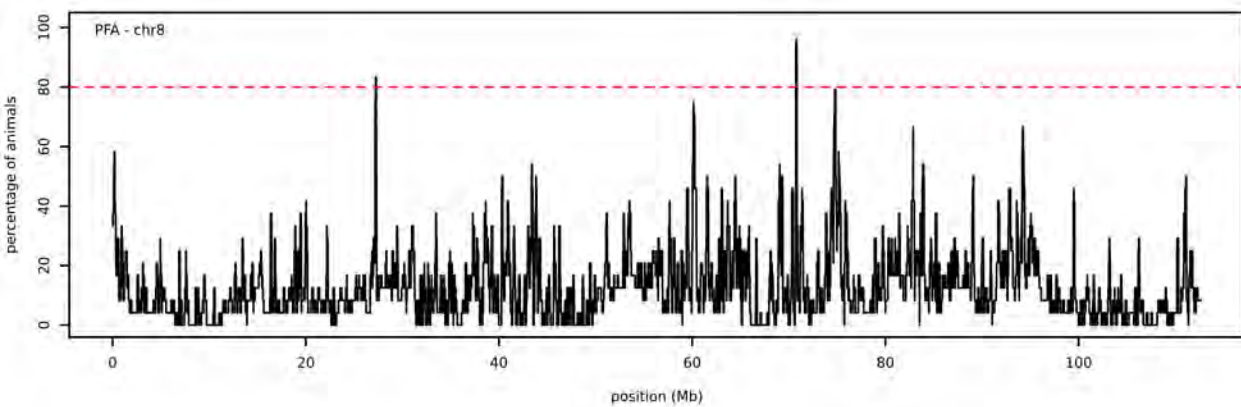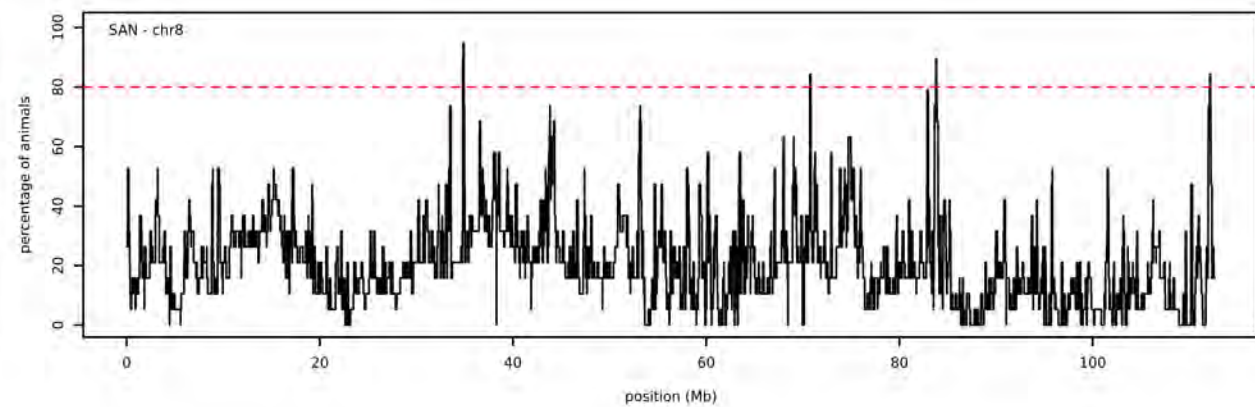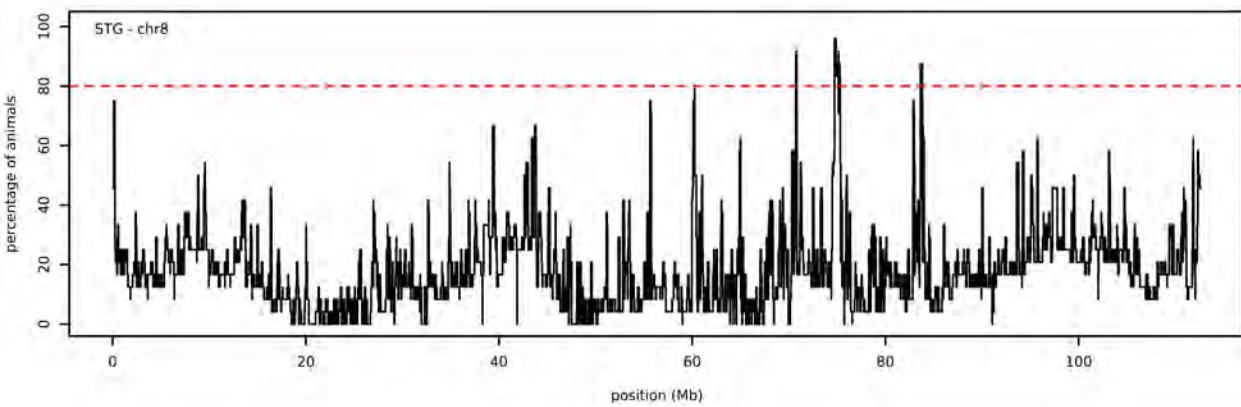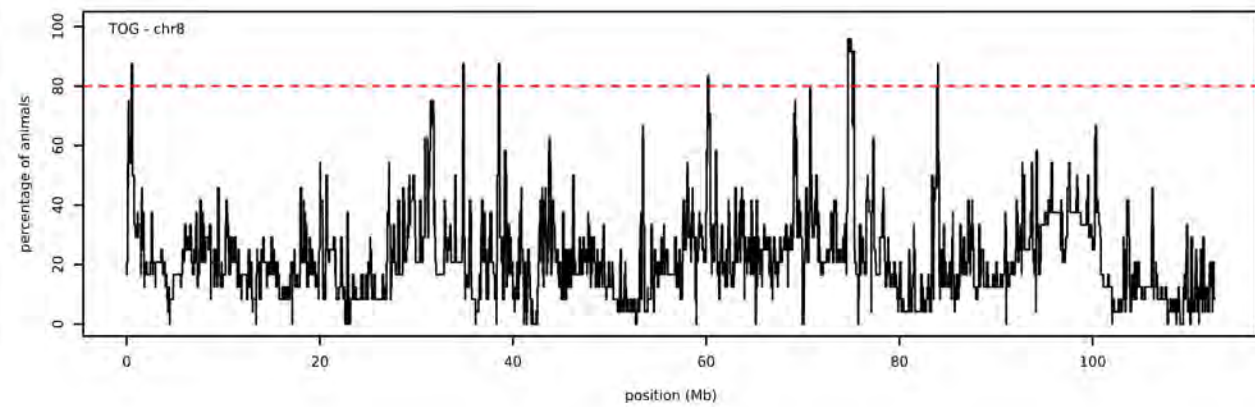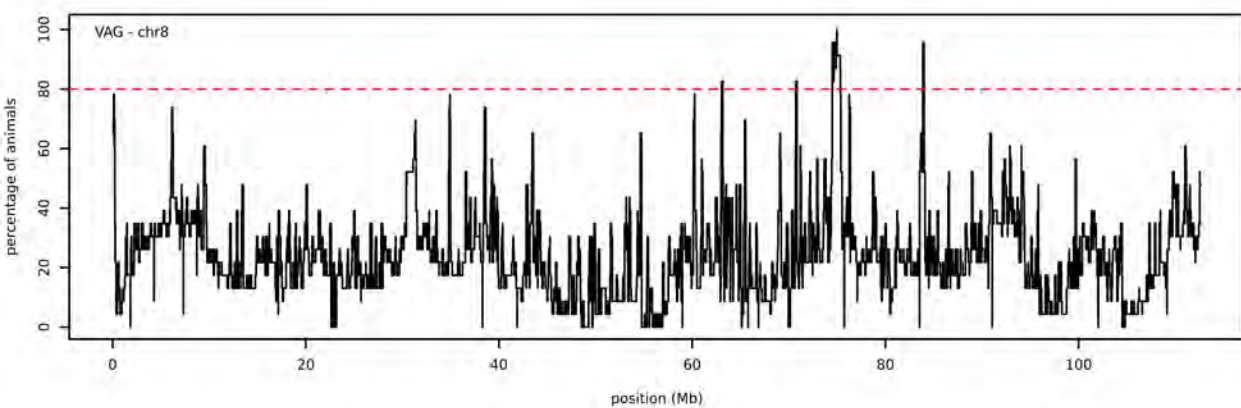

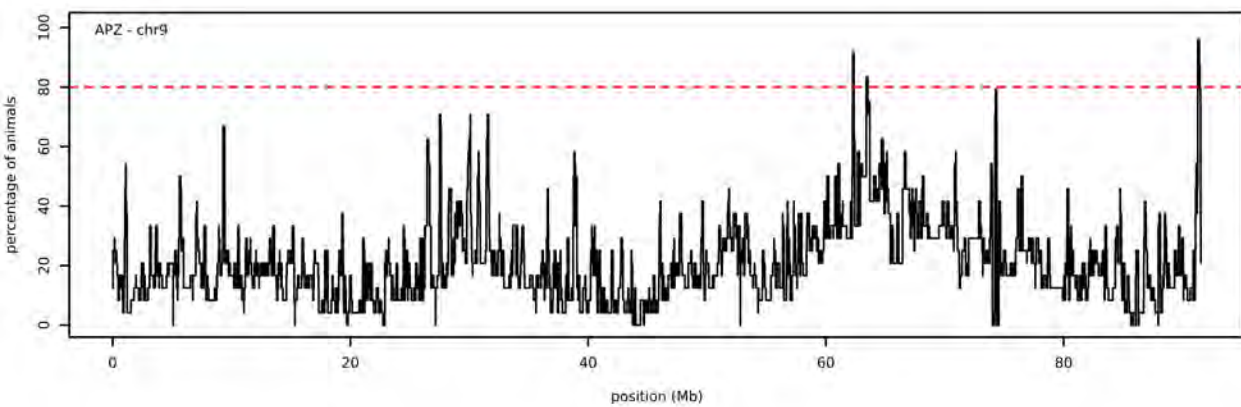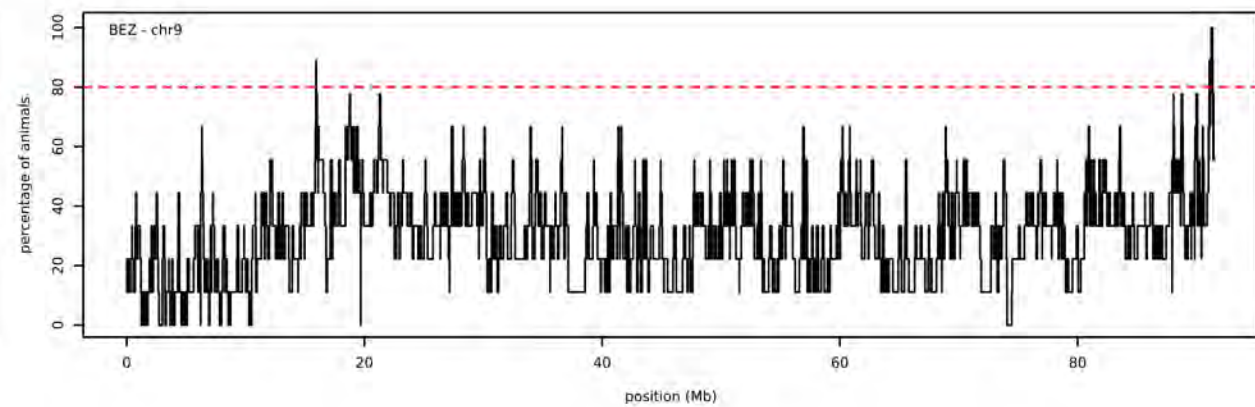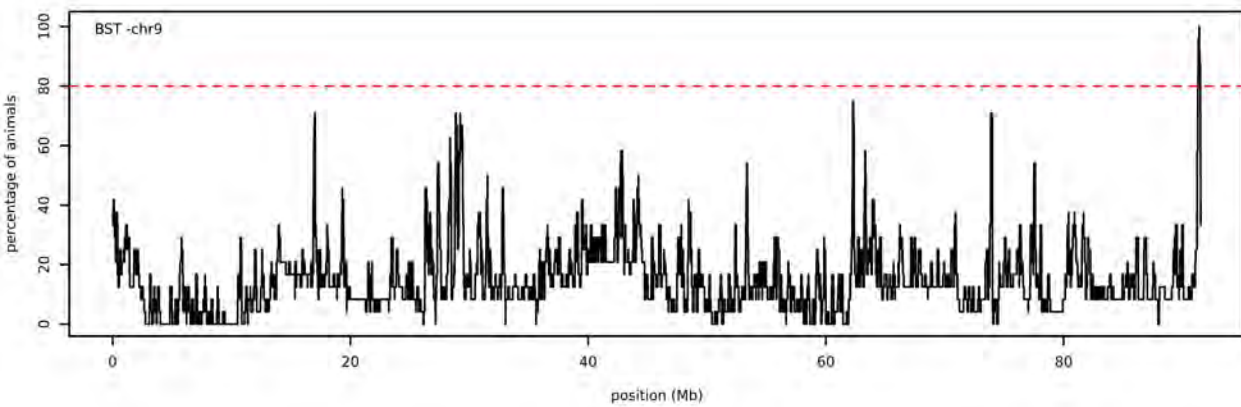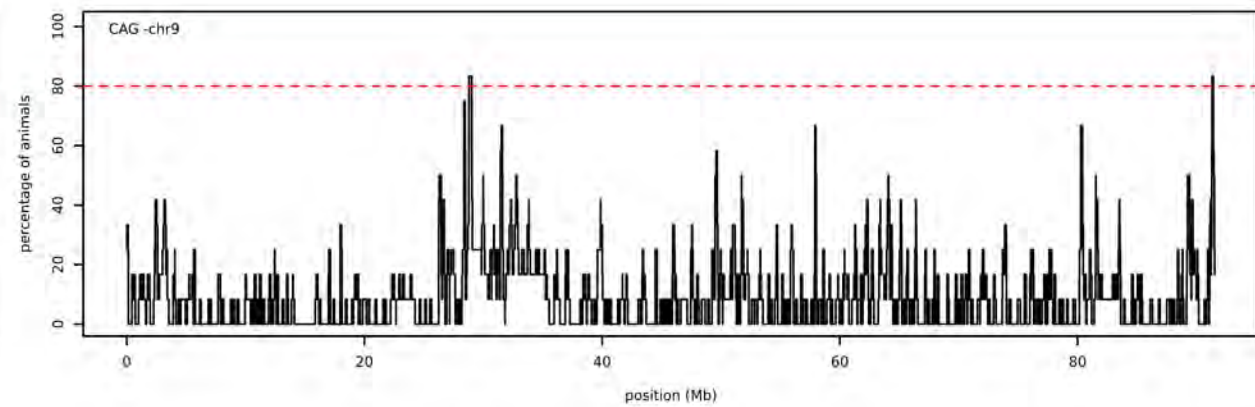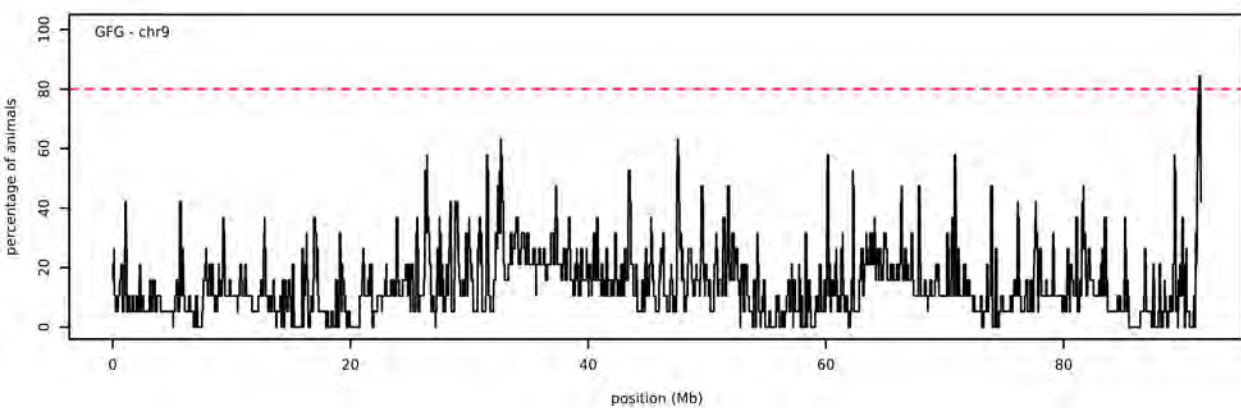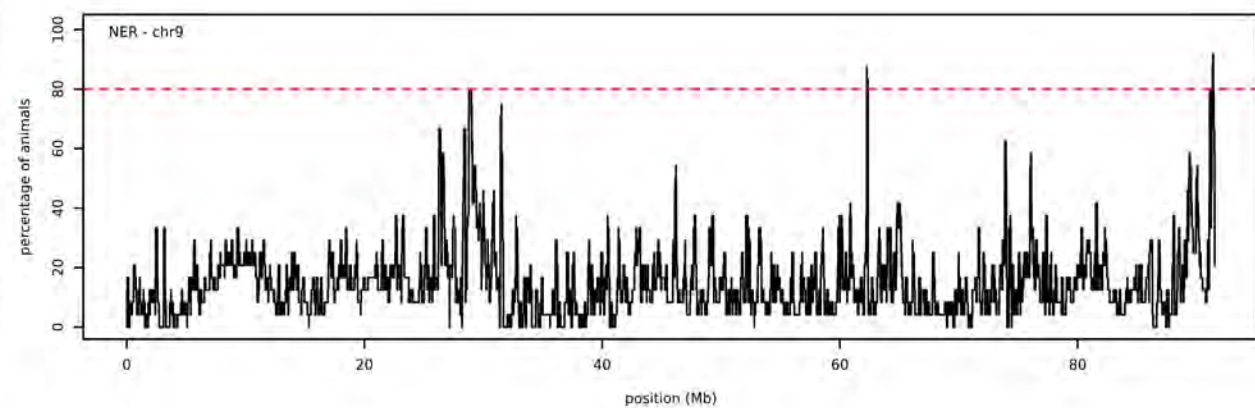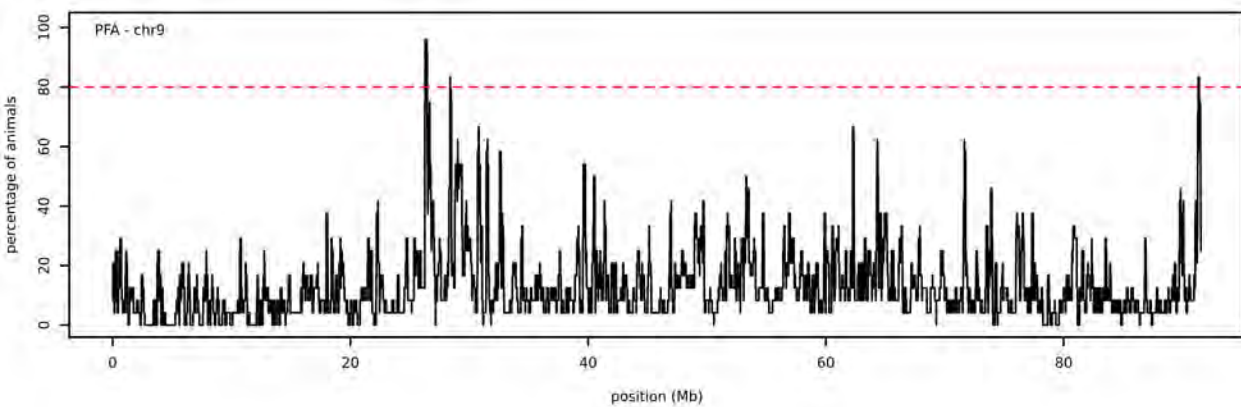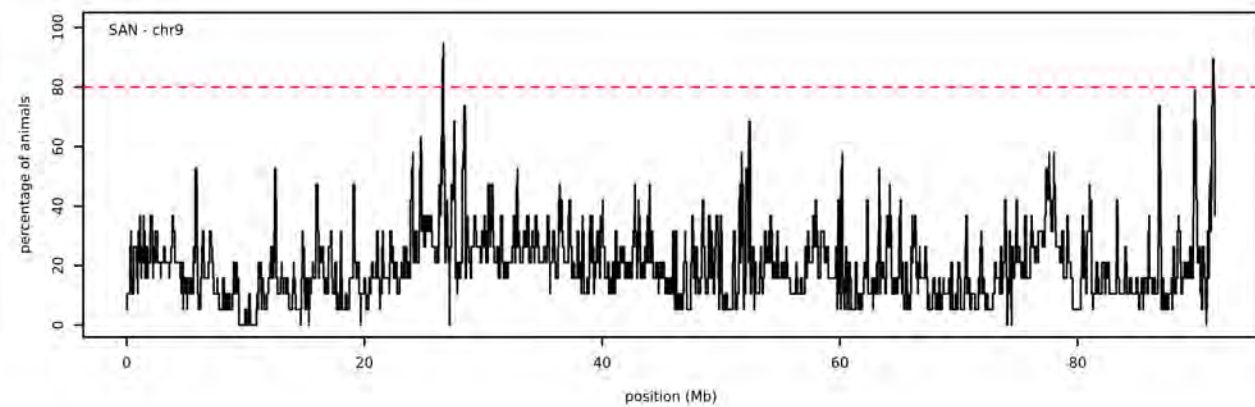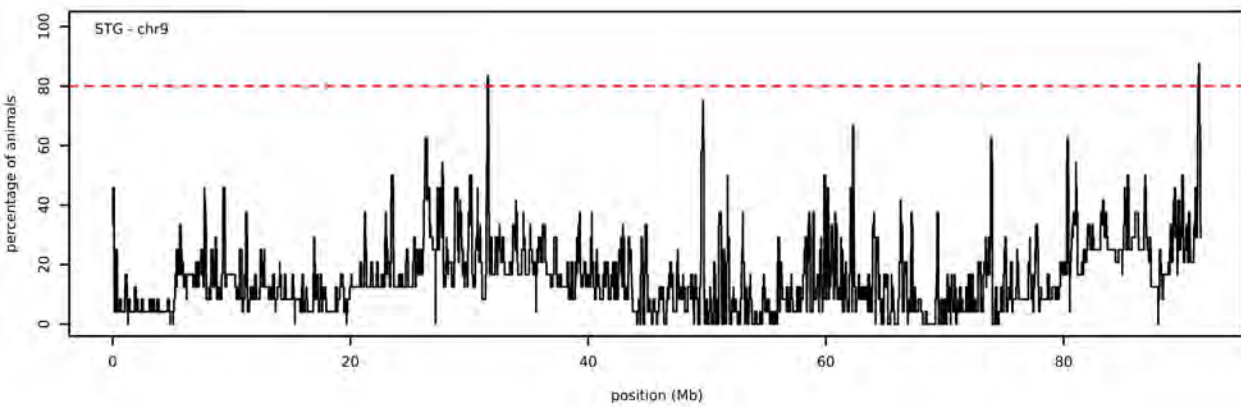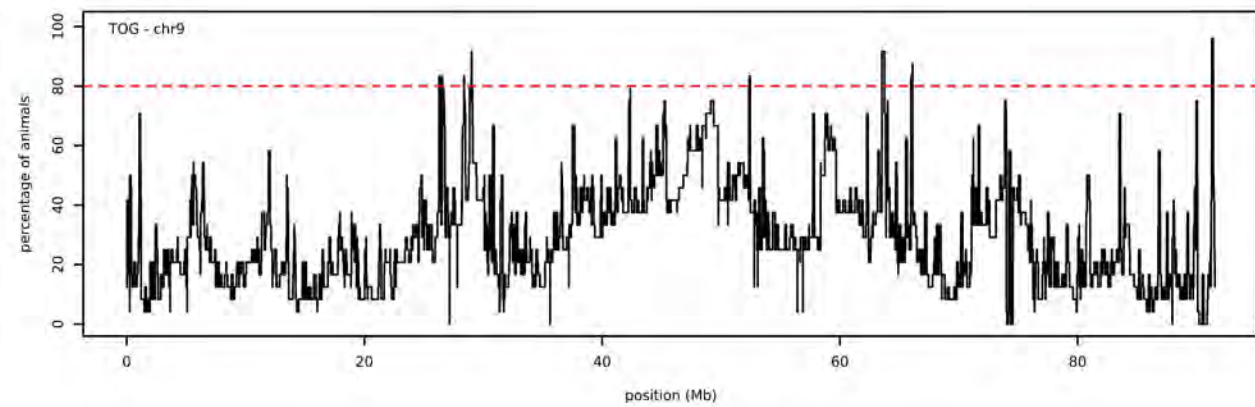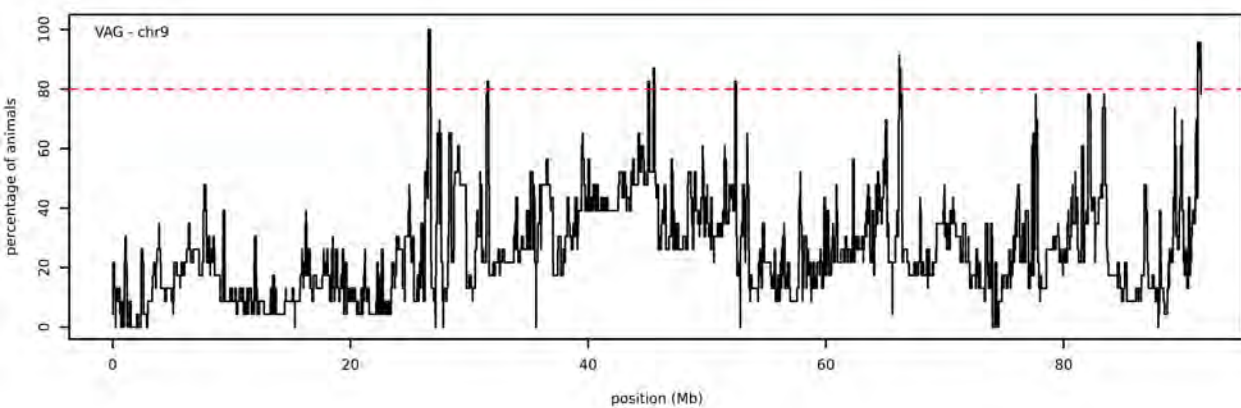

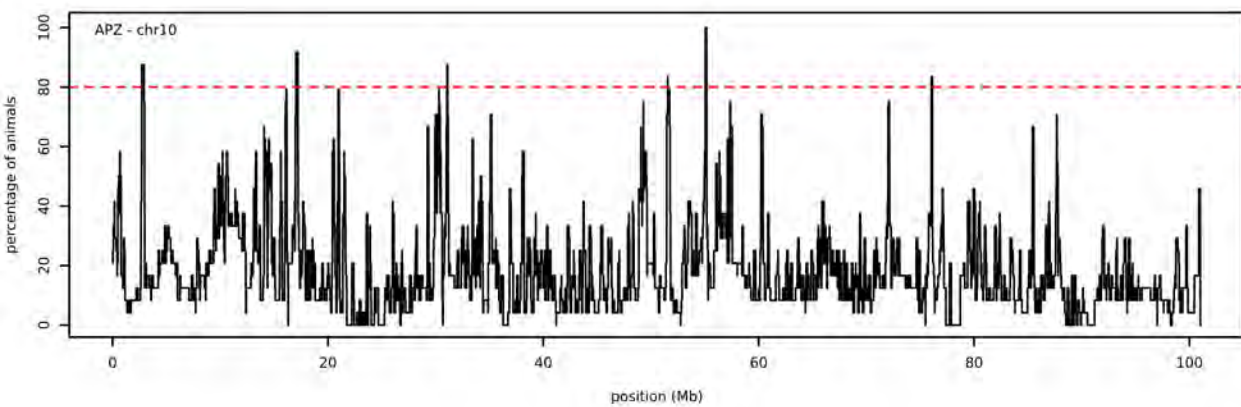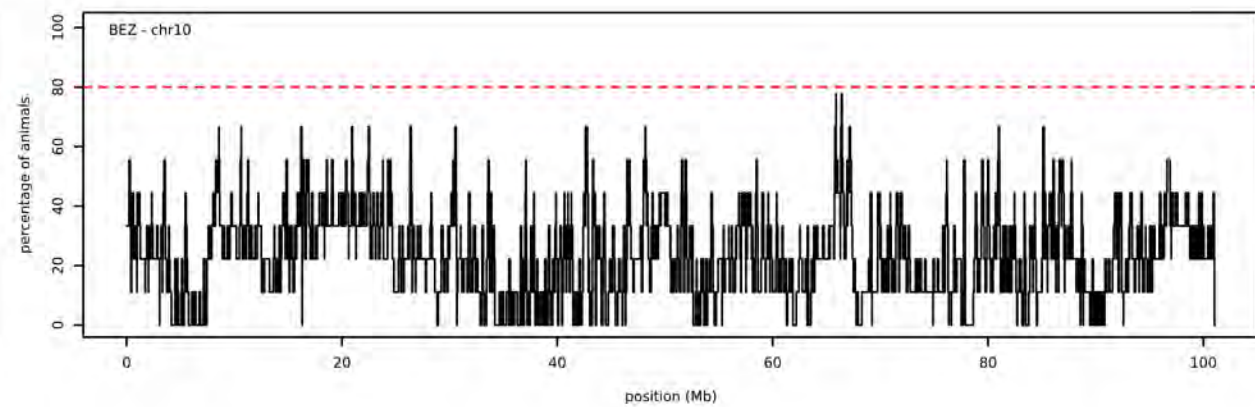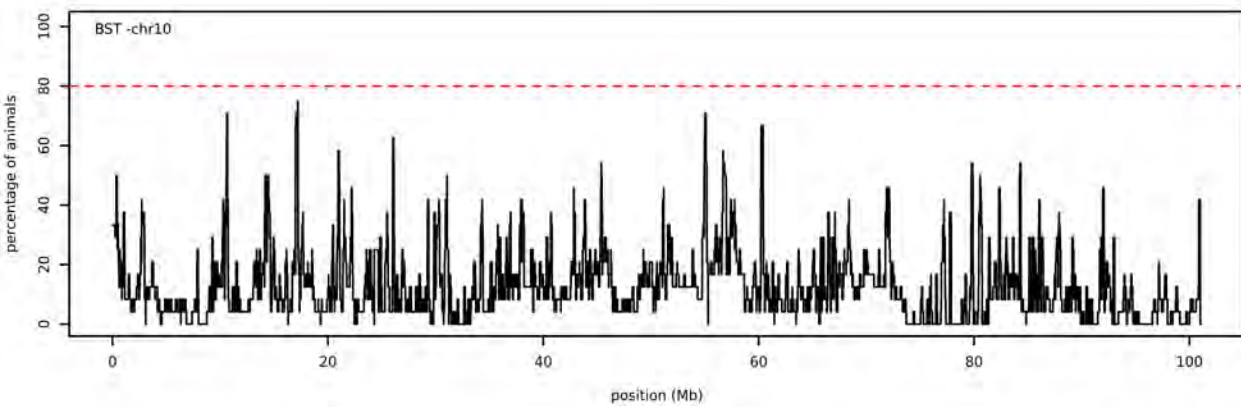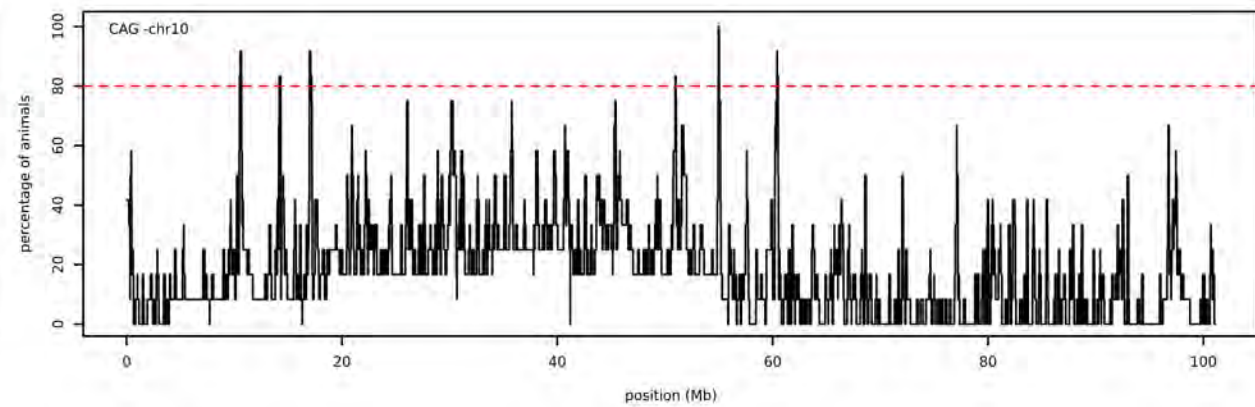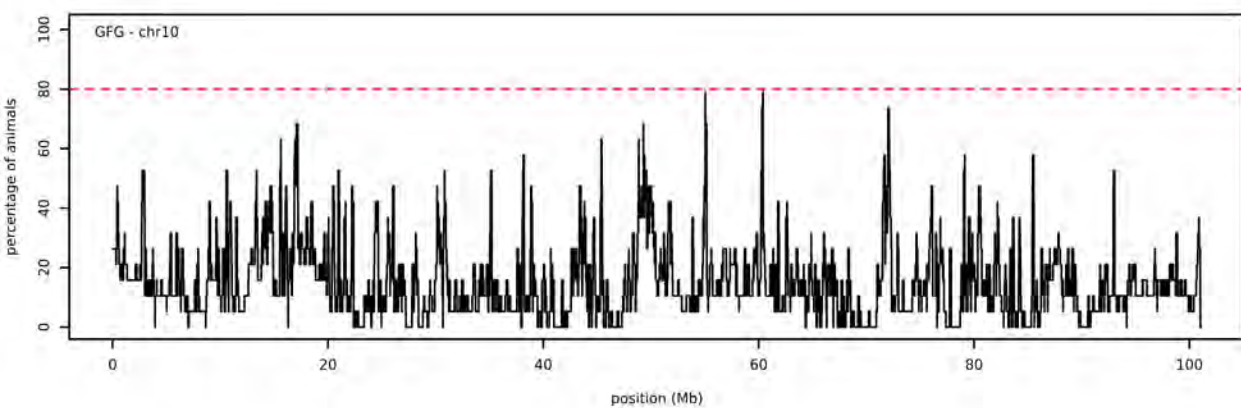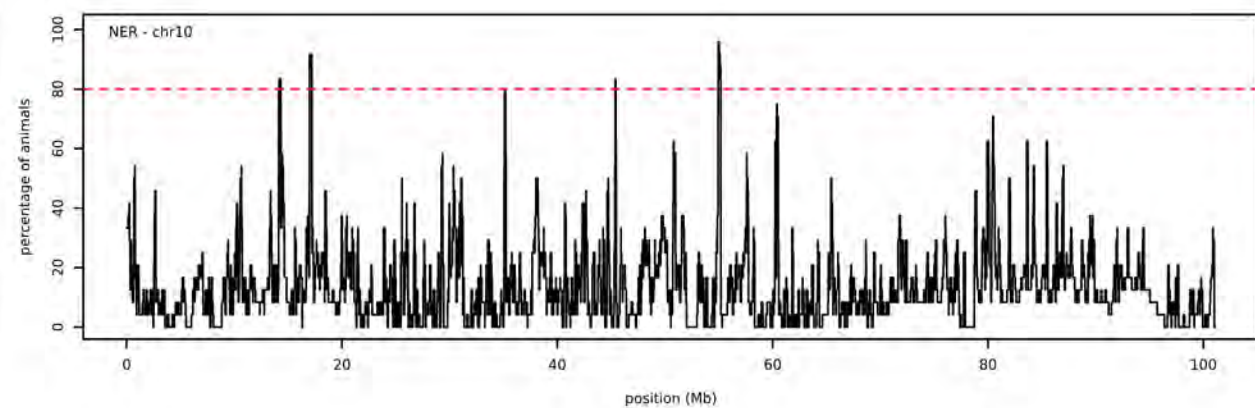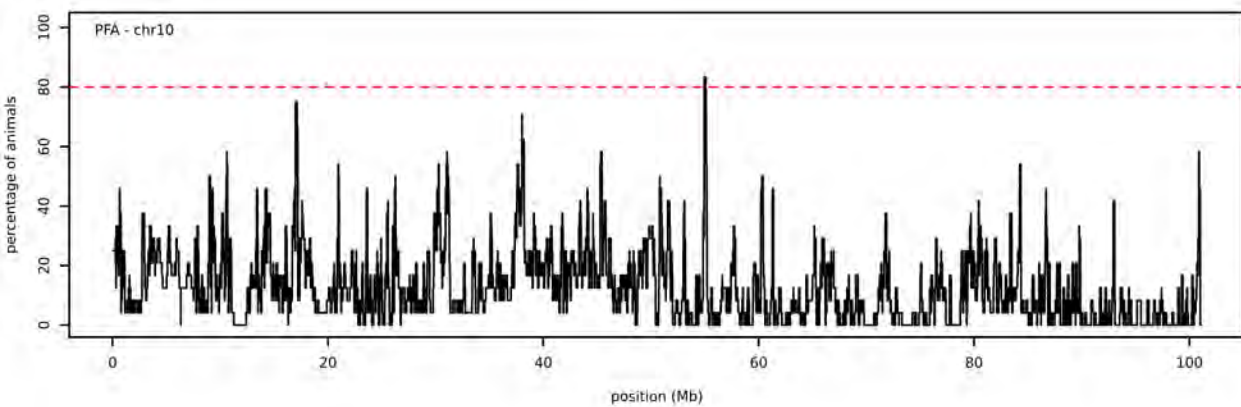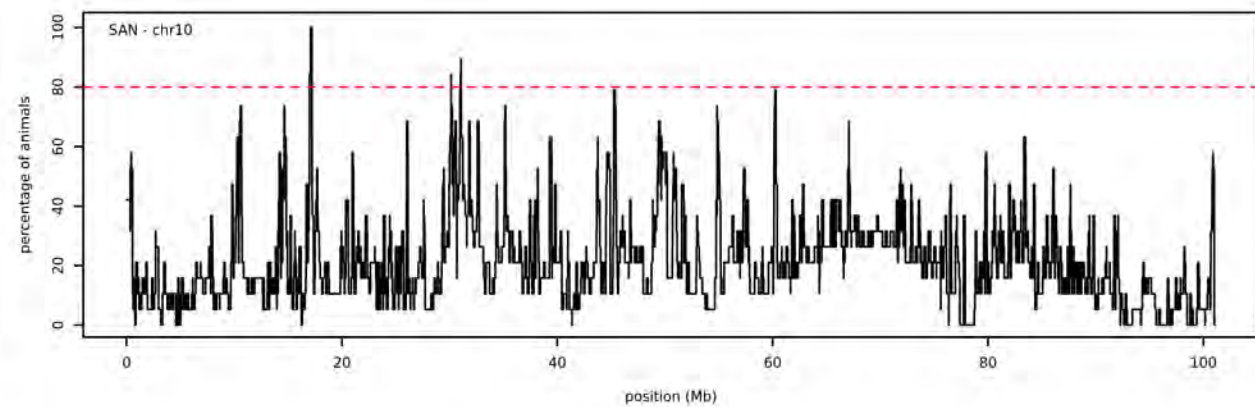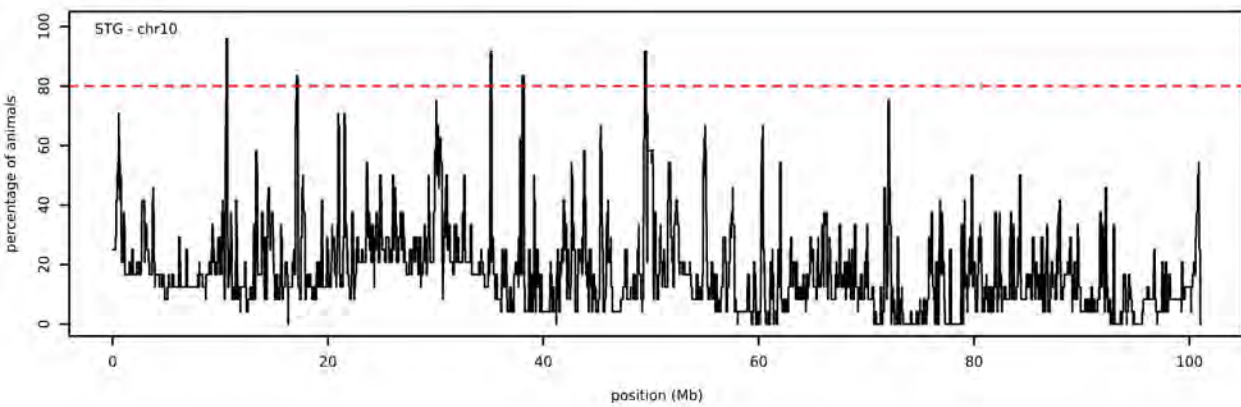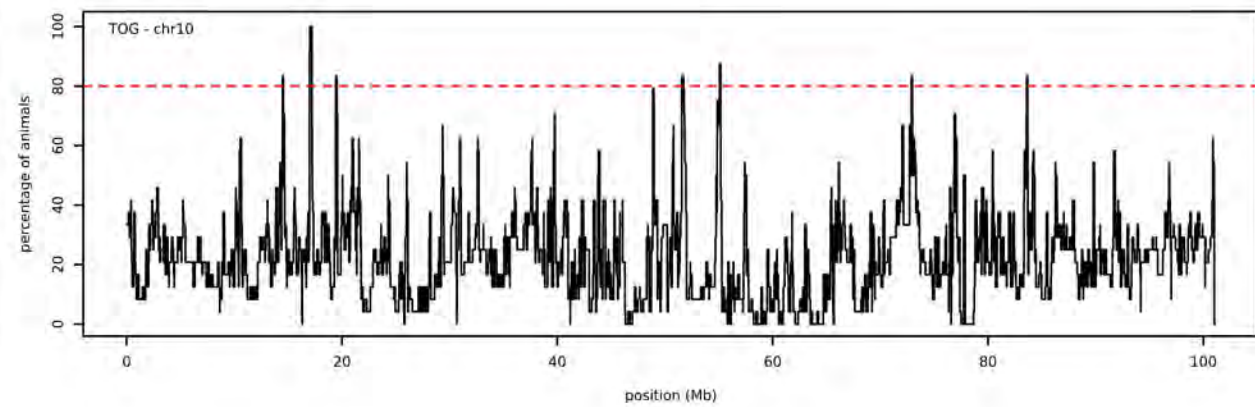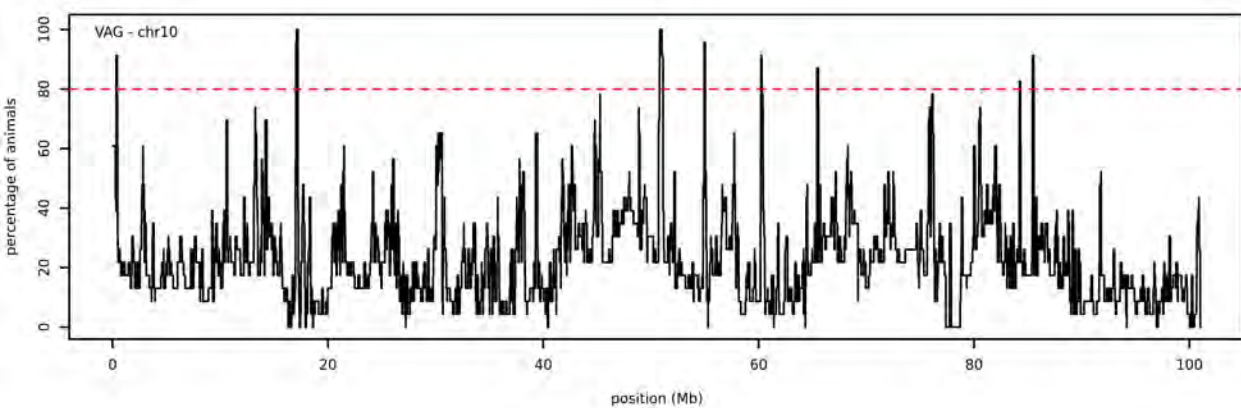

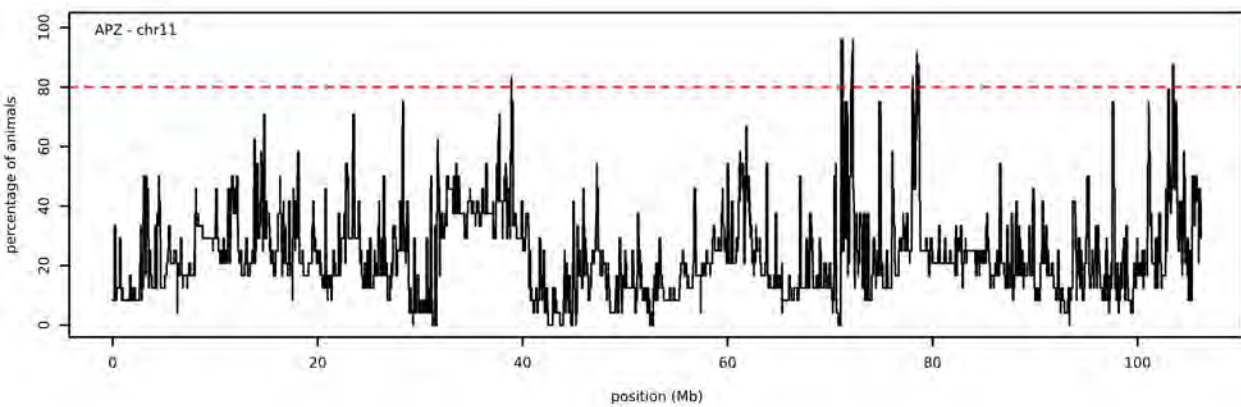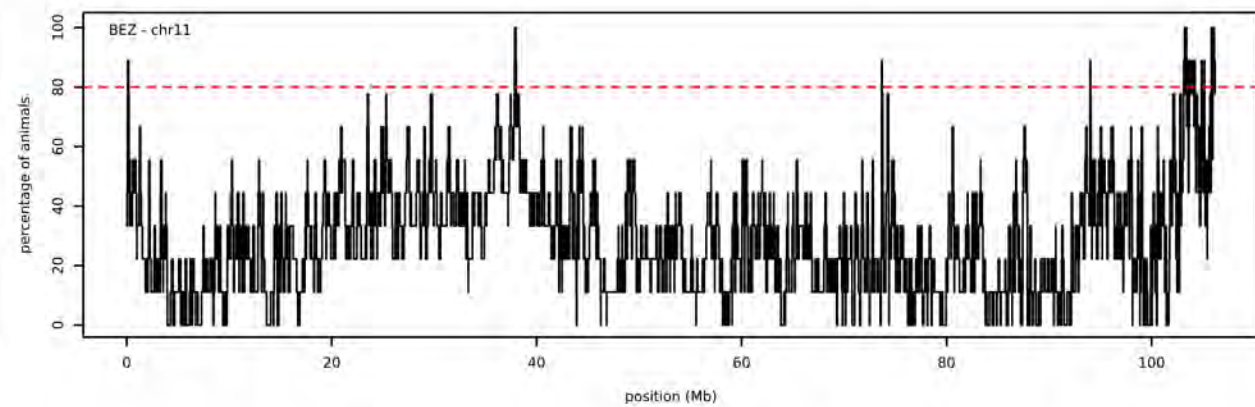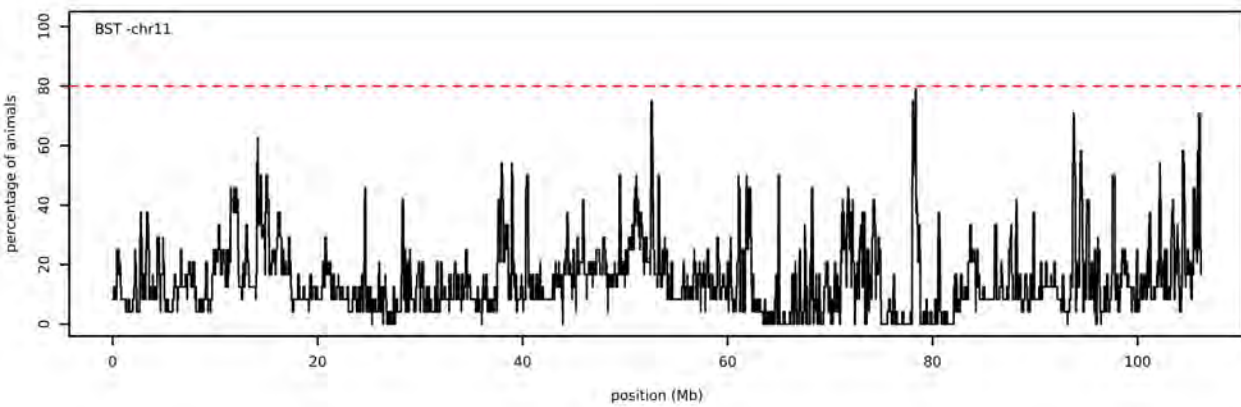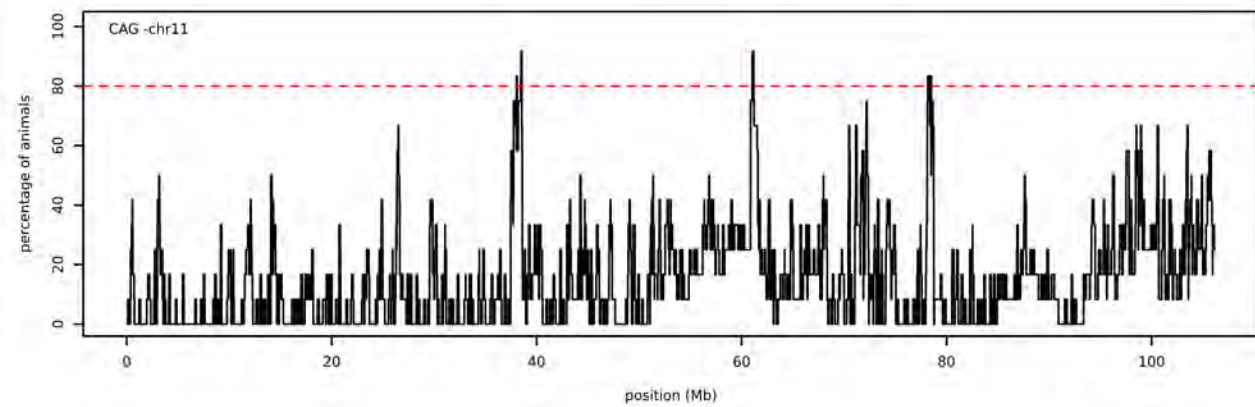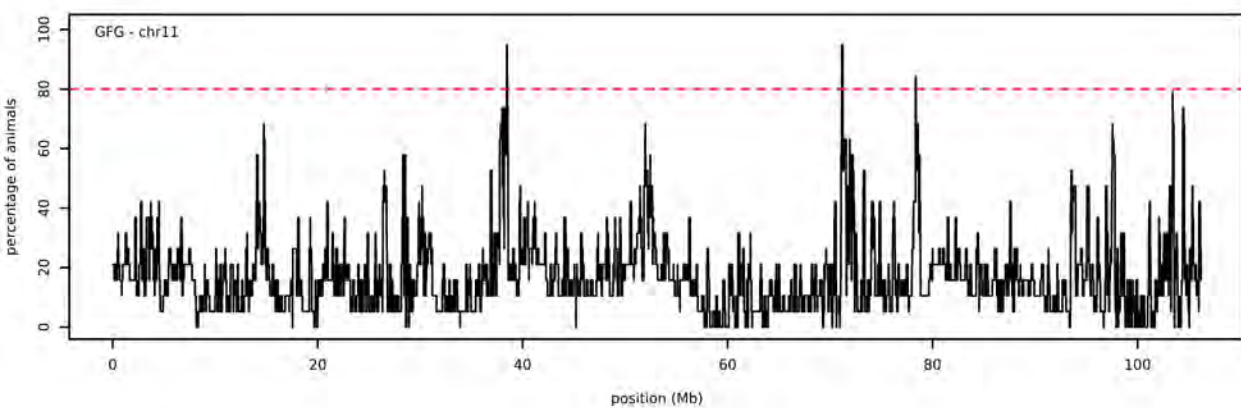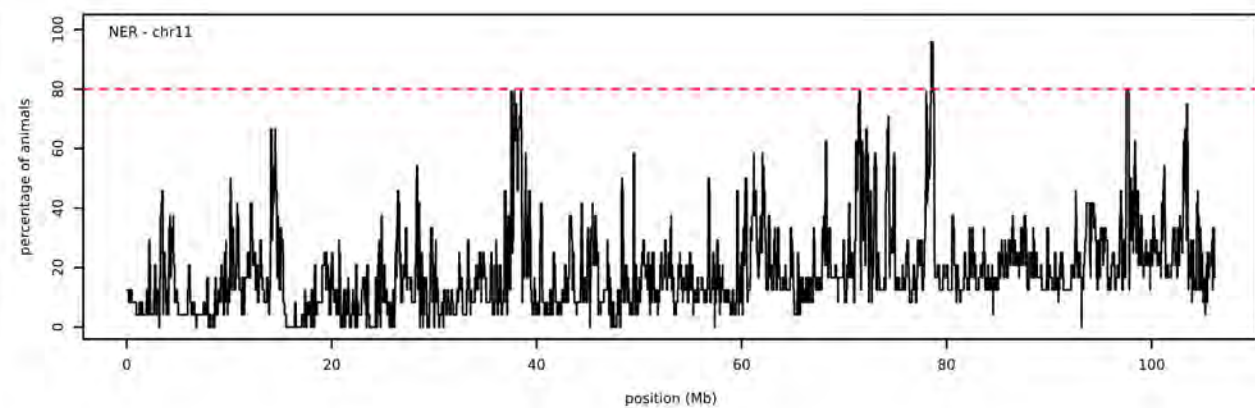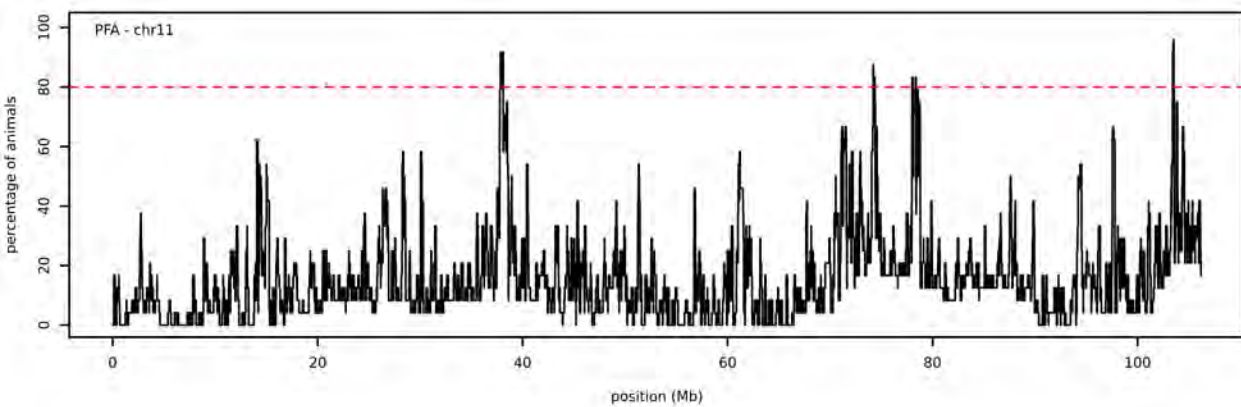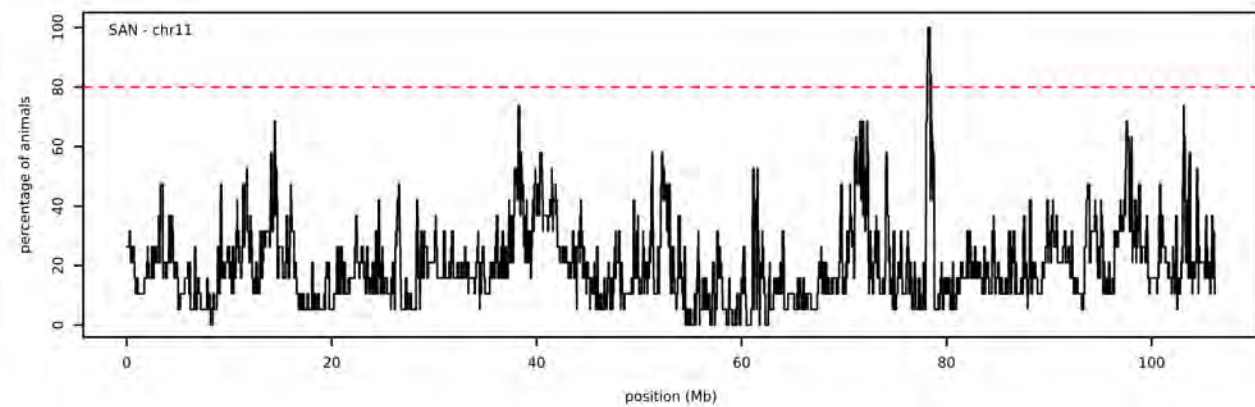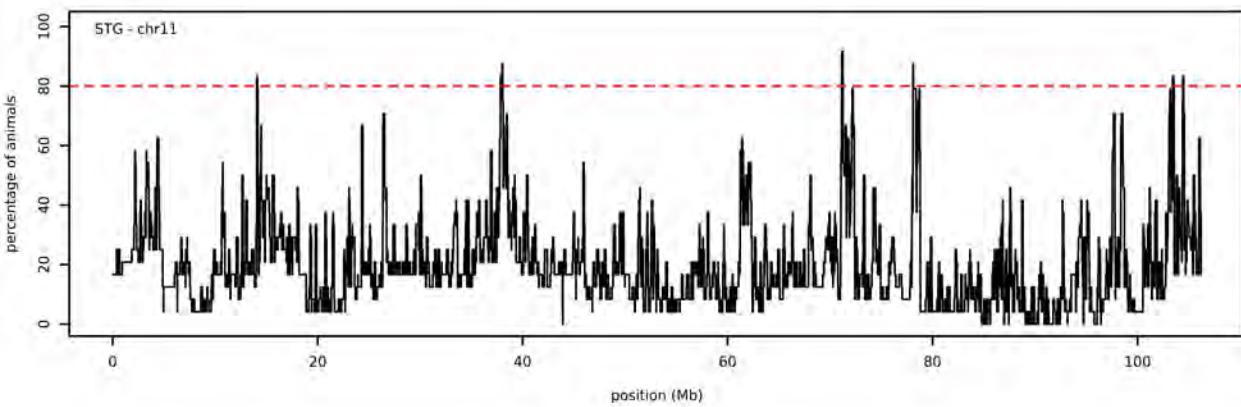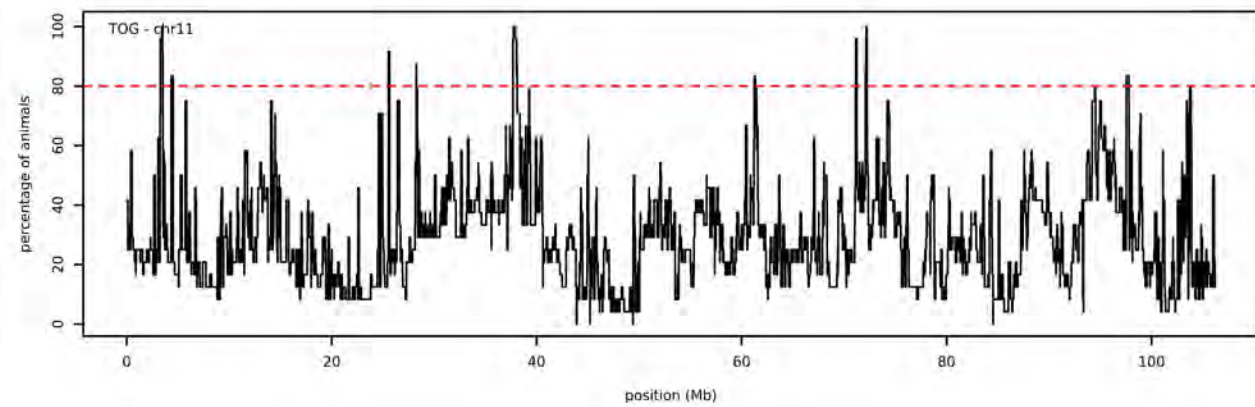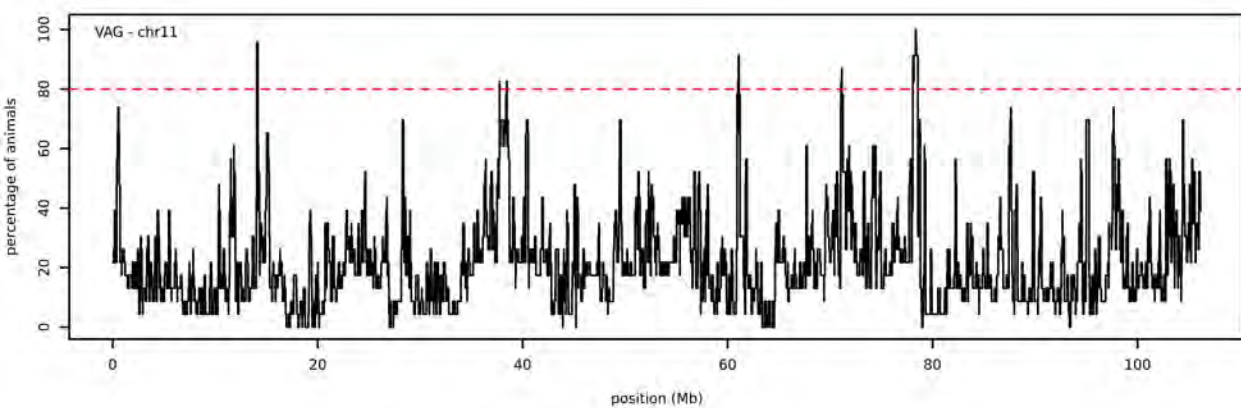

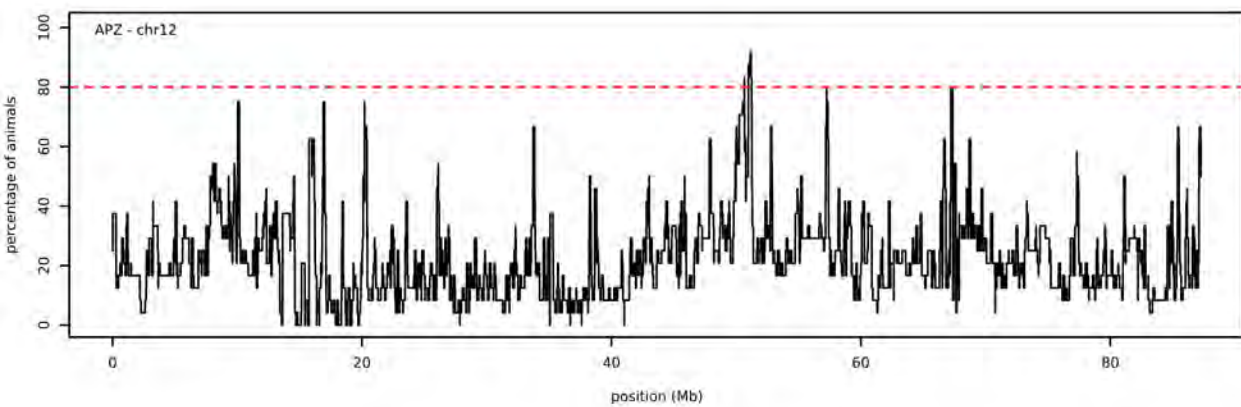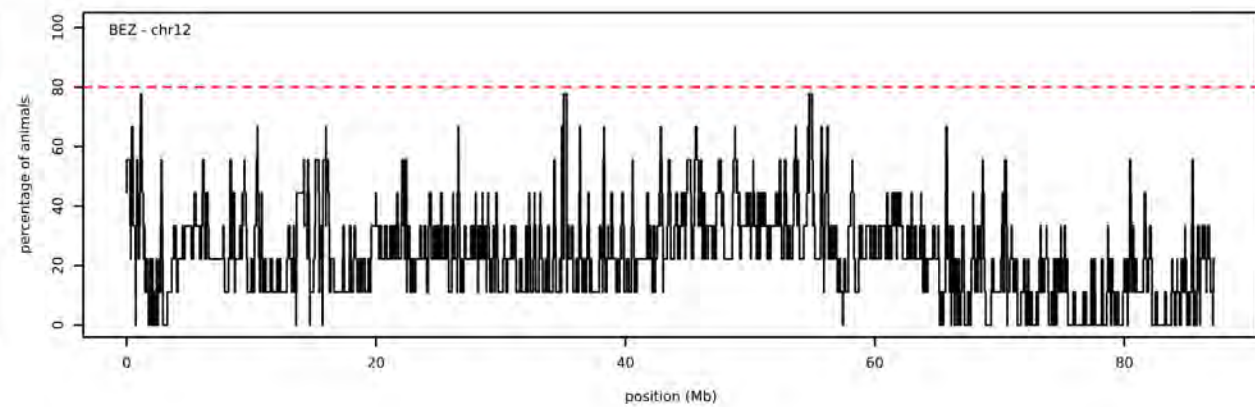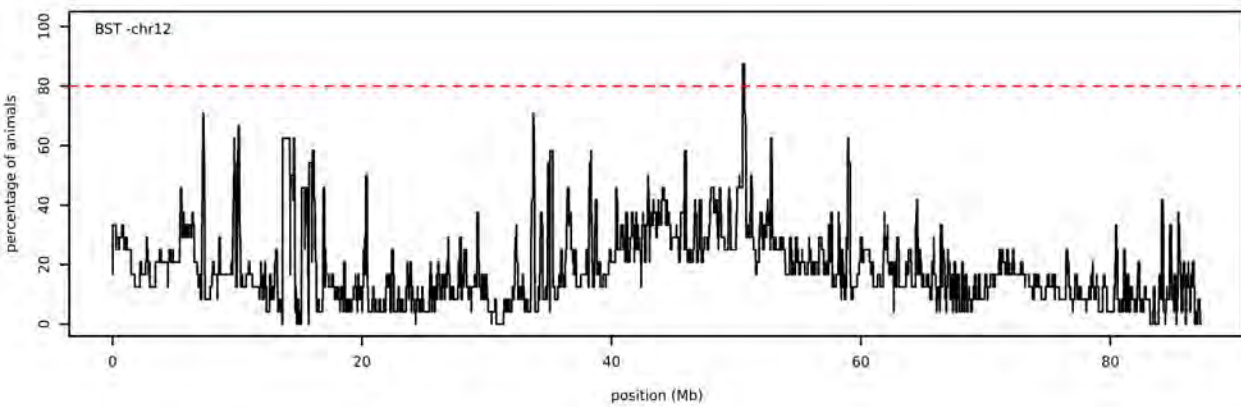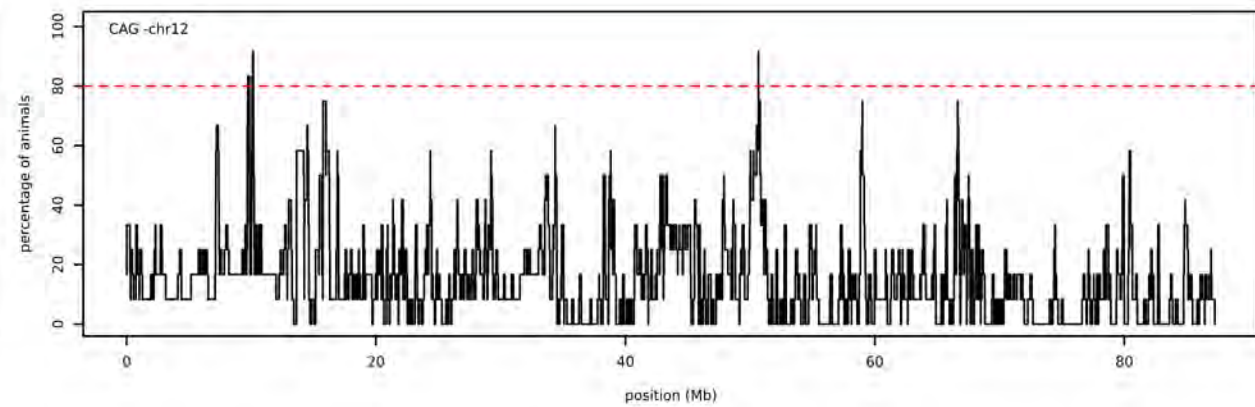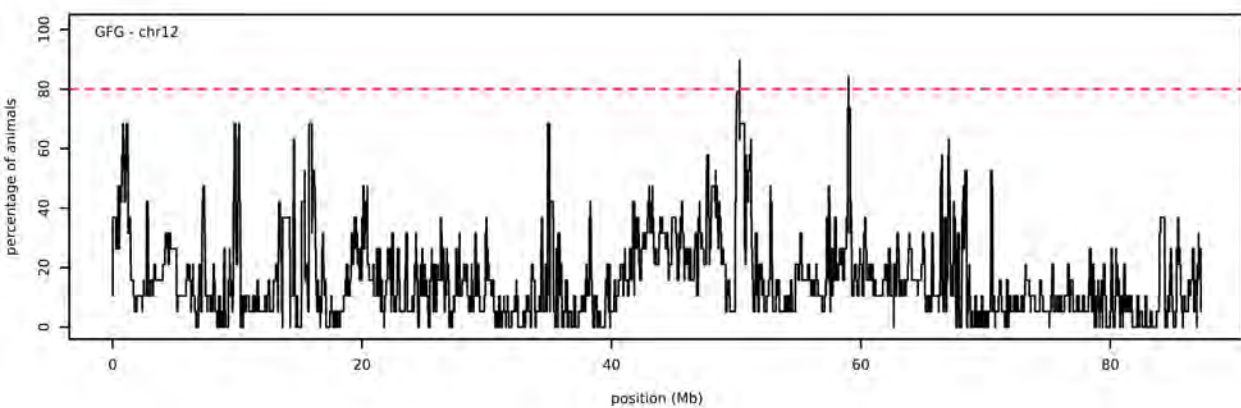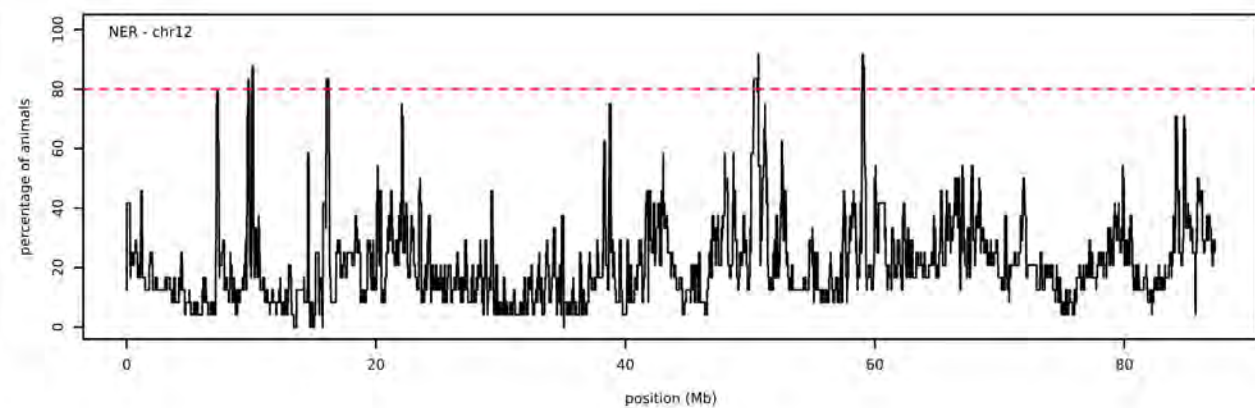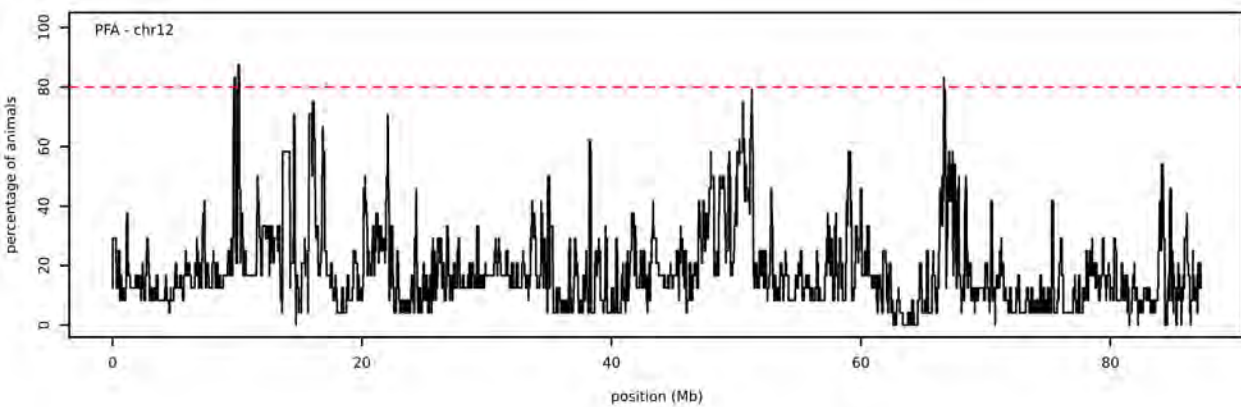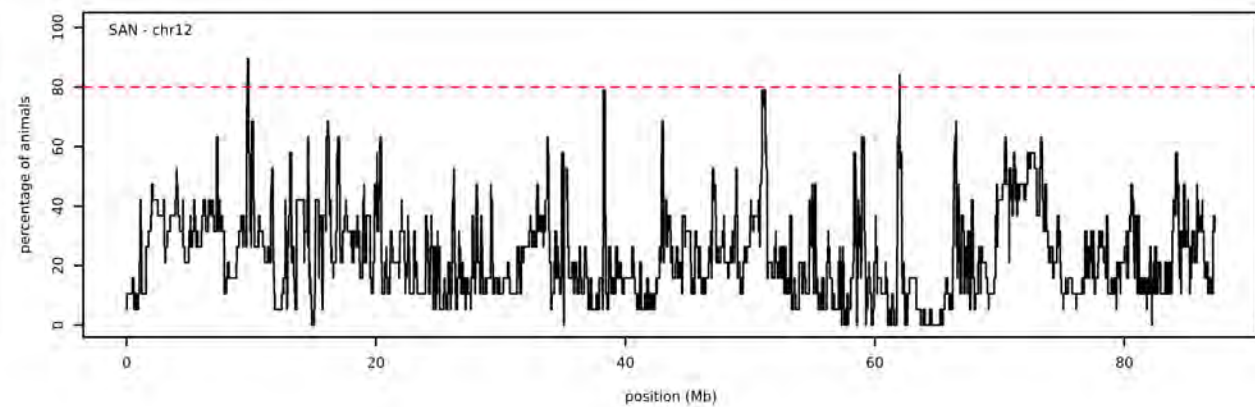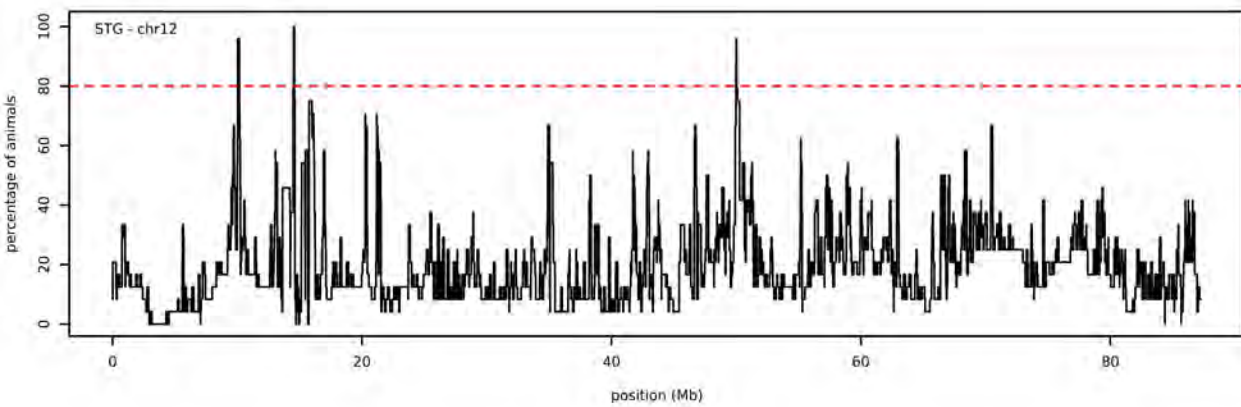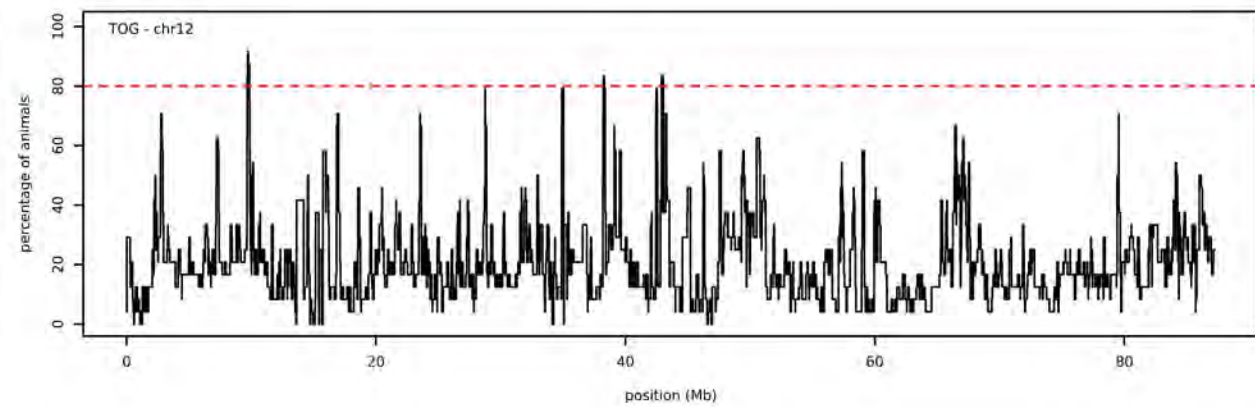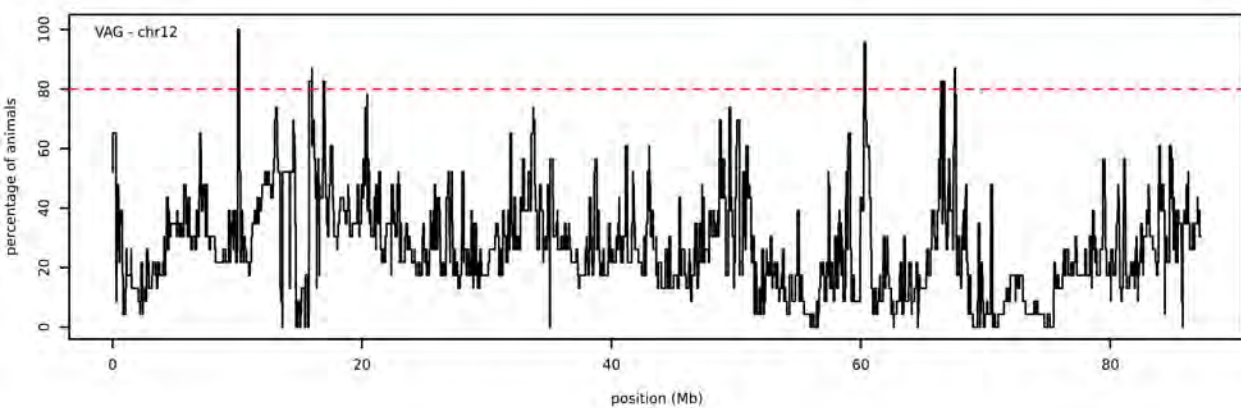

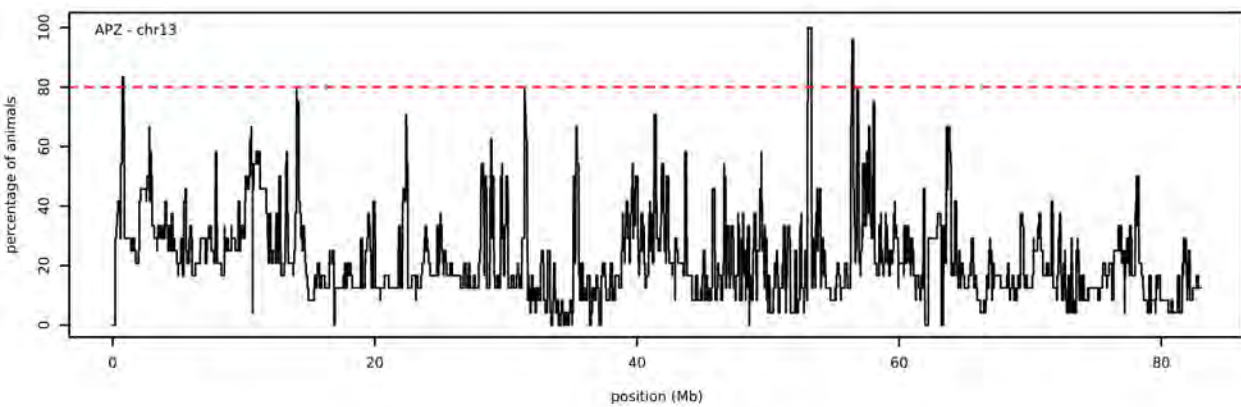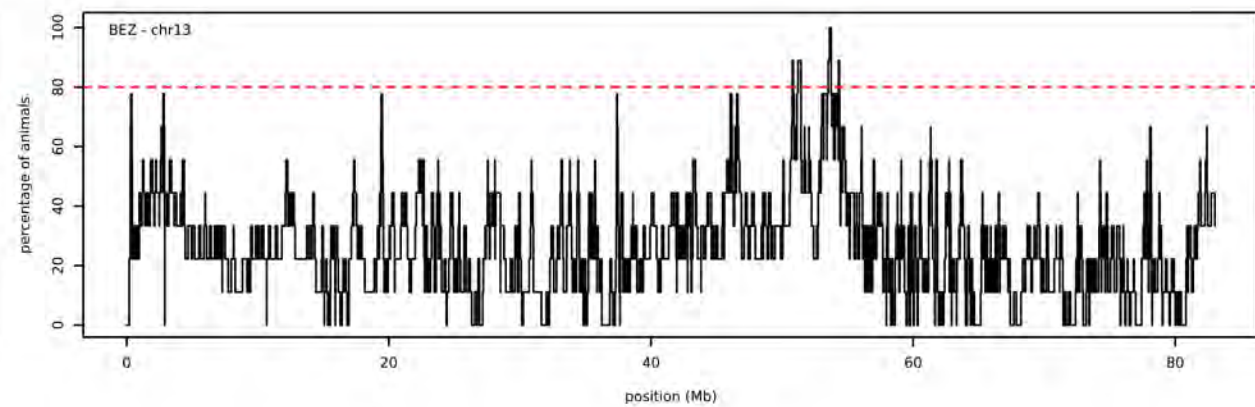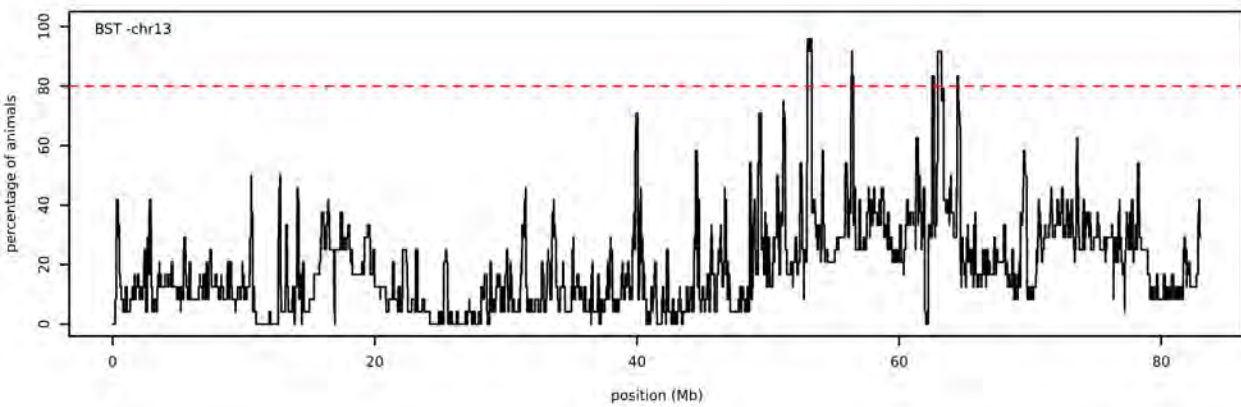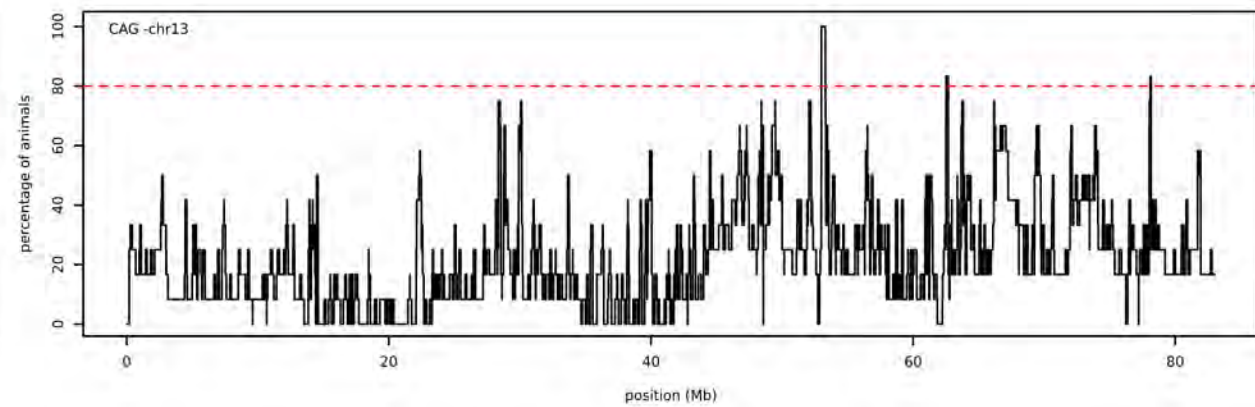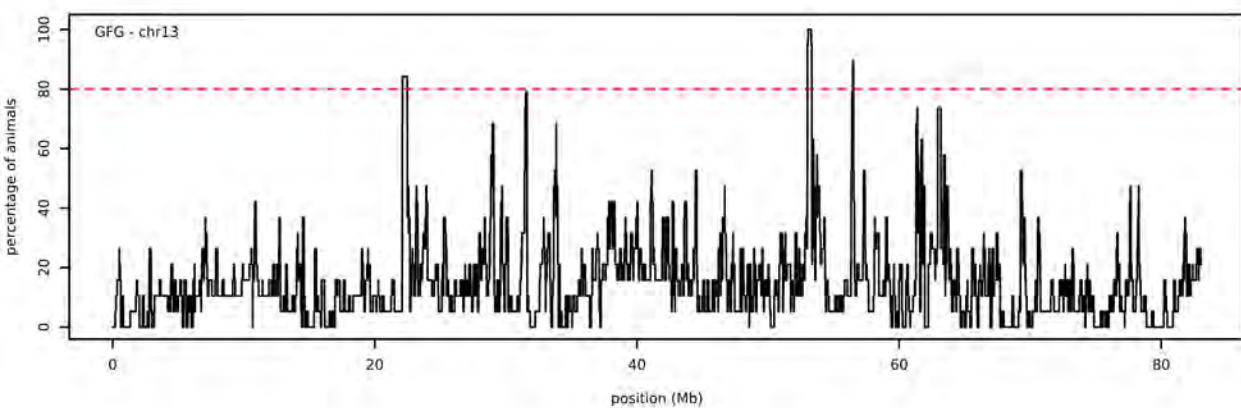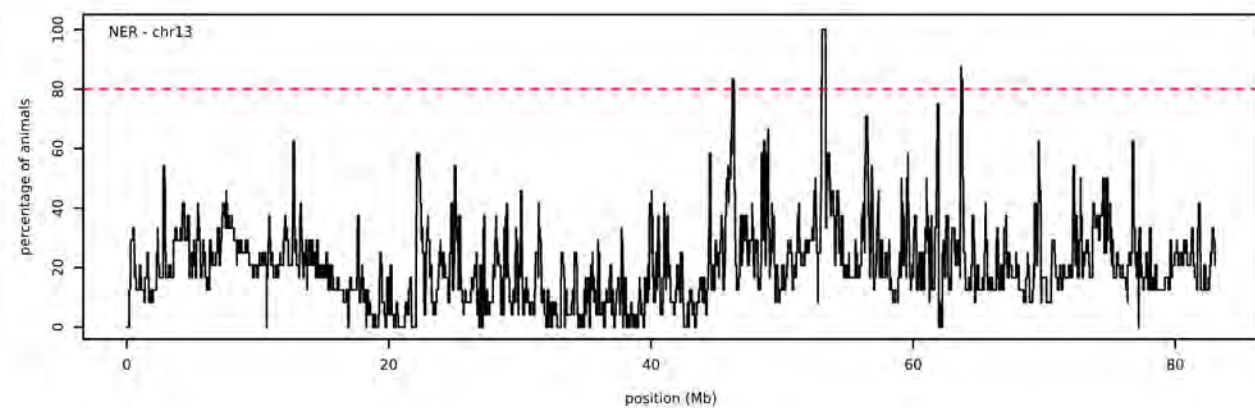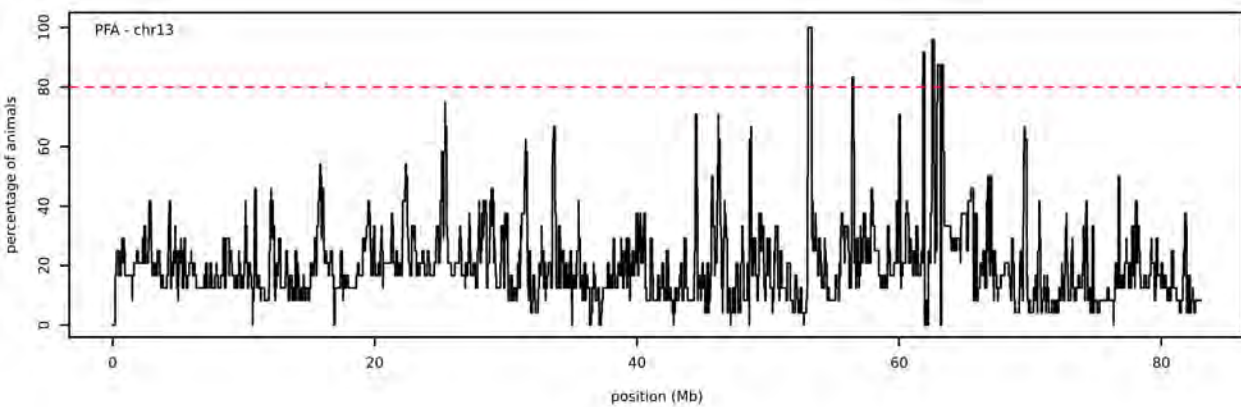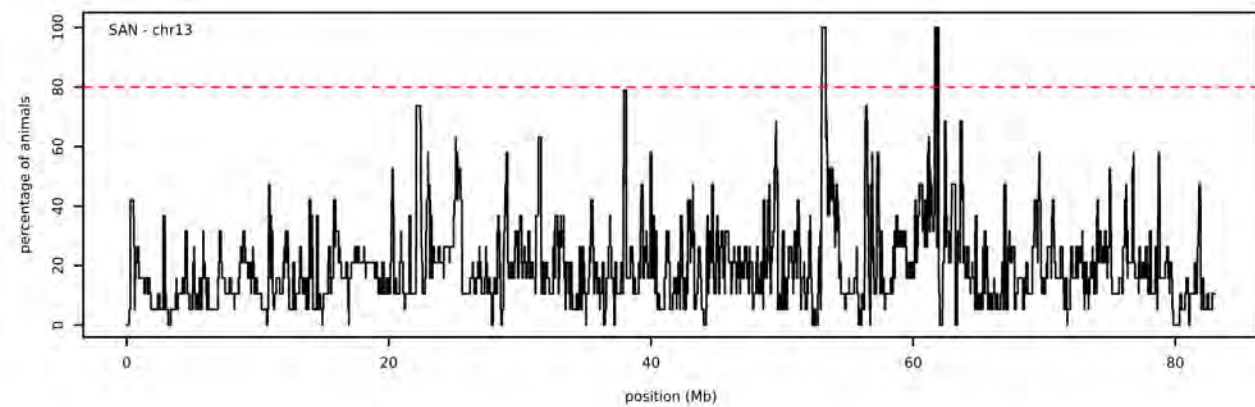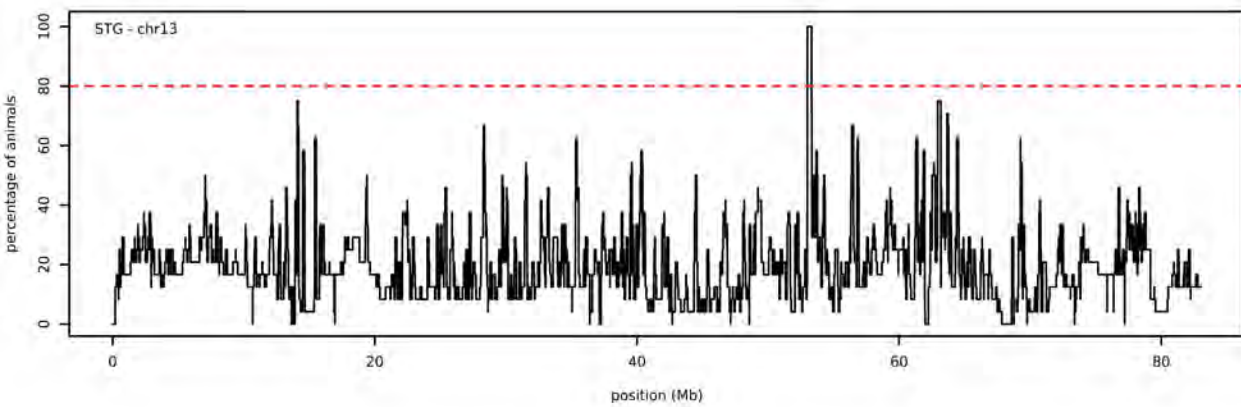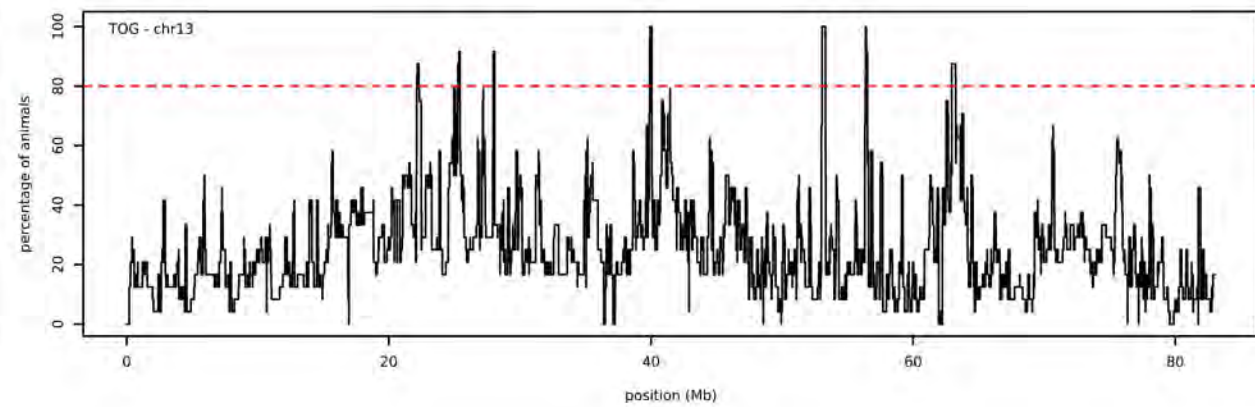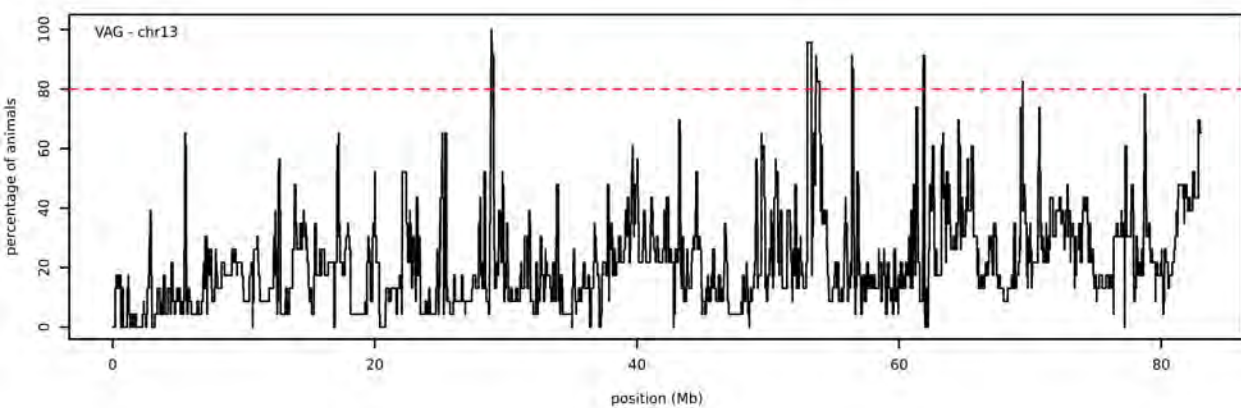

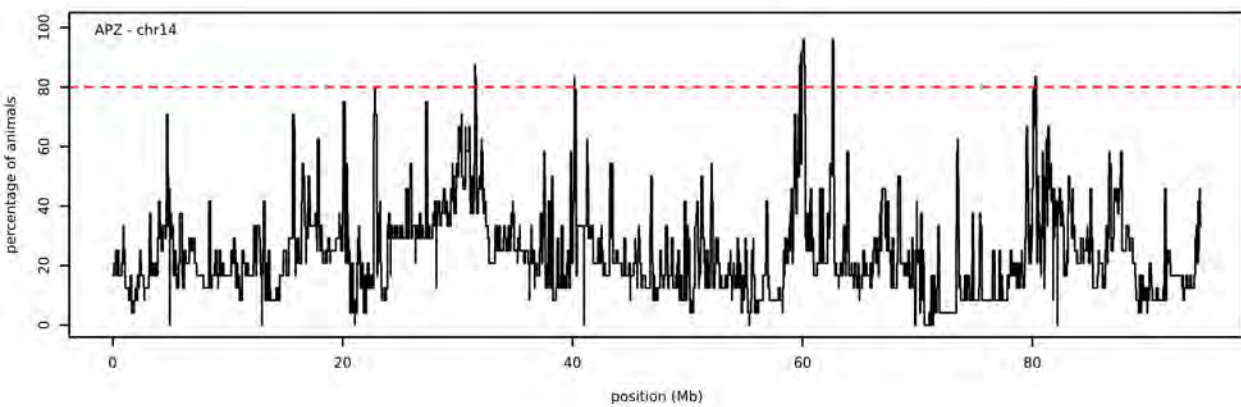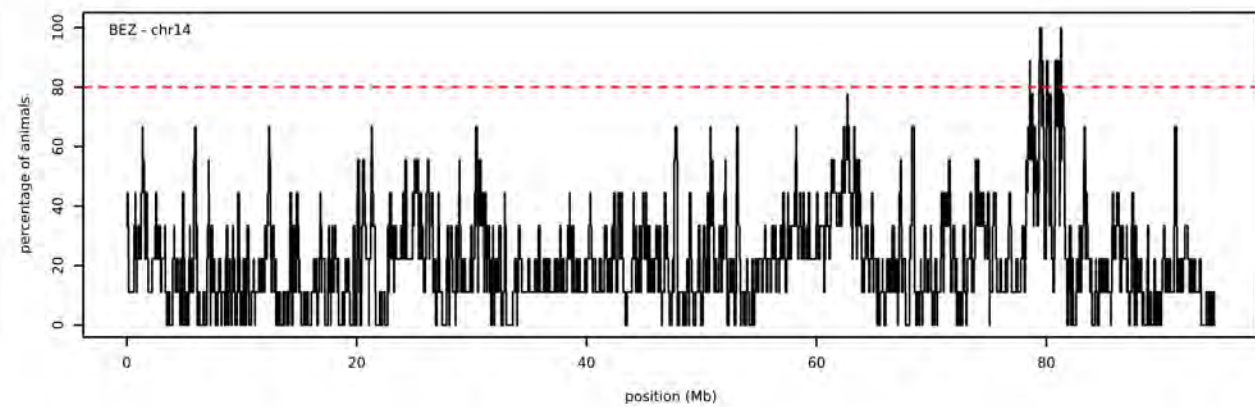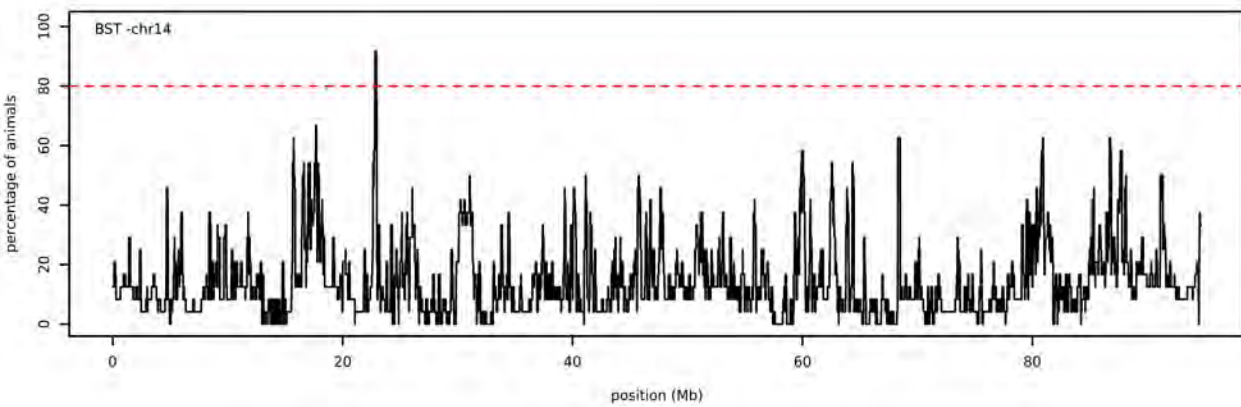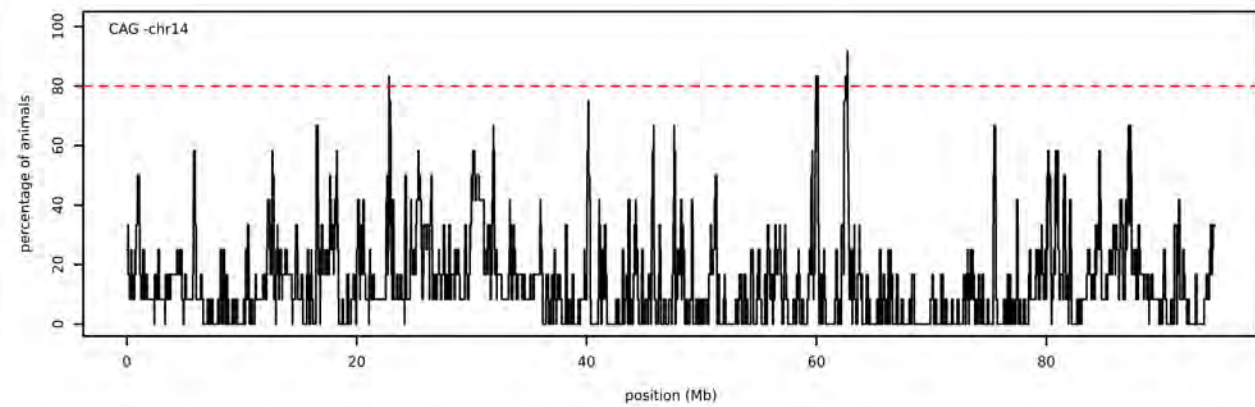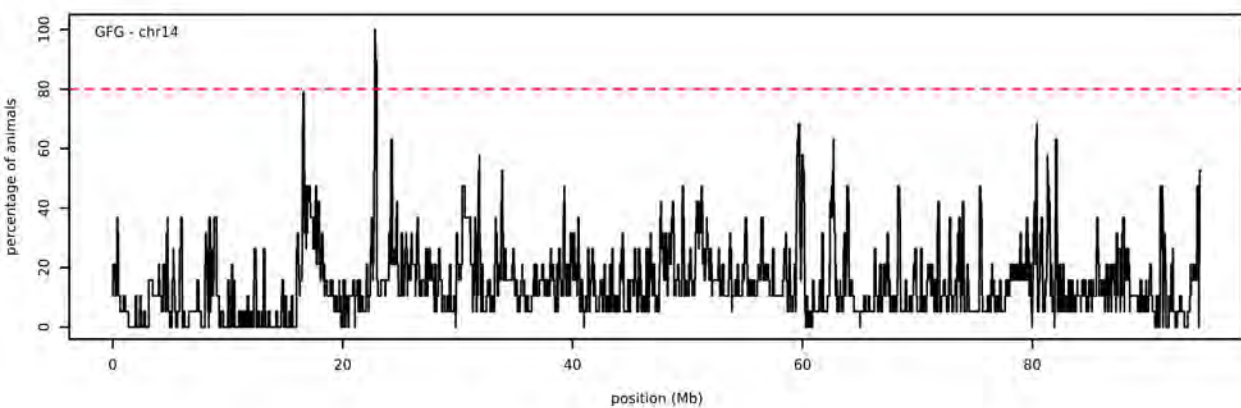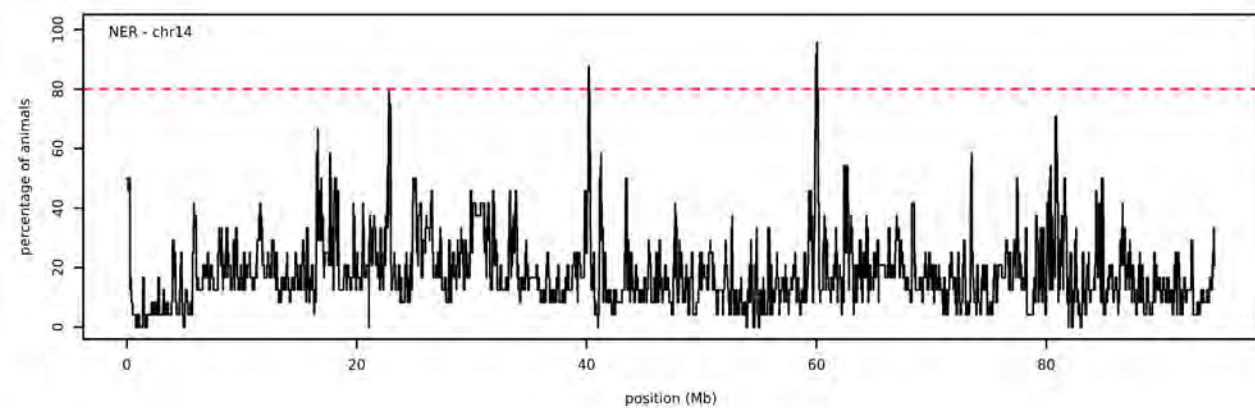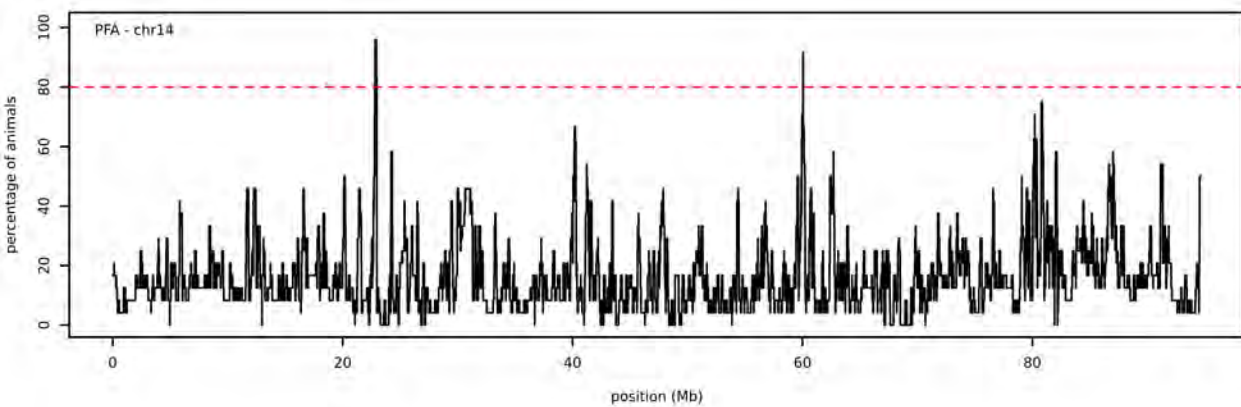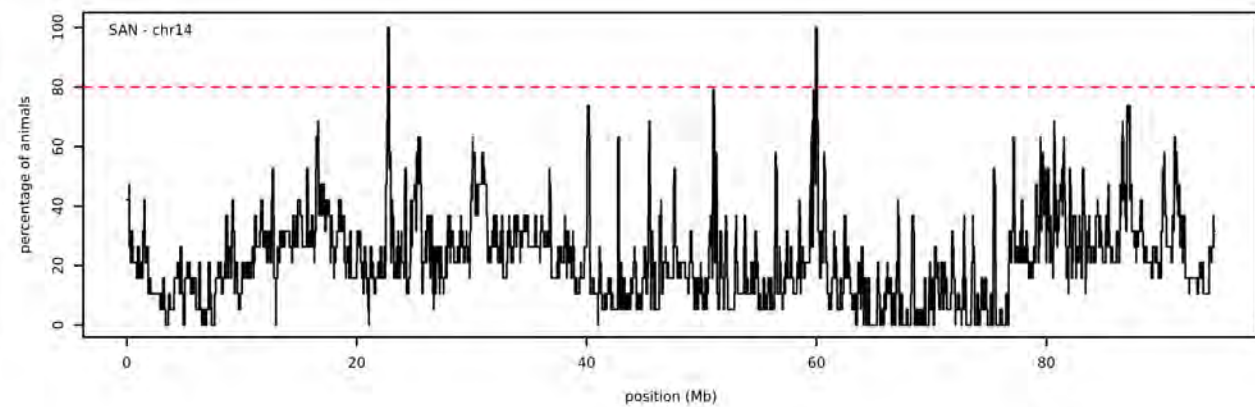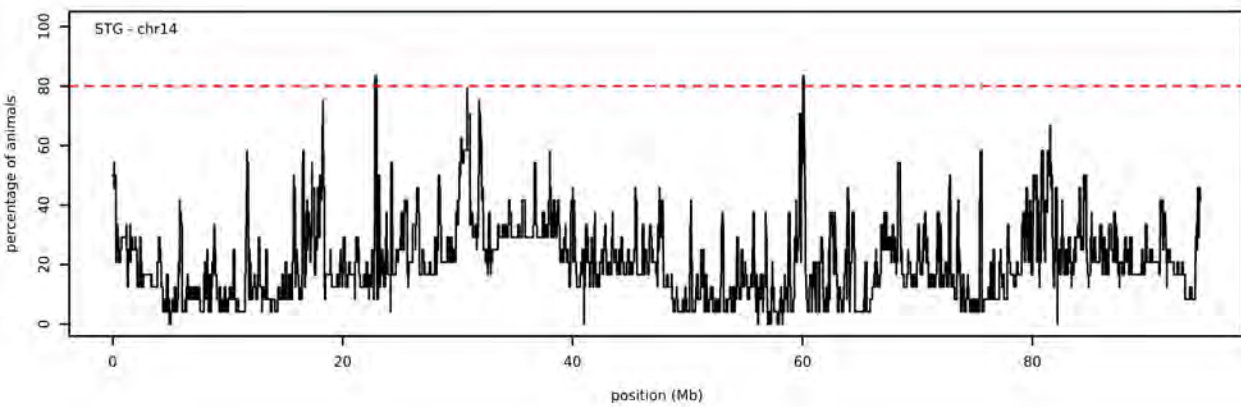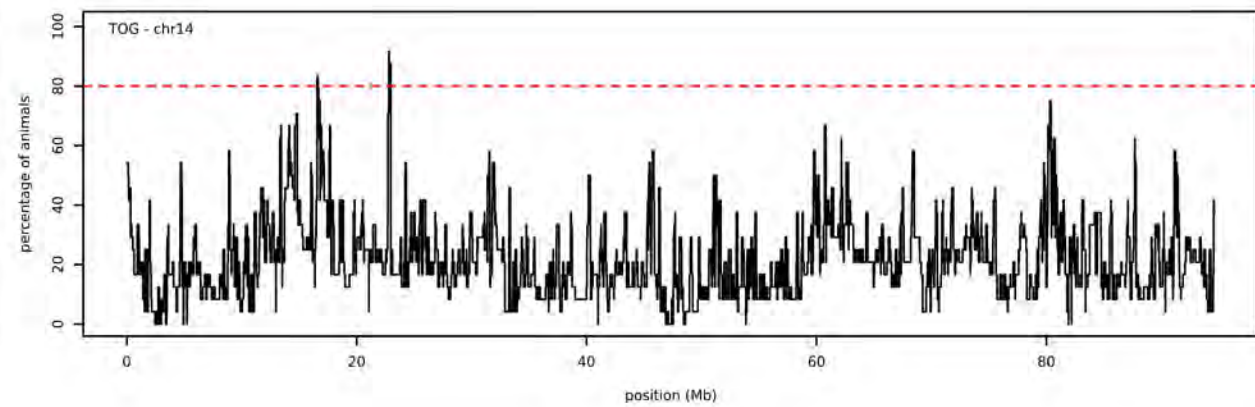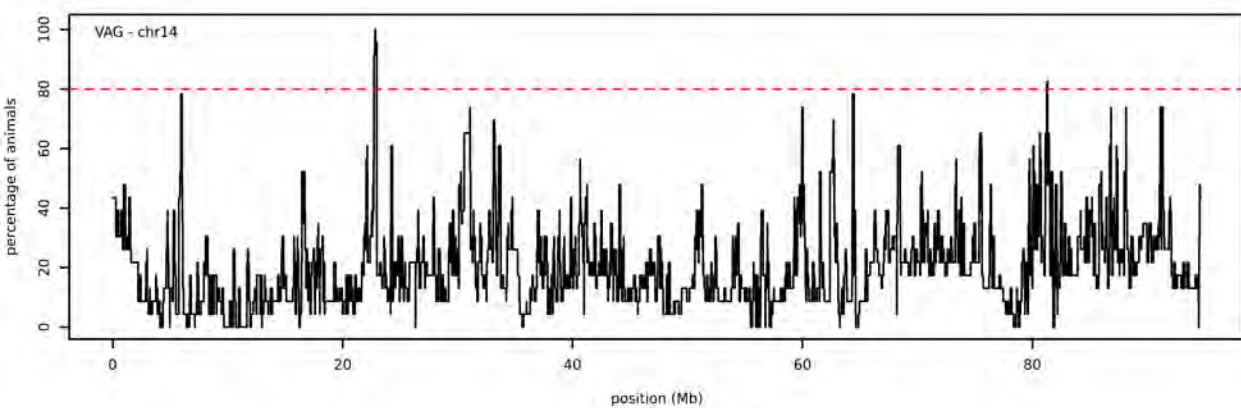

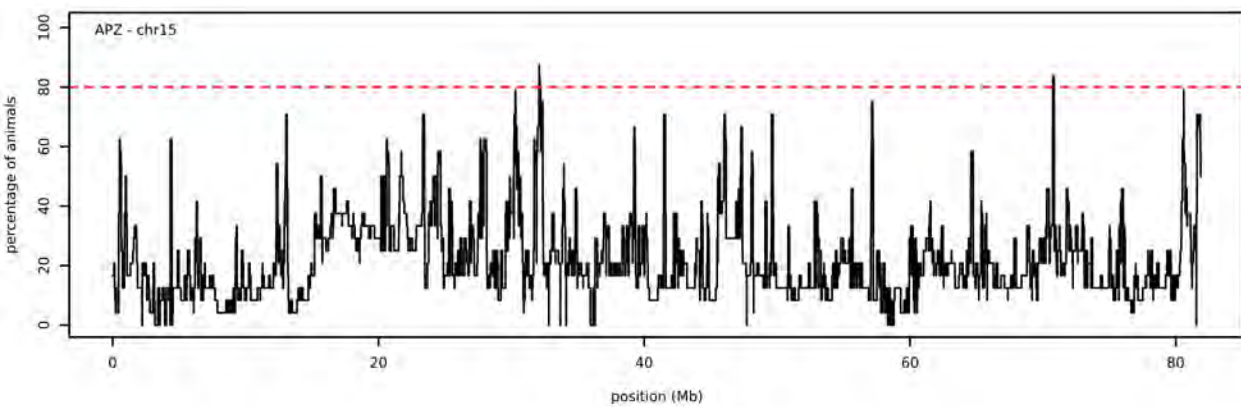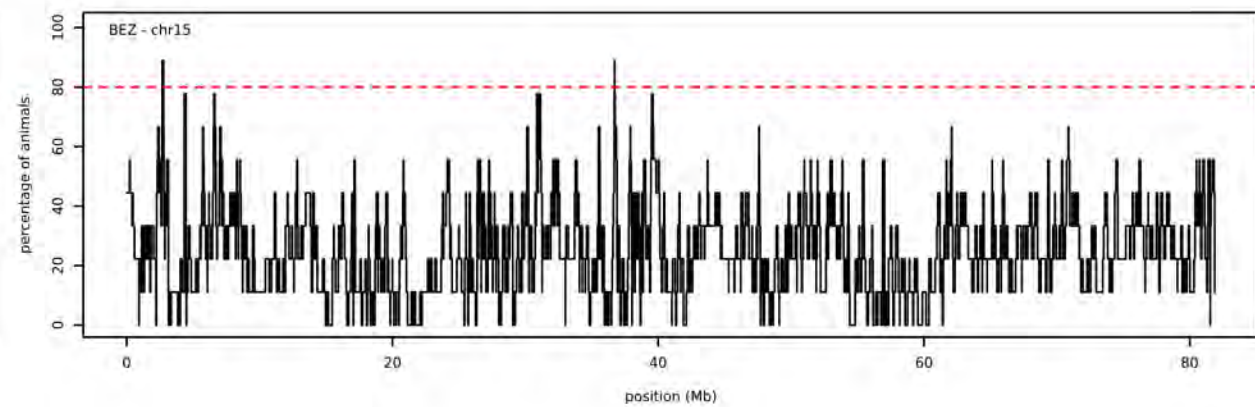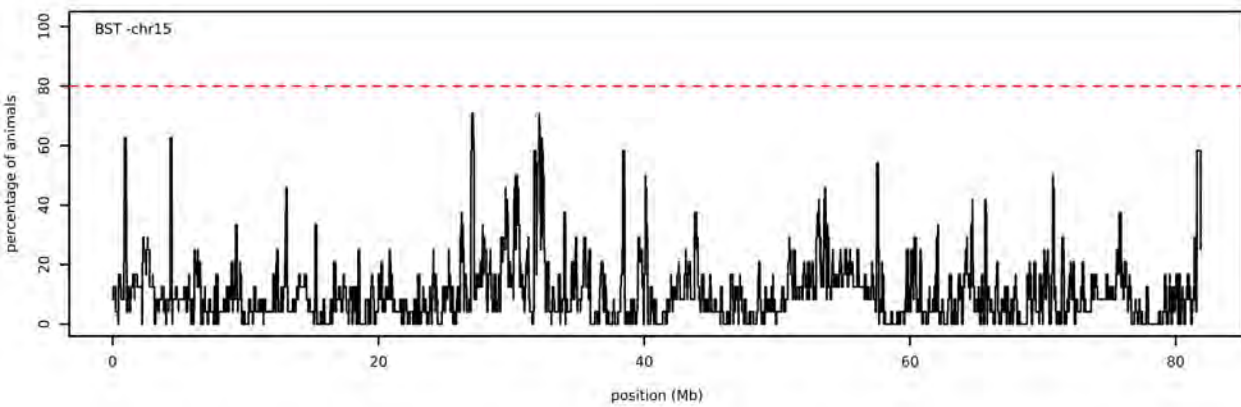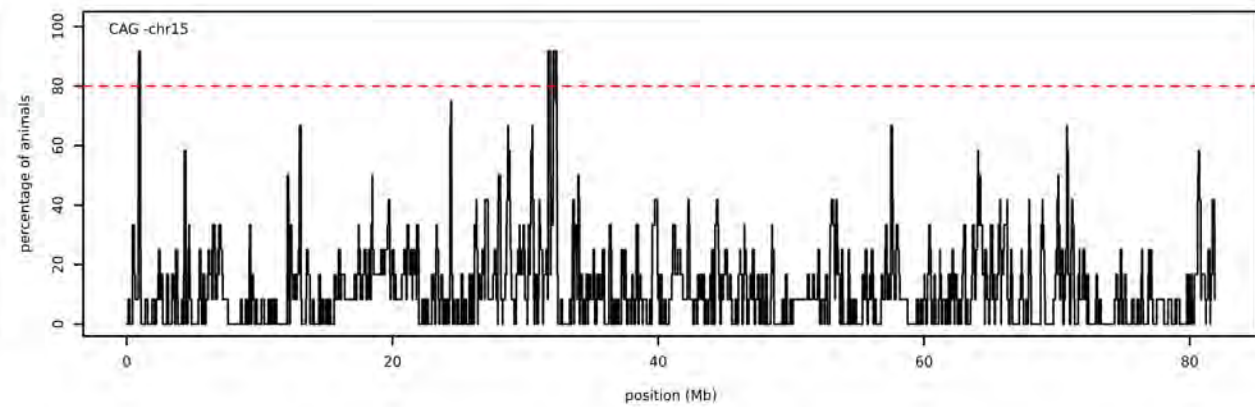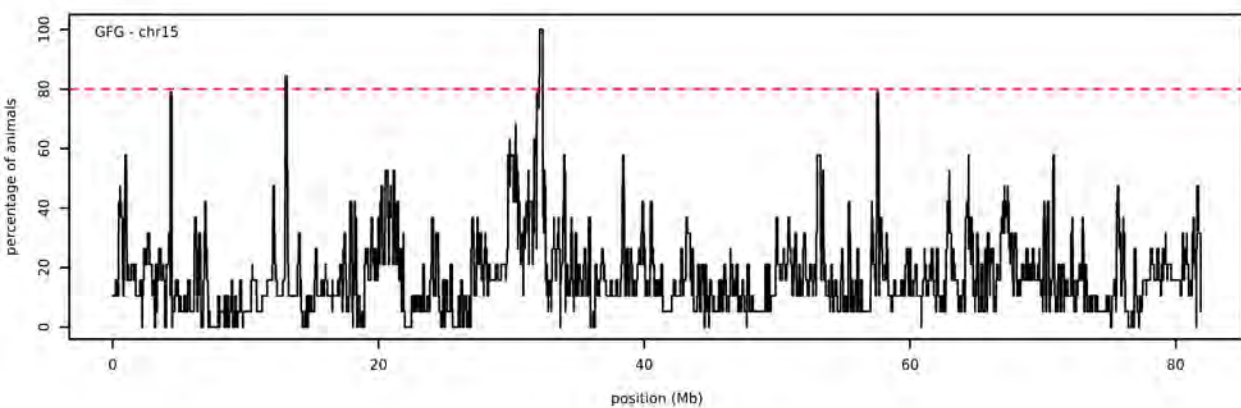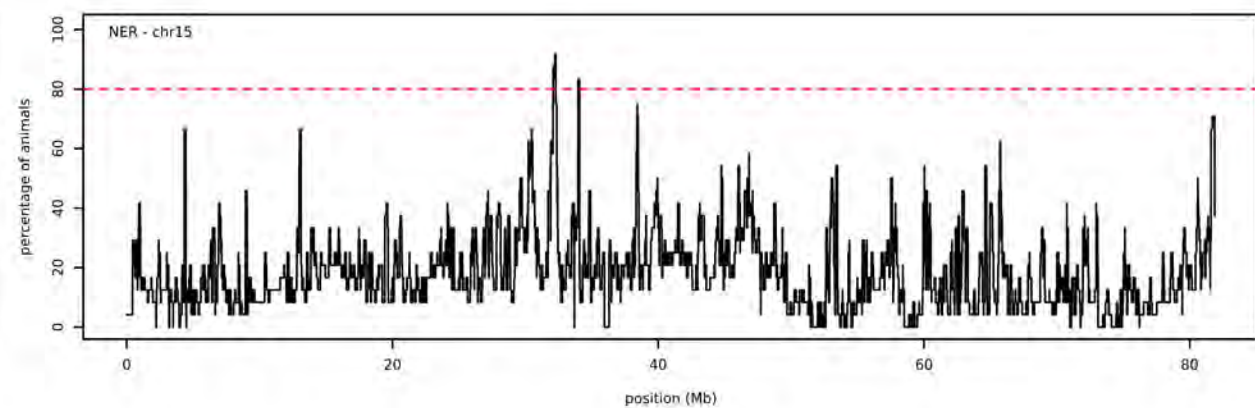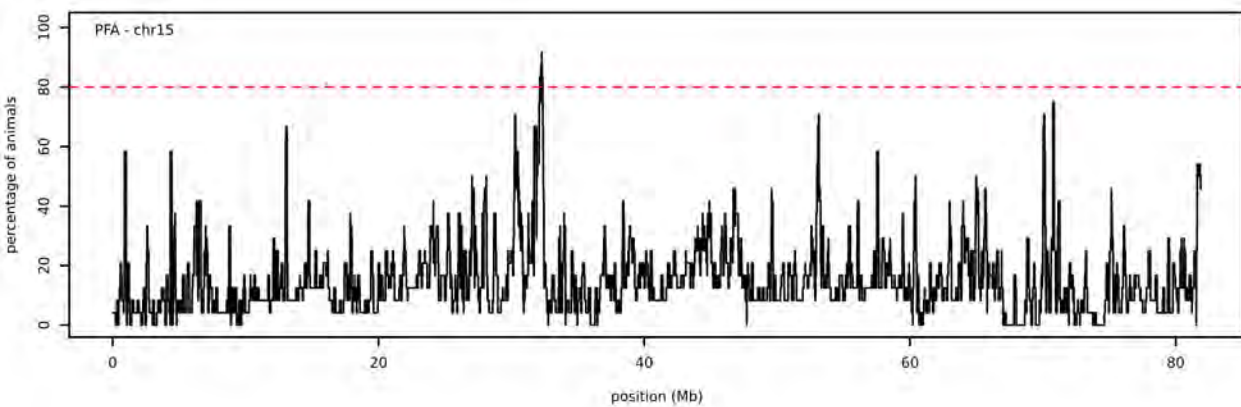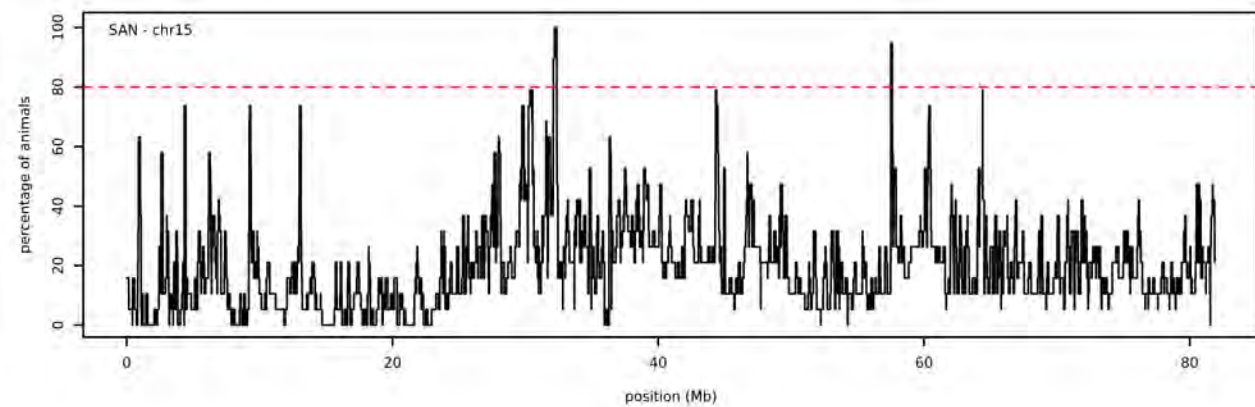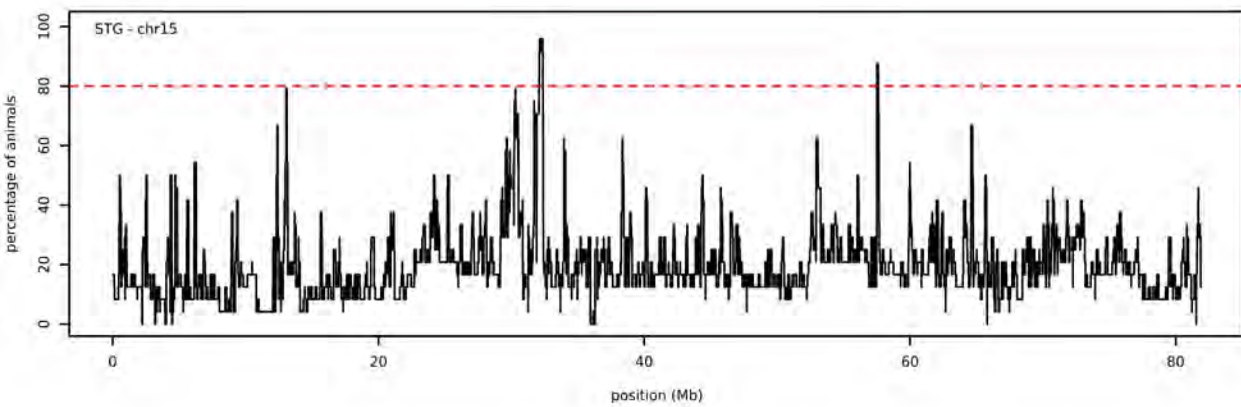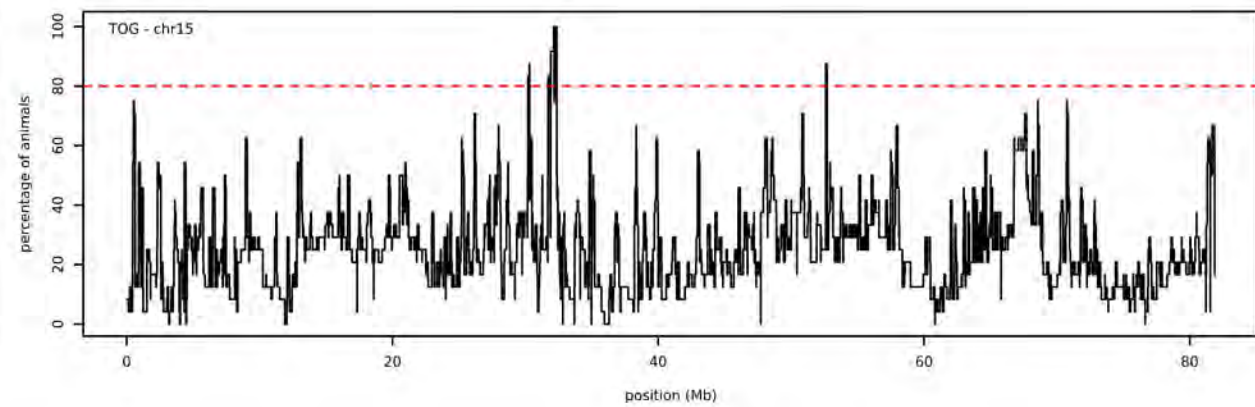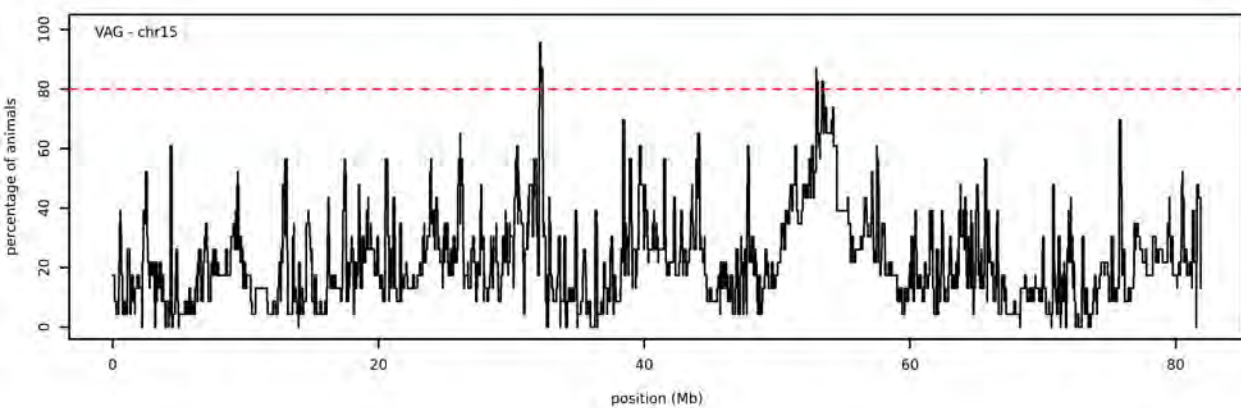

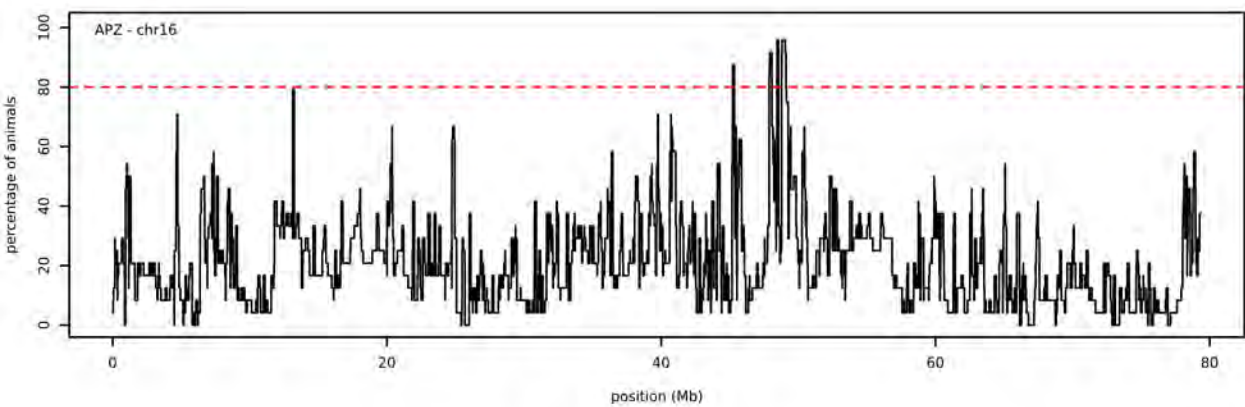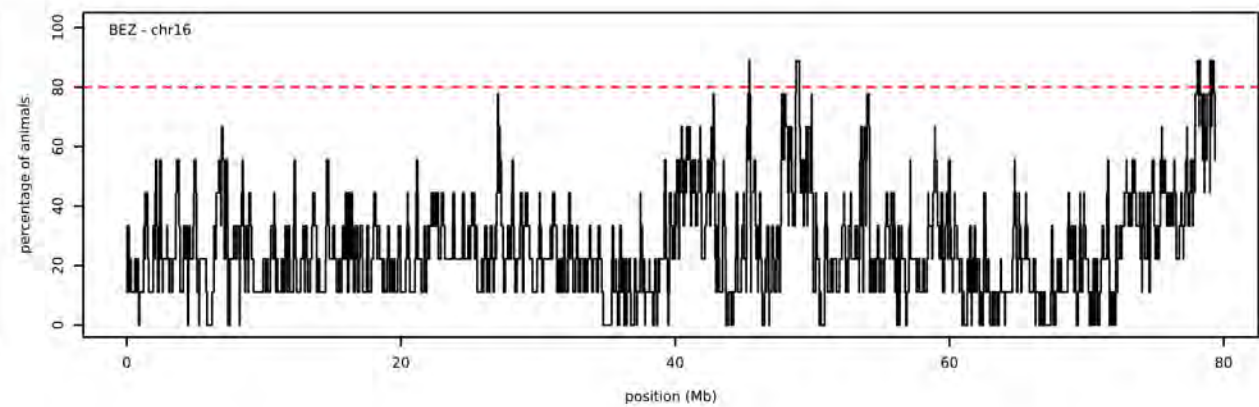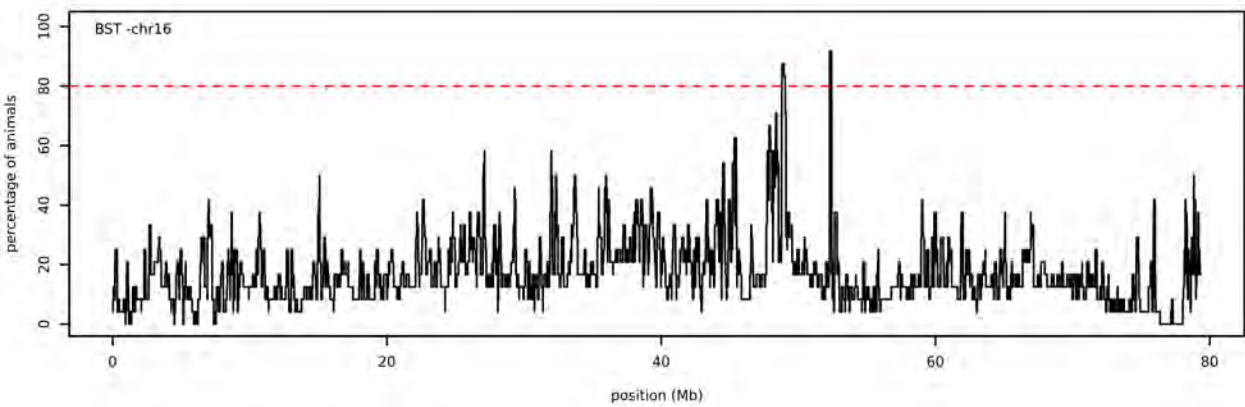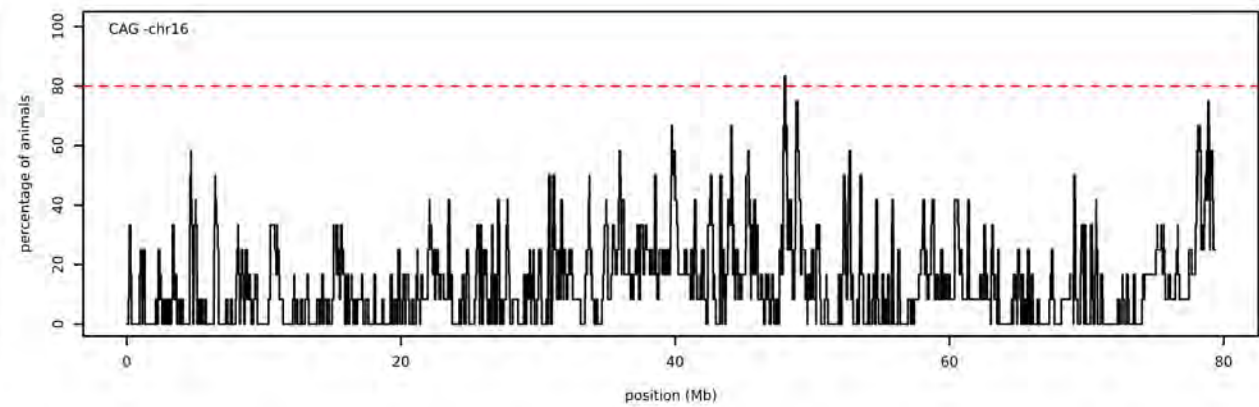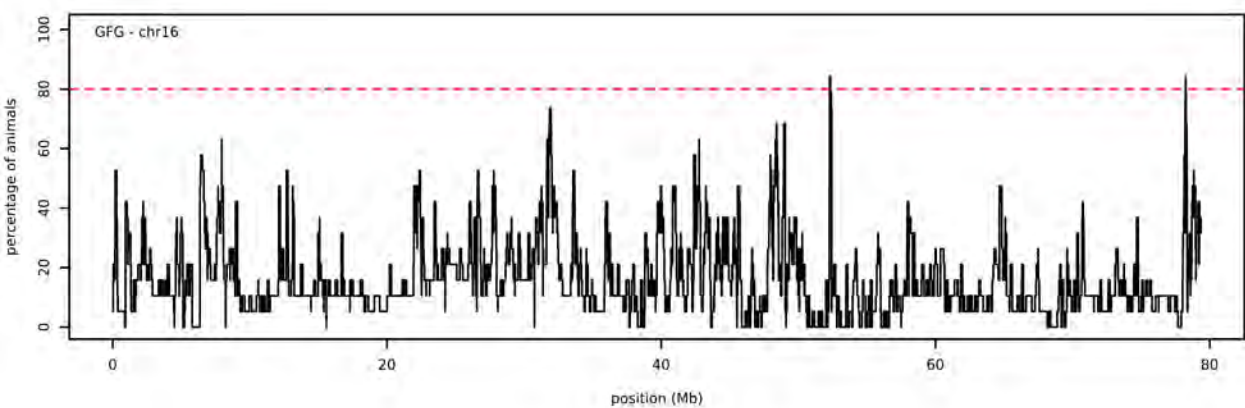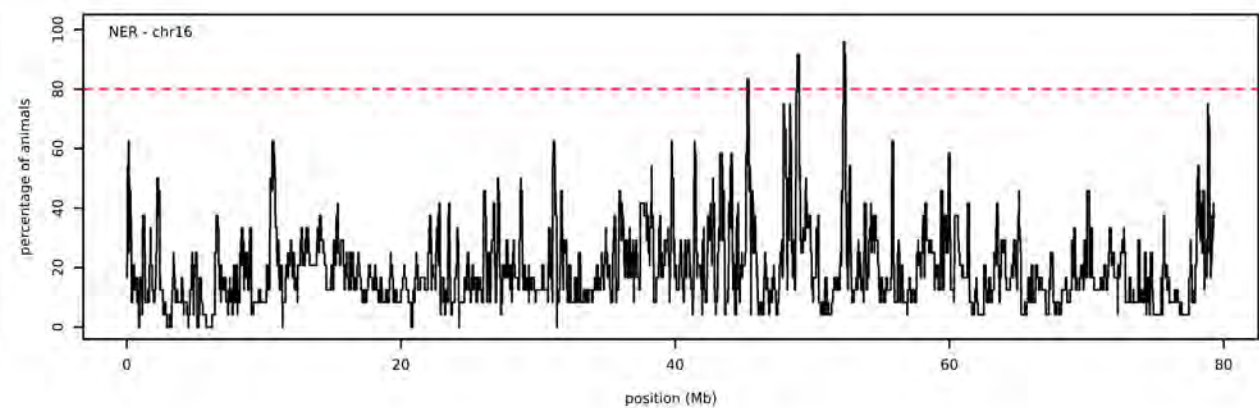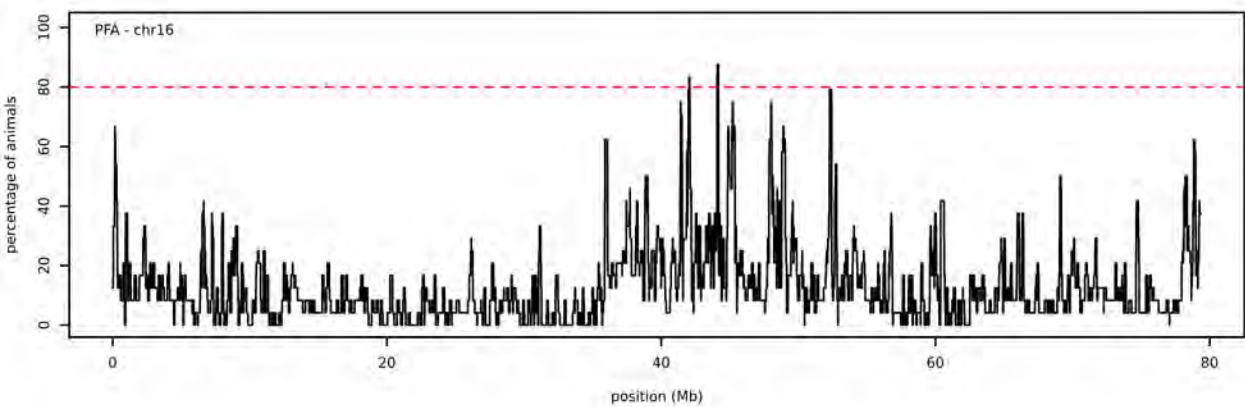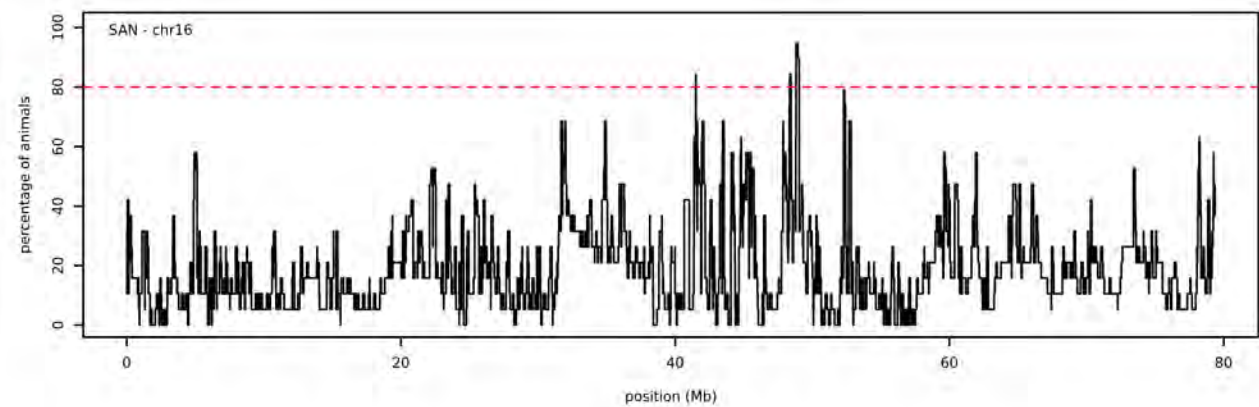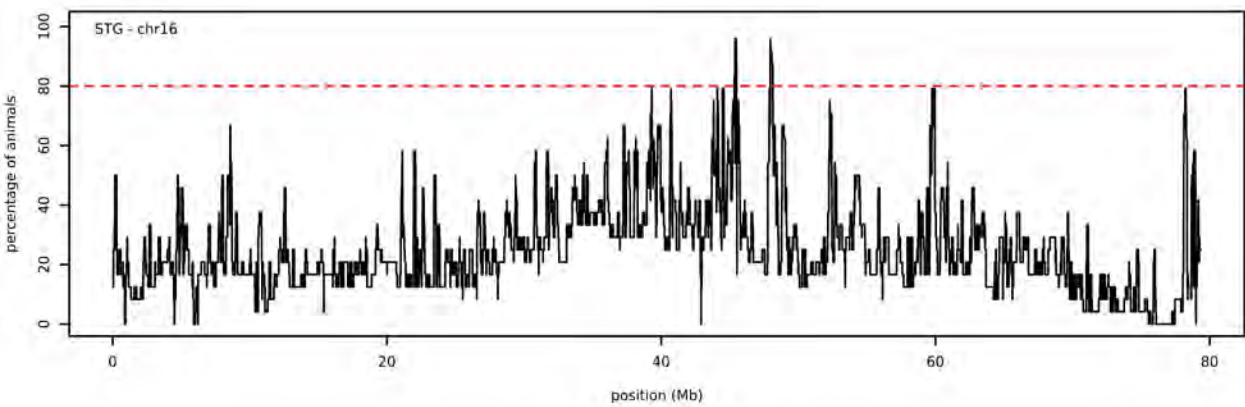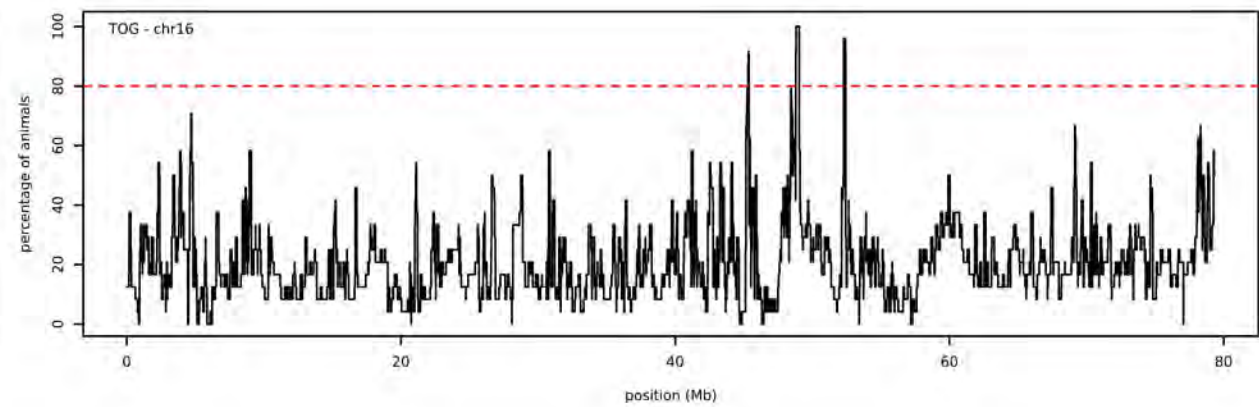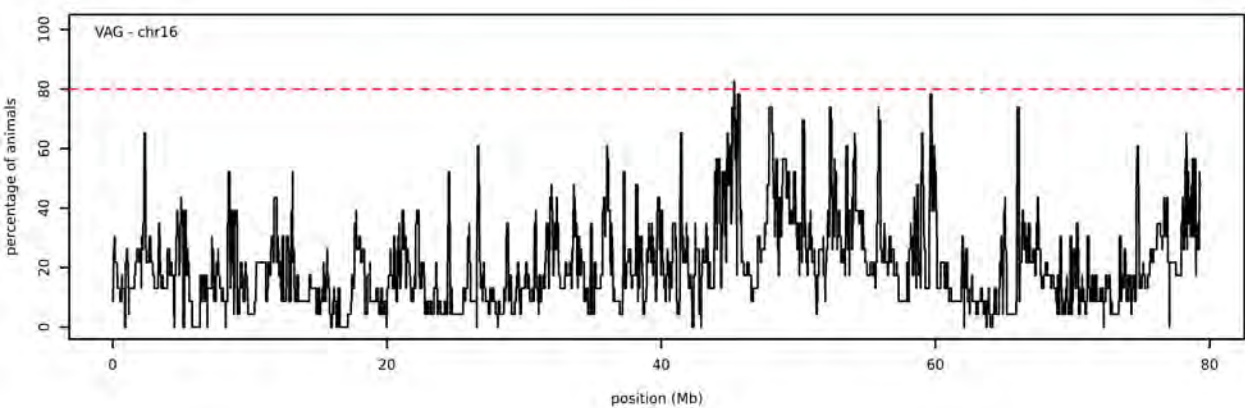

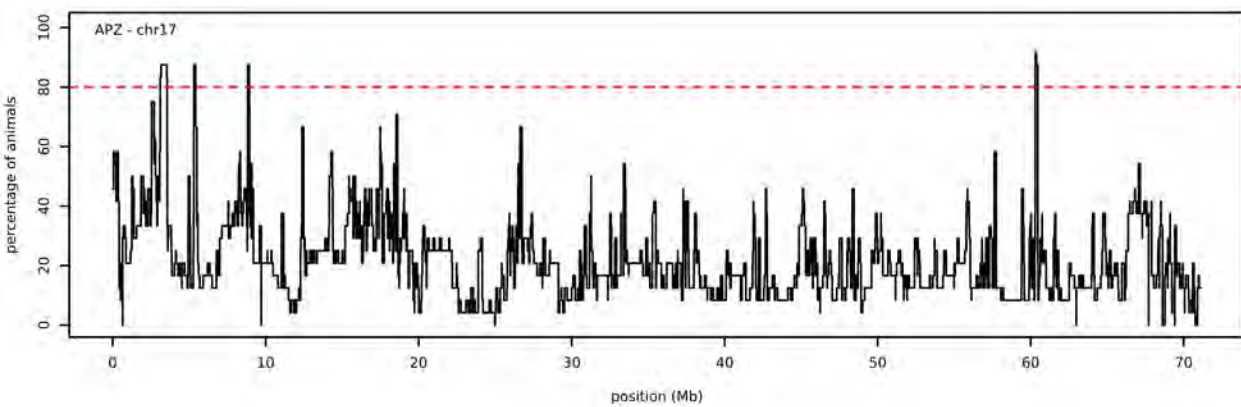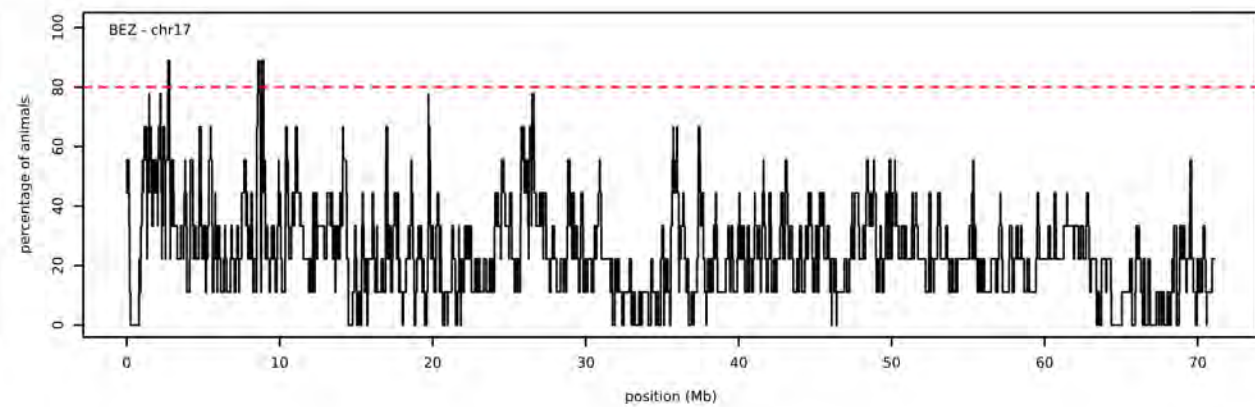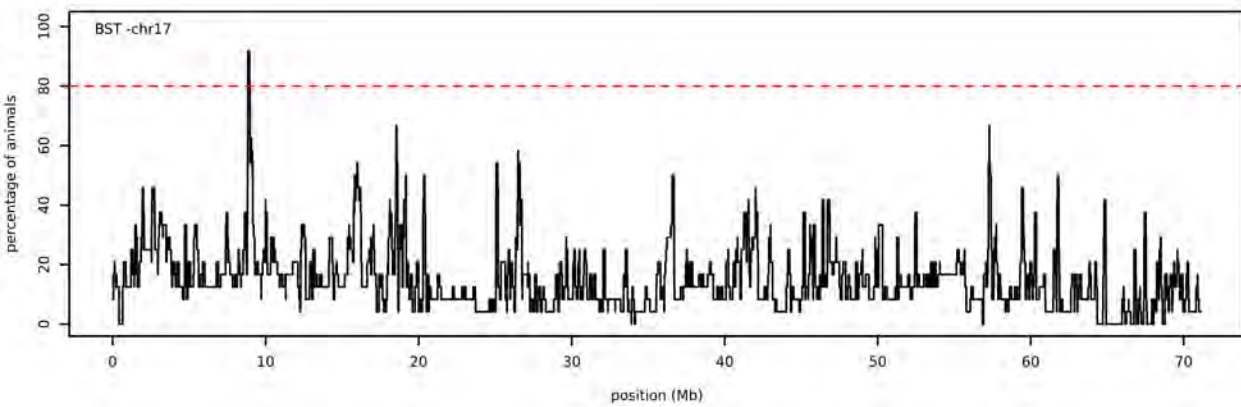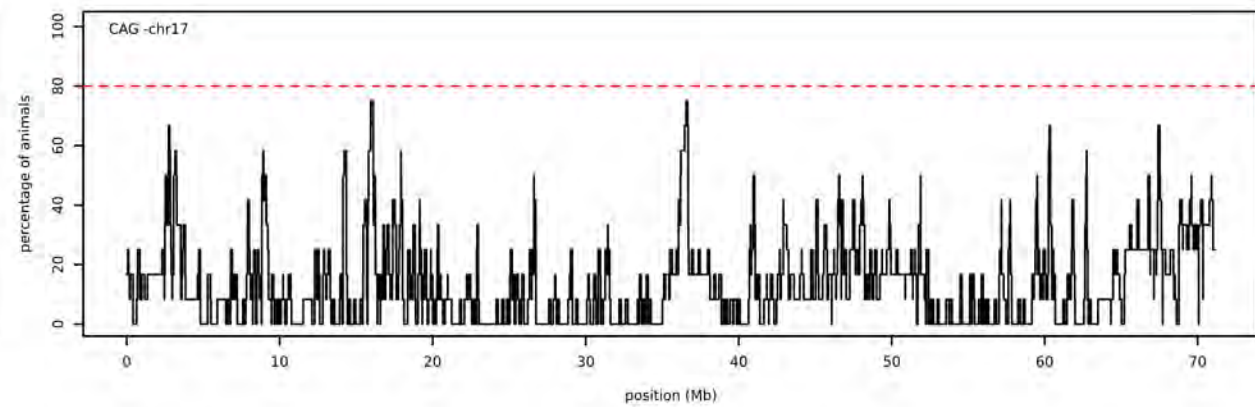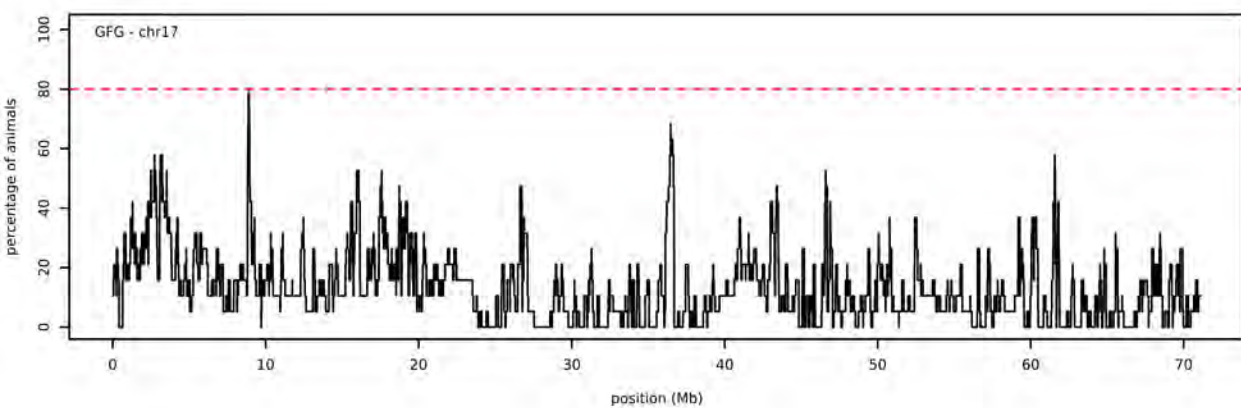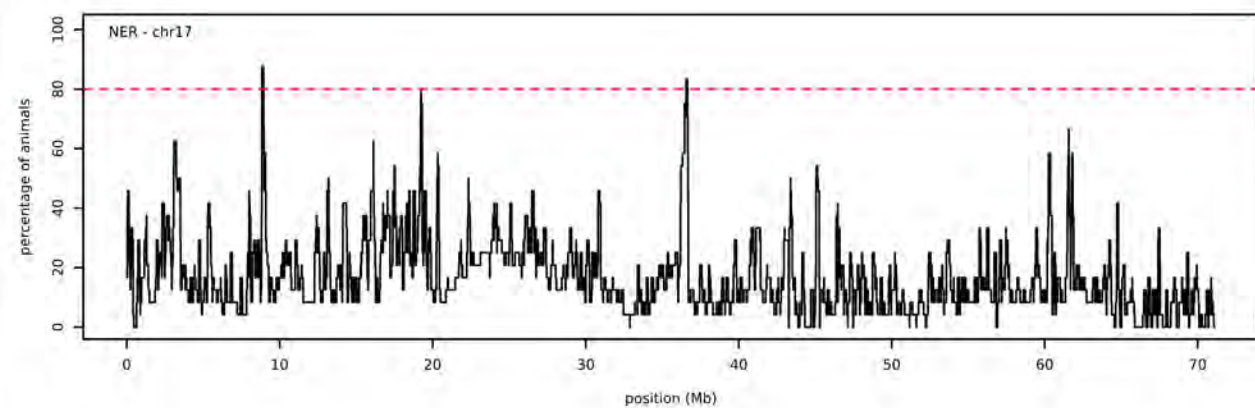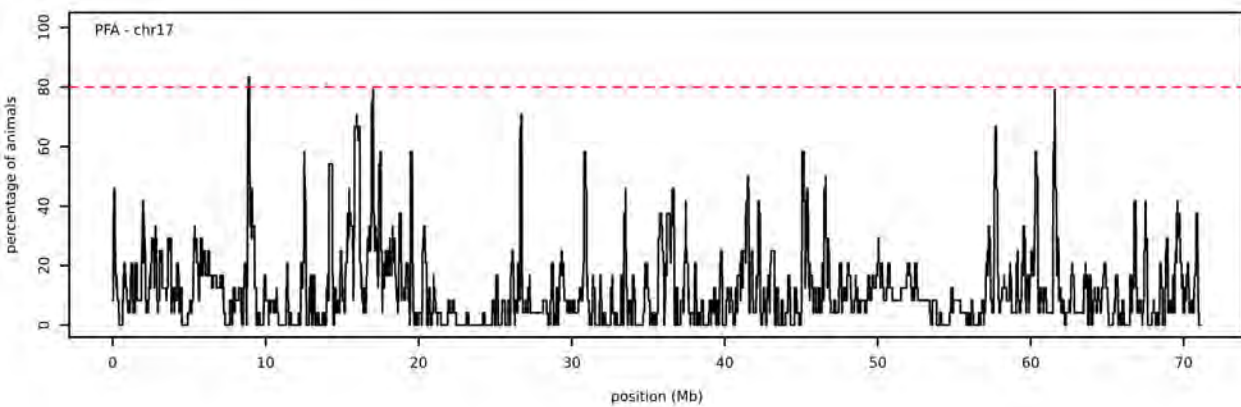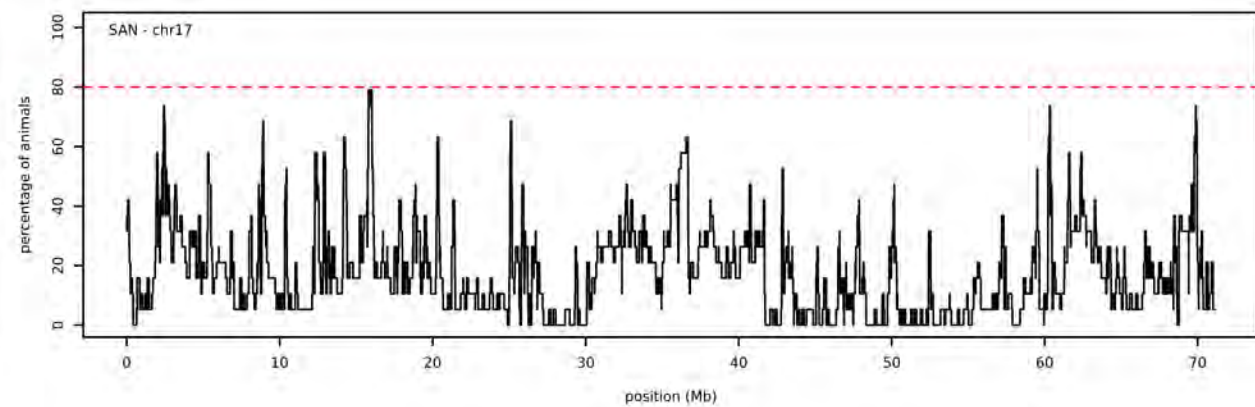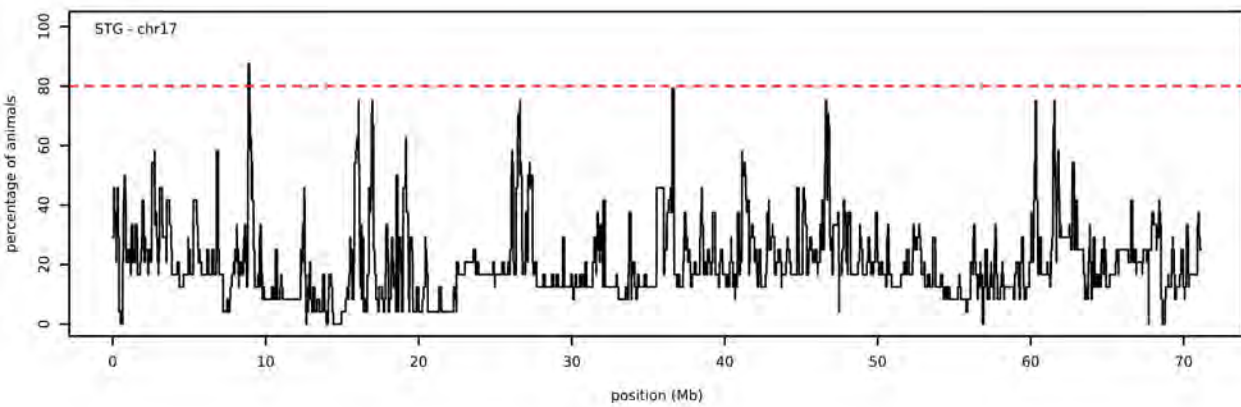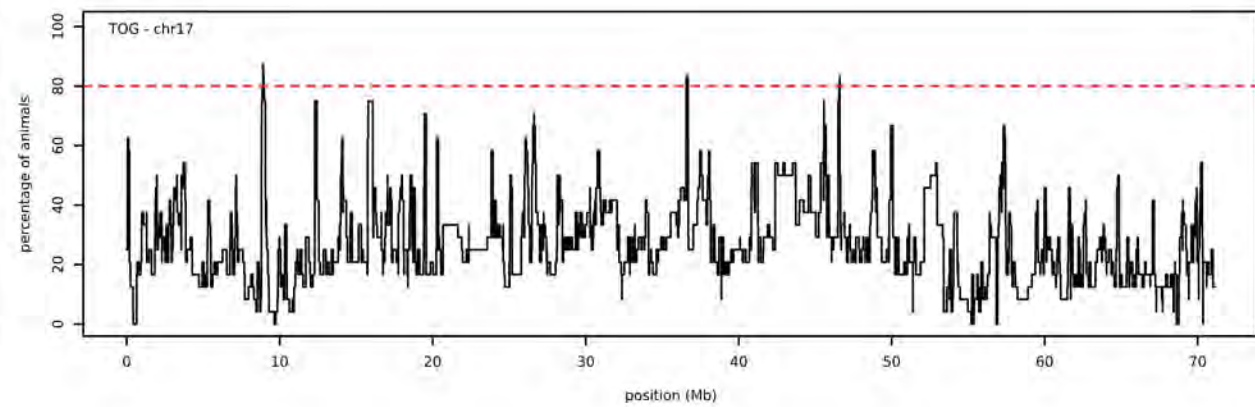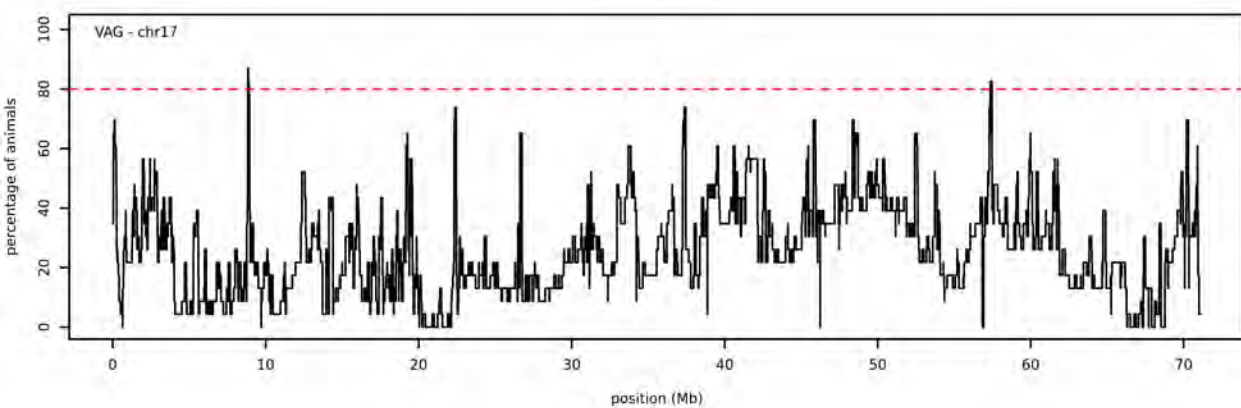

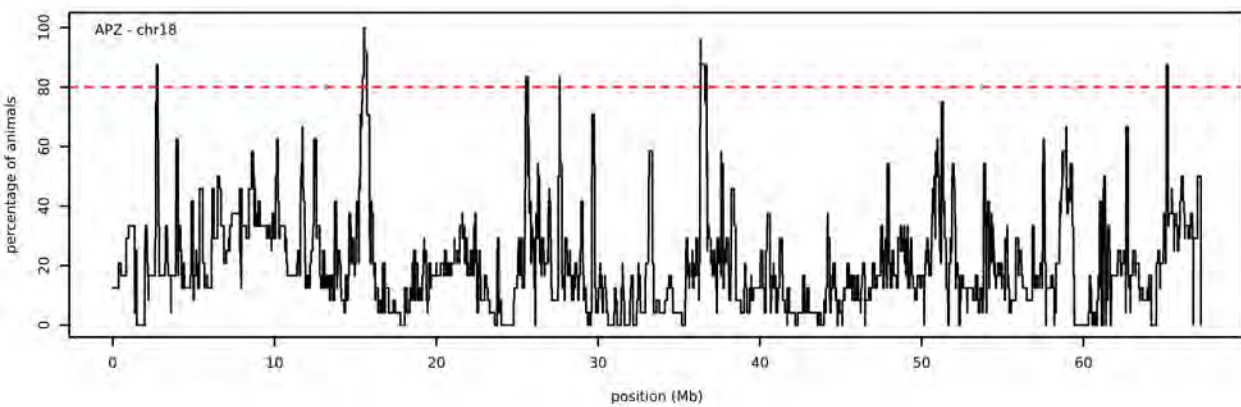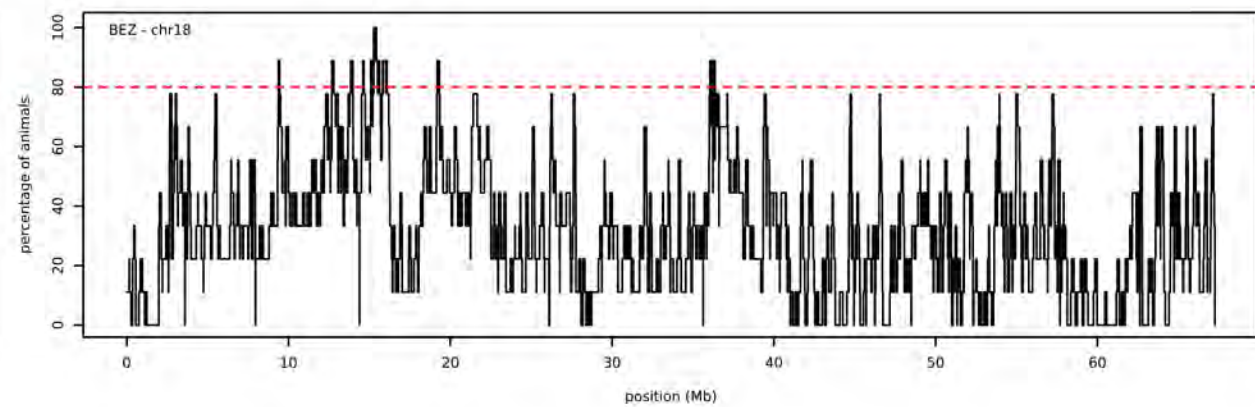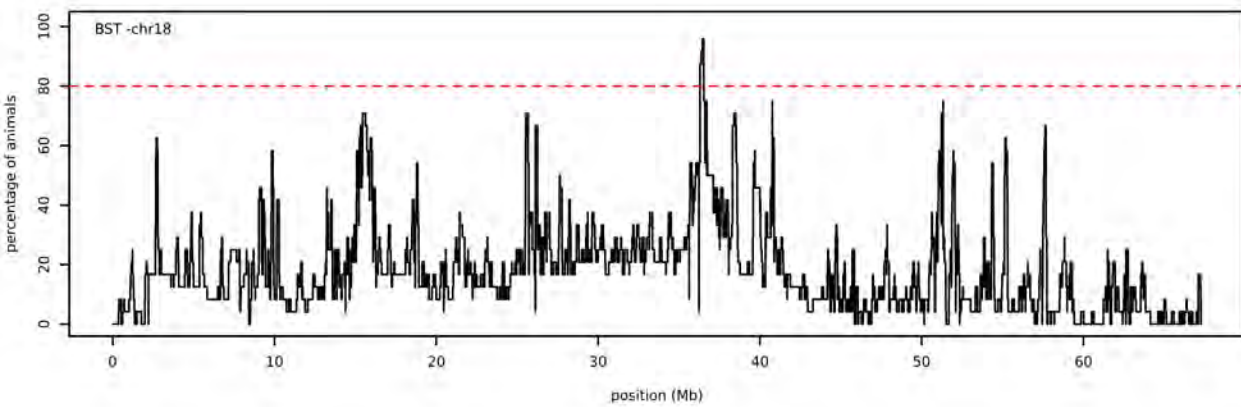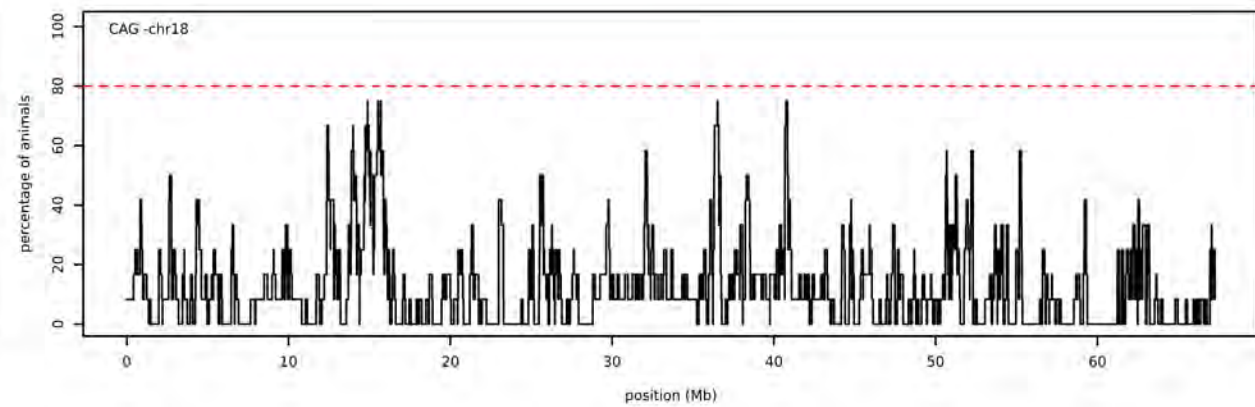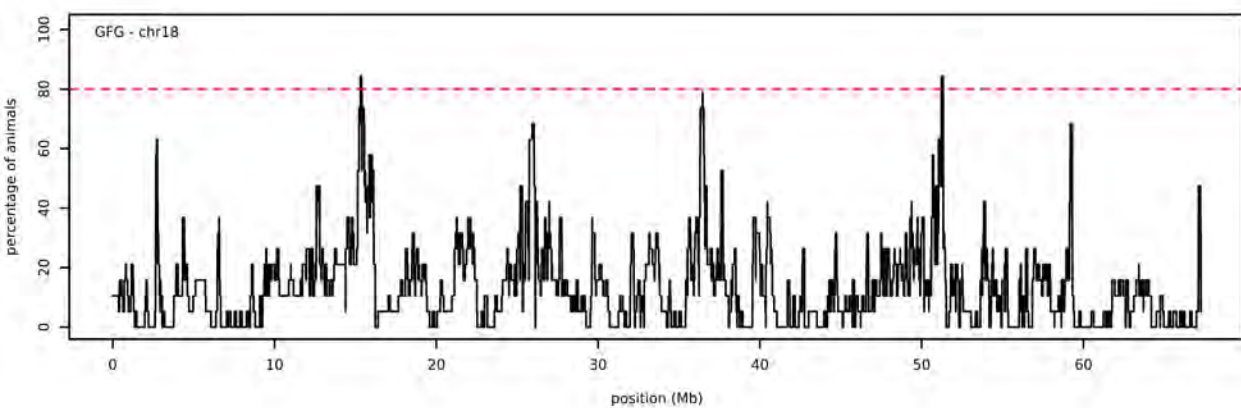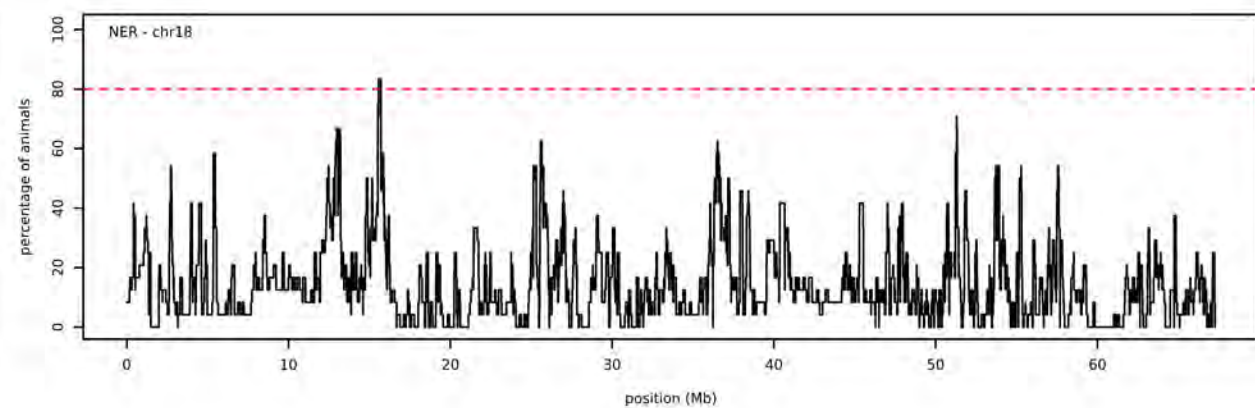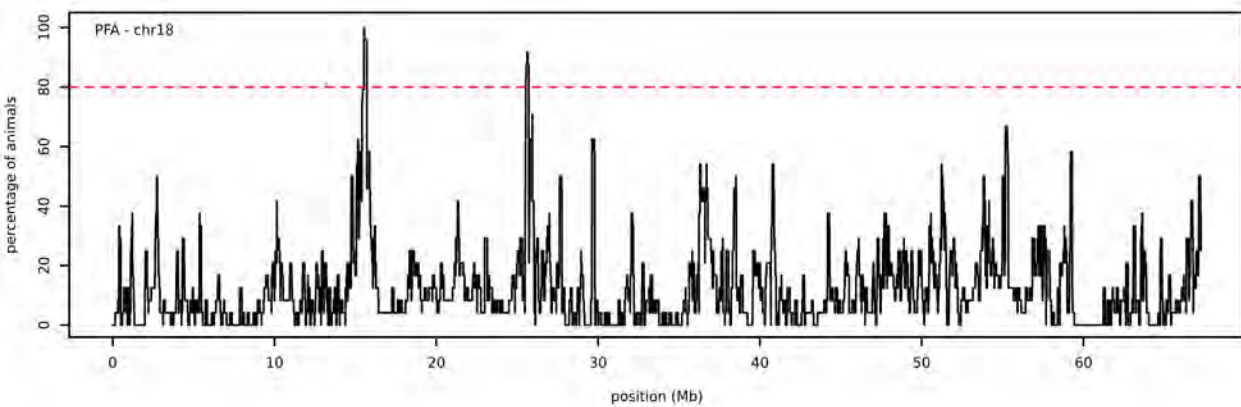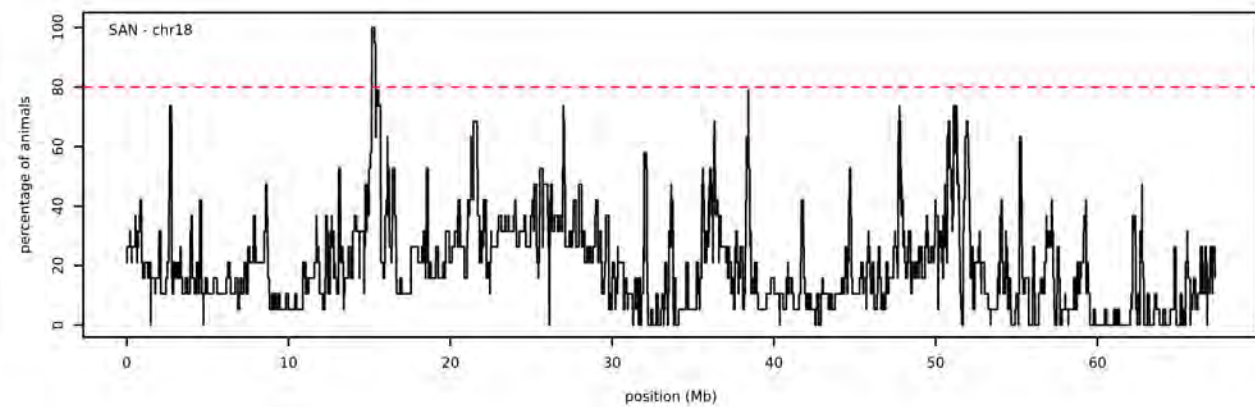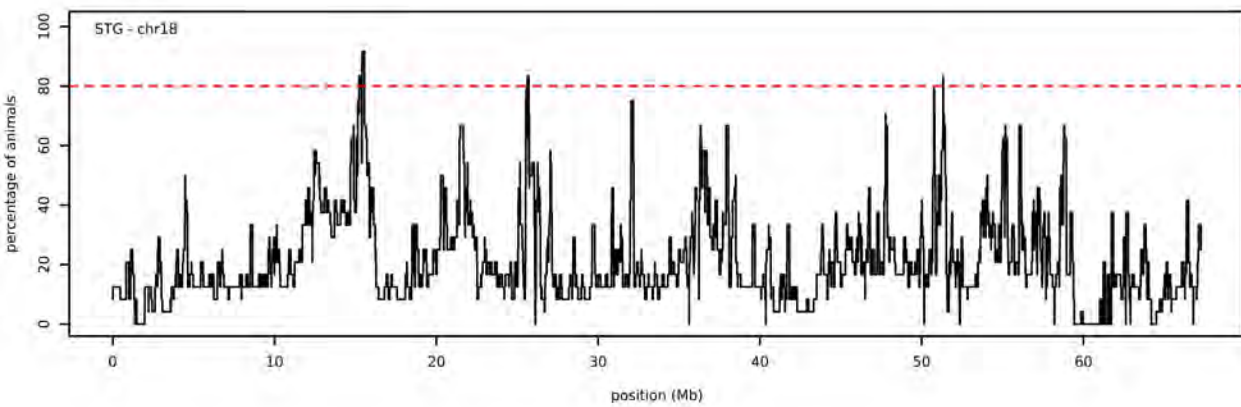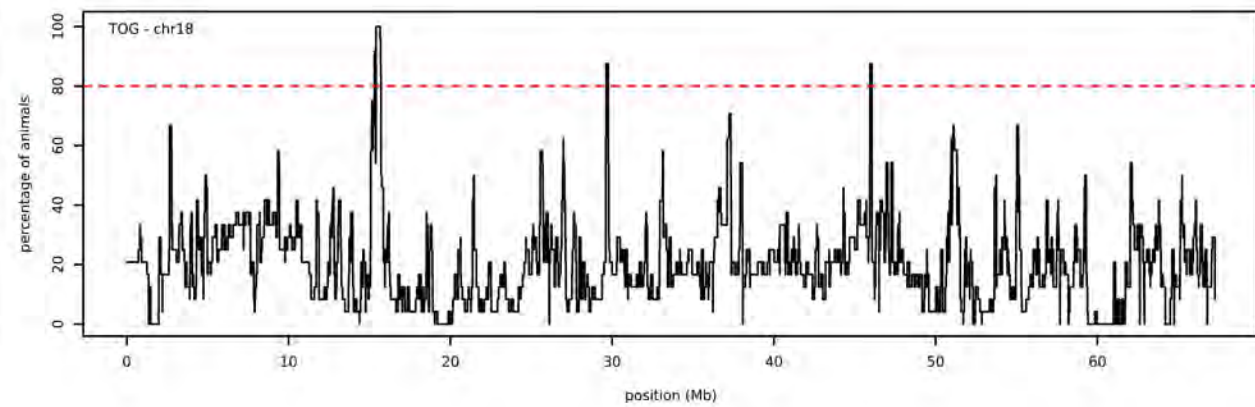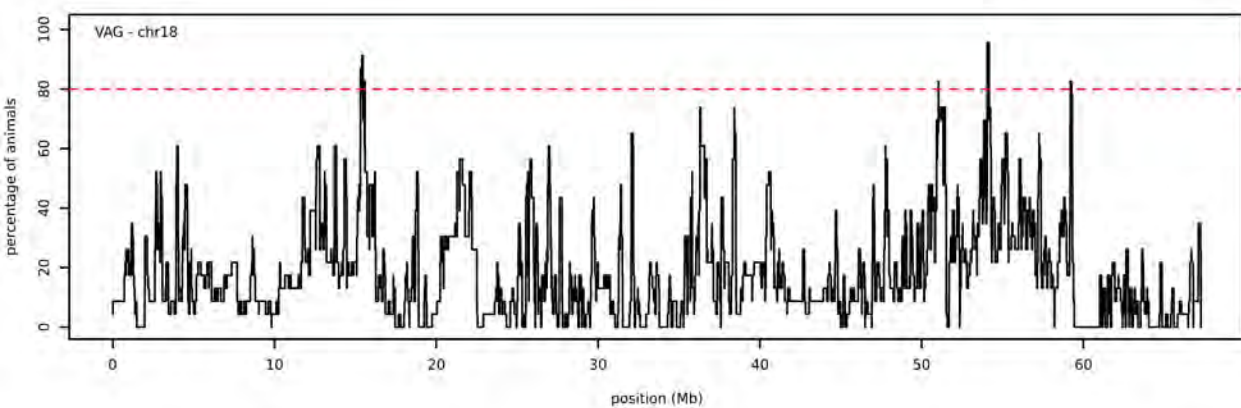

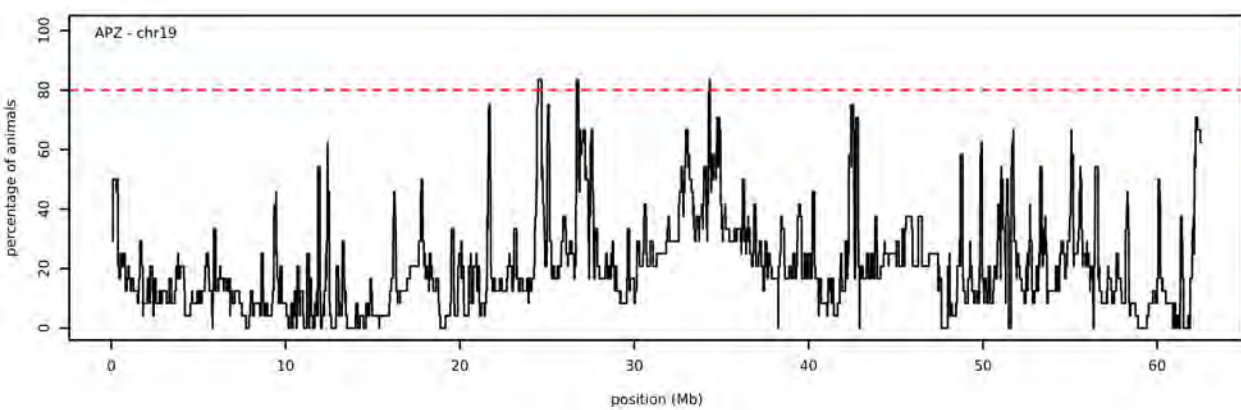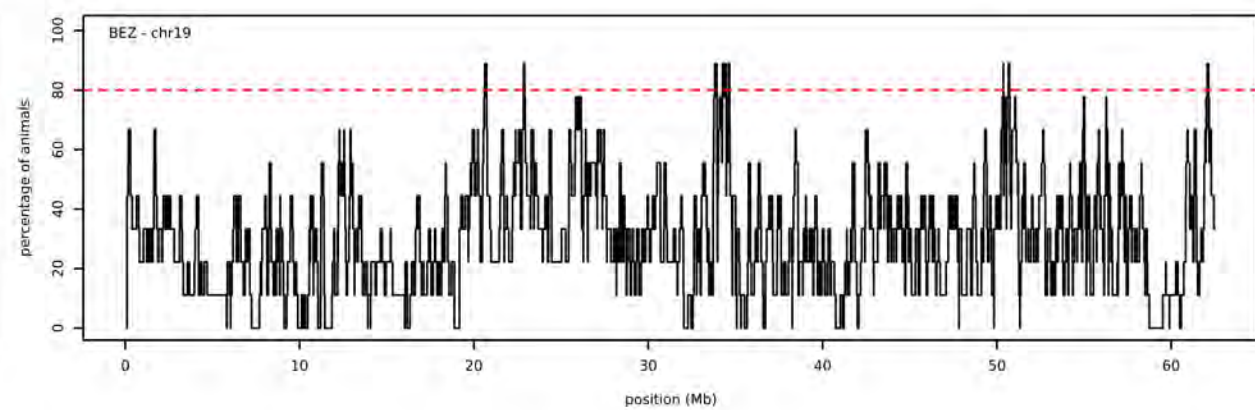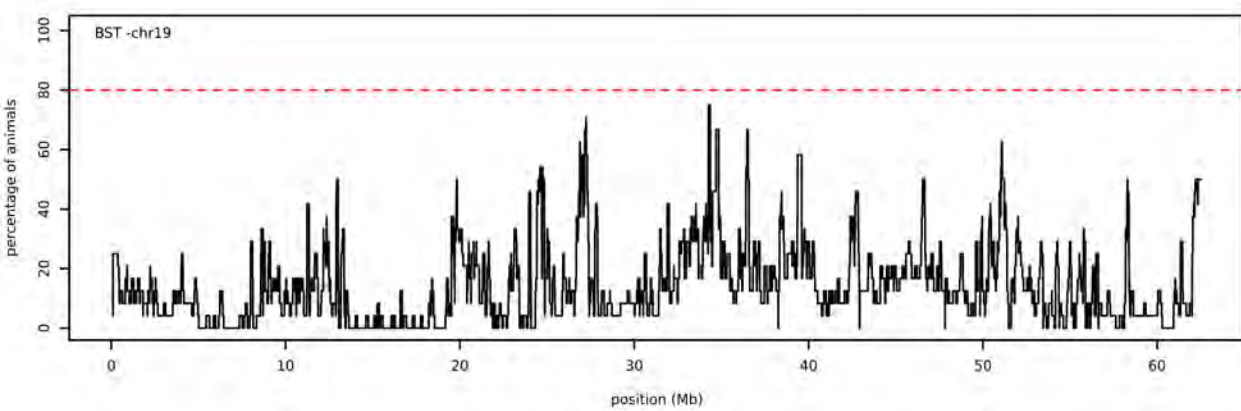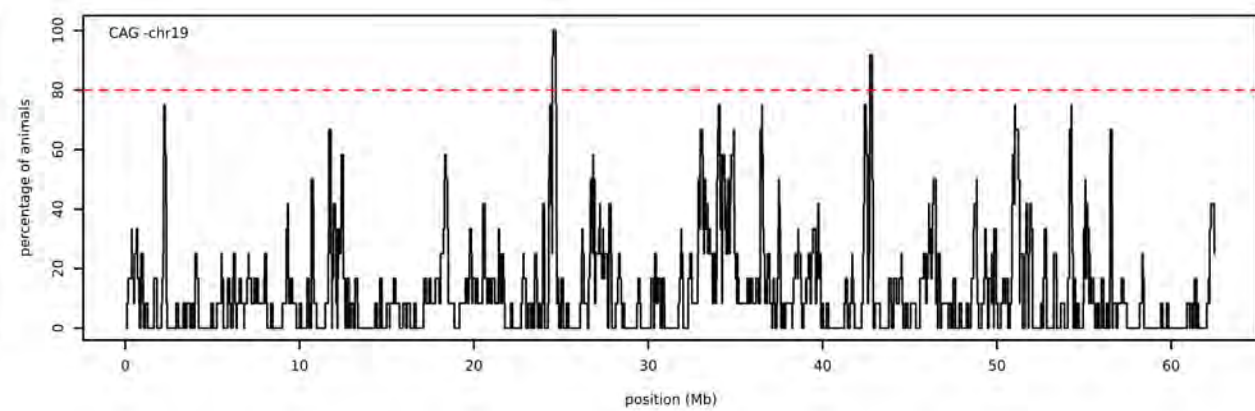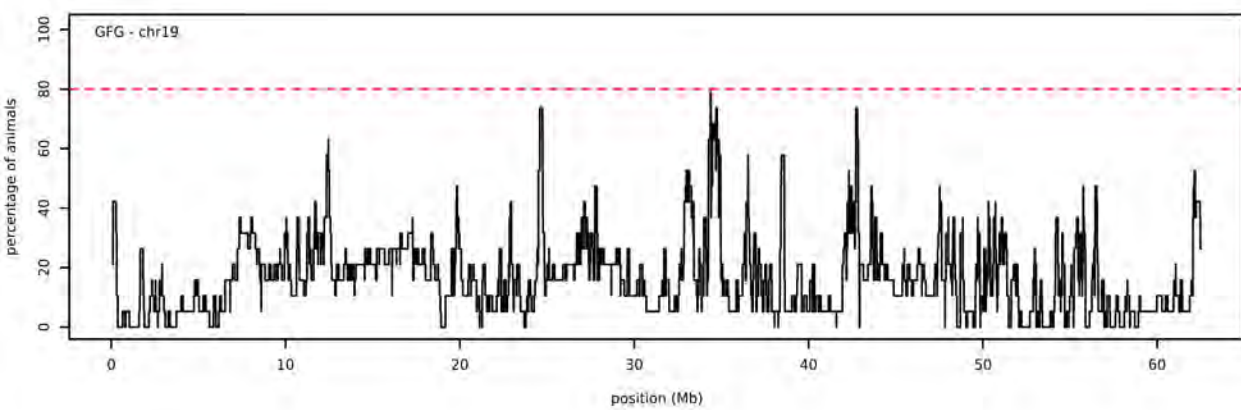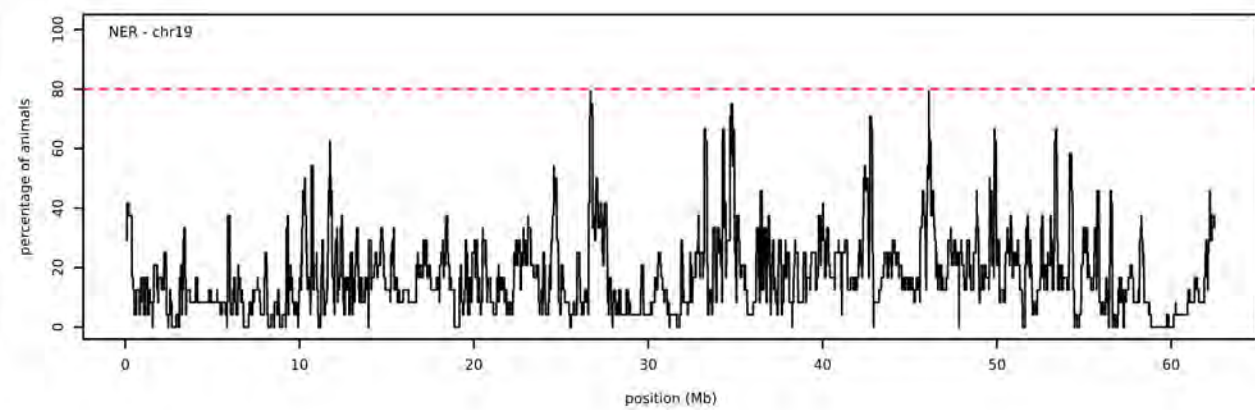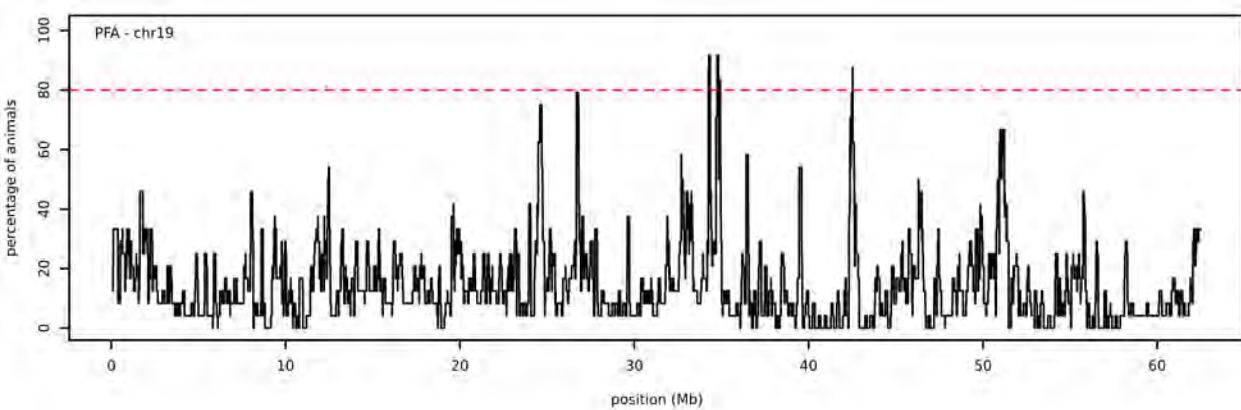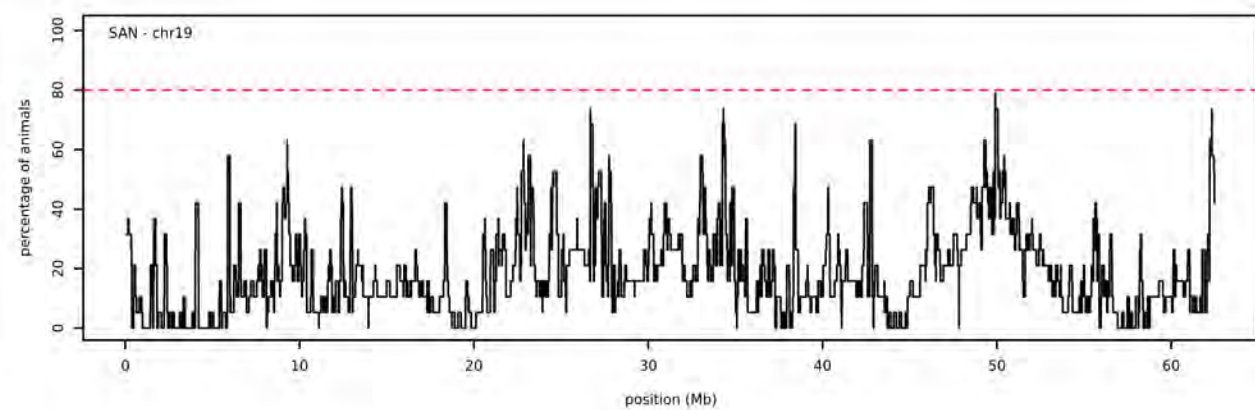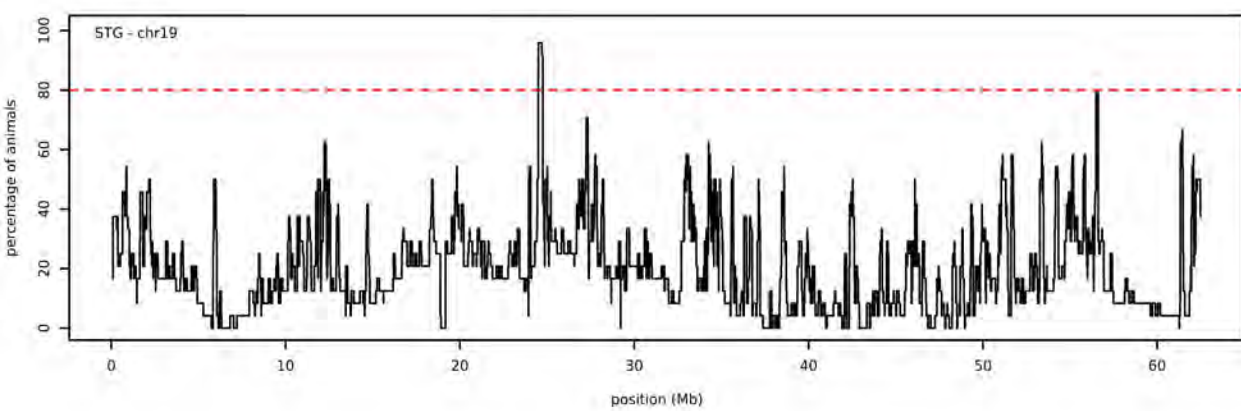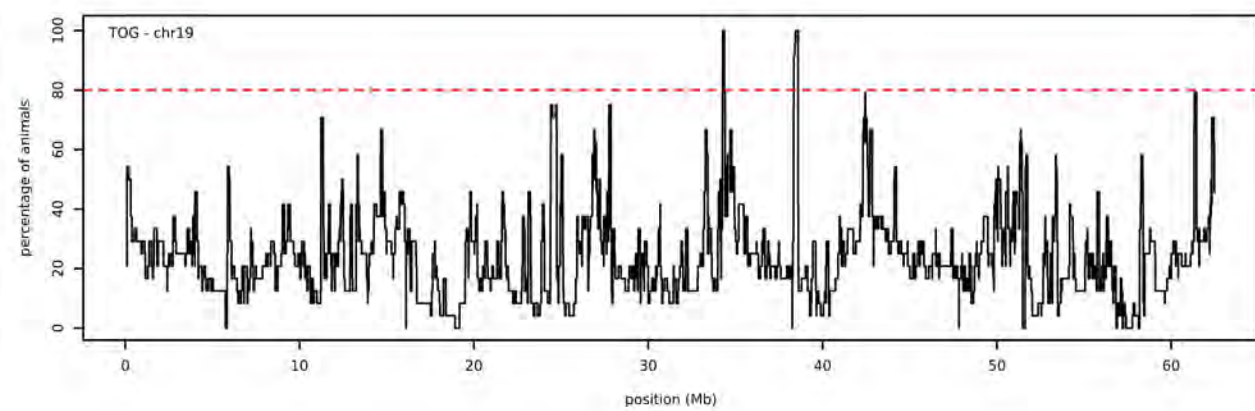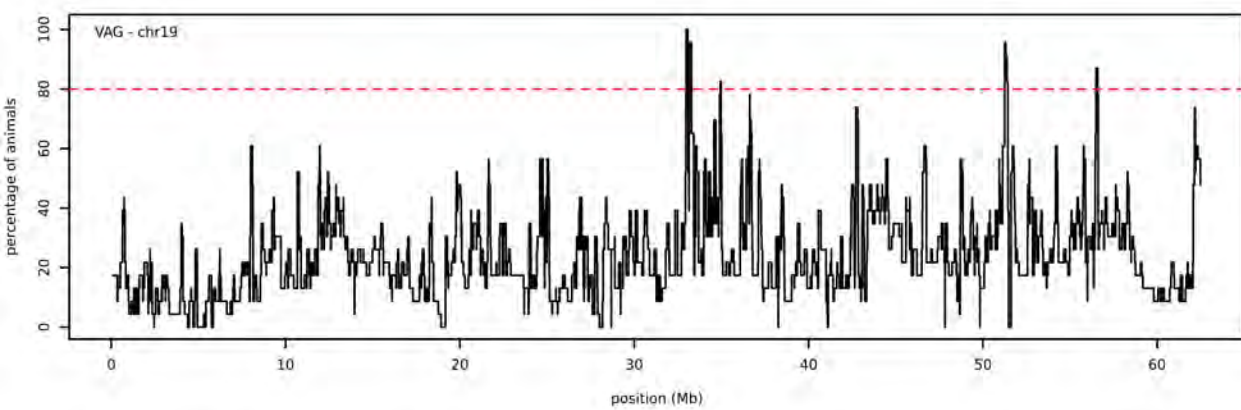

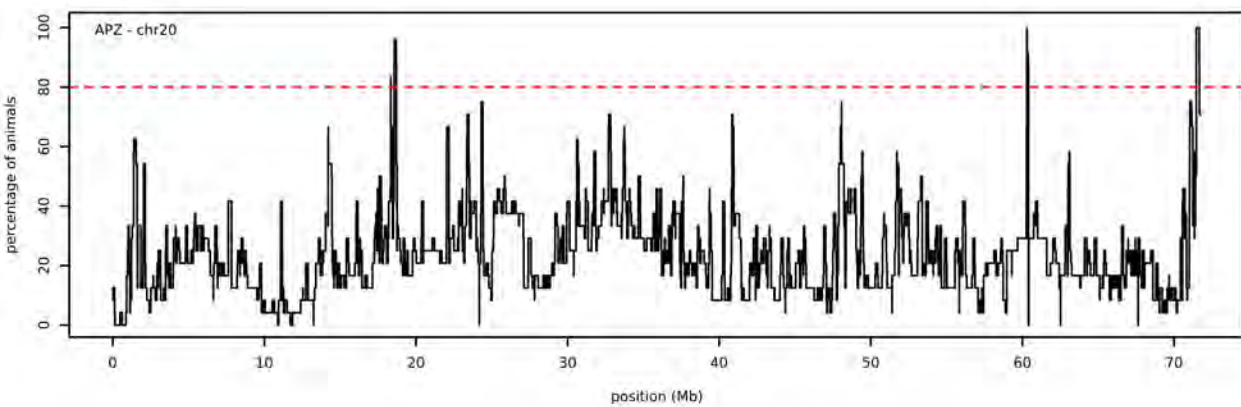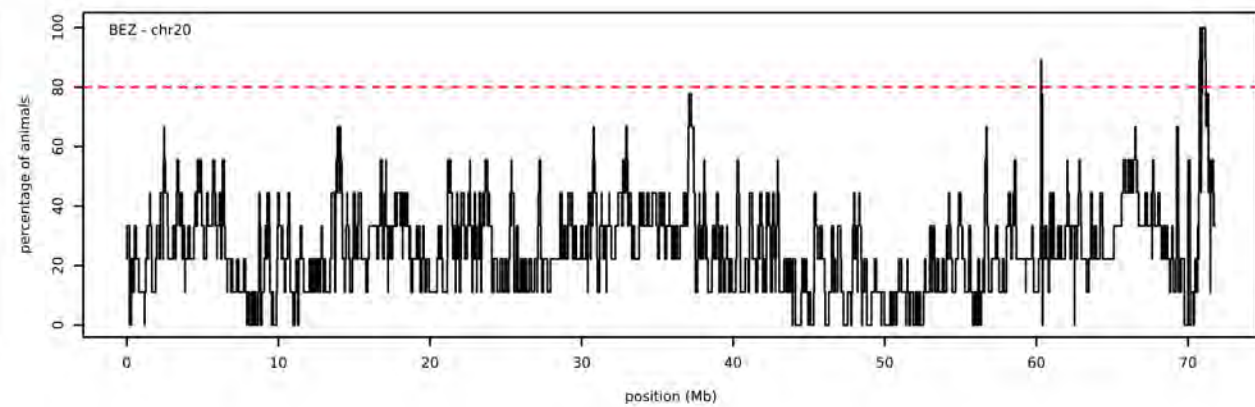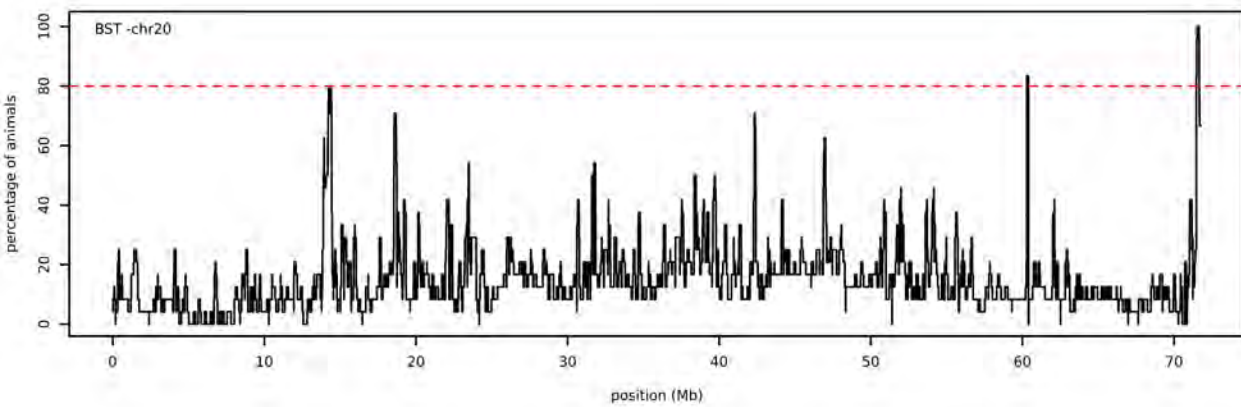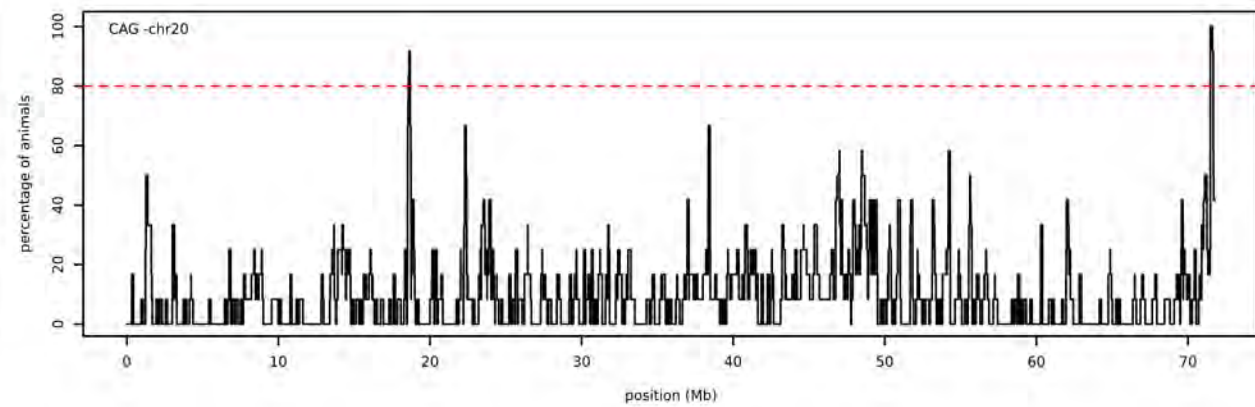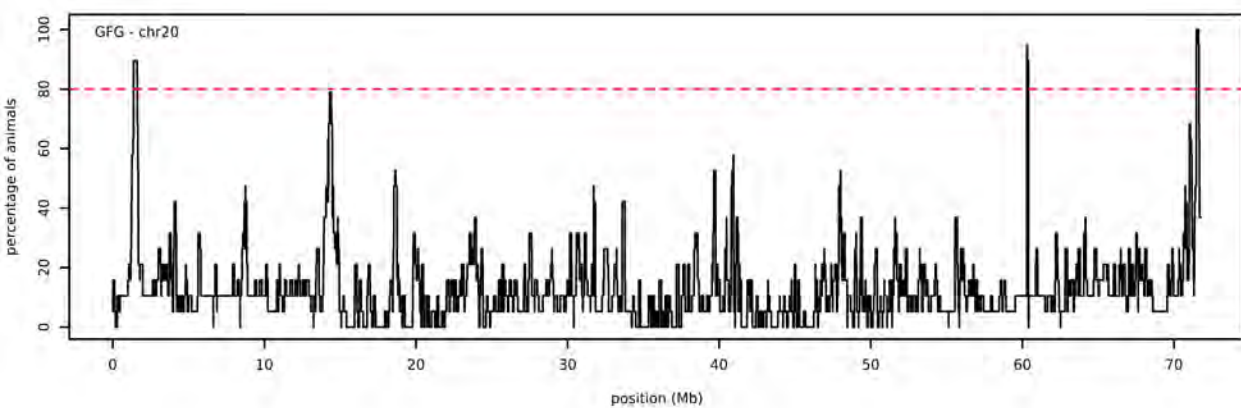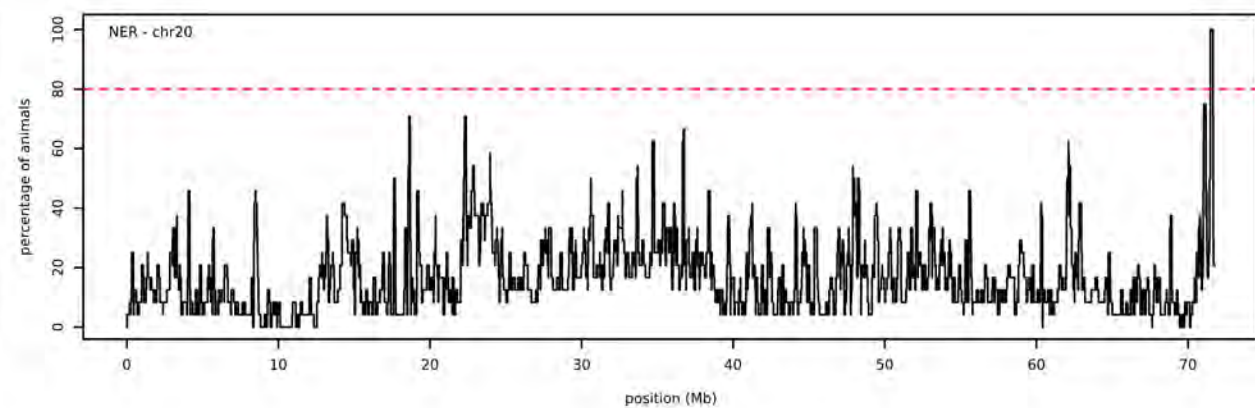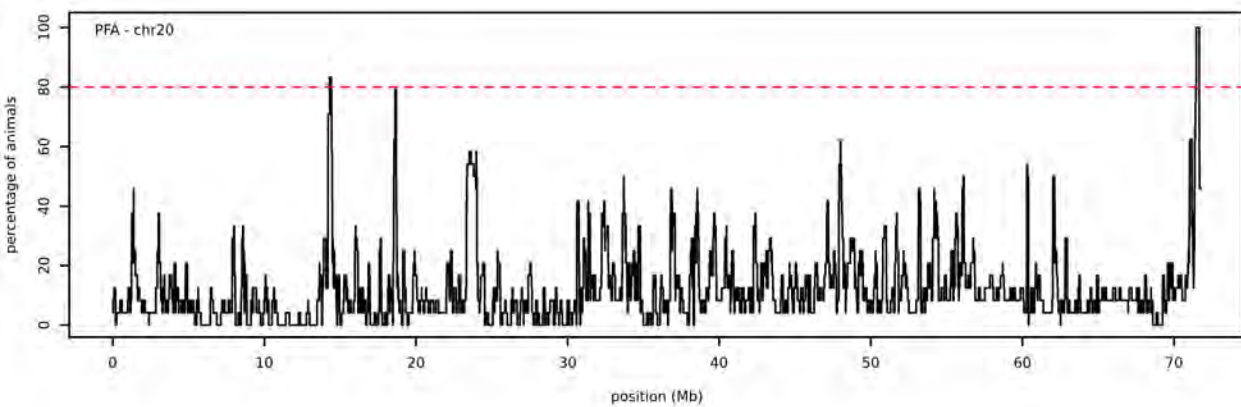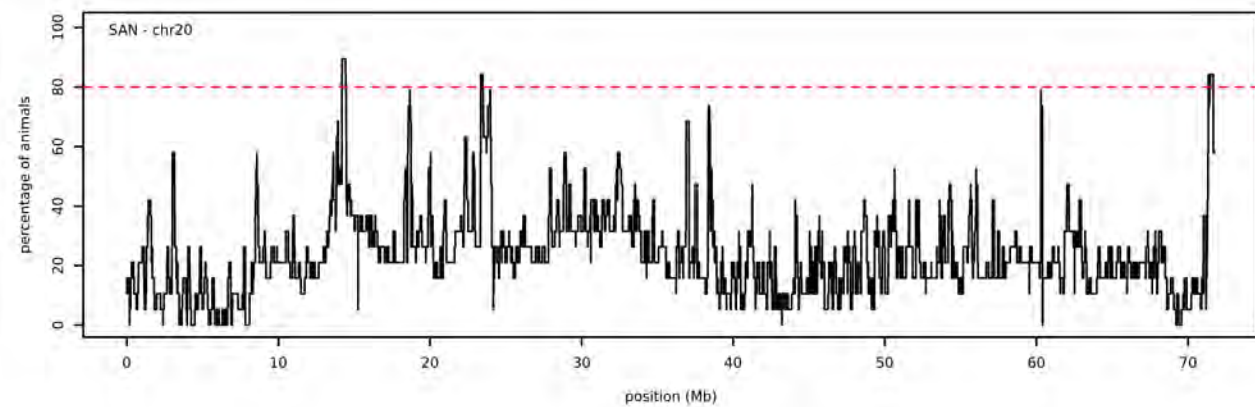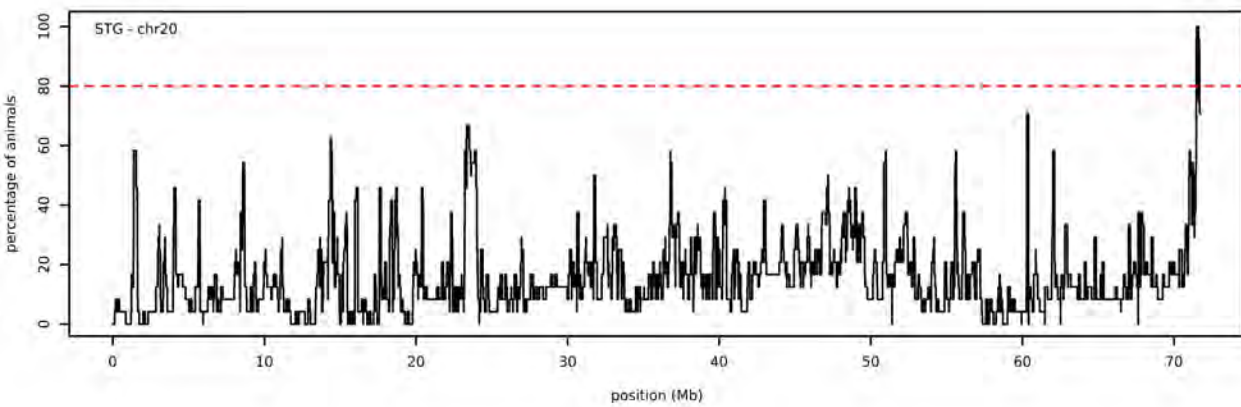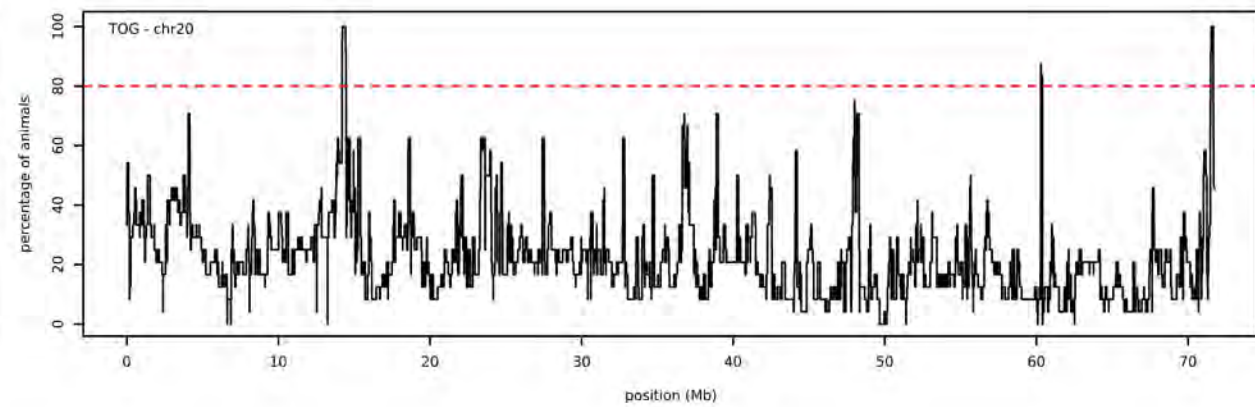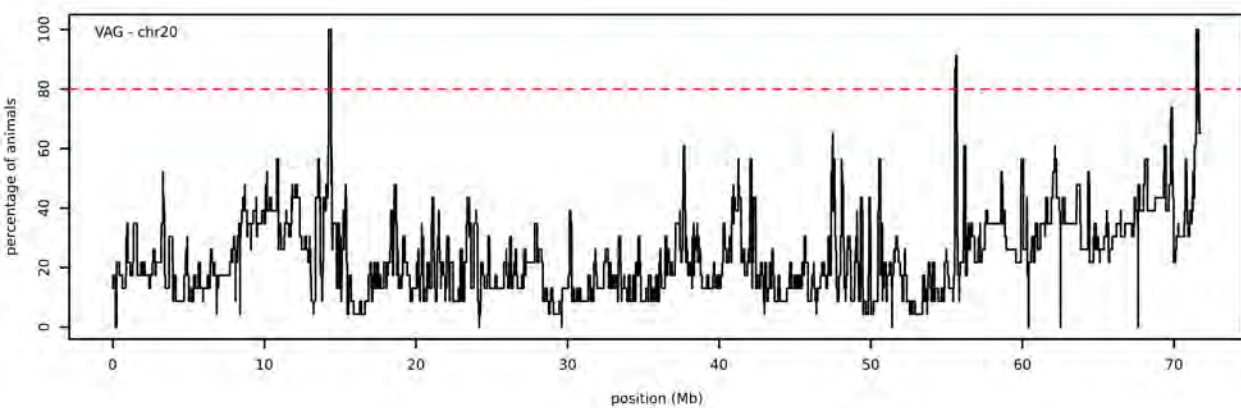

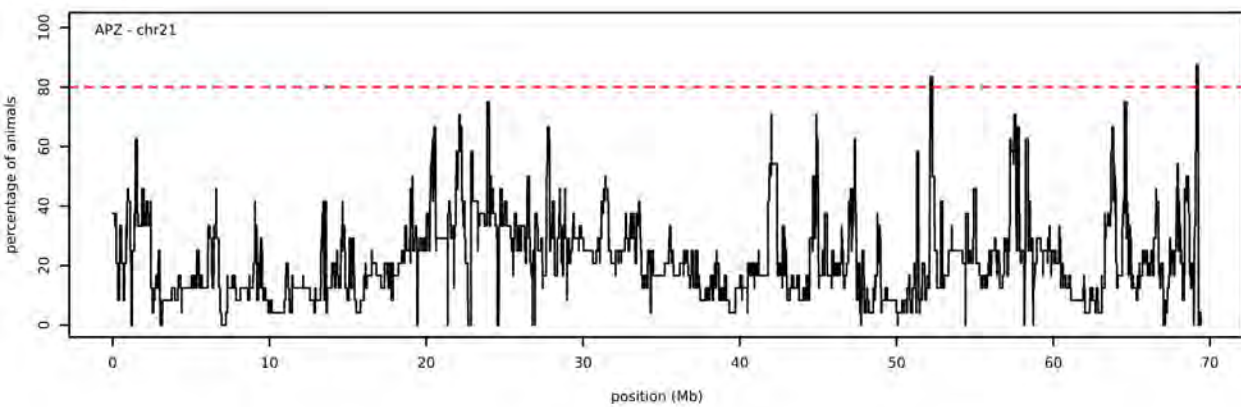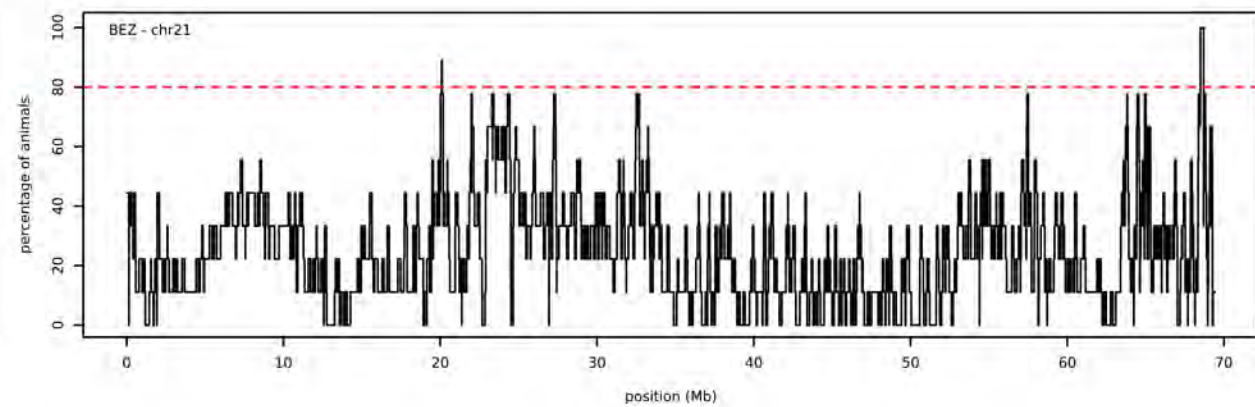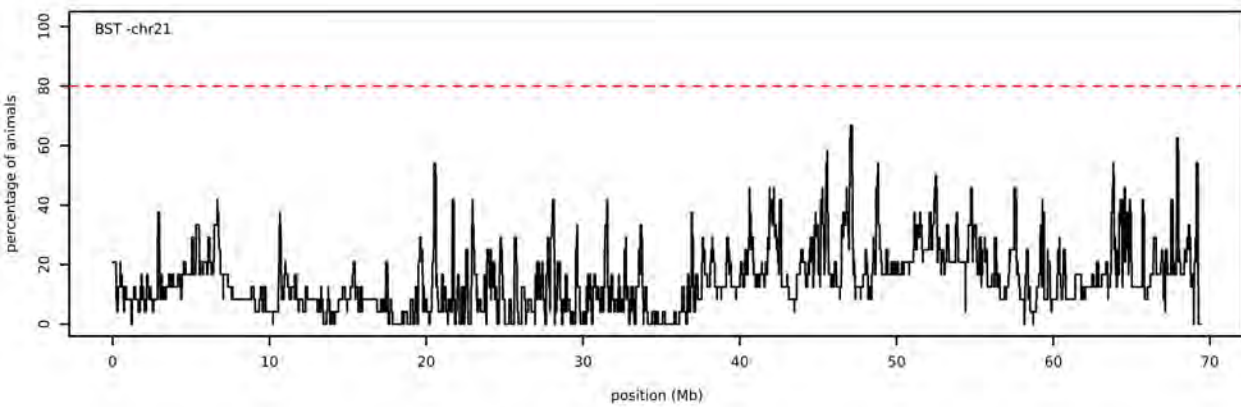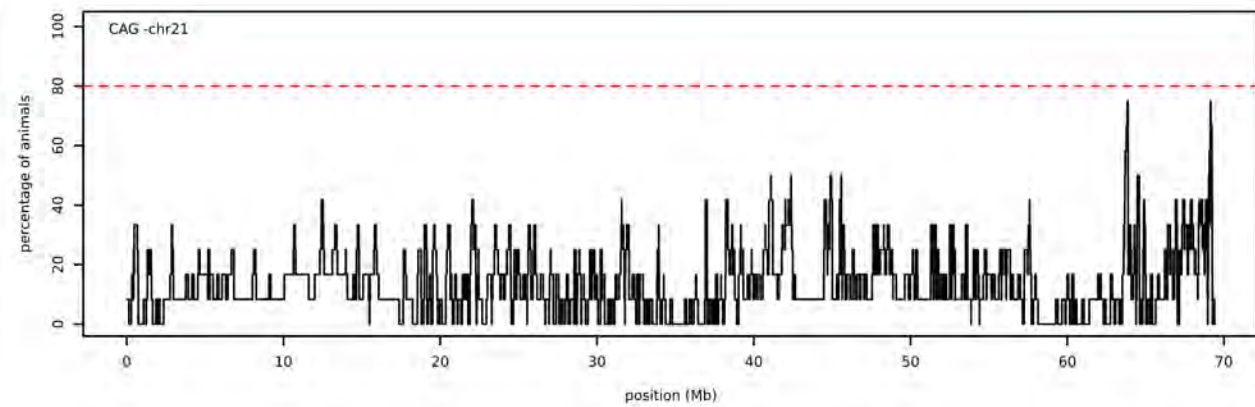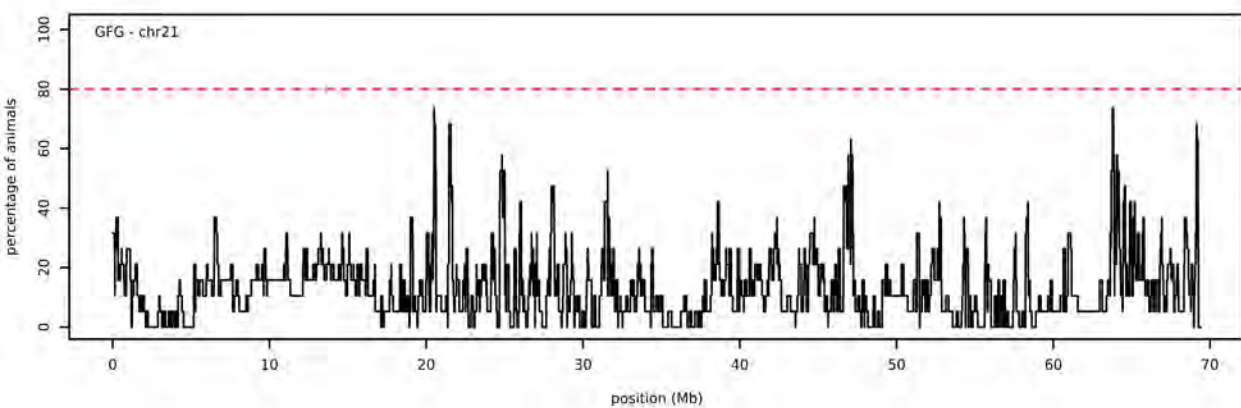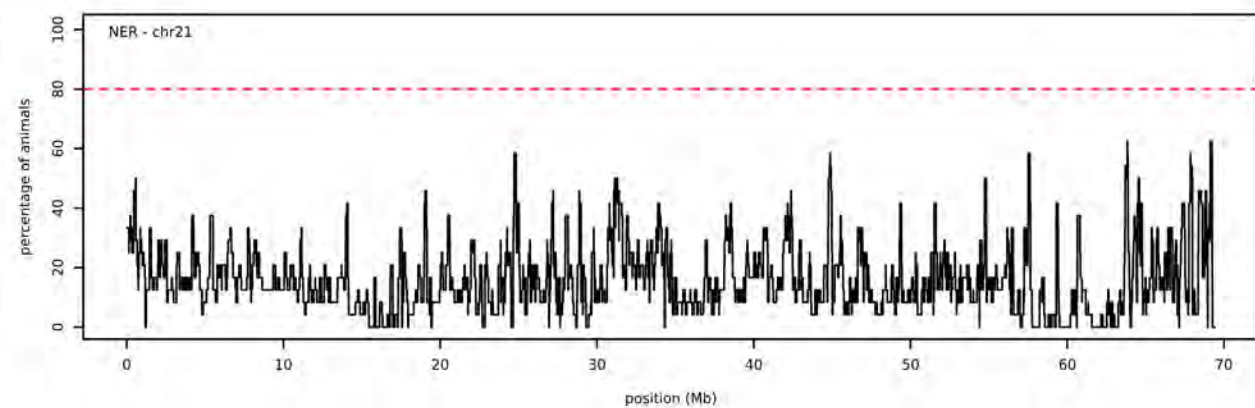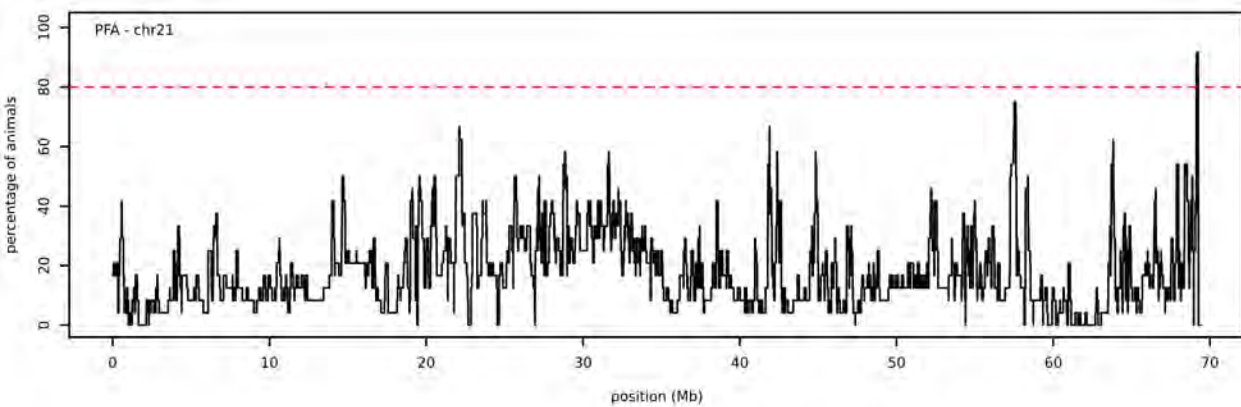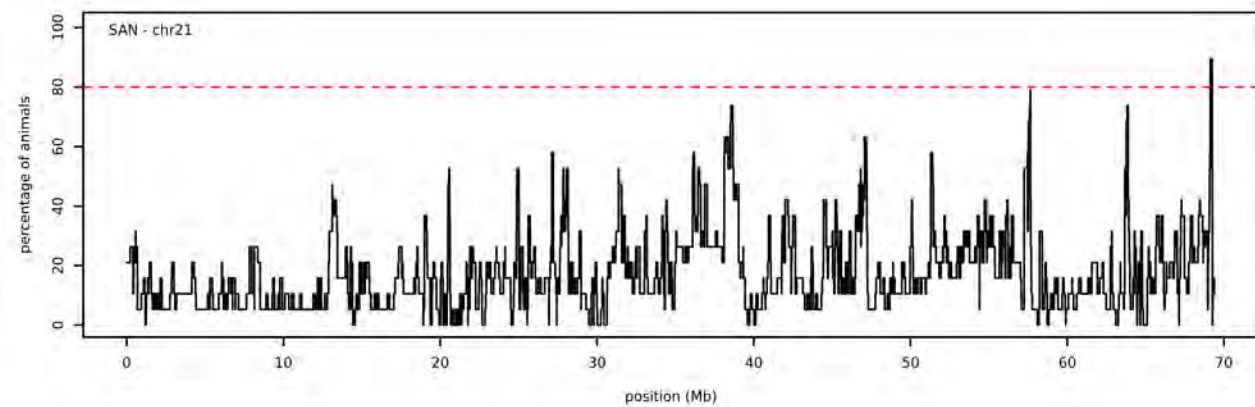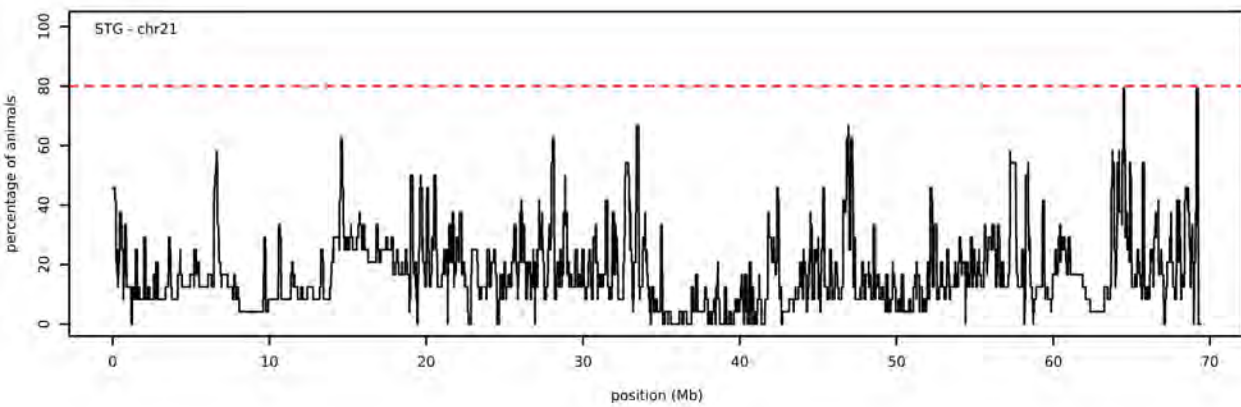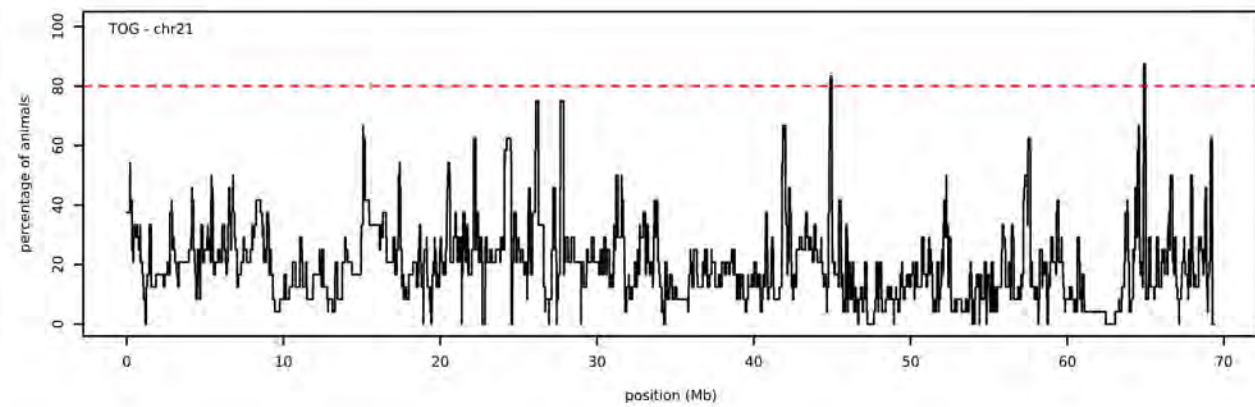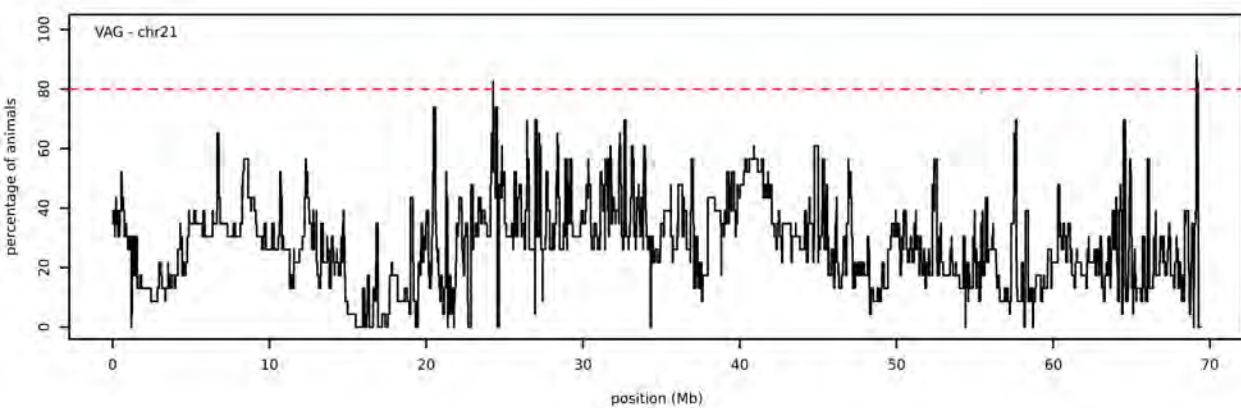

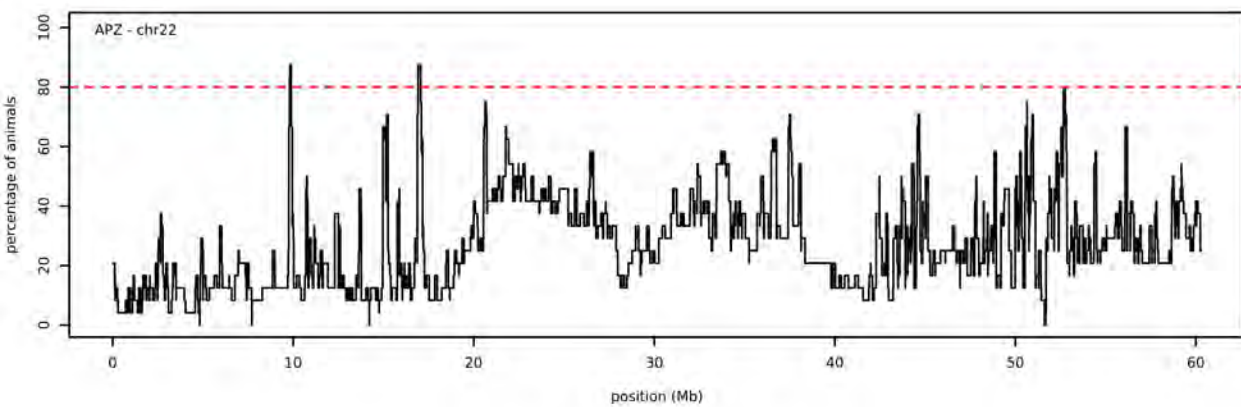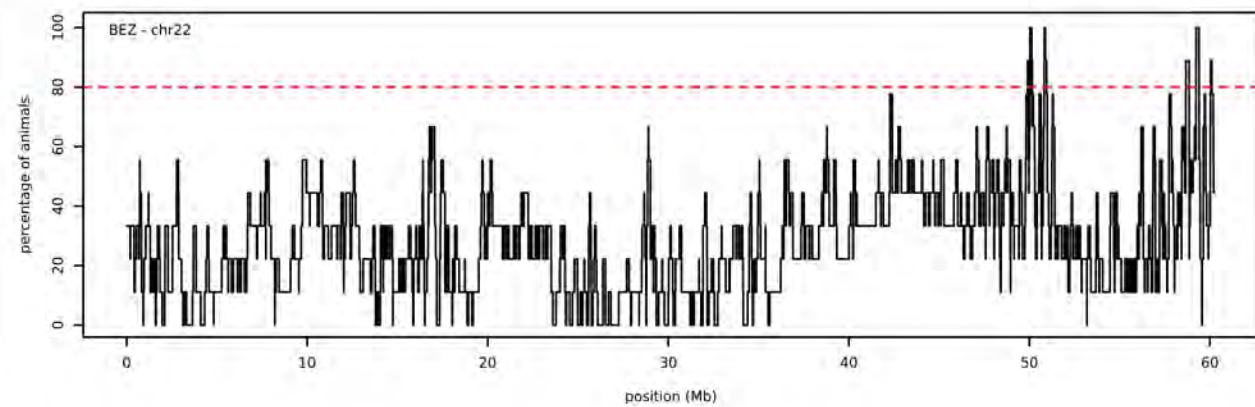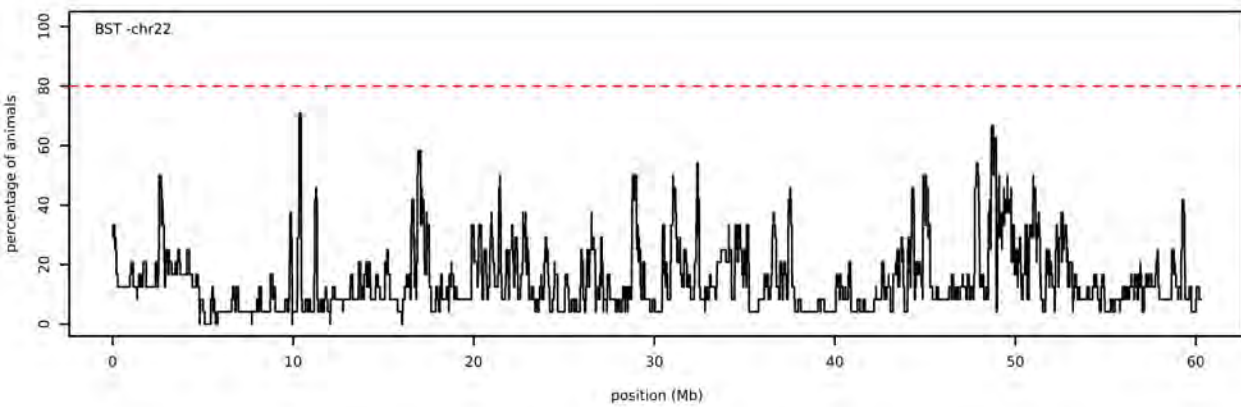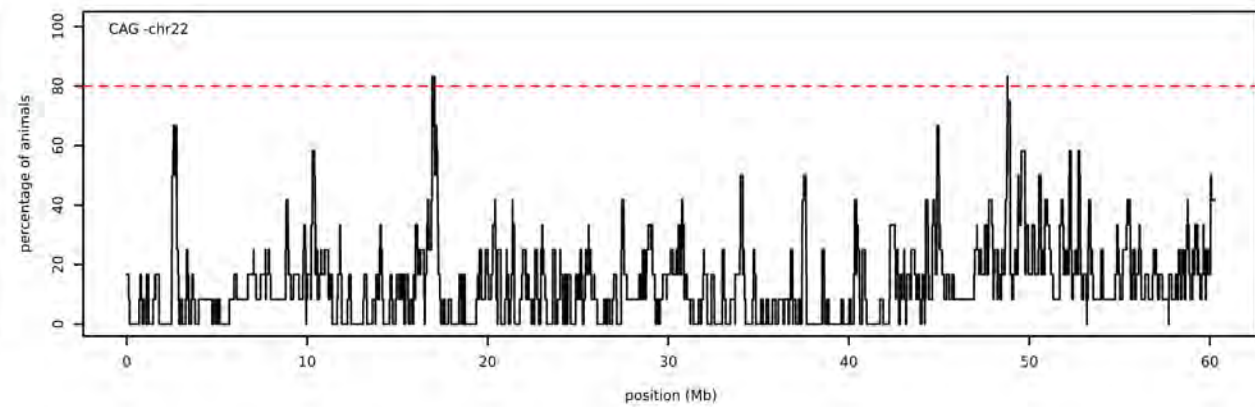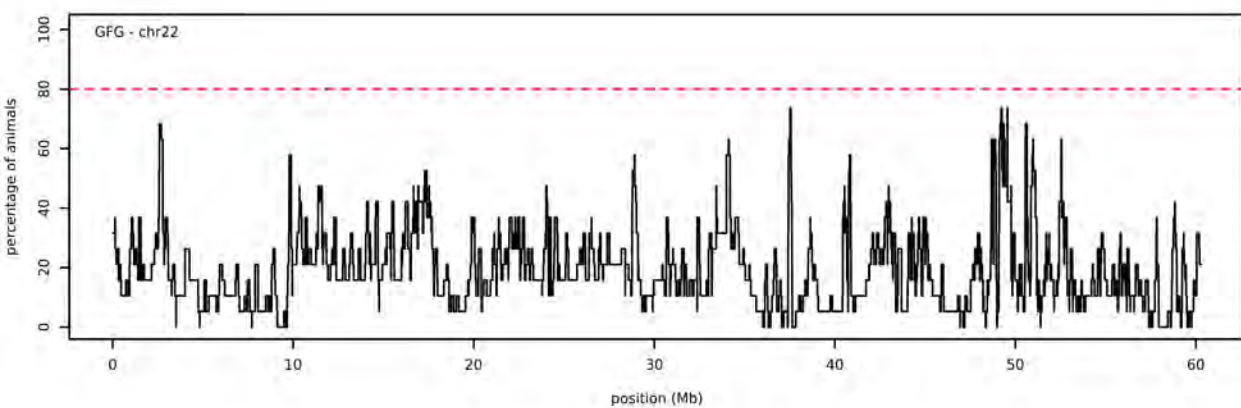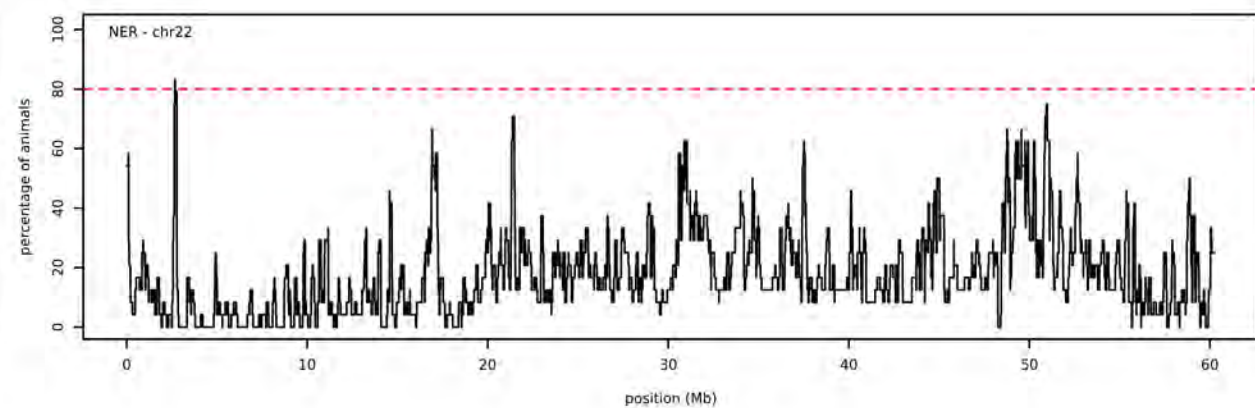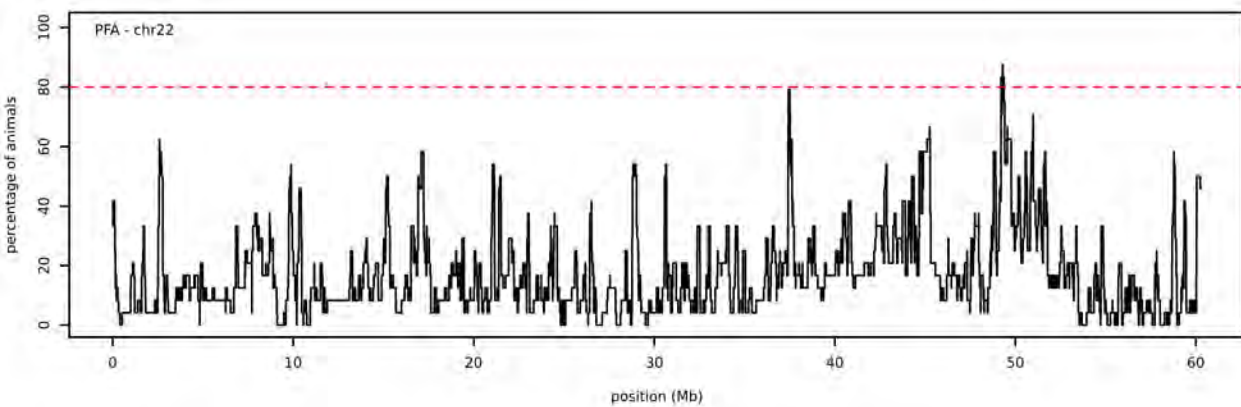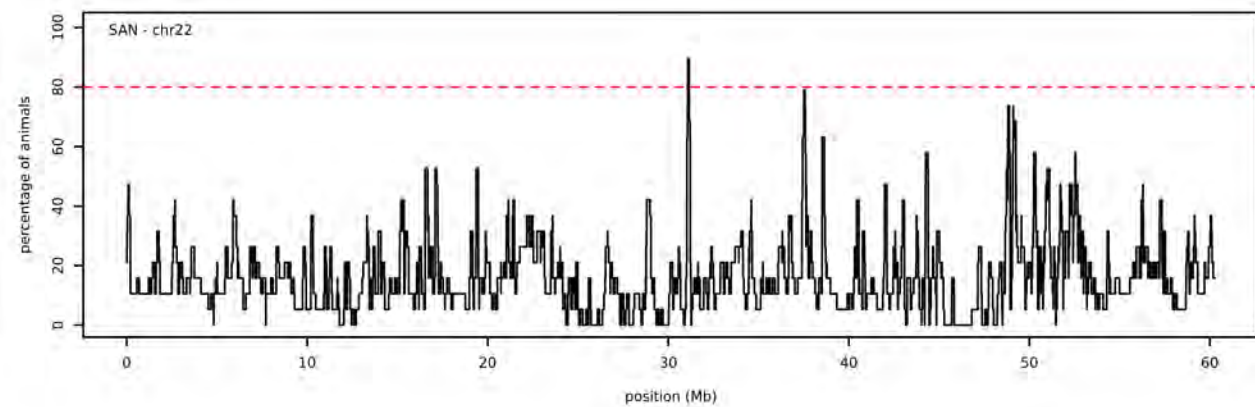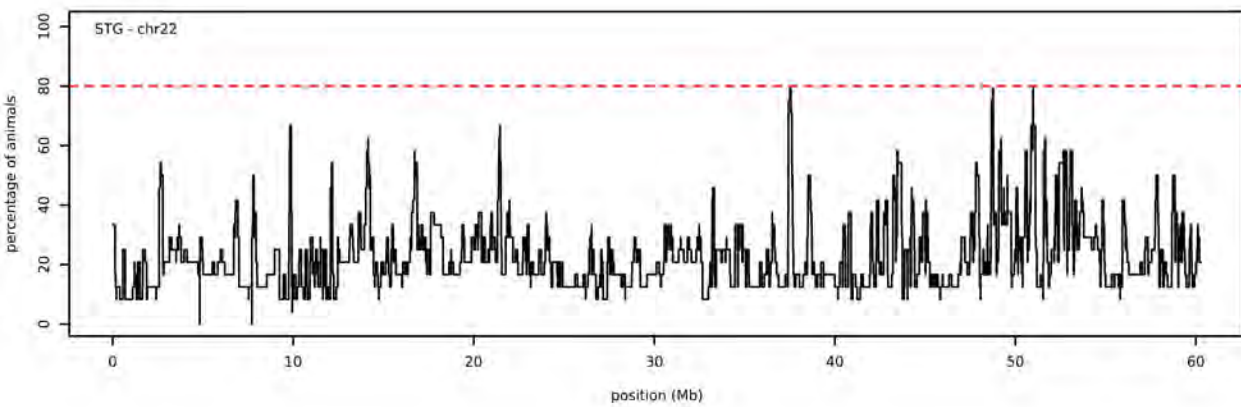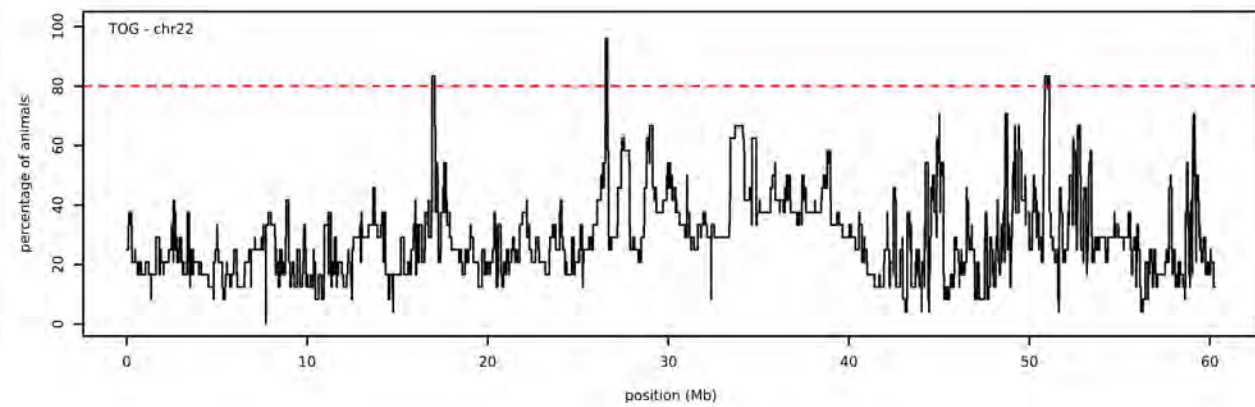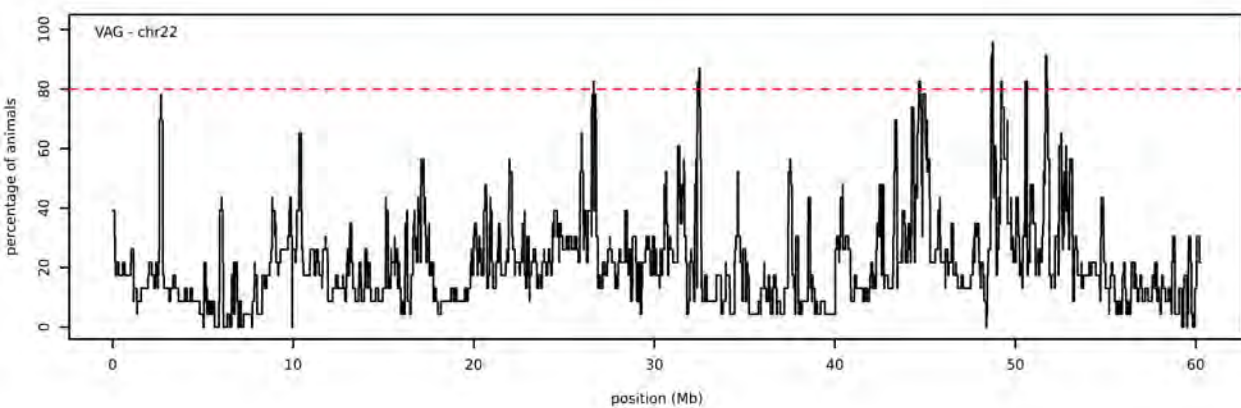

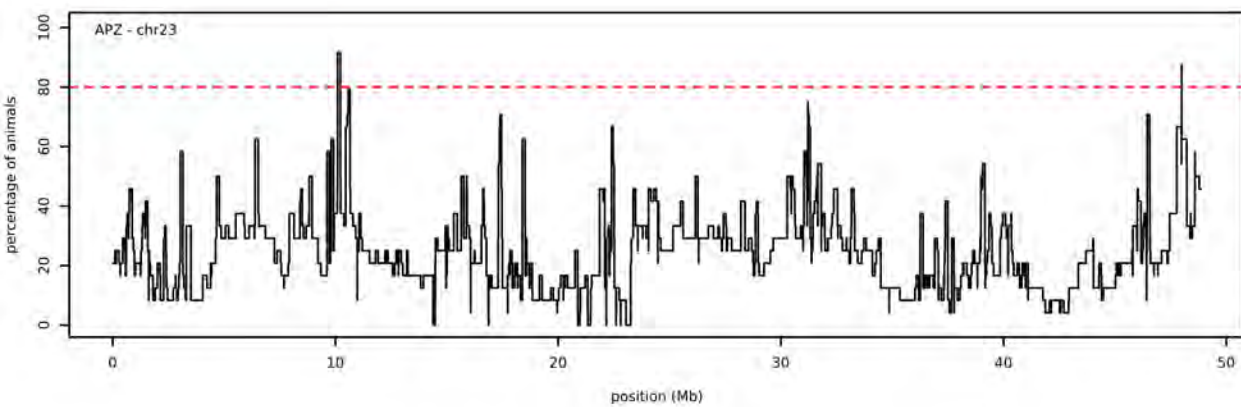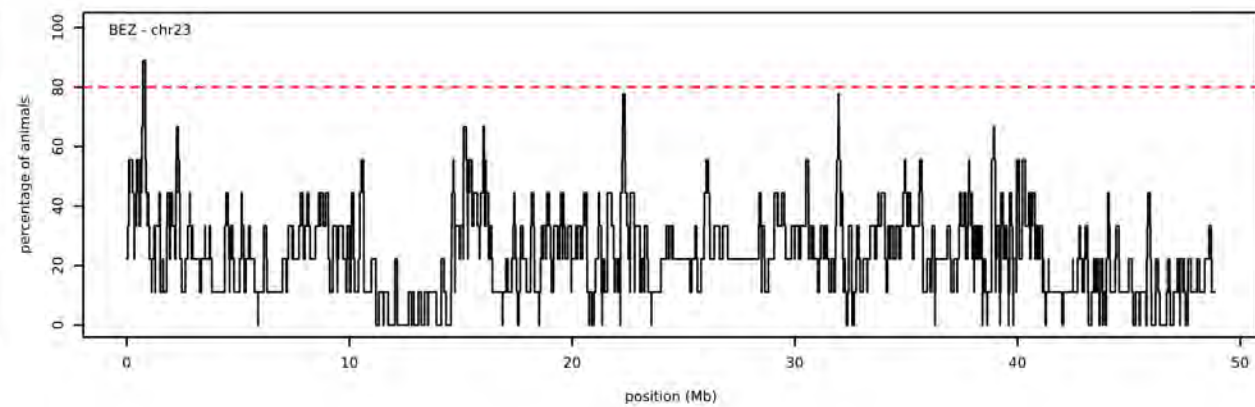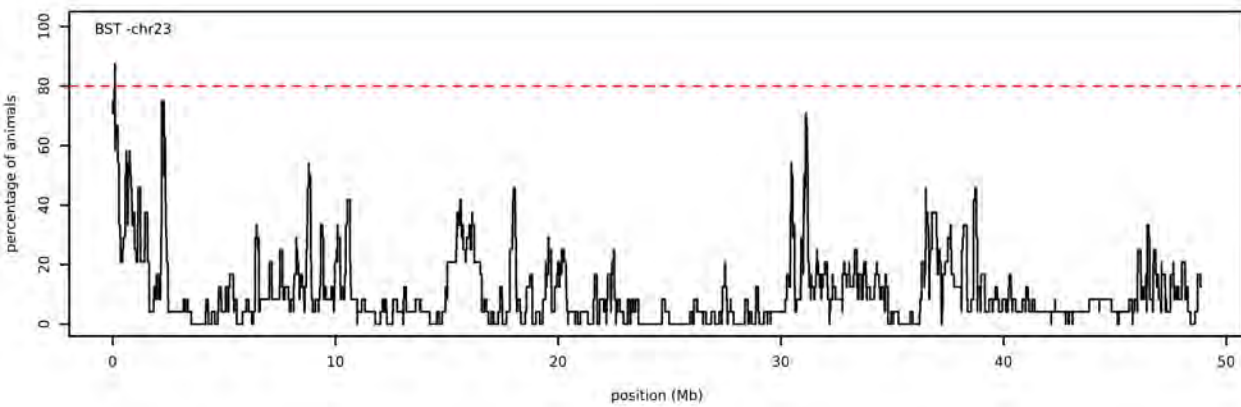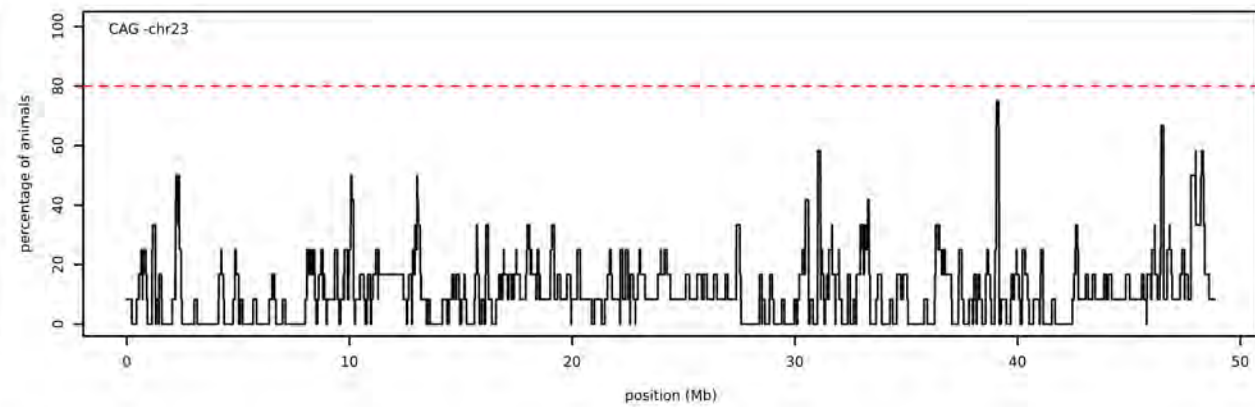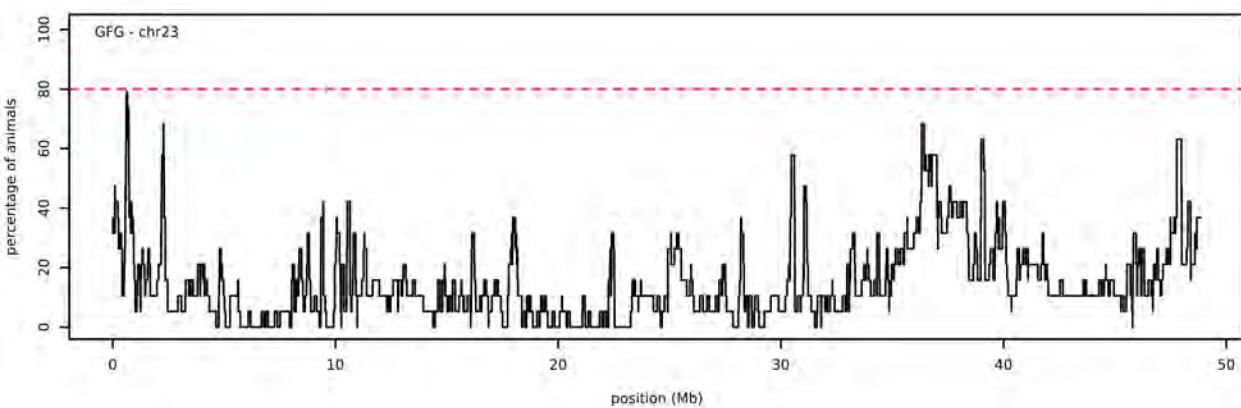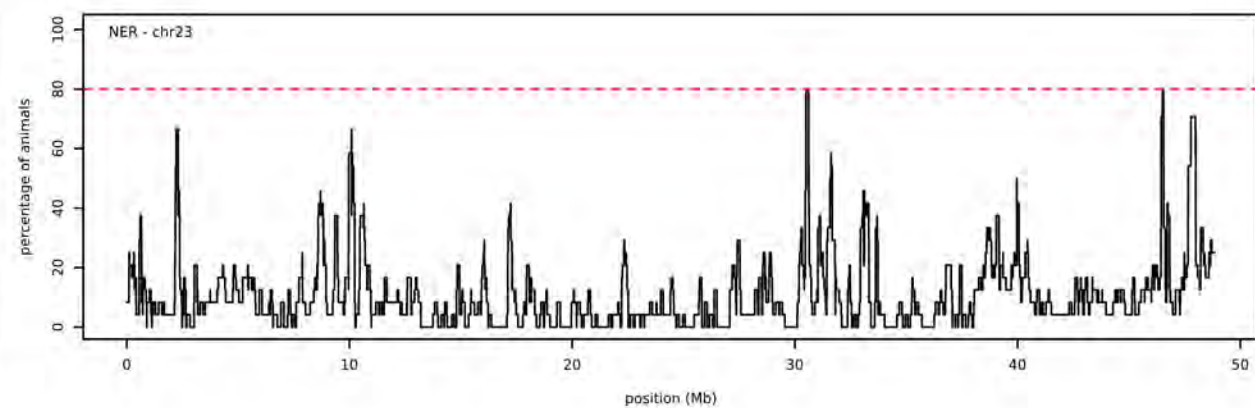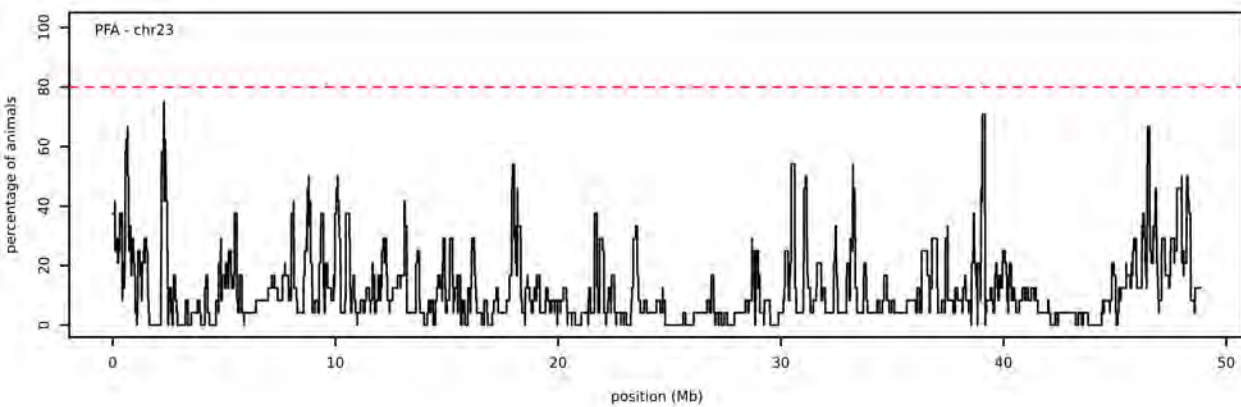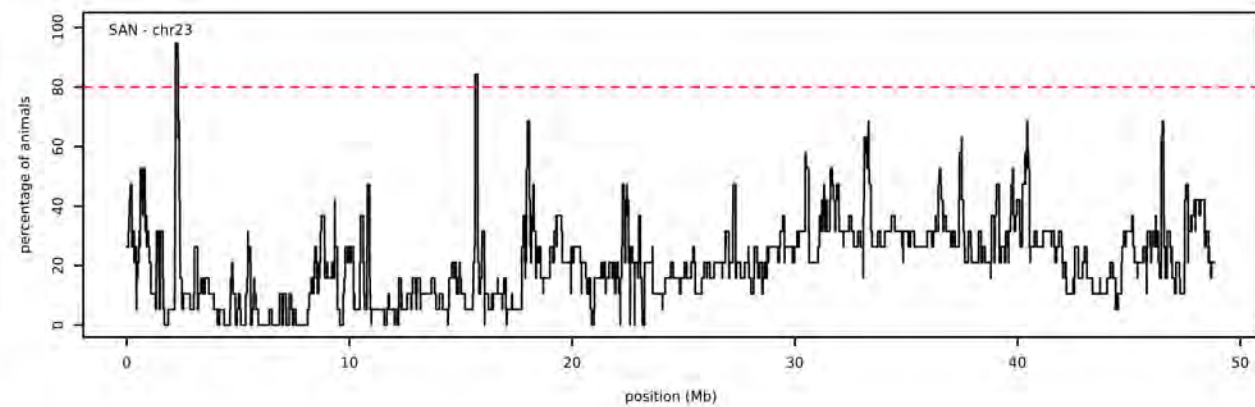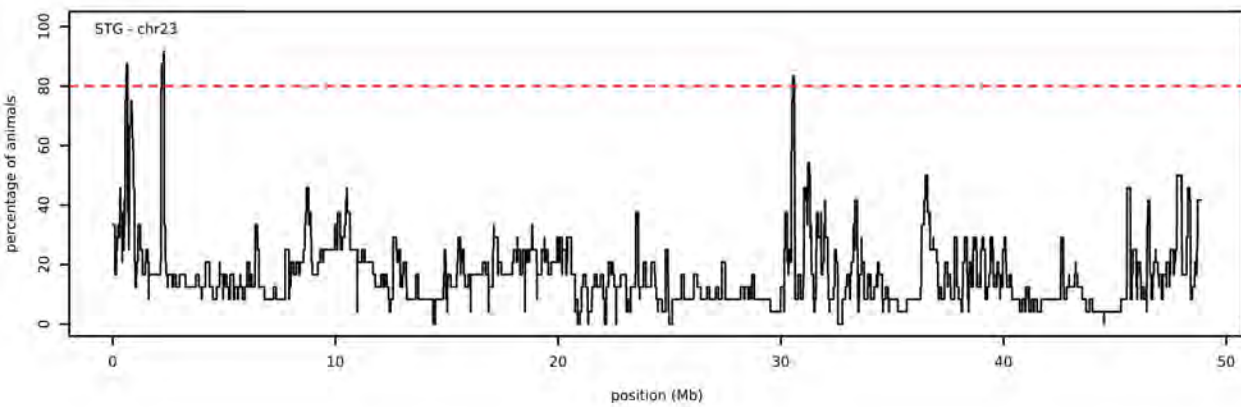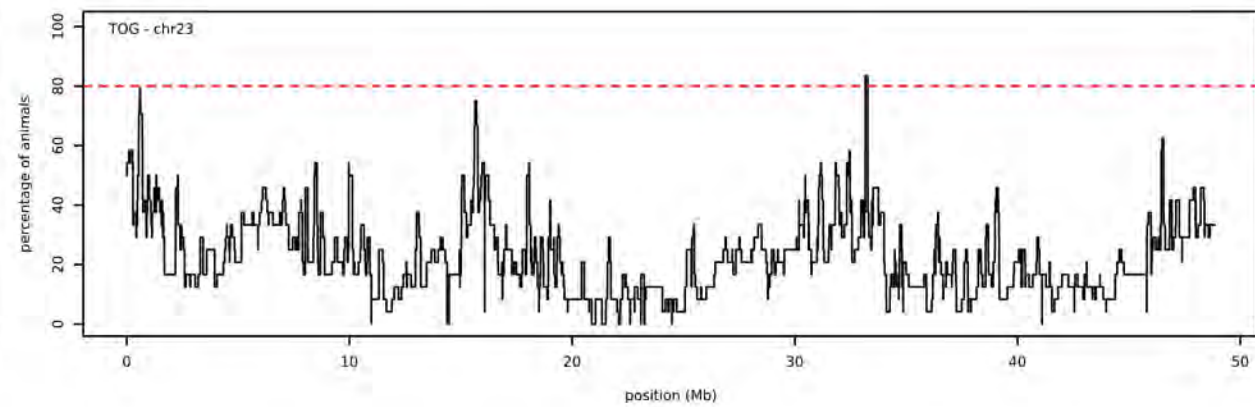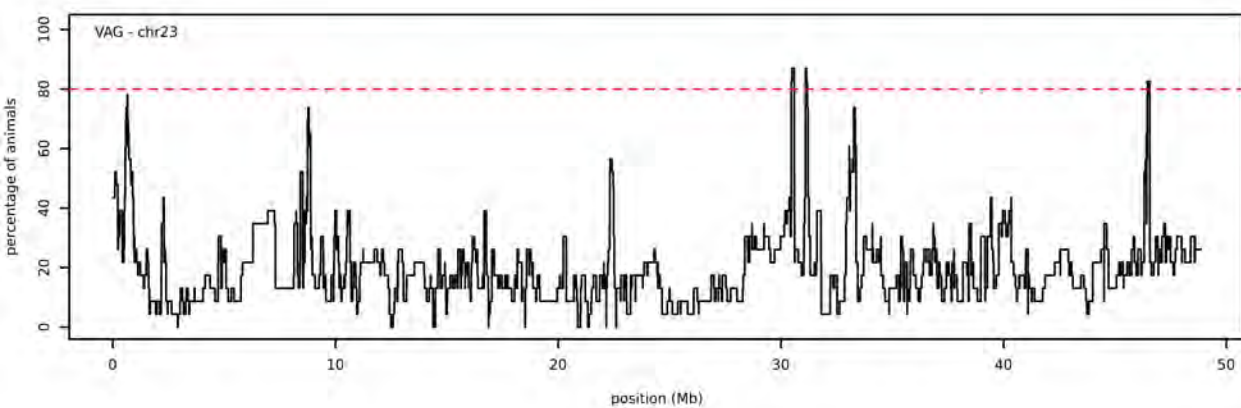

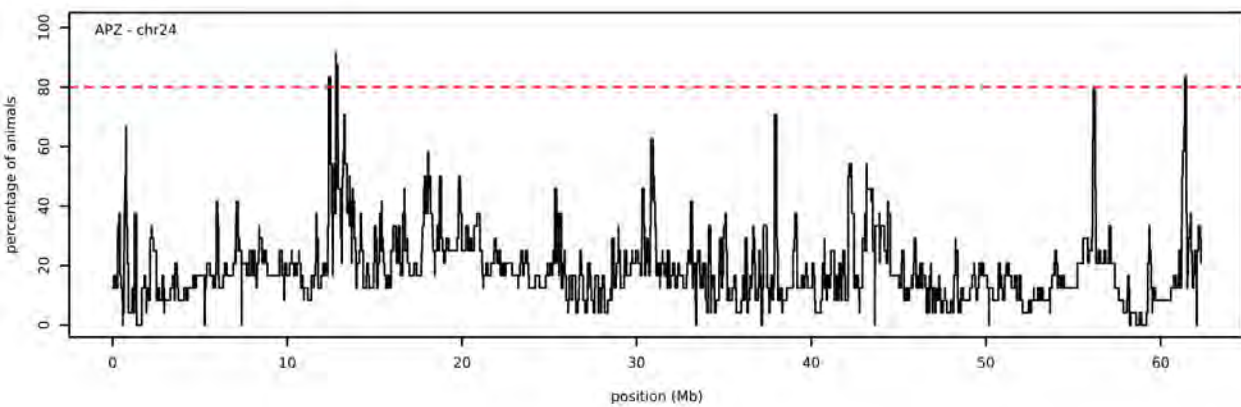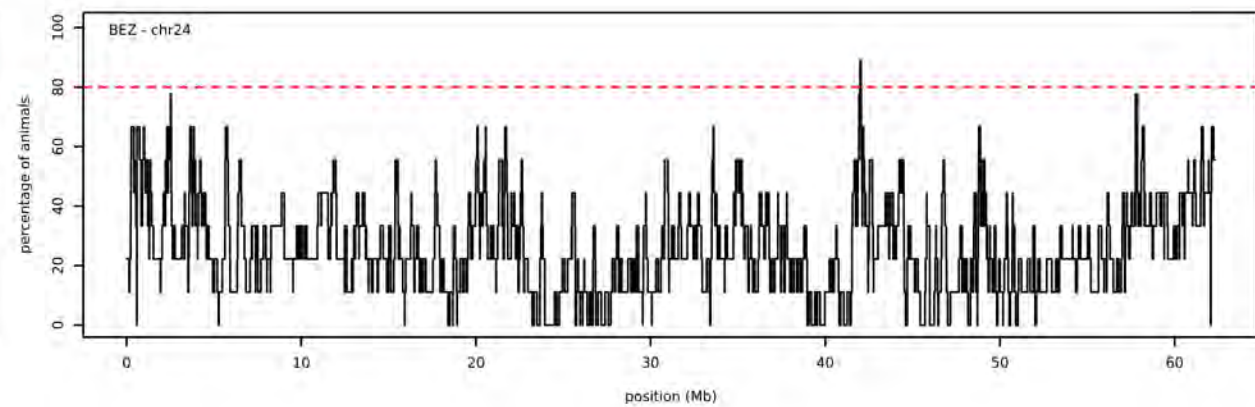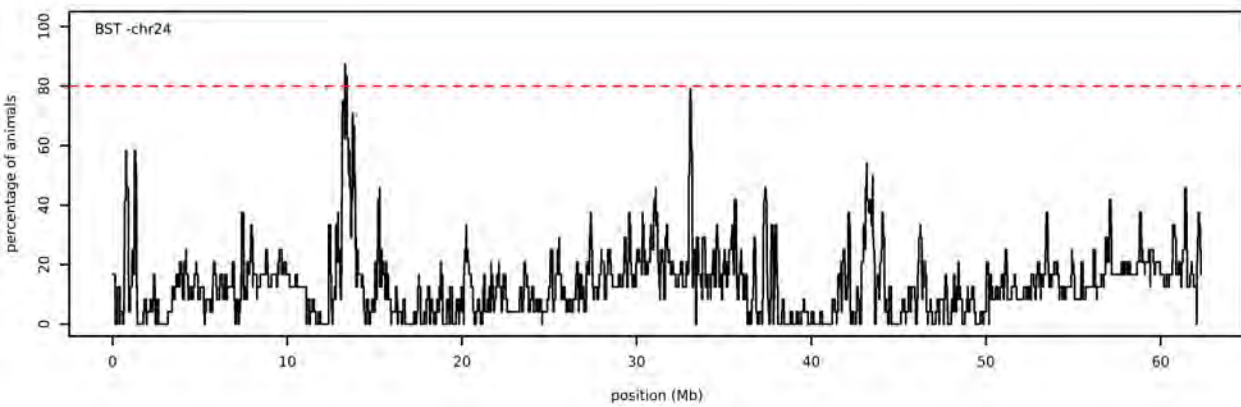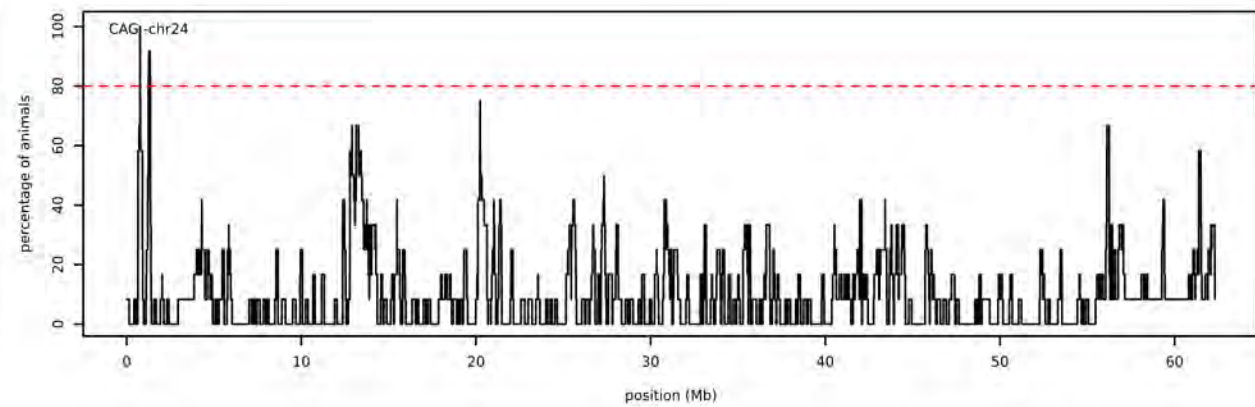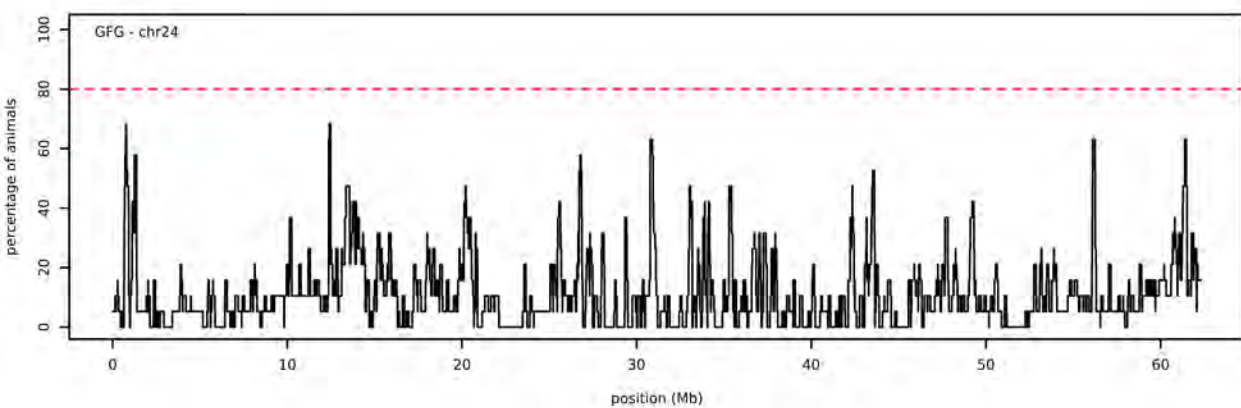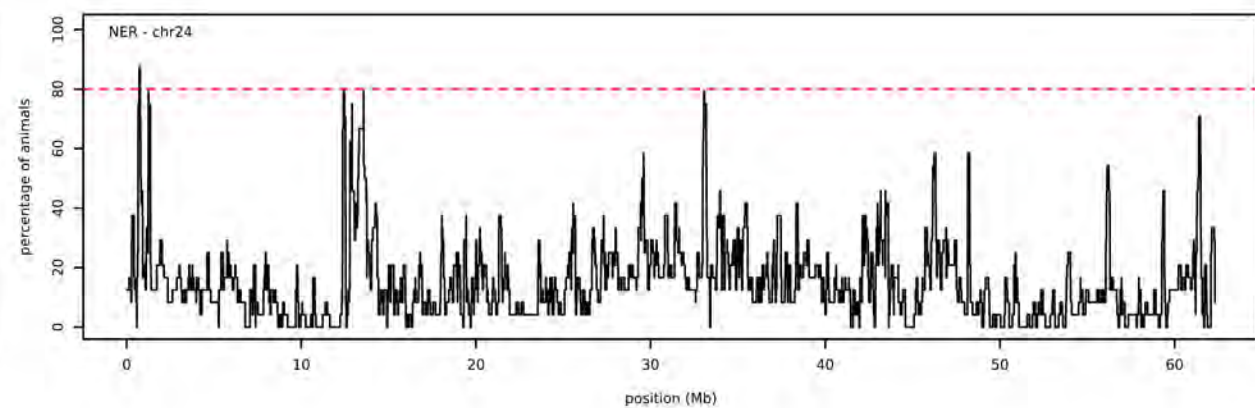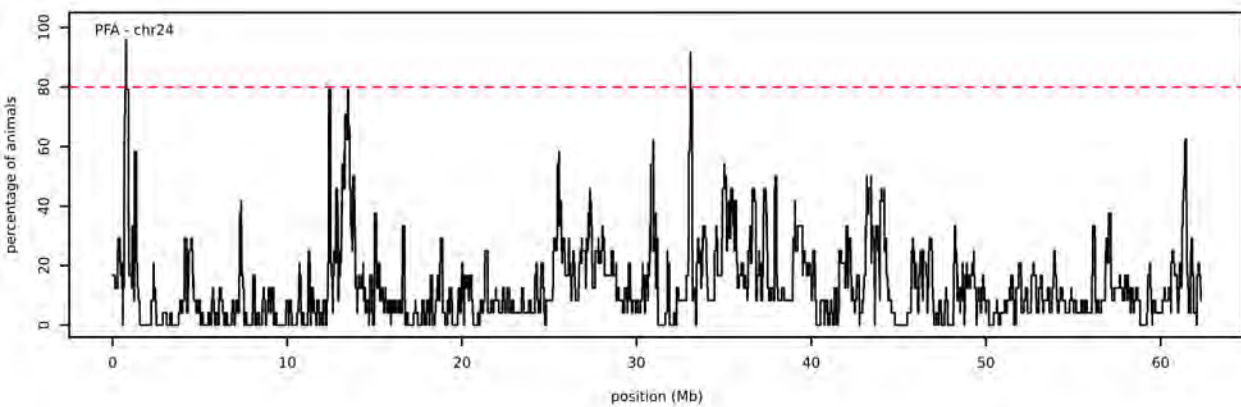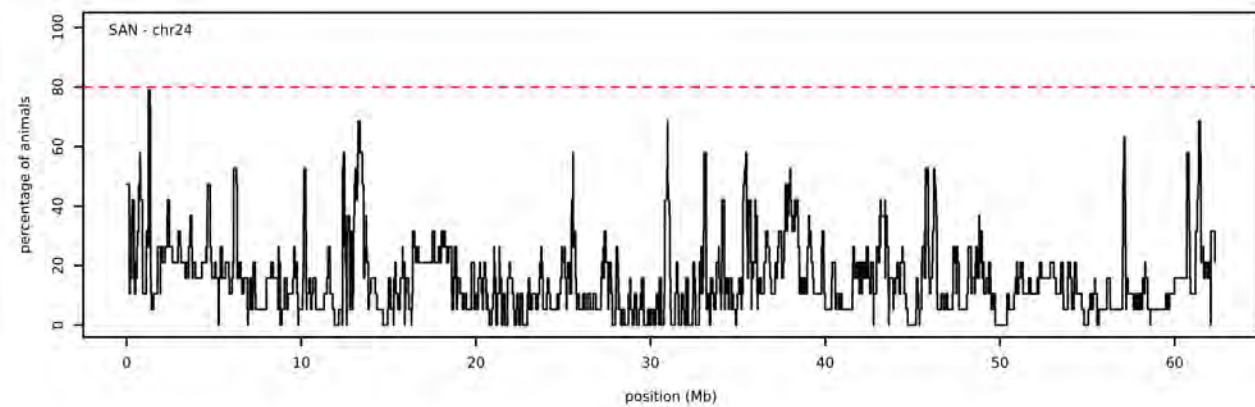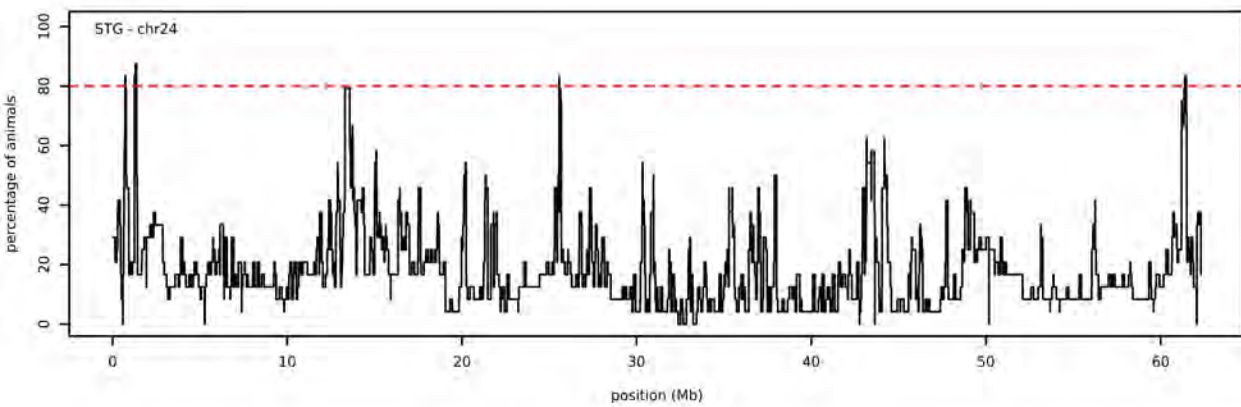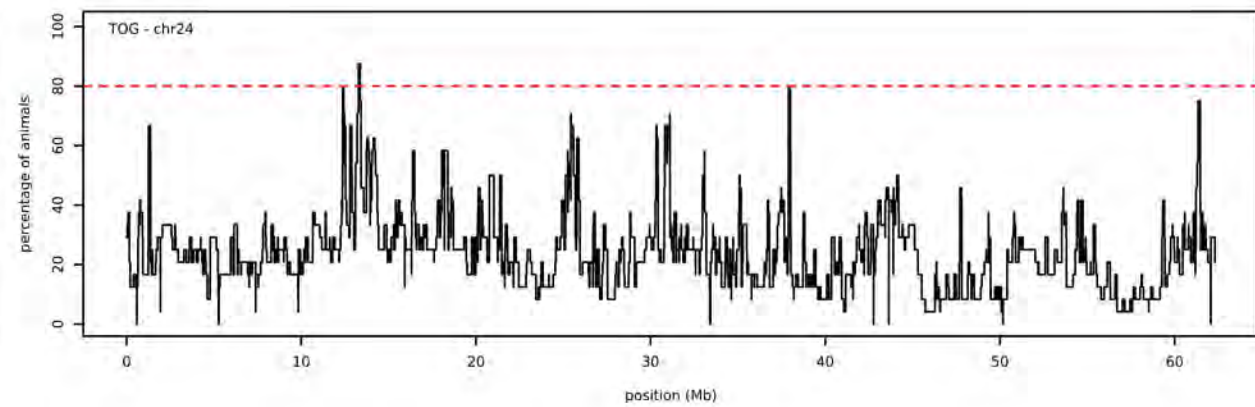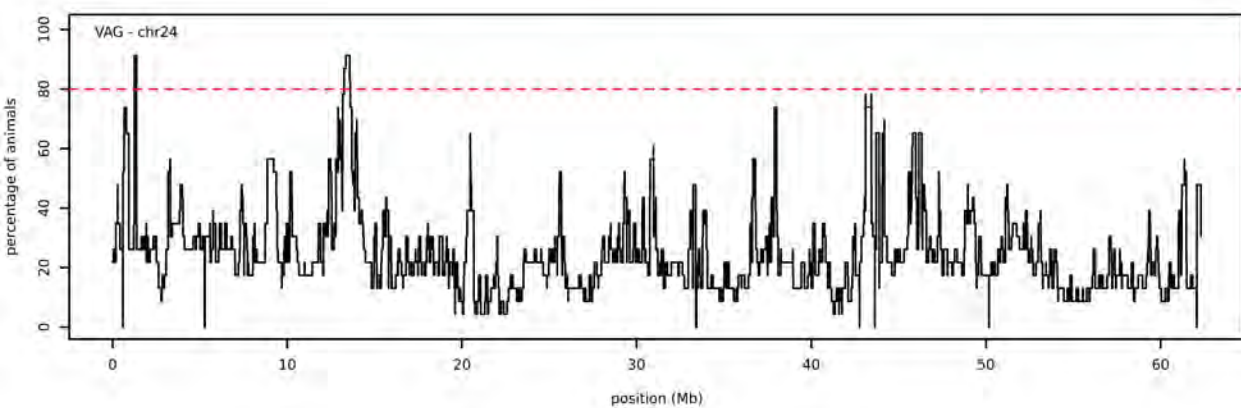

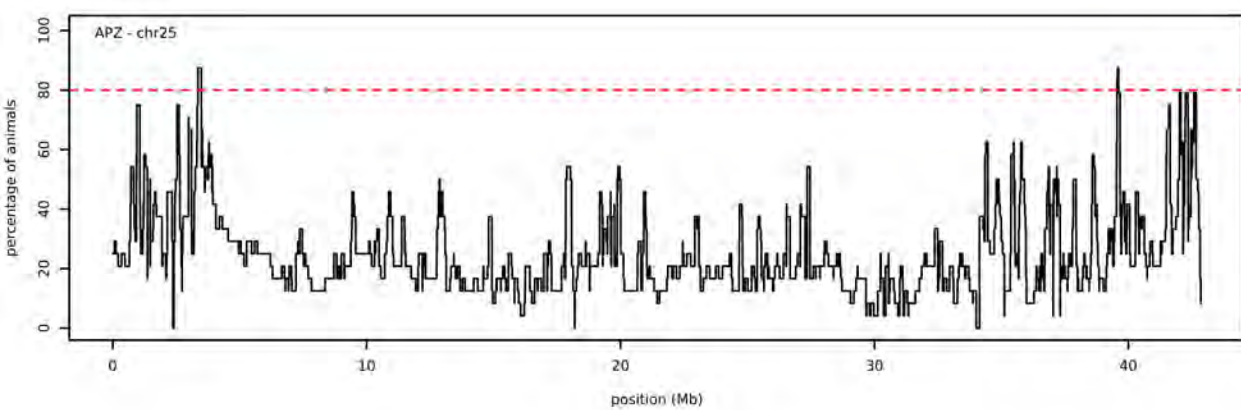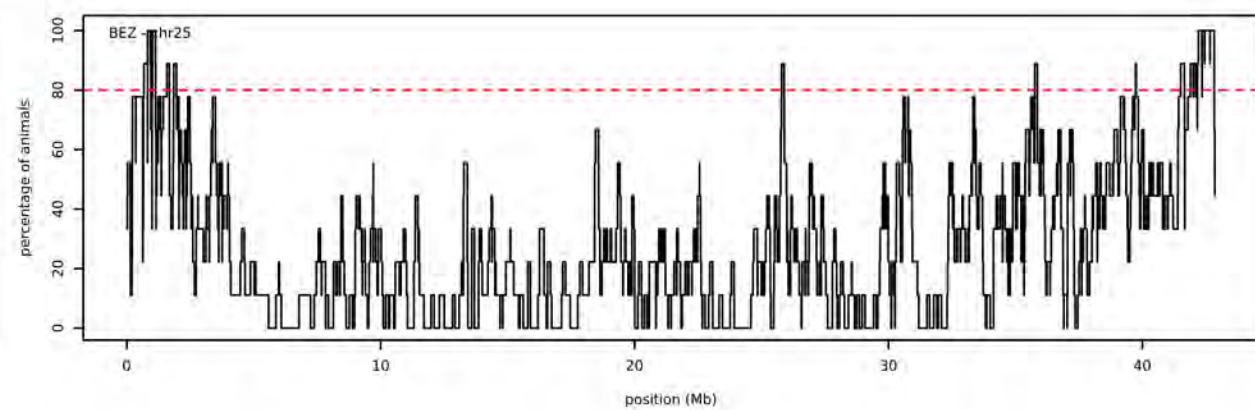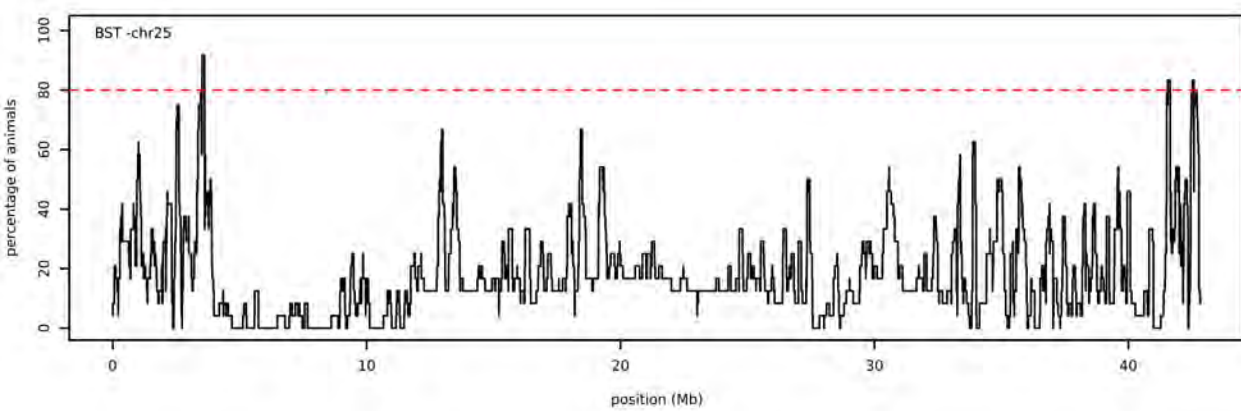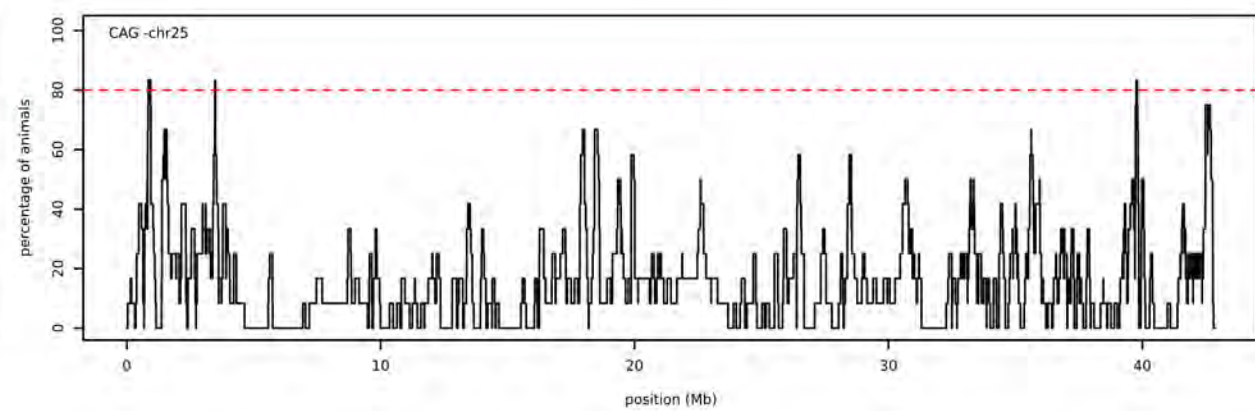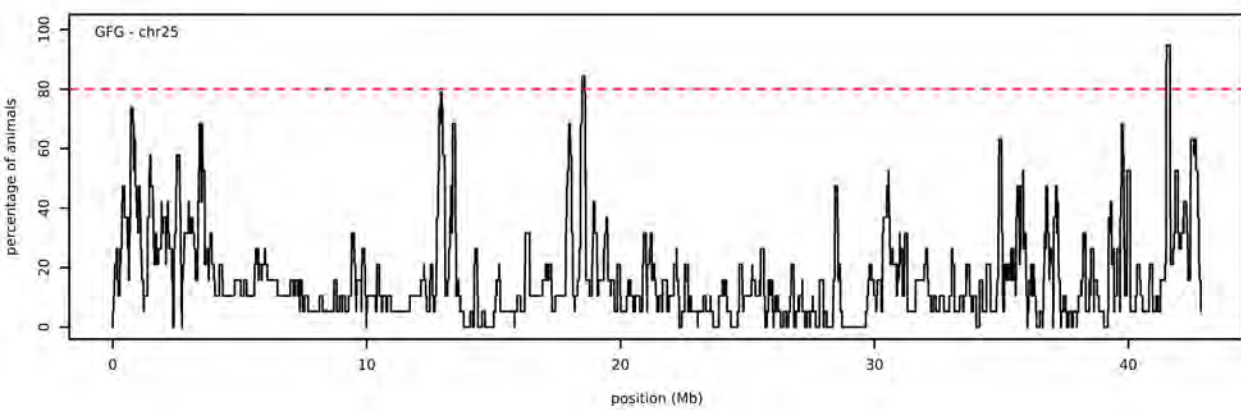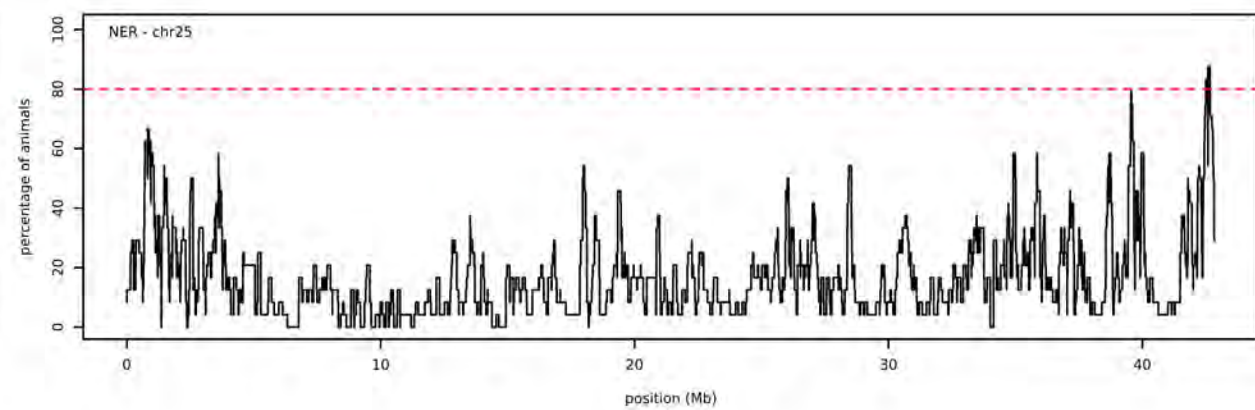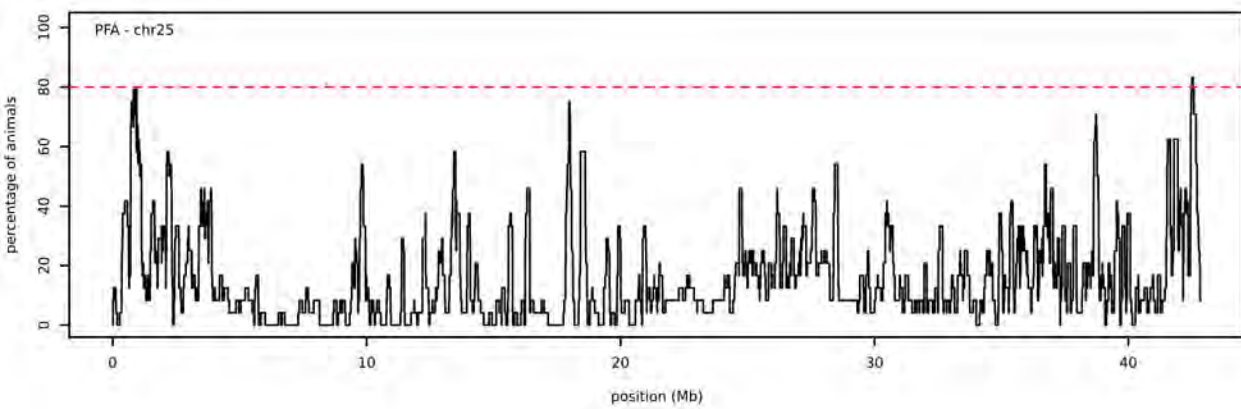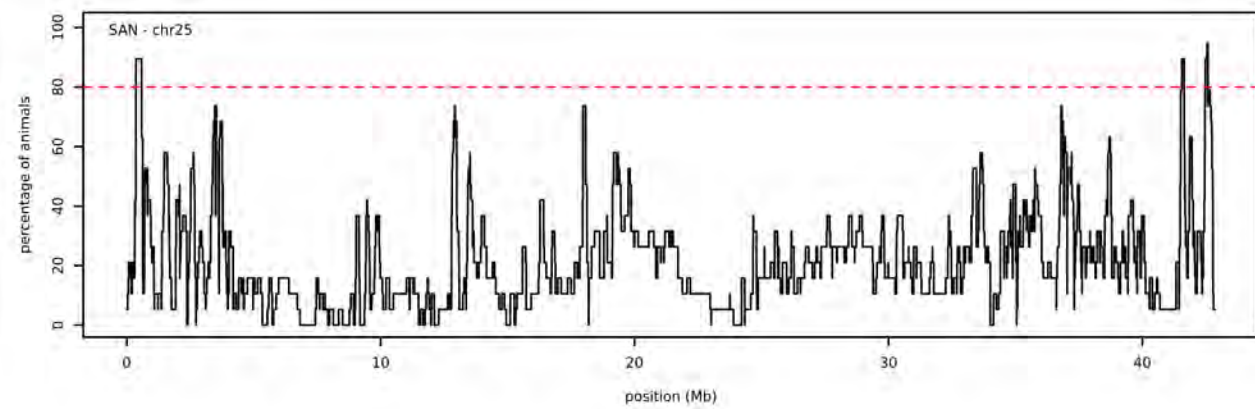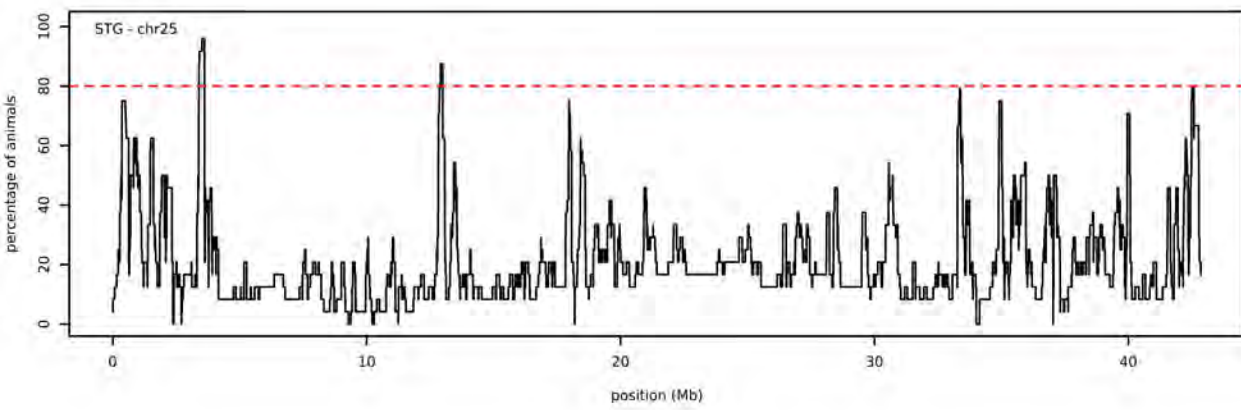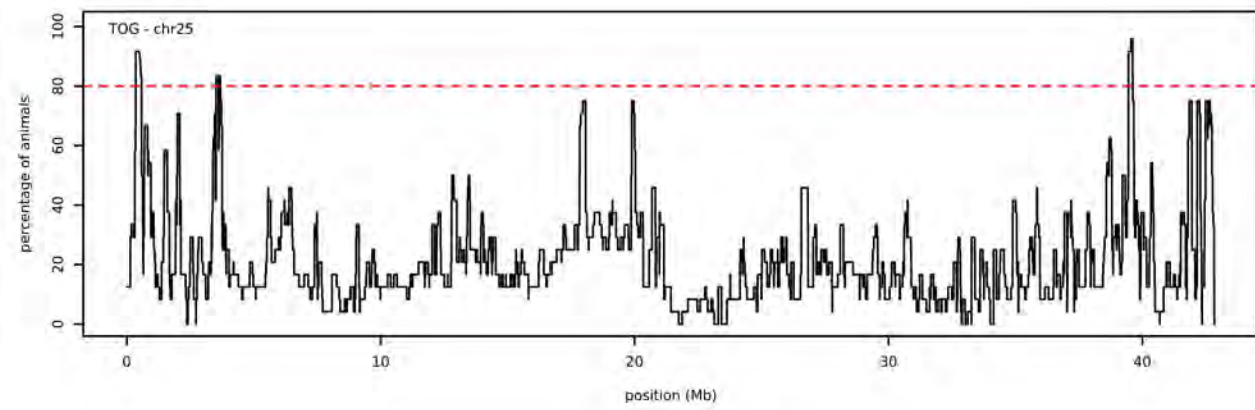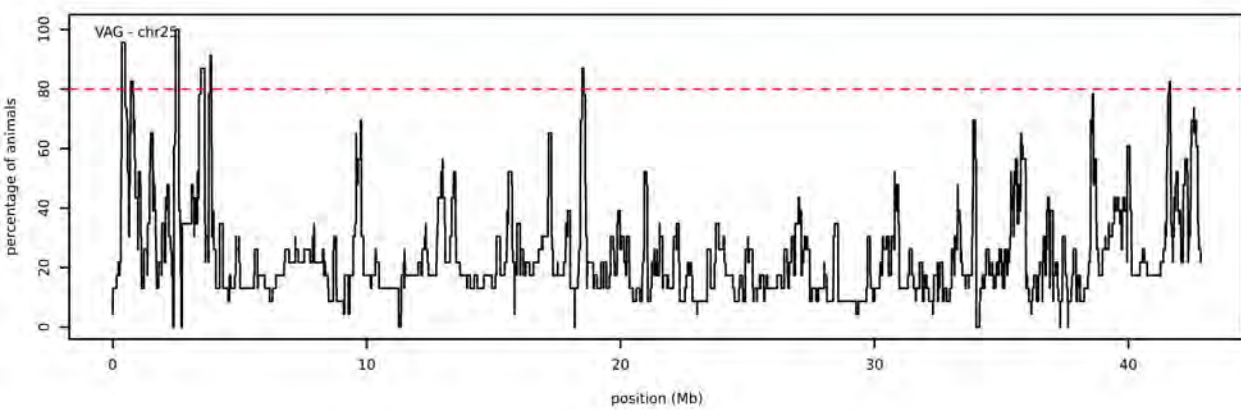

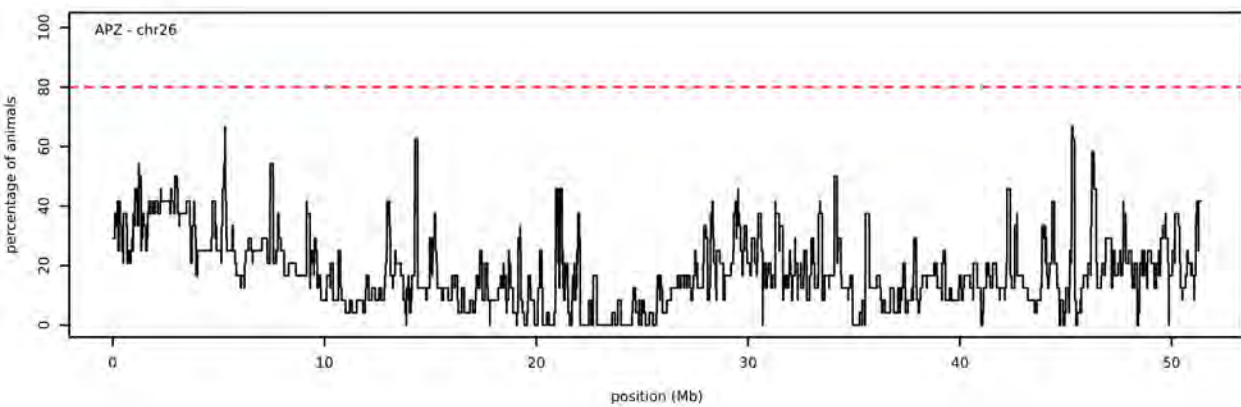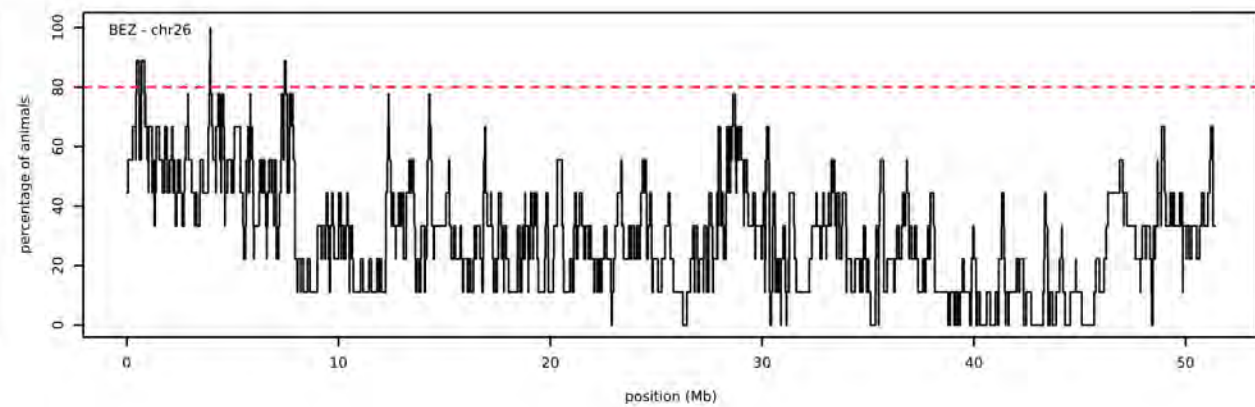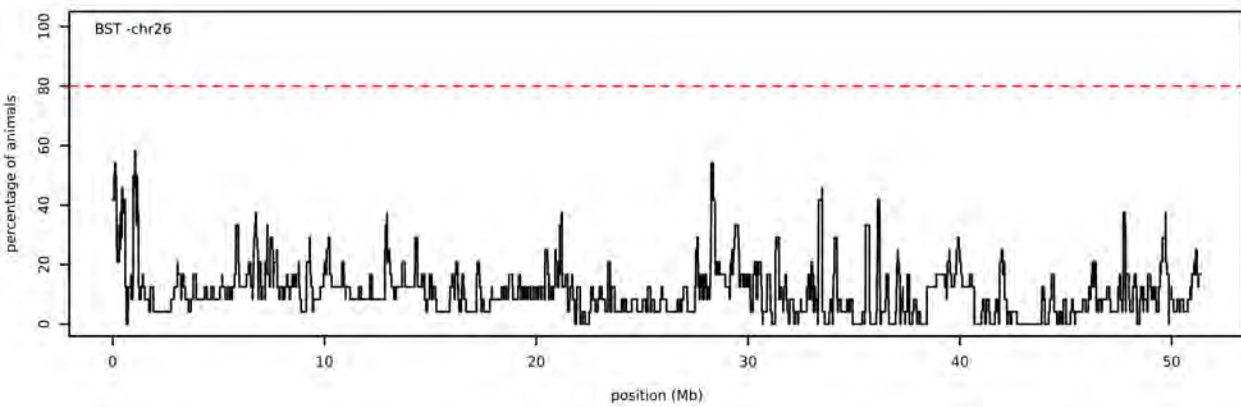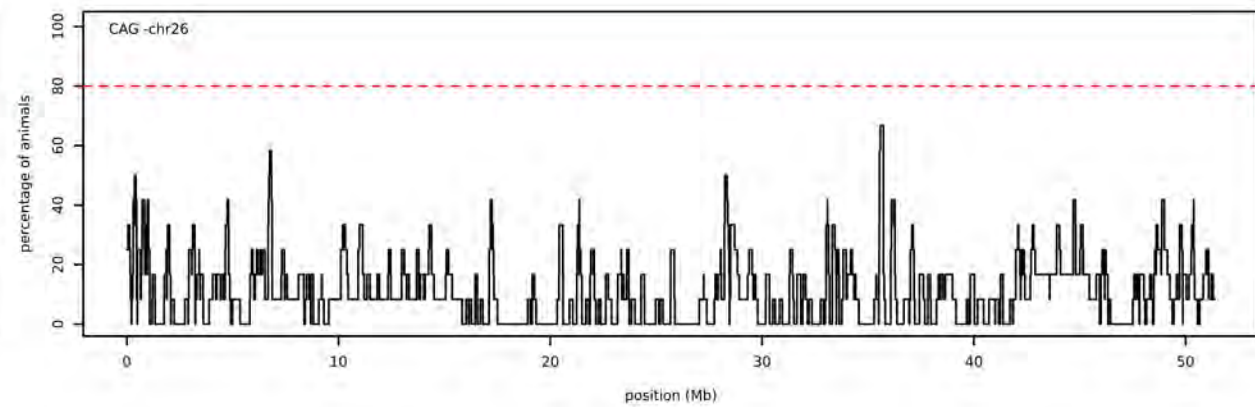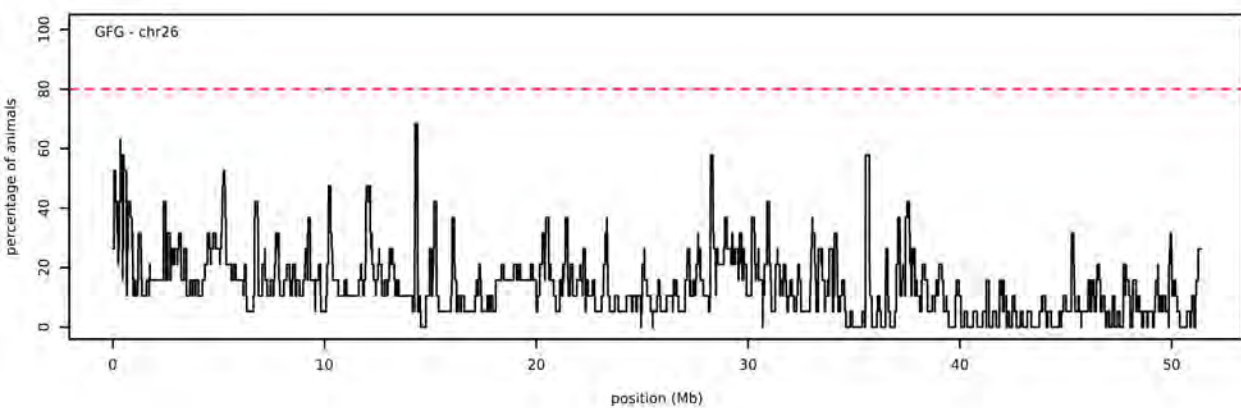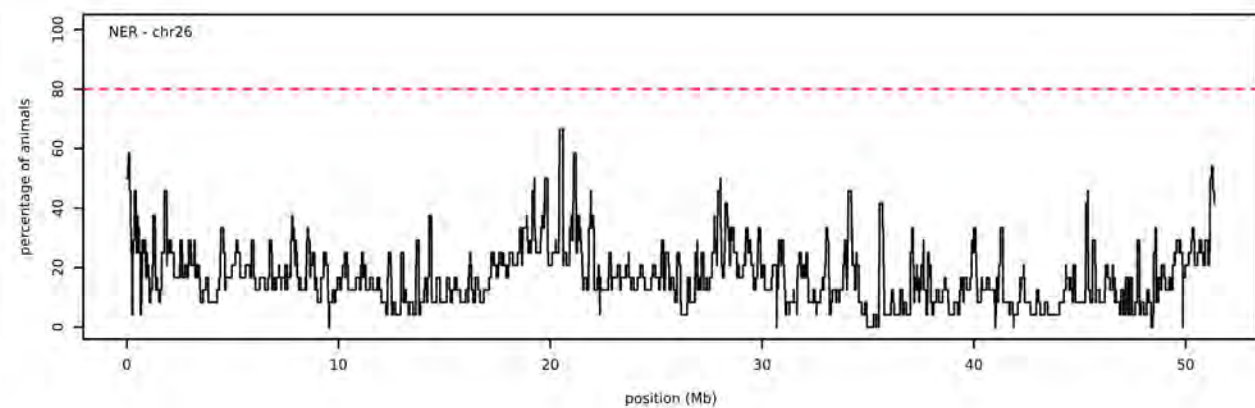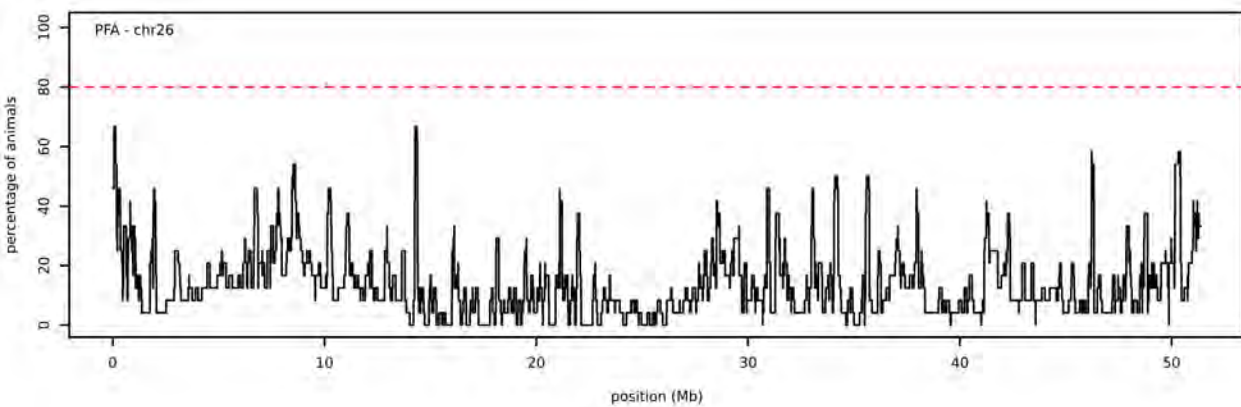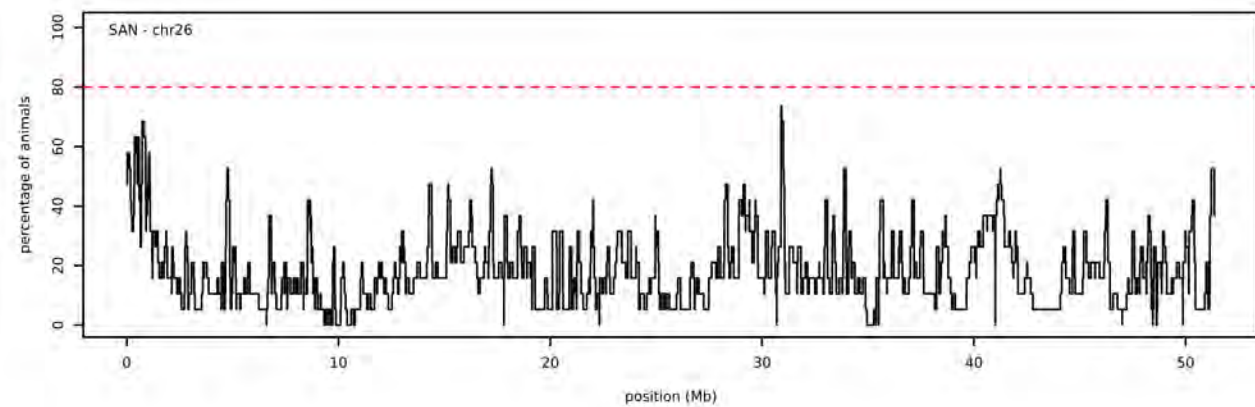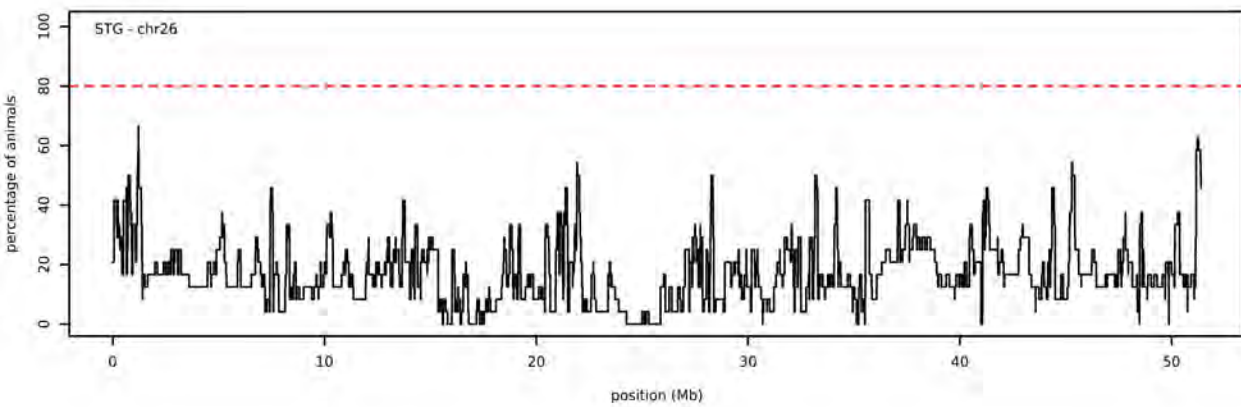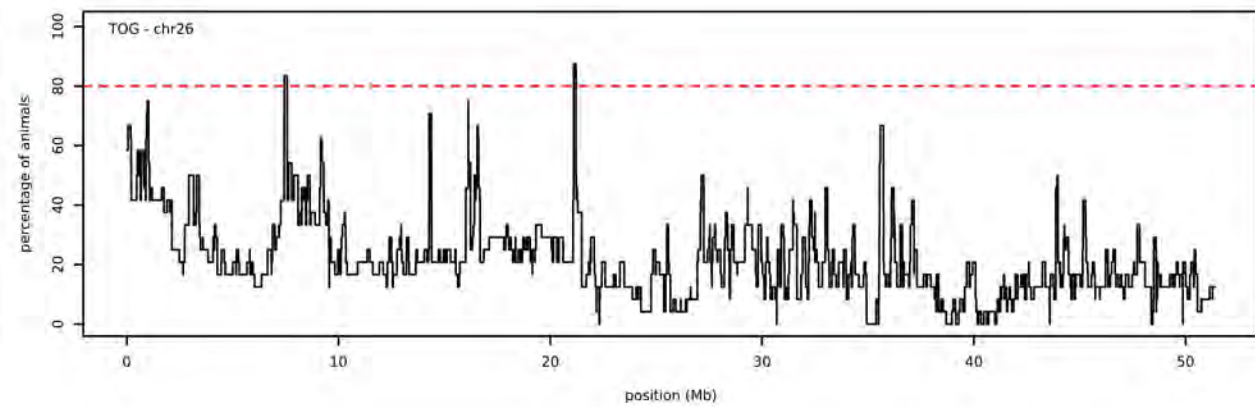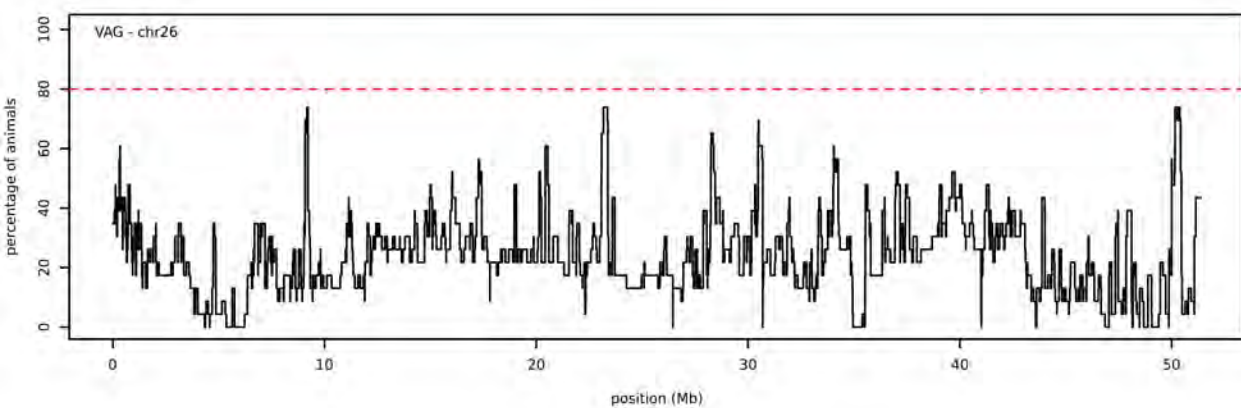

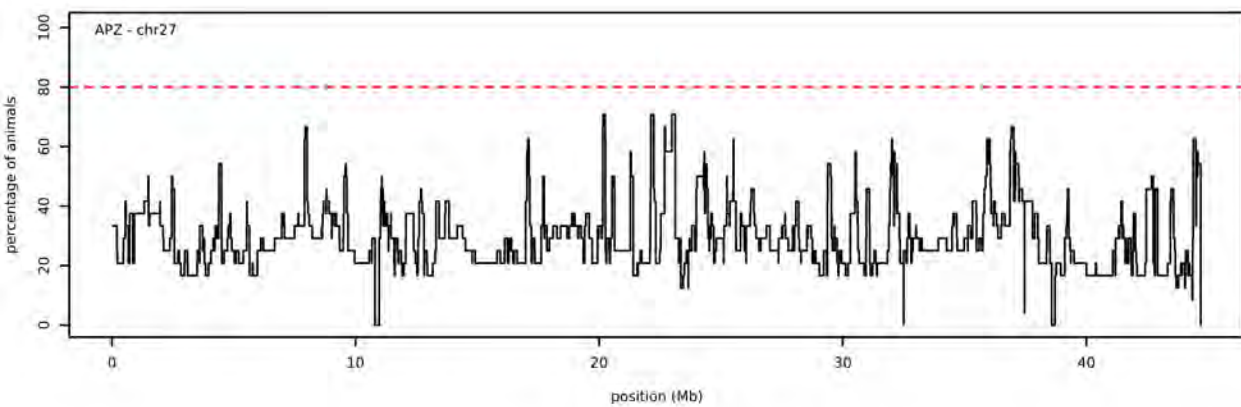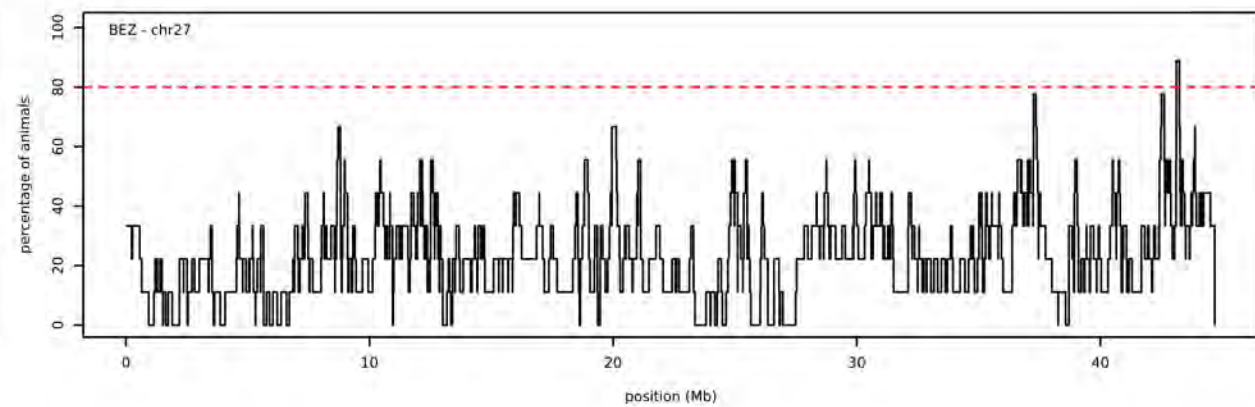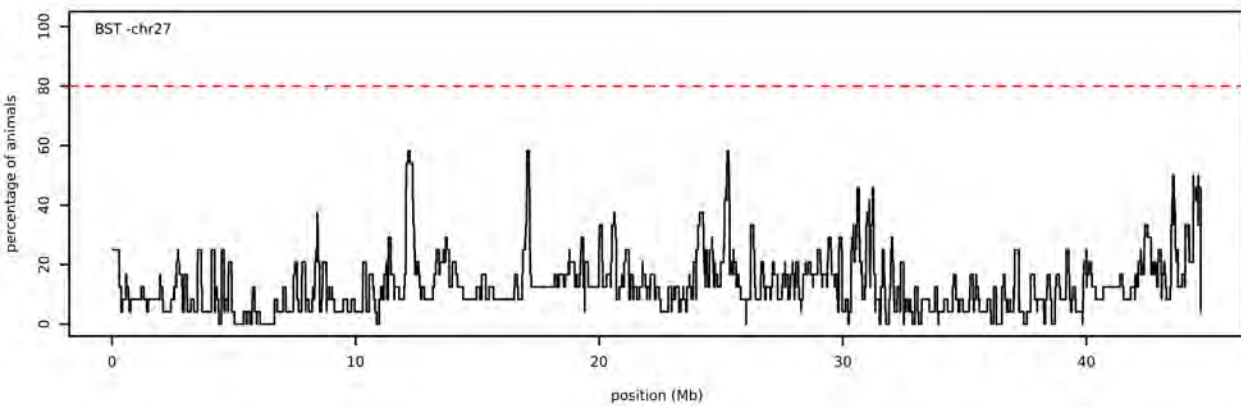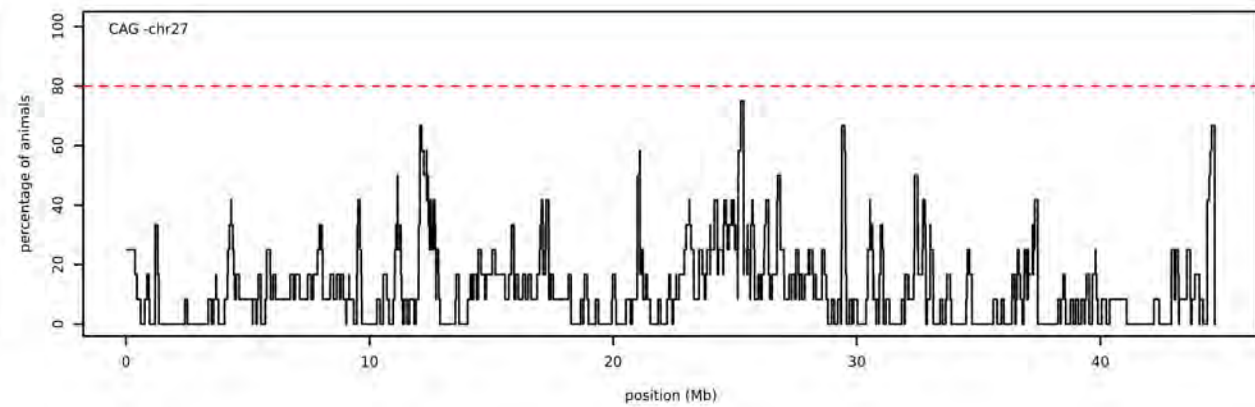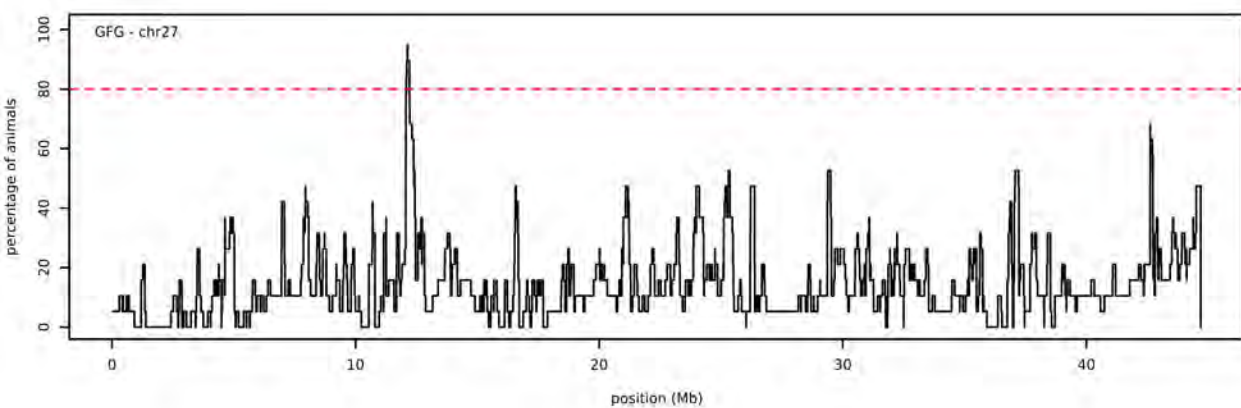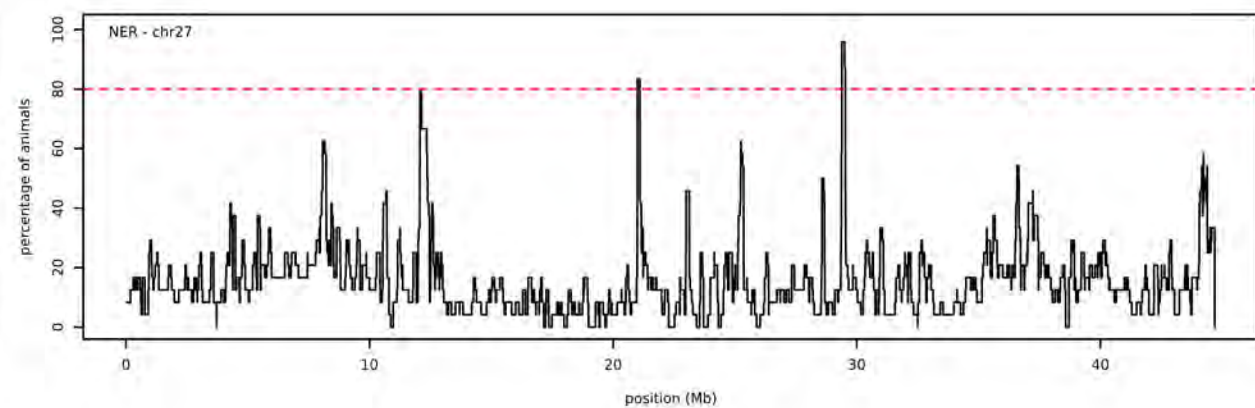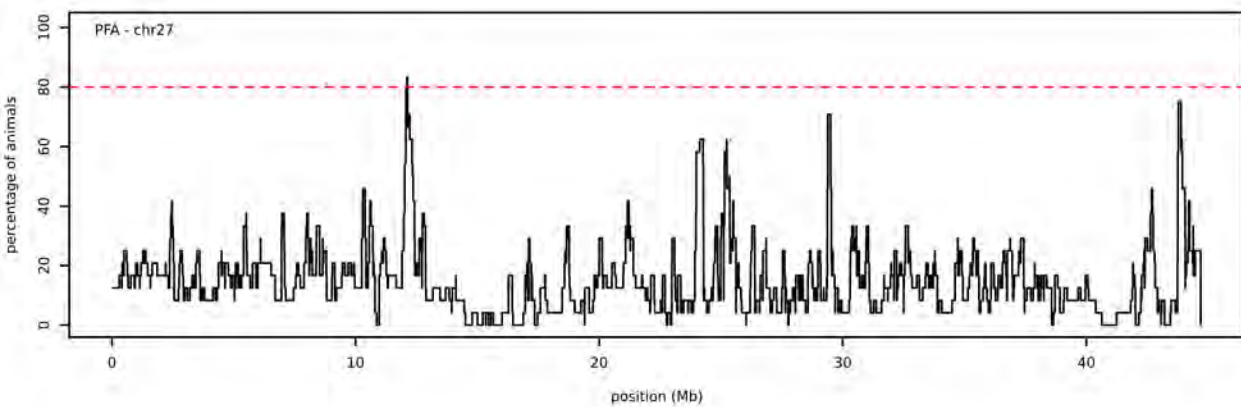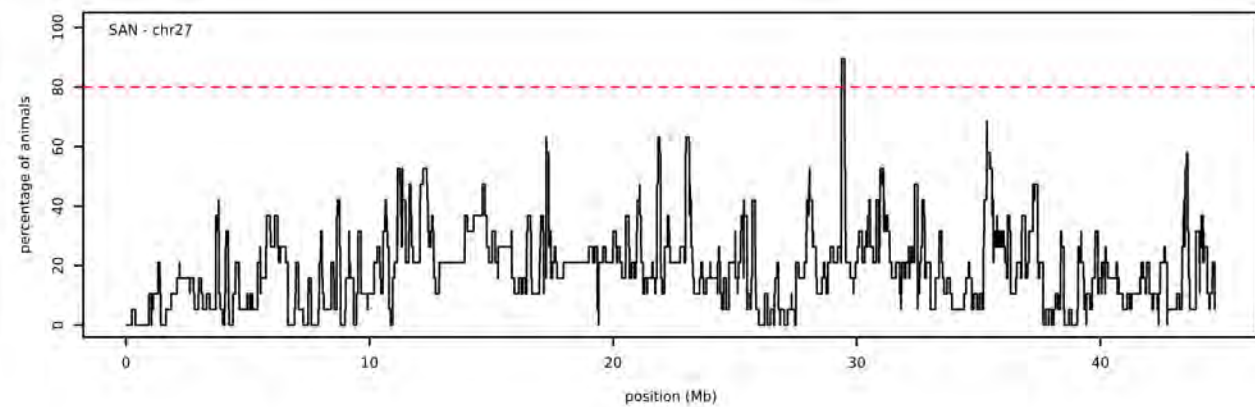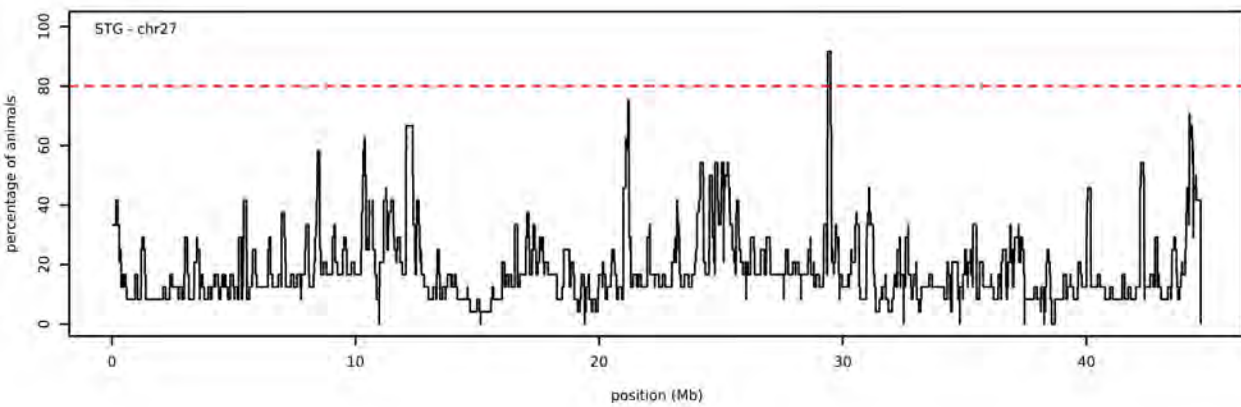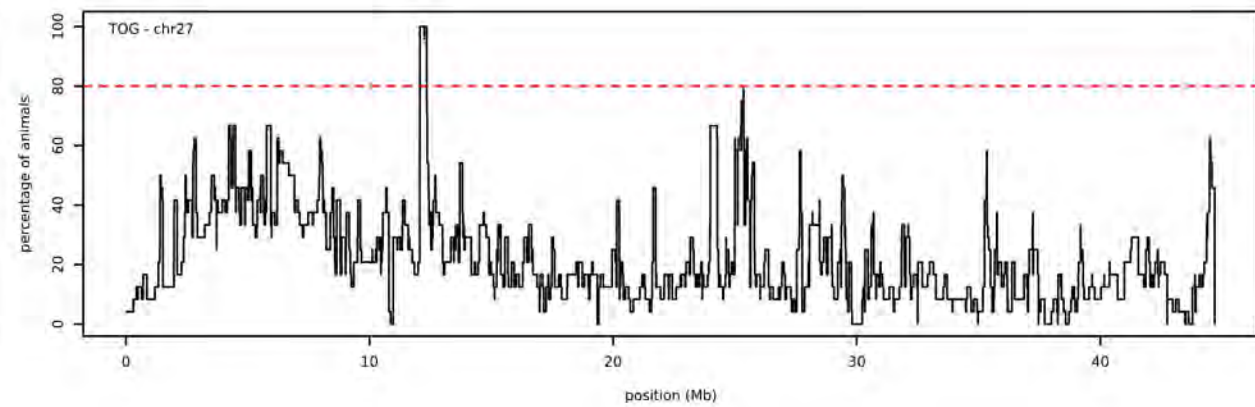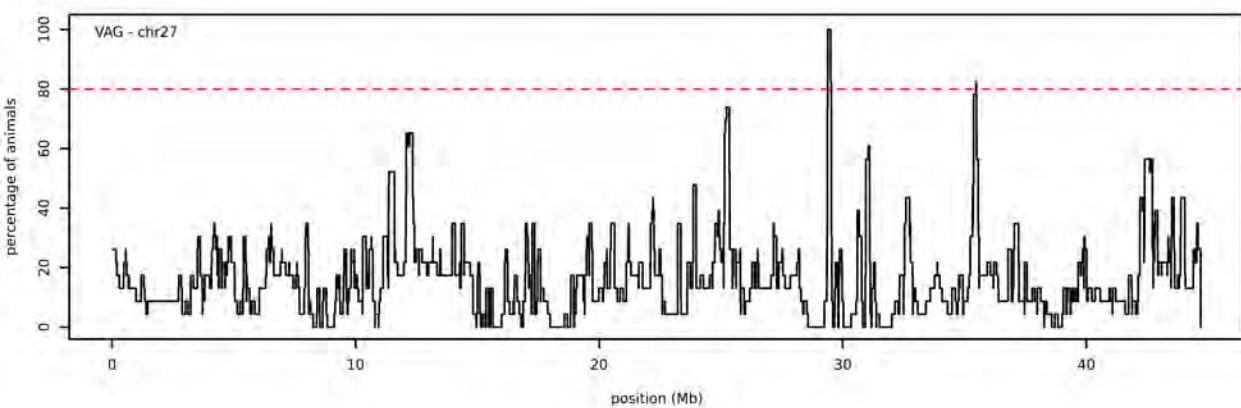

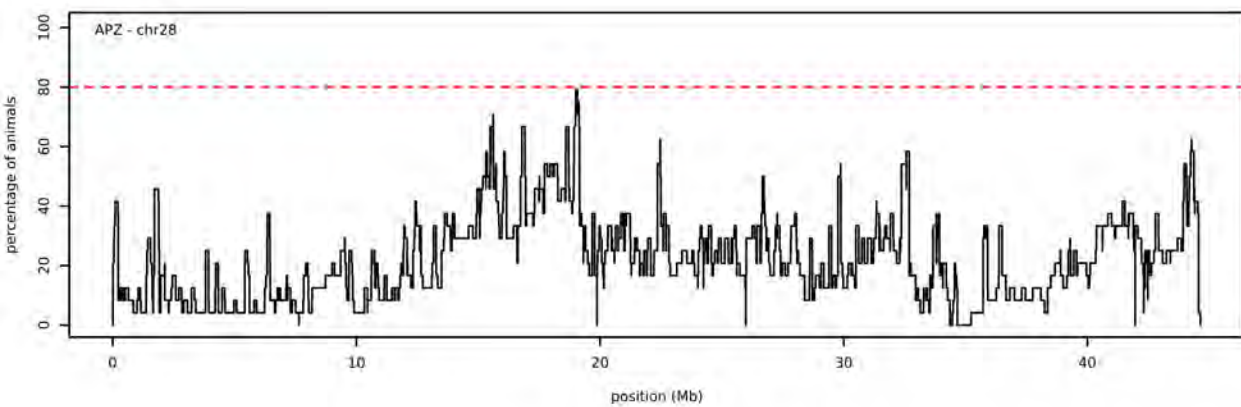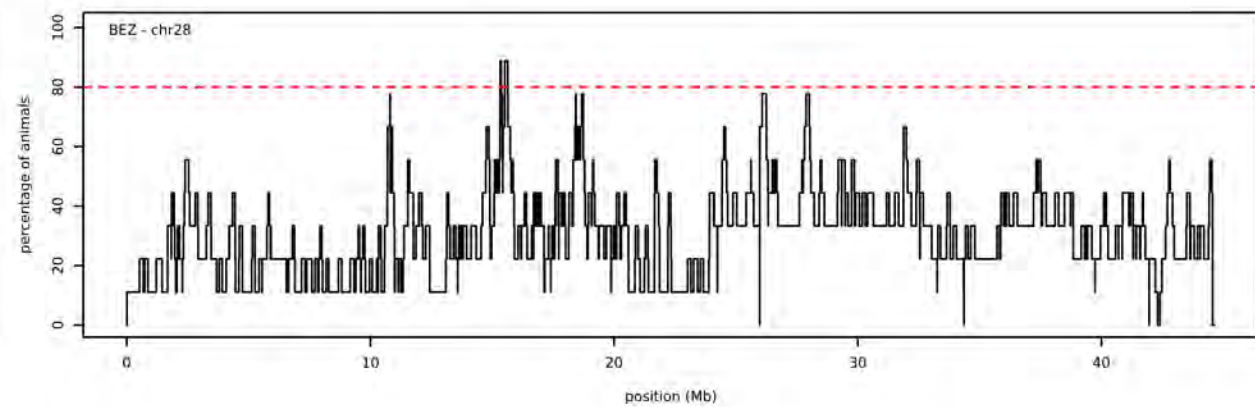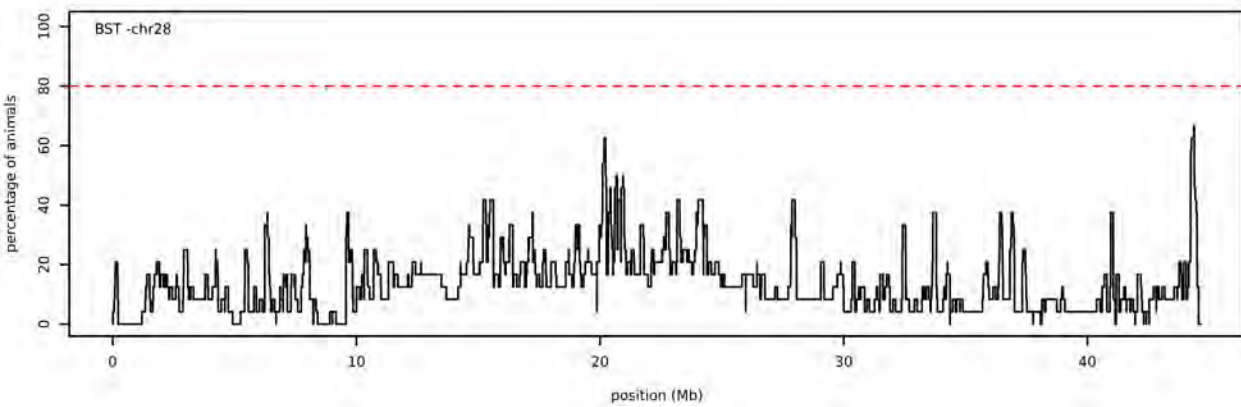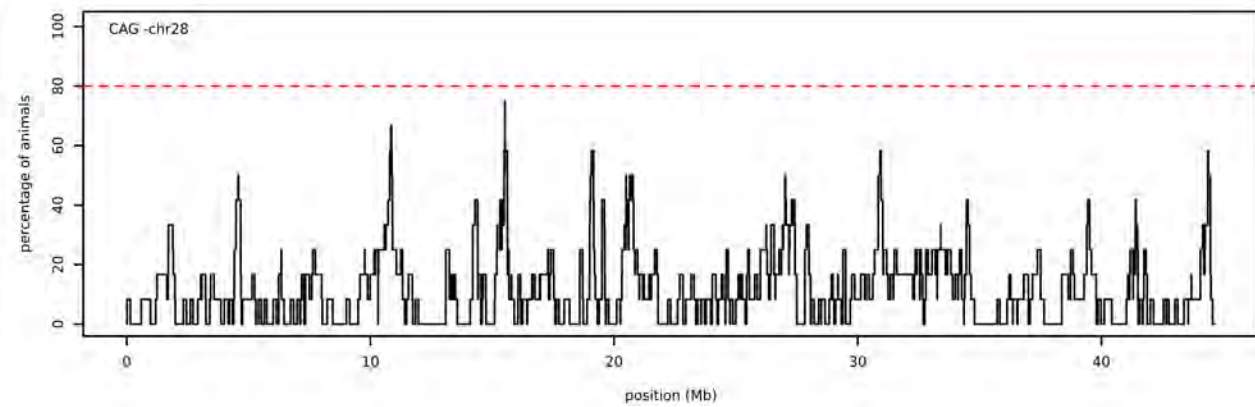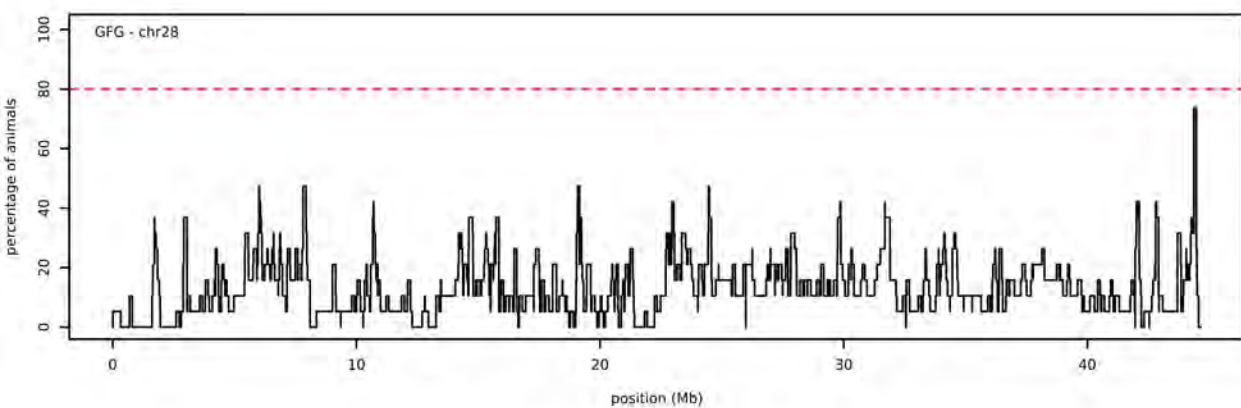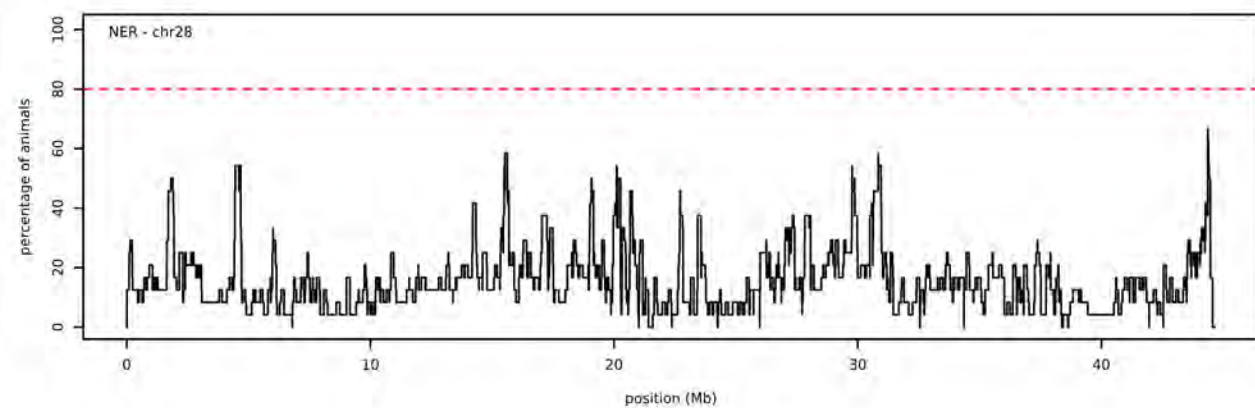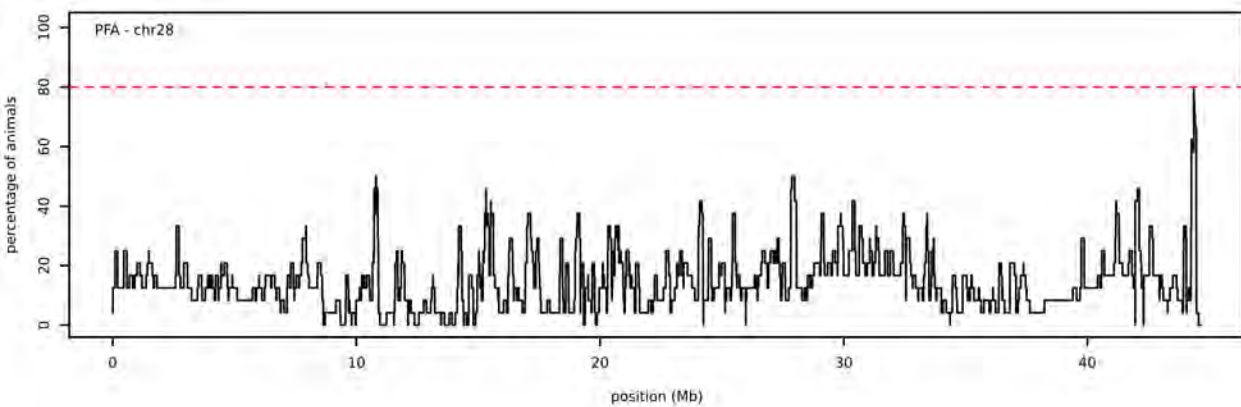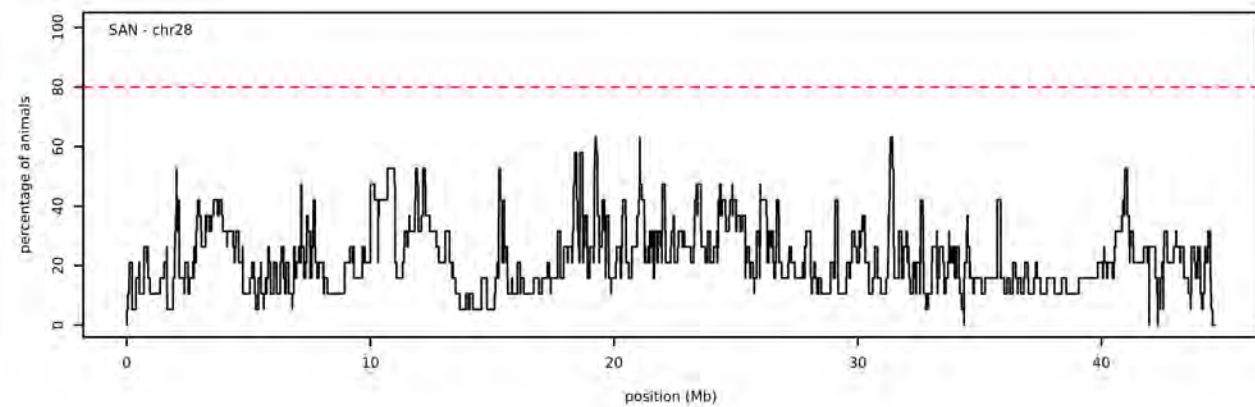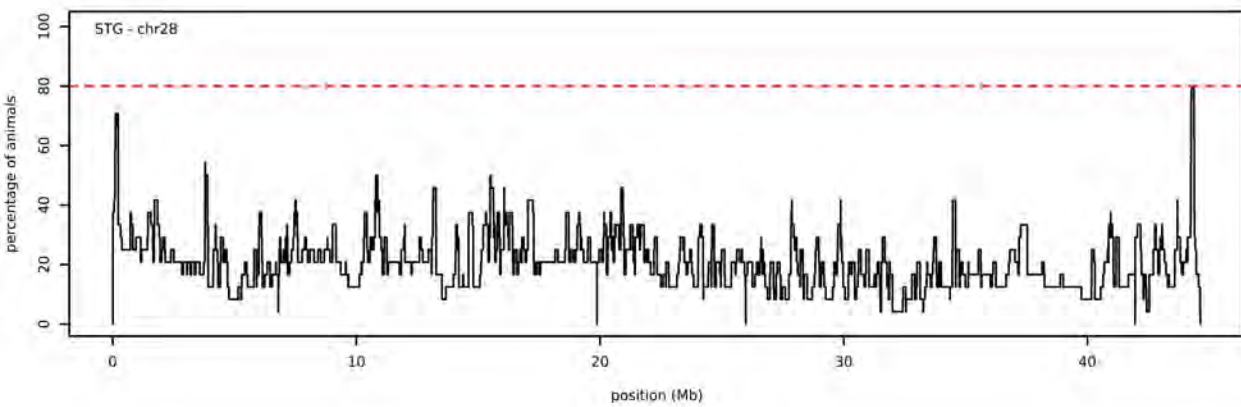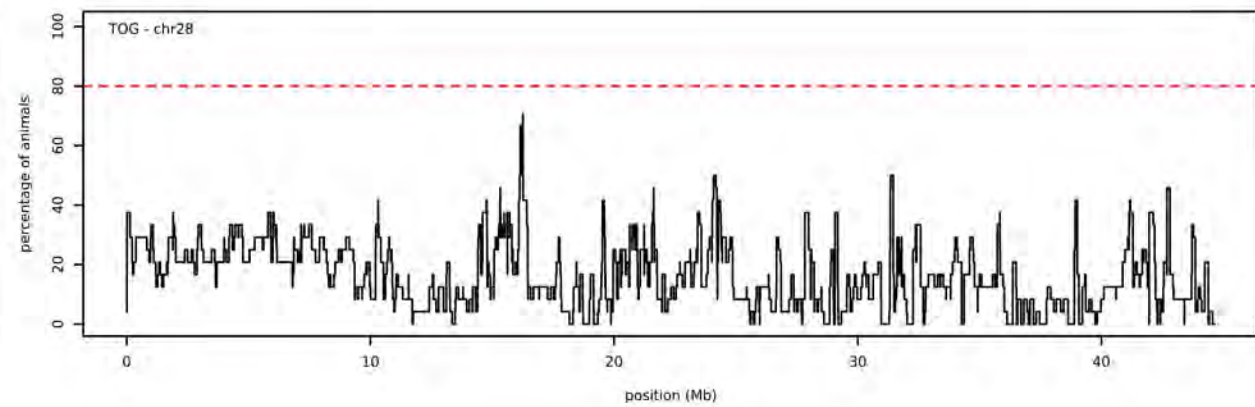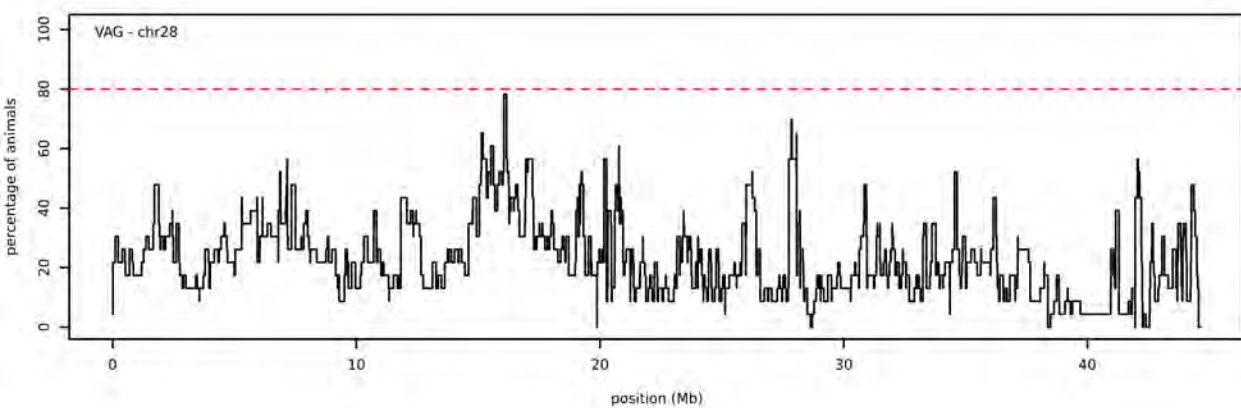

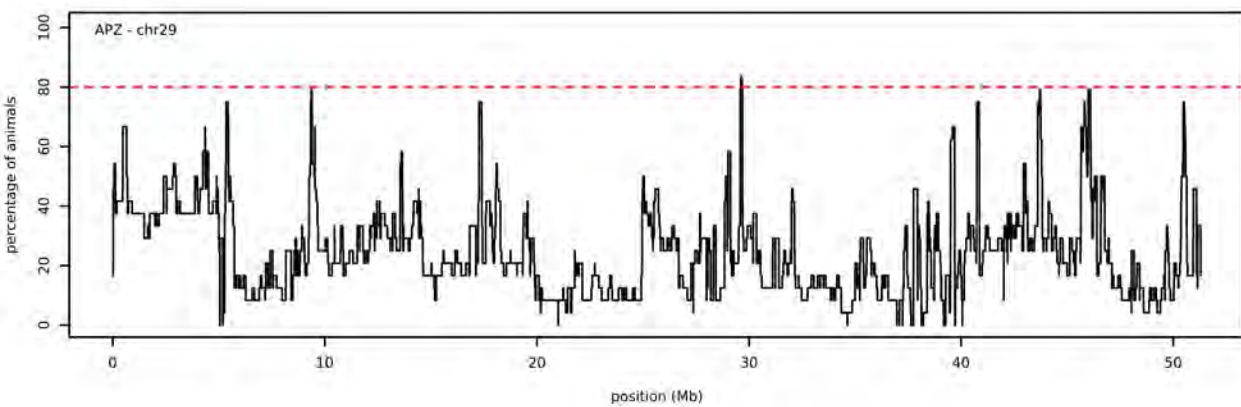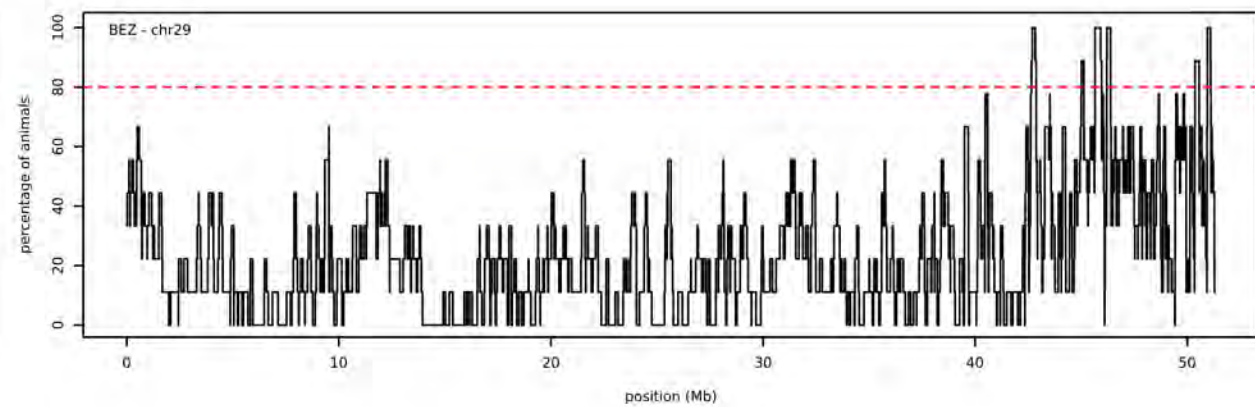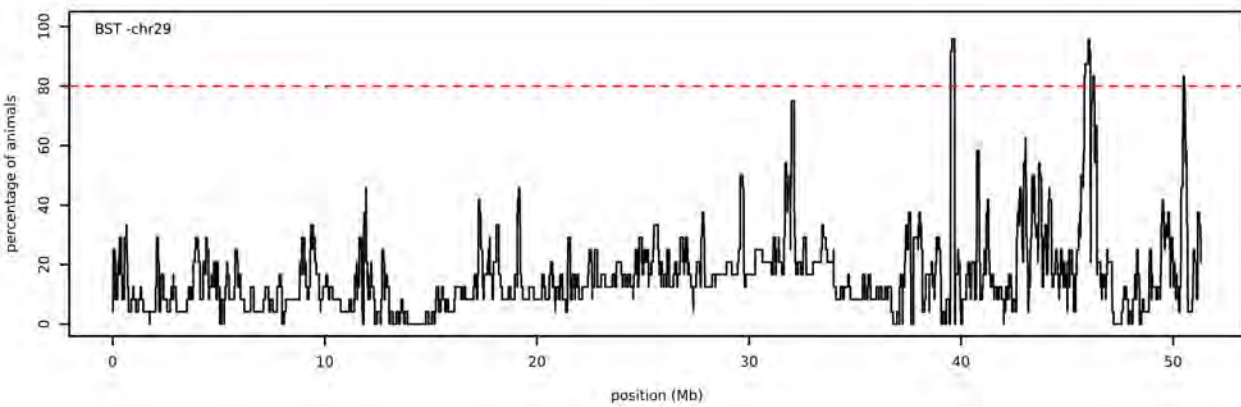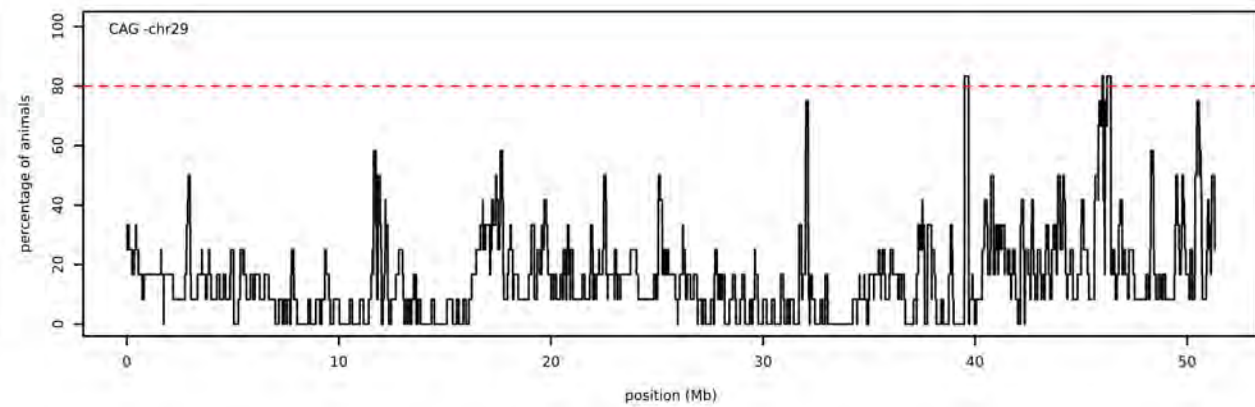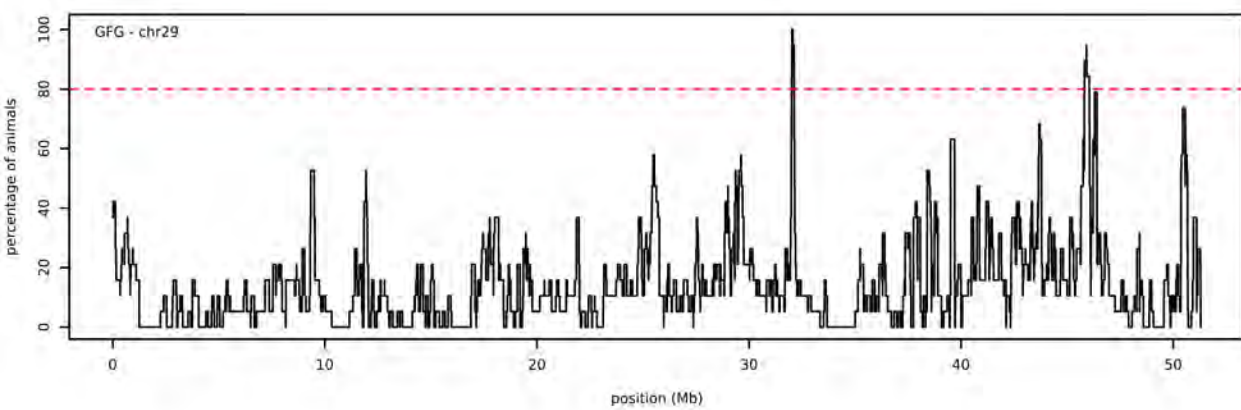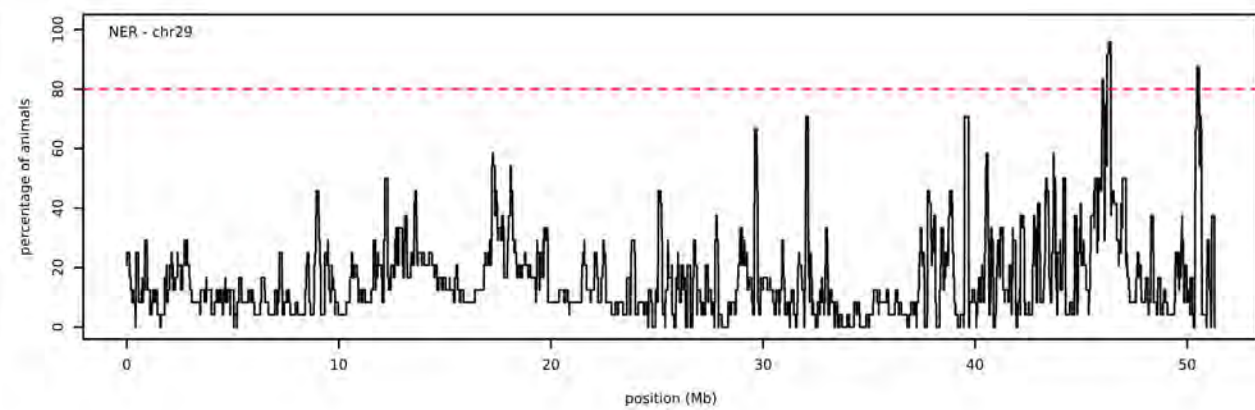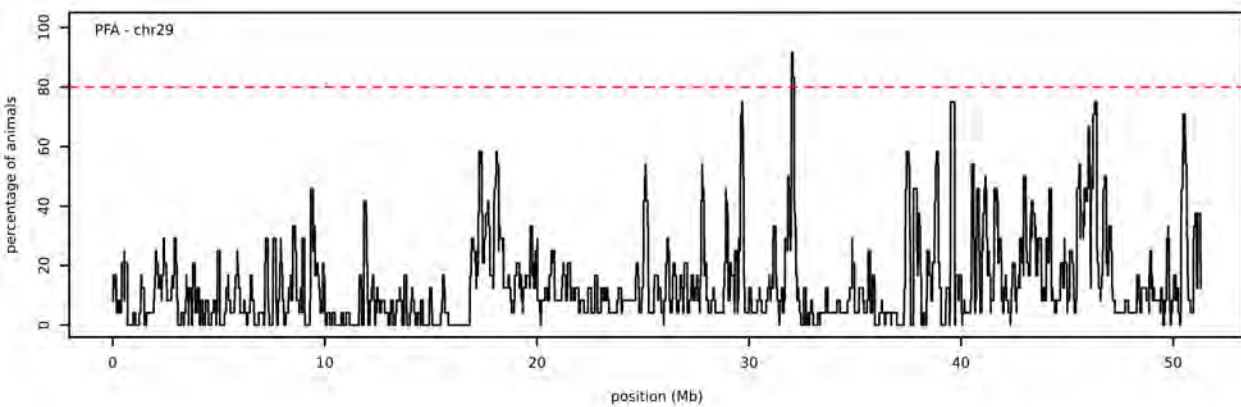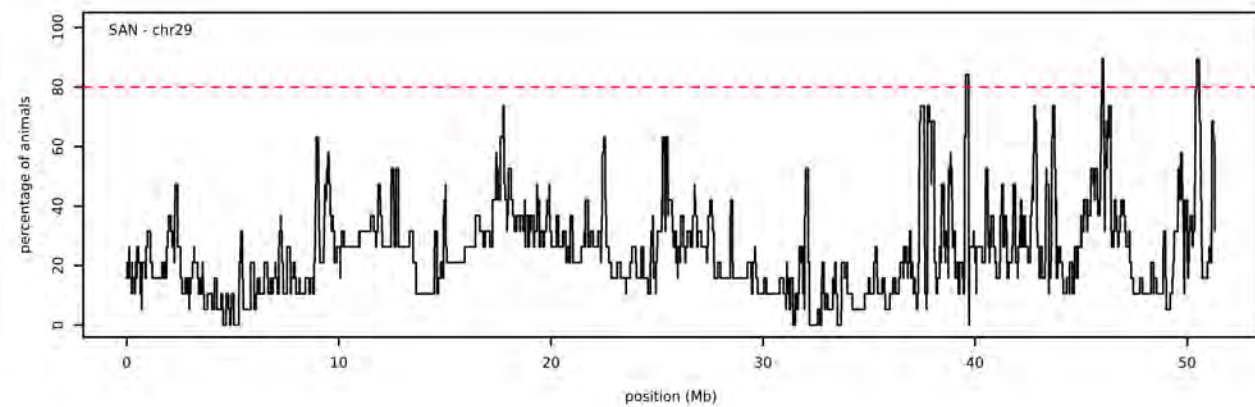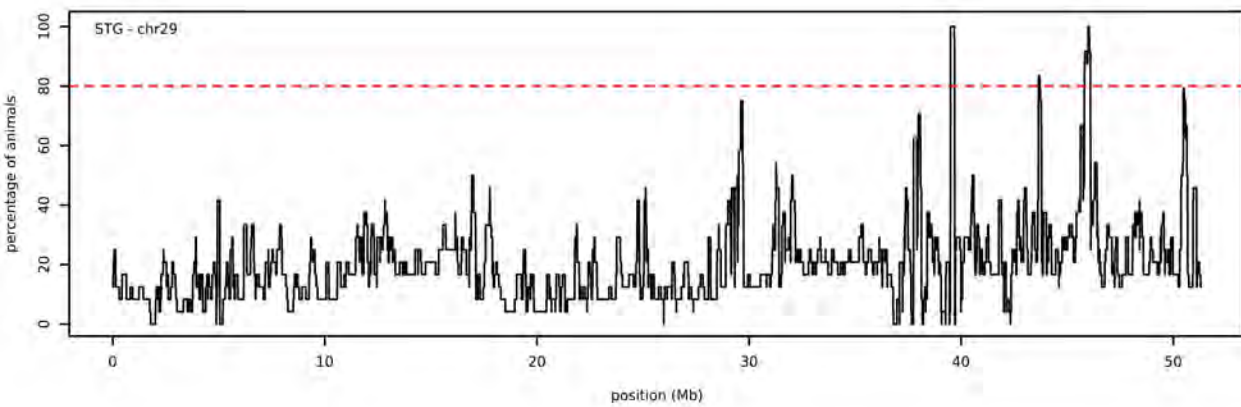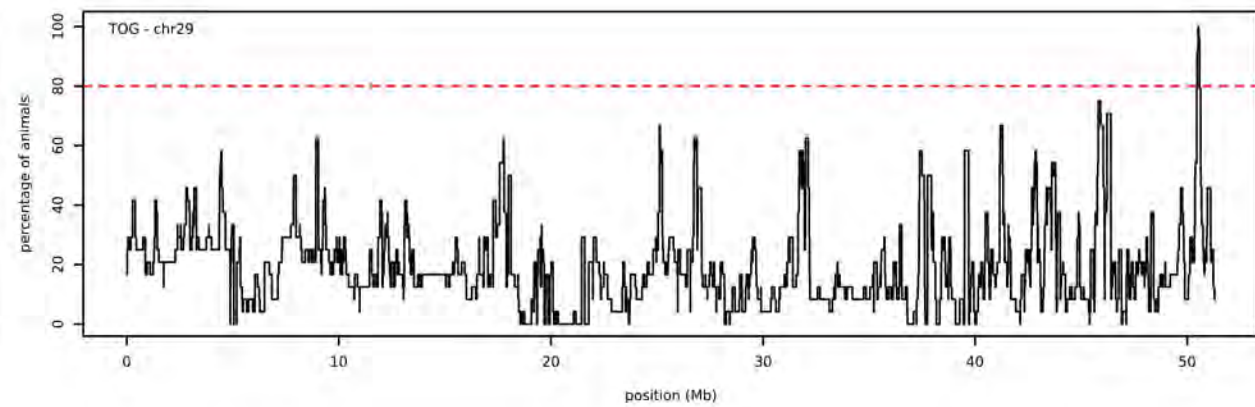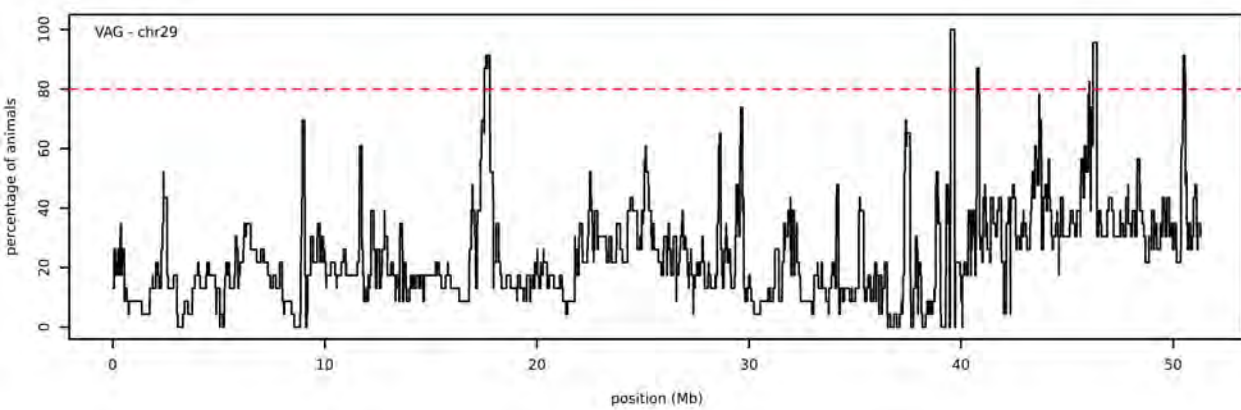

Supplement: Supplementary file 3 — Additional file 3: Figure S6. Manhattan plots representing the breed-wise average fraction of goats having a given SNV in a ROH for the 29 autosomes. The threshold (red dashed line) was set to 80%. SNVs that are above the threshold describe ROH islands. [file 12711_2022_695_MOESM3_ESM.pdf]
